# Effects of dietary *n-3-*PUFA supplementation, post-insemination plane of nutrition and pregnancy status on the endometrial transcriptome of beef heifers

**DOI:** 10.1038/s41598-020-77604-y

**Published:** 2020-11-27

**Authors:** Carla Surlis, Paul Cormican, Sinead M. Waters, Patrick Lonergan, Kate Keogh, David N. Doyle, David A. Kenny

**Affiliations:** 1 Animal and Grassland Research and Innovation Centre, Teagasc, Grange, Dunsany, Co. Meath Ireland; 2 School of Agriculture and Food Science, University College Dublin, Belfield, Dublin 4, Ireland

**Keywords:** Molecular biology, Transcription, Transcriptomics

## Abstract

Supplementation of cattle diets with *n-3*-polyunsaturated fatty acids (PUFA) can improve reproductive efficiency. Conversely, short-term fluctuations in feed supply can impact pregnancy establishment. The objectives of this study were to examine the effects of (1) dietary supplementation with *n-3-*PUFA and (2) post-insemination plane of nutrition on the endometrial transcriptome. Beef crossbred heifers were offered concentrate based diets fortified with *n-3*-PUFA (PUFA; n = 32) or not (CONT; n = 28) for 30 days prior to breeding at a synchronised oestrous. Following artificial insemination, heifers were allocated within treatment to either a high or low plane of nutrition. Heifers were maintained on these diets for 16 days following which endometrial tissue was harvested at slaughter for subsequent RNAseq analysis. The influence of pregnancy status on the endomentrial transcriptome, within each dietary treatment group, was also examined. Post-insemination diet affected (*P* < 0.05) the endometrial transcriptome. Specifically, within *n-3-*PUFA-supplemented heifers, genes involved in embryonic development and mTOR signalling pathways, important in pregnancy establishment, were identified as differentially expressed. Results indicate that dietary supplementation of cattle diets with *n-3-*PUFA may have a positive effect on the expression of key fertility-related genes and pathways, during the critical window of maternal recognition of pregnancy, particularly where animals are underfed.

## Introduction

Reproductive performance is influenced by many factors, including those available for manipulation through management, such as diet^1^. Supplementing diets with the *n-3* polyunsaturated fatty acids (PUFA), eicosapentaenoic acid (EPA) and docosahexaenoic acid (DHA), has been shown to enhance a range of beneficial traits in cattle, including evidence for improved reproductive status^2^. Optimum profitability within beef herds relies on a compact calving interval, which in turn is reliant on successful establishment and maintenance of pregnancy^3^. Following insemination, the greatest increment of reproductive wastage in cattle is due to early embryonic loss, with approximately 80% of these losses occurring prior to Day 14–16 post-insemination^4^. Maternal recognition of pregnancy is arguably the most critical phase of pregnancy in ruminants, occurring immediately prior to attachment of the conceptus (embryo and associated extraembryonic tissues) to the uterine endometrium^5,6^. Supplementation of cattle diets with *n-3* PUFA has been postulated to aid with maternal recognition of pregnancy and implantation of the conceptus through suppression of uterine secretion of luteolytic prostaglandin F2α (PGF2α), with a number of studies providing evidence for inhibition of uterine PGF2α by EPA and/or DHA, both in vitro and in vivo^7–11^.

While many studies have examined the effects of various fatty acid-based supplements on aspects of the reproductive process in dairy cows^12–14^, there have been few reports of their effects in beef cattle. Differences in the length of the post-partum period and in physiology between dairy and beef cows during this period^15^, such as body condition score, means that a different and possibly more beneficial effect of dietary manipulation may be expected in beef cows. Despite clear evidence to suggest a direct effect on uterine functionality during the early embryogenesis period, we and others have failed to establish an effect of omega-3 PUFA supplementation on measures of embryo viability^16^ or indeed on pregnancy rate in beef cattle, suggesting that this may, in part, be due to their overall general positive metabolic energy status in comparison to their dairy counterparts^3^.

Fluctuations in dietary energy intake during early embryogenesis may impact upon embryo survival^3^. For example, in a study with beef heifers maintained on a high plane of nutrition at pasture, and subsequently offered either a high or sub-maintenance plane of nutrition immediately post AI, a close to 50% reduction in conception rate was reported in nutritionally restricted heifers following AI^17^.

In ruminants, as well as in all studied mammals, the preparation of the uterine luminal epithelium for trophectoderm attachment and implantation requires specific orchestrated alterations within the endometrium transcriptome. In particular, progesterone is critically important to this process. Progesterone stimulates and maintains endometrial function which is necessary for the subsequent growth of the conceptus, implantation, placentation and overall development to term. Similar changes take place within the endometrial transcriptome up to the time of maternal recognition of pregnancy, in both cyclic and pregnant cattle^18,19^. This suggests that the primary default role of the uterus is to prepare for pregnancy. It is only by approximately Day 16, when pregnancy recognition occurs in cattle^18,19^ that significant alterations are apparent within the endometrial transcriptome between cyclic and pregnant cattle.

Thus, the aim of this study was to examine the individual and interactive effects of plane of nutrition, dietary supplementation with *n-3* PUFA and pregnancy status on the global transcriptome of uterine endometrial tissue during the period of maternal recognition of pregnancy.

## Methods

### Animals and feeding regime—pre insemination

**Figure 1:**
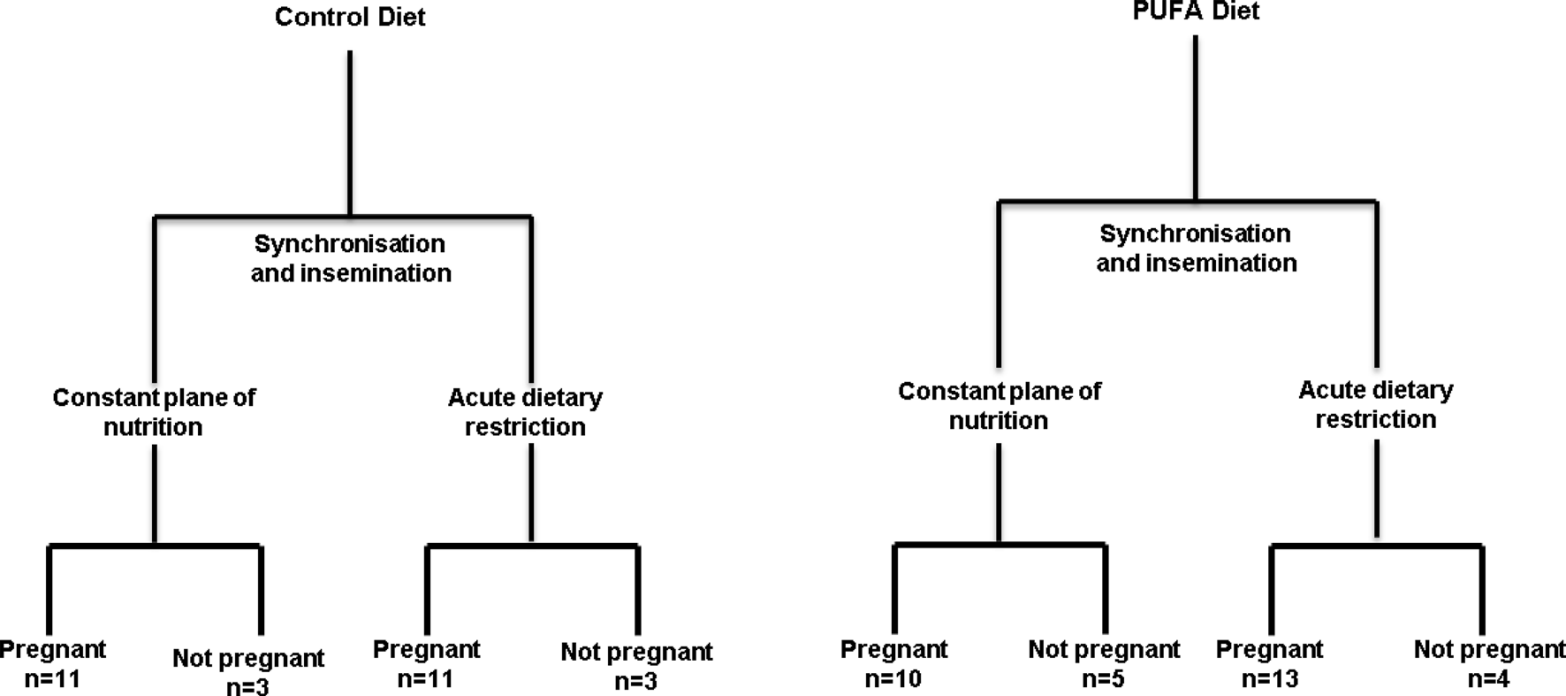
Animal model and experimental design. The overall 2 × 2 factorial design describing pre-insemination dietary groups and the diets fed post insemination is displayed graphically, including pregnancy outcomes in each animal group. Heifers received the pre-insemination diet for 30 days and the post-insemination diet for an additional 16 days after insemination. 14, 14, 15 and 17 heifers represented the, CON_Low, CON_High, PUFA_Low and PUFA_High dietary groups, respectively.

This study was conducted under licence, at University College Dublin’s Lyons Research Farm and at the Teagasc Research Centre, Athenry Co. Galway, in accordance with the Cruelty to Animals Act (Ireland 1876, as amended by the European Communities regulations 2002 and 2005) and the European Community Directive 86/609/EC and was sanctioned by the Animal Research Ethics Committee of University College Dublin. The work carried out here was part of a larger study^20^ which examined the effect of *n-3* PUFA supplementation and post-insemination plane of nutrition on pregnancy rate and embryo morphology as well as systemic concentrations of fatty acids, metabolic hormones and progesterone (see Fig. 1 for animal model and experimental design). The nutritional and reproductive management of the animals is explained in detail in the aforementioned study. Briefly, estrous cycles were synchronized in reproductively normal nulliparous crossbred beef heifers (n = 179) by intramuscular administration of two injections of 500 µg of PGF2α analogue (Cloprostanol, Estrumate, Schering-Plough Ltd., Hertfordshire, U.K.) 11 days apart. Animals were offered a barley straw (1.4 kg dry matter; DM), molasses (0.28 kg DM) and concentrate (5.50 kg DM)-based ration. This was supplemented with either a ruminally-protected source of palmitic acid (Palmit80; Trouw Nutrition, Belfast, Northern Ireland) which acted as the control diet (Control), or a high *n-3* PUFA diet (*n-3* PUFA) consisting of 275 g of a partially rumen-protected *n-3* PUFA (EPA: DHA; 1.5: 1)-enriched supplement, based on fish oil (Trouw Nutrition, 36 Ship Street Belfast, BT15 1JL, Northern Ireland). Concentrations of fish oil in the total DM offered were 0 for the control and 4.15% for the *n-3* PUFA diet. Both diets were formulated to be isonitrogenous and isolipidic (8% added lipid DM). Ingredient composition and the chemical analysis of the treatment diets, including the fatty acid concentration has been outlined in^20^. Overall, there were four dietary treatments (pre and post breeding combinations) viz. CON_Low, CON_High, *n-3* PUFA_Low and *n-3* PUFA_High. All heifers received the pre-breeding diets for 30 days prior to induced oestrus/insemination. All heifers were checked for ovulation and insemination subsequently performed, on average, 12 h after detection of estrus. Semen from a single high fertility sire was used for all artificial inseminations (AI).

### Post insemination experimental diets

On the day of insemination (Day 0), heifers were blocked within *n-3* PUFA pre-insemination diet and assigned to one of two post-insemination diets: (1) remain on their pre-insemination high dietary plane of nutrition (High) or (2) restricted to 0.6 × estimated maintenance energy requirements (National Academies of Sciences, Engineering, and Medicine, 2016; Low)^20^. The latter group received a total of 2 kg DM concentrate daily, which included the same level of lipid supplement as that offered pre-insemination, together with 0.85 kg DM barley straw^20^. Specifically, 14, 14, 15 and 17 heifers represented the, CON_Low, CON_High, PUFA_Low and PUFA_High dietary groups, respectively. The experiment was constructed as a 2 × 2 factorial arrangement of treatments. Heifers were maintained on their assigned diets until slaughter and embryo recovery on Day 16 post insemination^20^ (Fig. 1).

### Post-mortem sample collection (Day 16)

A representative sub-sample of heifers from within each of the four treatment groups were slaughtered at an EU-licenced abattoir on Day 16 post-AI. Specifically, 14, 14, 15 and 17 heifers from the, CON_Low, CON_High, *n-3* PUFA_Low and *n-3* PUFA_High dietary groups, respectively were harvested. Reproductive tracts were recovered and transported on ice to the laboratory. The uterine horns were trimmed free of excess tissue and flushed with 100 ml of phosphate-buffered saline (PBS), containing 5% fetal calf serum. This was injected into the uterine horn at the uterotuberal junction contralateral to the corpus luteum (CL) and massaged carefully through an incision in the horn ipsilateral to the CL. Pregnancy was confirmed by locating a conceptus under a stereomicroscope. Within the control fed heifers, 11 heifers on the constant (High) plane of nutrition and 11 on the restricted (Low) plane of nutrition were pregnant, while 3 were not pregnant in both groups. In PUFA-supplemented heifers on the constant plane of nutrition, 10 were pregnant with 5 not pregnant. In the PUFA-supplemented restricted group, 13 heifers were pregnant versus 4 not pregnant. Endometrial tissue from the antimesometrial border of the uterine horn ipsilateral to the CL was dissected from the myometrium as described previously^15^, snap frozen in liquid nitrogen and stored at – 80 °C for subsequent analysis.

### RNA isolation and purification

RNA was isolated from all uterine endometrial tissue samples using the Qiagen RNeasy mini kit (Qiagen, Manchester, UK) according to the manufacturer’s instructions. For each isolation, approximately 60 mg of frozen tissue was used. The amount of RNA recovered was determined by measuring the absorbance at 260 nm on a Nanodrop spectrophotometer ND-1000 (Nanodrop Technologies, DE, USA). Additionally the quality of the isolated RNA was evaluated through determining the RNA integrity number (RIN) using the RNA 6000 Nano Lab Chip kit (Agilent Technologies Ireland Ltd., Dublin, Ireland) on an Agilent Bioanalyser 2100. RNA samples with RIN values of between 8 and 10 were deemed to be of sufficiently high quality for subsequent RNAseq analysis.

### cDNA library preparation and sequencing

cDNA libraries were prepared from high quality RNA samples using the Illumina TruSeq RNA sample prep kit following the manufacturer’s instructions (Illumina, San Diego, CA, USA). For each sample, 3 µg of RNA was used for cDNA preparation. Individual cDNA libraries prepared from mRNA were validated through the DNA 1000 Nano Lab Chip kit on the Agilent Bioanalyser 2100 ensuring that library fragment size was ~ 260 bp and library concentration was > 30 ng/µl. Individual RNAseq libraries were then sequenced on an Illumina HiSeq 2000 sequencer, resulting in 100 bp single end sequencing reads.

### RNAseq data analysis

**Figure 2:**
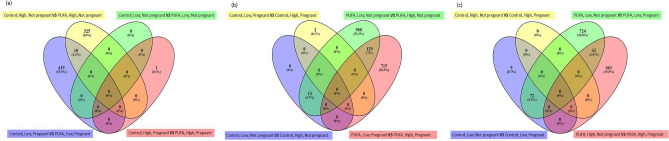
Visual representation of numbers of differentially expressed genes within all comparisons. Figure highlights the number of DEG that were identified across all comparisons. (**a**) Venn diagram depicting DEG identified when investigating the effect of *n-3* PUFA supplementation on uterine endometrial gene expression. (**b**) Venn diagram highlighting DEG across all four comparisons investigating the effect of modulation of plane of nutrition during peri-insemination period on uterine endometrial gene expression. (**c**) Venn diagram highlighting DEG across all four comparisons elucidating the effect of pregnancy status on uterine endometrial gene expression. 14, 14, 15 and 17 heifers represented the, CON_Low, CON_High, PUFA_Low and PUFA_High dietary groups, respectively. Venn diagram were generated using Venny 2.1 (http://bioinfogp.cnb.csic.es/tools/venny/)^21^.

Raw sequence reads were first checked for quality using FASTQC software (version 0.10.0). TopHat (v2.0.9) was used to align sequencing reads to the UMD3.1 version of the bovine reference genome. The number of sequence reads aligned to all protein-coding genes of the bovine genome was then calculated using the HTSeq (v0.5.4p5) (http://pypi.python.org/pypi/HTSeq) software package. The resultant read counts for each sample were subsequently collated into a single file and used to determine differentially expressed genes (DEG). To determine genes that were differentially expressed as a consequence of treatment the R (v2.14.1) Bioconductor package, EdgeR (v3.4.1) was applied to read counts for each sample. Resultant DEG are defined as having a Benjamini and Hochberg false discovery rate of < 0.1% and a fold change > 1.5.Venn diagrams to highlight evidence of interactions between *n-3* PUFA supplementation, post-insemination plane of nutrition and pregnancy status were constructed using the online resource Venny 2.1 (http://bioinfogp.cnb.csic.es/tools/venny/)^21^. Venn diagrams are shown for all comparisons in Fig. 2.

### Pathway analysis

The online software tool BioMart (www.ensembl.org/biomart.martview) was used to convert bovine gene IDs to their human orthologs. The resultant set of DEG was analysed using KEGG pathways for under/over-representation (www.genome.jp/kegg/pathway.html). To examine the molecular functions and genetic networks, the RNAseq data were further analysed using Ingenuity Pathway Analysis (IPA; v. 8.8, Ingenuity Systems, Mountain View, CA; http://www.ingenuity.com), a web-based software application that enables identification of over-represented biological mechanisms, pathways and functions most relevant to experimental datasets or genes of interest^22^. Network eligible molecules derived from these datasets were overlaid onto a global molecular network developed from information contained in the Ingenuity Knowledge Base. Networks of these molecules were then generated algorithmically based on their connectivity. The network score is based on the hypergeometric distribution and is calculated with the right-tailed Fisher’s Exact Test. Additionally, canonical pathway analysis identified the pathways from the IPA library of canonical pathways that were most significant to the dataset in terms of the ratio of the number of molecules that mapped to the pathway from the dataset and a right-tailed Fisher’s exact t-test to determine the probability that the molecules mapped to the pathway by chance alone.

## Results

### Animal performance

The growth, endocrinological and reproductive responses of the heifers to the various dietary regimens employed have been previously reported^20^. Briefly, *n-3* PUFA supplementation increased plasma concentration of *n-3* PUFA and reduced plasma concentration of *n-6:n-3* PUFA. Plasma concentration of progesterone was increased (*P* < 0.01) from Days 7–16 in heifers on the low plane of nutrition post-insemination. Plasma concentration of IGF-1 was higher (*P* < 0.05) in *n-3* PUFA-supplemented heifers and those on a higher post-insemination diet. Endometrial tissue samples were harvested from 60 heifers in total for uterine RNAseq analysis (28 Control fed, 32 *n-3* PUFA; see Fig. 1). Specifically, 14, 14, 15 and 17 heifers represented the, CON_Low, CON_High, PUFA_Low and PUFA_High dietary groups, respectively.

### Differential gene expression

Identified DEG datasets were subjected to a number of a priori comparisons to examine the effects of (1) n-3 PUFA supplementation; (2) post-insemination plane of nutrition and (3) pregnancy status on differential gene expression profiles.

#### Effect of *n-3* PUFA supplementation

Numbers of DEG in each comparison carried out to elucidate the effect of *n-3* PUFA supplementation on endometrial tissue expression are highlighted in Fig. 2a. For non-pregnant heifers, there were no DEG identified between those offered the control *versus* the *n-3* PUFA supplement. The same dietary comparison for pregnant animals, however, revealed 429 DEG (225 present at lower levels of transcript abundance and 204 at higher levels in the control heifers; Supplementary Table S1).

Analysis of the effect of *n-3* PUFA supplementation when combined with a high plane of nutrition post-insemination revealed 335 DEG, with 257 present at lower levels and 78 present at higher levels of abundance in the control non-pregnant heifers compared with the *n-3* PUFA non-pregnant group (Supplementary Table S2). Comparison of tissues from pregnant control and *n-3* PUFA heifers resulted in only one DEG (*GJB2*), which was present at a higher abundance in the control compared to the *n-3* PUFA-supplemented heifers (Supplementary Table S3).

#### Effect of post-insemination plane of nutrition within control and *n-3* PUFA supplemented groups

In order to examine the effect of *n-3* PUFA supplementation when combined with post-insemination nutrition, comparisons were made within the control and *n-3* PUFA groups, for both pregnant and non-pregnant heifers. Numbers of DEG for each comparison are shown in Fig. 2b. Amongst non-pregnant control heifers, there were 13 DEG at lower abundance in heifers on the low plane of nutrition (Supplementary Table S4), while 2 DEG were present at lower abundance in control pregnant heifers (Supplementary Table S5).

Similar comparisons were carried out within the *n-3* PUFA supplemented group, with 1123 DEG identified between the low and high post-insemination plane of nutrition diets (762 decreased and 361 increased in abundance) (Supplementary Table S6). When comparing the effects of *n-3* PUFA supplementation and post-insemination plane of nutrition within pregnant animals, 502 DEG were present at lower expression and 342 DEG at higher levels in the low in comparison with the high plane of nutrition group (Supplementary Table S7).

### Interaction of *n-3* PUFA supplementation and post-insemination diet in pregnant animals

The next step was to identify if fluctuations in dietary nutrient intake during the peri-insemination period in *n-3* PUFA supplemented heifers led to alterations in the transcript abundance of critical genes. This comparison resulted in 1919 DEG in pregnant heifers, 955 present at lower and 964 at higher levels of abundance in the control animals (Supplementary Table S8).

#### Effect of pregnancy status on endometrial gene expression

In order to establish the effect of pregnancy status on uterine gene expression, comparisons were carried out across the various dietary groups between pregnant and non-pregnant heifers (Fig. 2c). There were 81 DEG reaching statistical significance between pregnant and non-pregnant control heifers on a low plane of post-insemination nutrition (76 at higher abundance and 5 at lower abundance in pregnant heifers) (Supplementary Table S9). Comparison of the pregnant and non-pregnant control heifers on a continuous high plane of post-insemination nutrition did not reveal any DEG. Comparison of pregnant versus non-pregnant *n-3* PUFA-supplemented heifers on the low plane of nutrition post-insemination resulted in 828 DEG, 620 present at lower levels and 208 at higher levels in the *n-3* PUFA supplemented non-pregnant heifers (Supplementary Table S10). Contrasting pregnant versus non-pregnant *n-3* PUFA supplemented heifers on the continuous higher plane of nutrition diet resulted in 493 statistically significant DEG, with 90 present at higher levels of abundance and 403 present at lower levels in the pregnant compared with non-pregnant heifers, respectively (Supplementary Table S11).

### Pathway analysis

#### Effect of *n-3* PUFA supplementation

**Figure 3:**
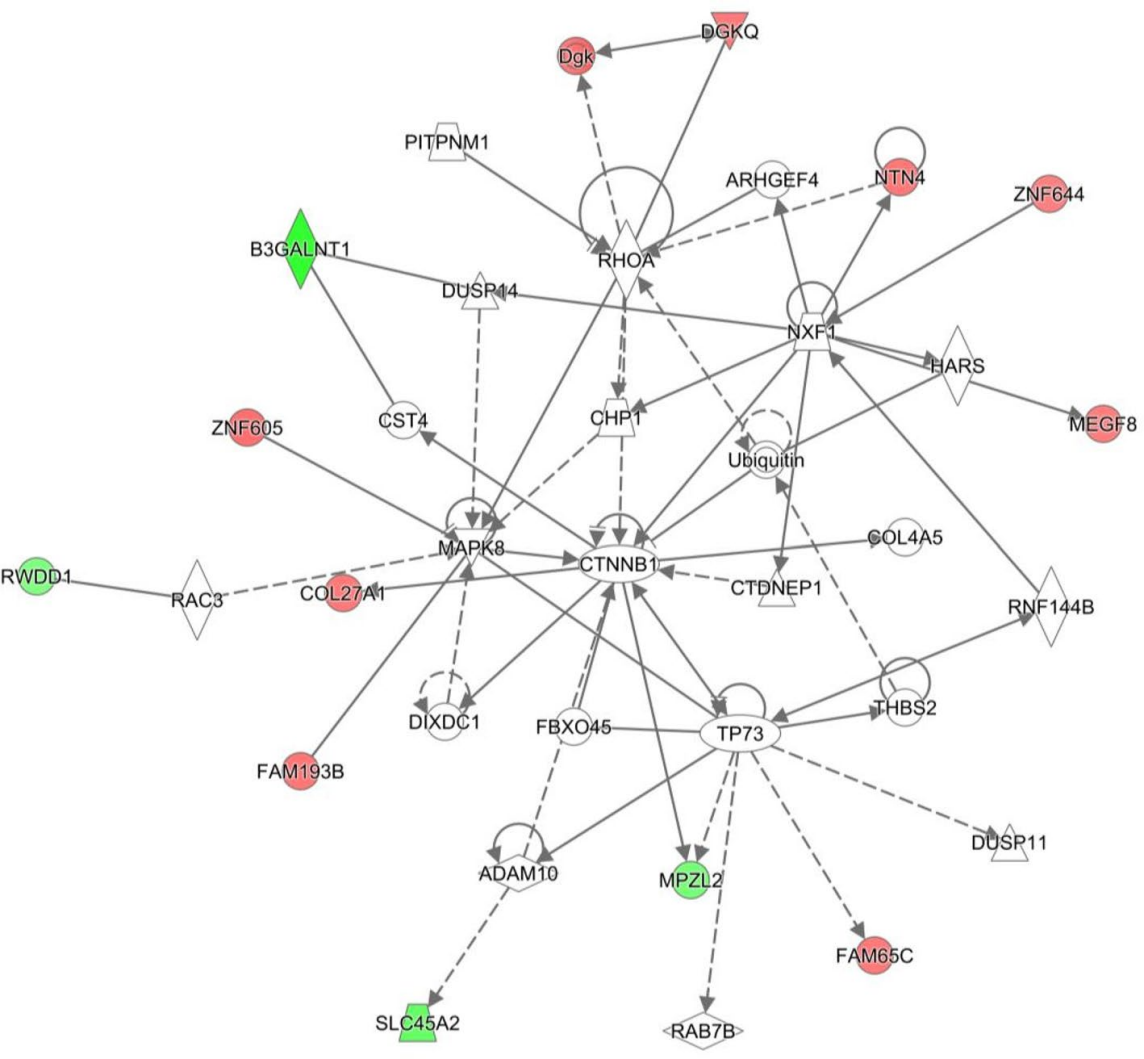
Embryonic Development, Nervous System Development and Function, Organ Development network in comparison of Control, pregnant, low diet versus *n-3* PUFA, pregnant, low diet. The network is displayed graphically as nodes (genes). The node colour indicates the expression of genes with red representing up-regulation and green indicating lower expression in the *n-3* PUFA supplemented pregnant heifers on the low plane of nutrition post-insemination, compared to the Control pregnant heifers on the low plane of nutrition. network displays nodes (genes/gene products) and edges (the biological relationship between nodes). The colour intensity of the nodes indicates the fold-change increase (red) or decrease (green) associated with a particular gene in control animals compared to *n-3* PUFA supplemented. A solid line indicates a direct interaction between nodes (genes/gene products), and a dashed line indicates an indirect relationship between nodes. The shape of the node is indicative of its function. The network image was generated through the use of IPA (QIAGEN Inc., https://www.qiagenbio-informatics.com/products/ingenuity-pathway-analysis)^22^.

The effect of *n-3* PUFA supplementation on both pregnant and non-pregnant heifers together with post-insemination plane of nutrition was examined using four separate comparisons within the treatment groups. Two of these, with a sufficient number of DEG, were further analysed using pathway analysis. Pregnant heifers offered the control or *n-3* PUFA supplemented diets and then assigned to a low post-insemination plane of nutrition yielded 429 DEG (Supplementary Table S1). Pathway analysis of these DEG resulted in a number of networks regarded as being biologically interesting from a cattle fertility perspective outlined in Supplementary Table S12. Particular networks of interest included embryonic development, nervous system development and function, organ development (Network 23) (Fig. 3). Enriched biological pathways for this comparison included eIF2 signalling, mTOR signalling, and embryonic development.

Non-pregnant control heifers on a high plane of nutrition compared to non-pregnant PUFA supplemented heifers on a high plane of nutrition resulted in 335 DEG. A total of 24 networks were identified within IPA for this comparison, with a varied range of functions presented including cellular function and maintenance (Network 1) and post-translational modification, cell–cell signalling and interaction (Network 6). Details of networks highlighting genes involved are accessible in Supplementary Table S12.

#### Effect of post-insemination plane of nutrition within control and *n-3* PUFA-supplemented groups

**Figure 4:**
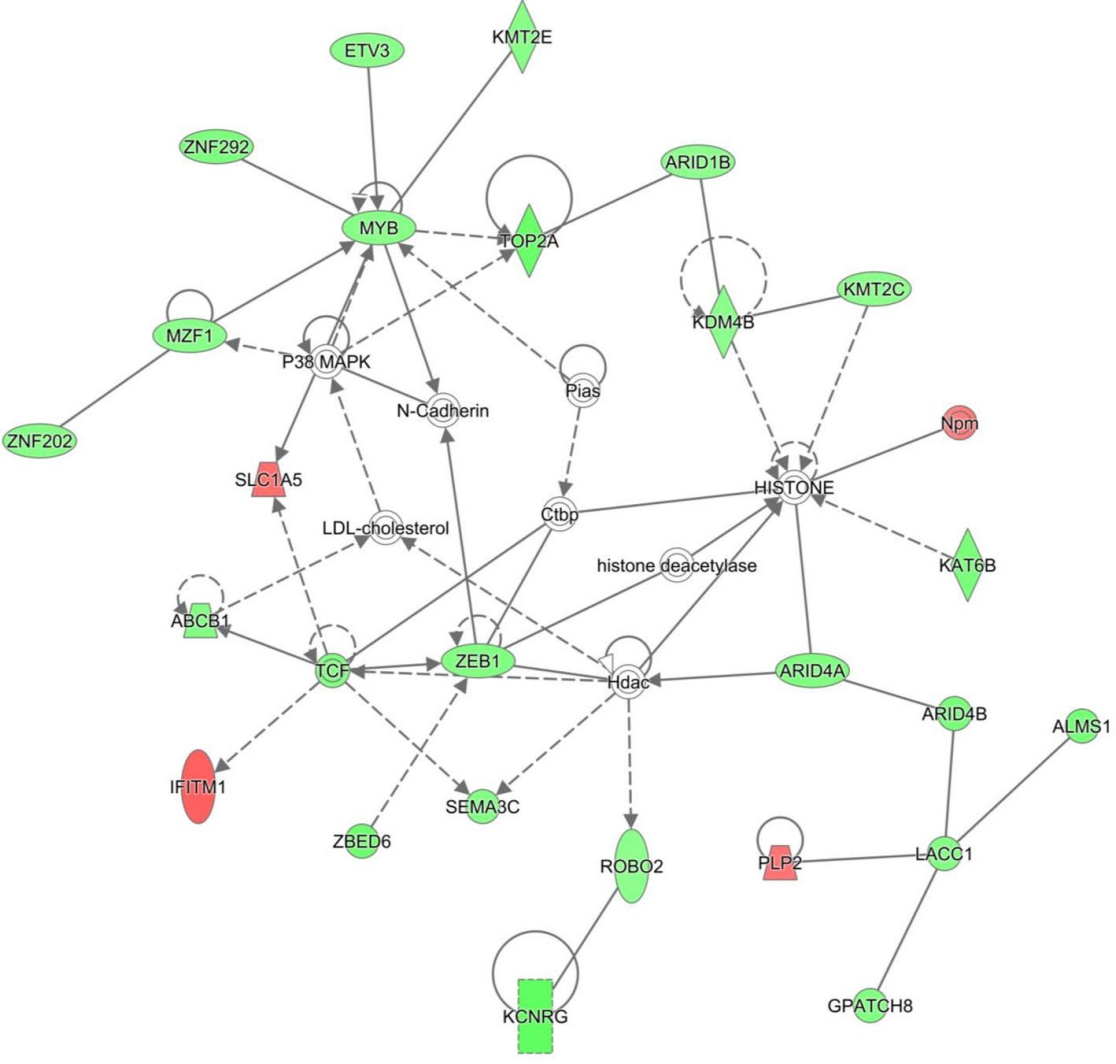
Cellular Development, Cellular Growth and Proliferation, Embryonic Development network in comparison of *n-3* PUFA, pregnant, low diet versus *n-3* PUFA, pregnant, high diet. The network is displayed graphically as nodes (genes). The node colour indicates the expression of genes with red representing up-regulation and green indicating lower expression in the *n-3* PUFA supplemented pregnant heifers on the high plane of nutrition post-insemination, compared to the *n-3* PUFA pregnant heifers on the low plane of nutrition. The network displays nodes (genes/gene products) and edges (the biological relationship between nodes). The colour intensity of the nodes indicates the fold-change increase (red) or decrease (green) associated with a particular gene in control animals compared to *n-3* PUFA supplemented. A solid line indicates a direct interaction between nodes (genes/gene products), and a dashed line indicates an indirect relationship between nodes. The shape of the node is indicative of its function. The network image was generated through the use of IPA (QIAGEN Inc., https://www.qiagenbio-informatics.com/products/ingenuity-pathway-analysis)^22^.

Heifers from within the restricted and unrestricted dietary groups were then compared in order to establish the effect of post-insemination plane of nutrition on uterine gene expression. Comparison of control animals on the varying planes of post-insemination nutrition resulted in only 13 DEG in non-pregnant heifers (Supplementary Table S4) and only 2 DEG in pregnant heifers (Supplementary Table S5); these data were not subjected to pathways analysis. Comparison of *n-3* PUFA supplemented pregnant animals based on post-insemination plane of nutrition, however, resulted in 1123 DEG which were successfully mapped in IPA (Supplementary Table S6). Biologically interesting networks derived from this comparison and the genes involved are presented in Supplementary Table S12. The data from the *n-3* PUFA supplemented pregnant animals across the two post-insemination planes of nutrition were then subjected to pathway analysis in IPA. Supplementary Table S12 contains details of the biologically interesting networks that were identified as over-represented in the comparison. Over-representation of Networks 11, cellular development, growth and proliferation; embryonic development, is depicted in Fig. 4.

#### Interaction of *n-3* PUFA supplementation and post-insemination diet on pregnant animals

**Figure 5:**
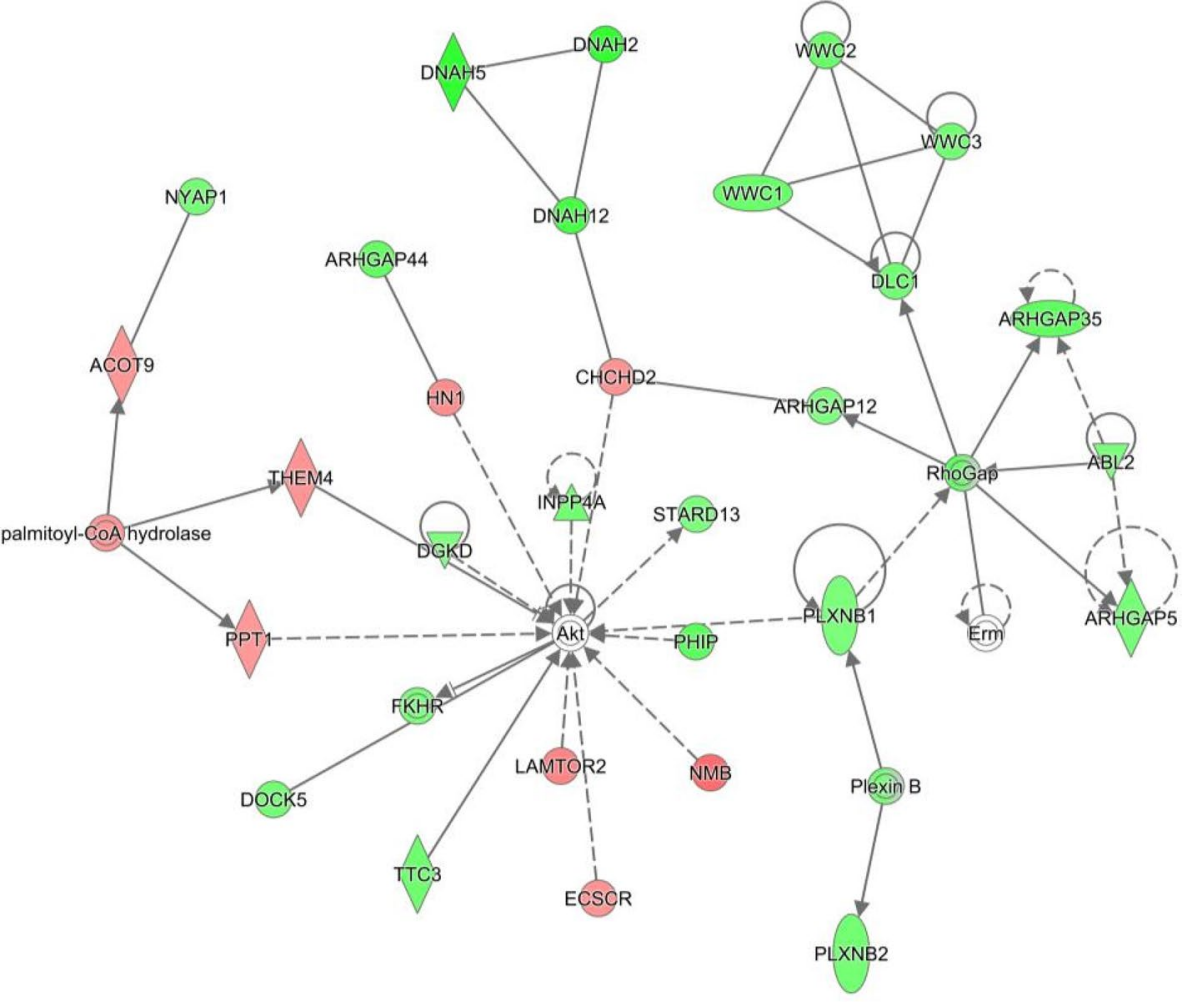
Cell Signalling, Cellular Movement, Embryonic Development network highlighted as enriched during the interaction analysis of diet and *n-3* PUFA in pregnant heifers. The network is displayed graphically as nodes (genes). The node colour indicates the expression of genes with red representing up-regulation and green indicating lower expression in the *n-3* PUFA supplemented pregnant animals compared to the control group of pregnant animals The network displays nodes (genes/gene products) and edges (the biological relationship between nodes). The colour intensity of the nodes indicates the fold-change increase (red) or decrease (green) associated with a particular gene in control animals compared to *n-3* PUFA supplemented. A solid line indicates a direct interaction between nodes (genes/gene products), and a dashed line indicates an indirect relationship between nodes. The shape of the node is indicative of its function. The network image was generated through the use of IPA (QIAGEN Inc., https://www.qiagenbio-informatics.com/products/ingenuity-pathway-analysis)^22^.

**Figure 6:**
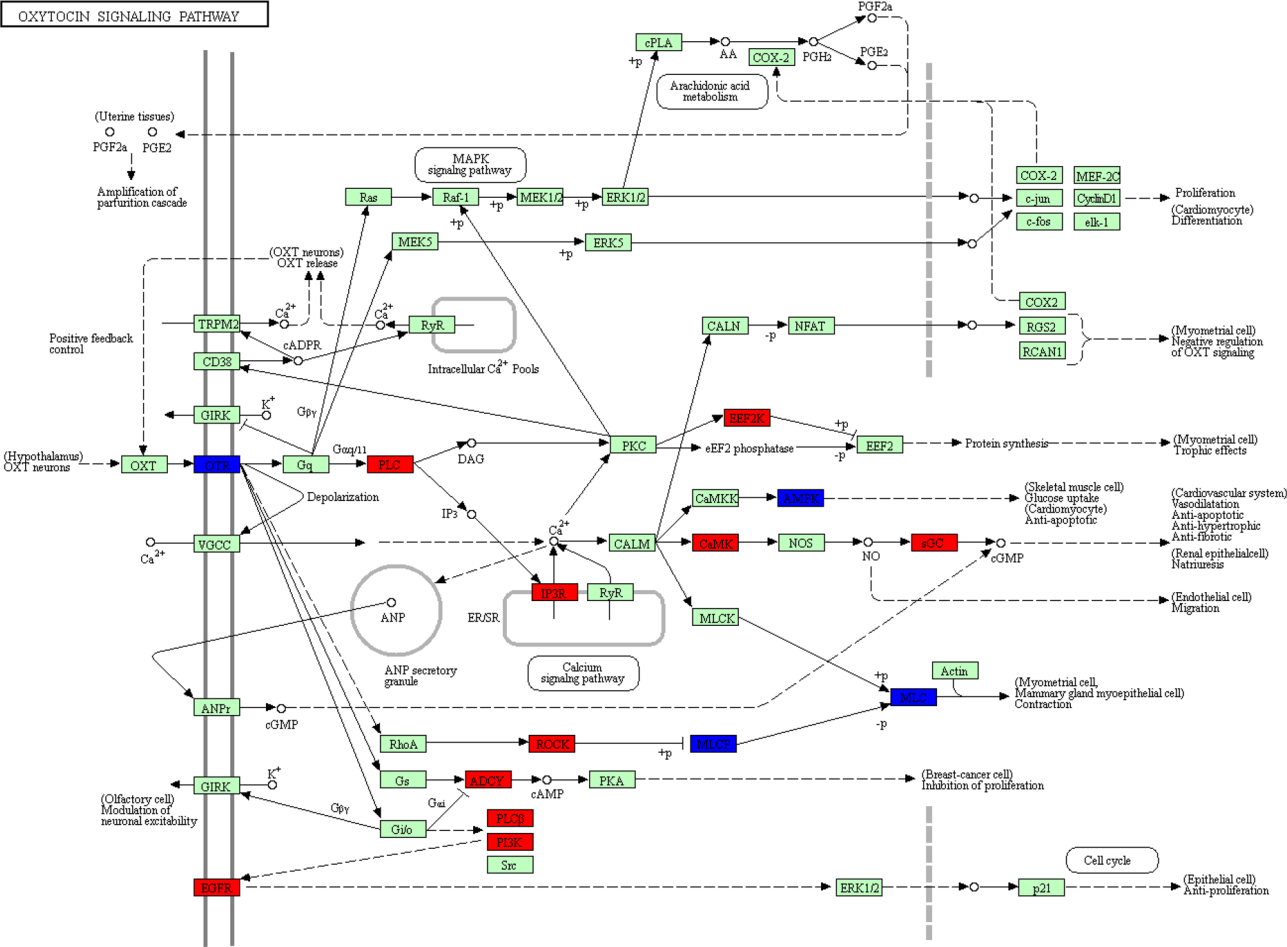
Oxytocin signalling network enriched in the analysis of the interaction of diet and *n-3* PUFA supplementation in pregnant heifers. Network is displayed graphically using the KEGG resource, with red representing genes up-regulated and blue representing genes down-regulated during the analysis of interaction of diet and *n-3* PUFA supplementation in pregnant heifers. Pathway image was generated using KEGG (https://www.genome.jp/kegg/tool/map_pathway2.html)^64^.

**Figure 7:**
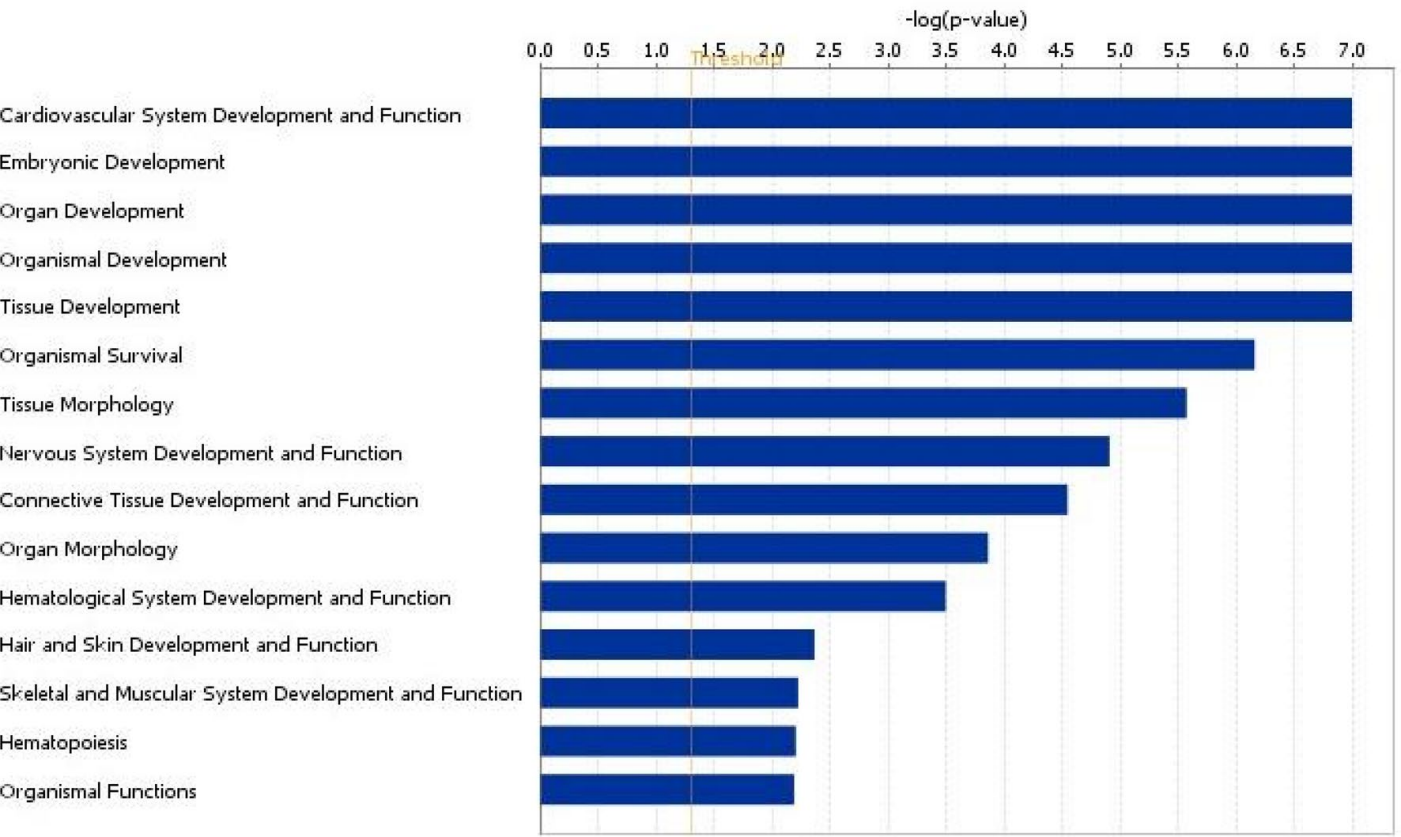
Enriched pathways in diseases and biological functions during analysis of the interaction of diet and *n-3* PUFA supplementation in pregnant heifers. The bars indicate the likelihood [-log (*P value*)] that the specific biological function was affected by the interaction of *n-3* PUFA supplementation and post-insemination plane of nutrition in pregnant heifers. The functional image was generated through the use of IPA (QIAGEN Inc., https://www.qiagenbio-informatics.com/products/ingenuity-pathway-analysis)^22^.

A total of 25 networks were identified from the 1919 DEG that were successfully mapped in IPA. Over-represented networks included cellular development, cellular growth and proliferation (Network 13) and cell signalling, cellular movement, embryonic development (Network 24, Supplementary Table S12). Enrichment of Network 24 is highlighted in Fig. 5. Additionally KEGG pathway analysis highlighted enrichment of an oxytocin signalling pathway (*P* < 0.01) (Fig. 6). DEG from the interaction were also further analysed and separated according to their biological functions, with the second most significantly associated biological function in the interaction comparison highlighted as embryonic development (Fig. 7).

#### Effect of pregnancy status on uterine gene expression

**Figure 8:**
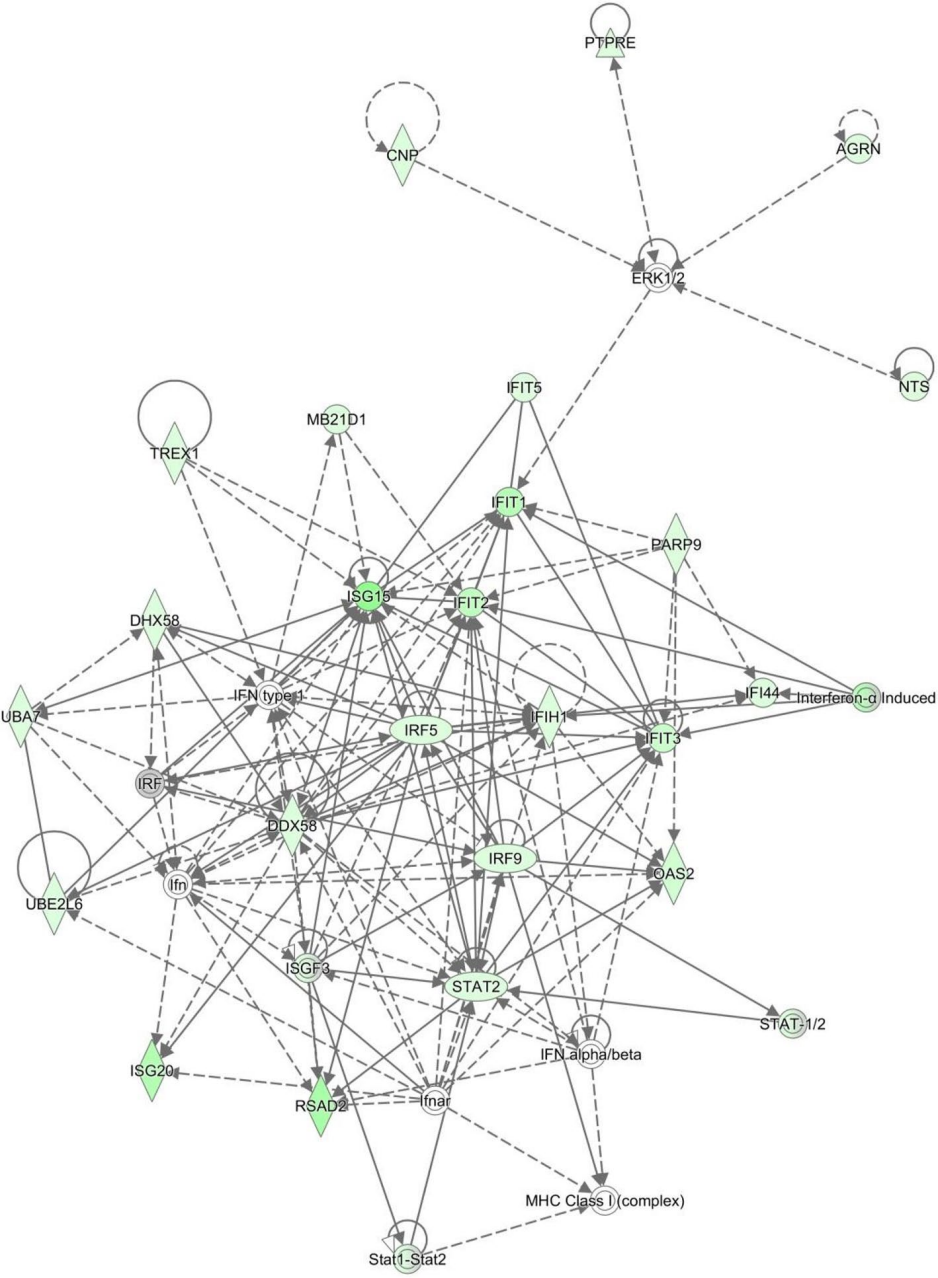
Antimicrobial response and inflammation network highlighted as enriched during the comparison of control fed heifers on the restricted plane of post-insemination nutrition, when compared by pregnancy status. The network is displayed graphically as nodes (genes). The node colour indicates the expression of genes with red representing up-regulation and green indicating lower expression in the *n-3* PUFA supplemented pregnant animals compared to the control group of pregnant animals The network displays nodes (genes/gene products) and edges (the biological relationship between nodes). The colour intensity of the nodes indicates the fold-change increase (red) or decrease (green) associated with a particular gene in control animals compared to *n-3* PUFA supplemented. A solid line indicates a direct interaction between nodes (genes/gene products), and a dashed line indicates an indirect relationship between nodes. The shape of the node is indicative of its function. The network image was generated through the use of IPA (QIAGEN Inc., https://www.qiagenbio-informatics.com/products/ingenuity-pathway-analysis)^22^.

Four comparisons were carried out within the four dietary treatment groups, comparing uterine endometrial gene expression profiles of pregnant versus non-pregnant heifers. Fold changes of all DEG that were successfully annotated using IPA are presented in Supplementary Tables S9, S10 and S11. Three of the comparisons gave rise to a significant number of DEG and were subjected to pathway analysis. Control heifers on the lower plane of post-insemination nutrition, when compared by pregnancy status, resulted in 81 DEG, with a total of 7 networks identified as enriched. Networks of interest were identified as being involved in inflammatory response (Supplementary Table S12 Network 1, Fig. 8). Canonical biological pathways enriched by the comparison included interferon signalling, indicative of signalling by the conceptus.

A comparison of pregnant versus non pregnant heifers supplemented with *n-3* PUFA and offered the low plane of post-insemination nutrition resulted in the successful mapping of 828 DEG to a biological or molecular pathway using IPA. Twenty-five networks were identified as enriched within this comparison, networks of particular interest identified as enriched in the comparison of *n-3* PUFA fed non-pregnant and pregnant heifers on the low plane of post-insemination nutrition are presented in Supplementary Table S12. The top canonical pathway identified in the comparison of pregnant versus non-pregnant heifers was interferon signalling.

The final comparison carried out to determine the effect of pregnancy status on uterine gene expression was between *n-3* PUFA-supplemented heifers on the continuous higher plane of nutrition post-insemination. A total of 493 DEG were identified and annotated as part of the IPA analysis, resulting in the identification of enriched networks of biological interest including embryonic development (Network 2) and embryonic development, and organismal development (Network 10), details of which are presented in Supplementary Table S12.

## Discussion

A large proportion of reproductive wastage in cattle arises from early pregnancy loss, typically occurring within the first 2–3 weeks of gestation, coinciding with maternal recognition of pregnancy^23^. Dietary inclusion of *n-3* PUFA generally increases both systemic and tissue concentrations of *n-3* PUFA, while reducing the concentration of *n-6* PUFA^10,16^. Previous studies carried out by our group, using microarray and cluster analyses, highlighted alteration of a number of important transcription factors following dietary supplementation with *n-3* PUFA, suggestive of a role for these fatty acids in the regulation of critical genes within uterine endometrium^1,17^. The effect of post-insemination plane of nutrition on uterine function has not been well documented in cattle and certainly not within the context of dietary *n-3* PUFA supplementation. In seasonal grass-based production systems such as those found in temperate climates, grass growth patterns do not always follow a uniform trajectory and short-term fluctuations in nutrient intake due to adverse weather are common. Indeed, when such a situation is coincident with the period of early embryogenesis in cattle, embryo mortality rates of up to 50% have been observed^24^. The aim of the research presented here was to comprehensively elucidate the effect of *n-3* PUFA supplementation and post-insemination plane of nutrition on global gene expression patterns of uterine endometrial tissue, and to determine whether effects were consistent across pregnant and non-pregnant uteri. The data generated here provides novel insight into the impact of nutrition in uterine function during early pregnancy which can be further explored using global protein characterisation and functional assays.

The effect of supplementation with *n-3* PUFA was analysed by comparing control heifers with those that received *n-3* PUFA supplementation, grouping the heifers based on their respective level of post-insemination plane of nutrition and following this, by characterising the relative impact of pregnancy status. A number of enriched networks were identified in the pregnant animals, including cellular development, growth and proliferation and embryonic development. Interestingly, however, the majority of the genes involved in this network were present at lower levels of abundance in the *n-3* PUFA-supplemented heifers. An additional embryonic development network was identified as being enriched in the comparison of control and *n-3* PUFA-fed heifers. This network highlighted 12 associated molecules, 9 of which were present at higher levels in the control-fed, and 4 of which were higher in the *n-3* PUFA-supplemented heifers. Network 16 was composed of genes involved in reproductive system development and function, with the majority of these genes present at higher levels in *n-3* PUFA-supplemented heifers. These results indicate that dietary supplementation with *n-3* PUFA has a definite effect on gene expression within the uterus, with a positive effect on genes involved in reproductive system development and function. The classification by canonical pathways highlighted mTOR signalling as associated with the DEG between pregnant control and *n-3* PUFA-supplemented heifers. The mTOR complex is key to the nutrient sensing signalling networks that control cell metabolism and governs a wide range of cellular processes including cell growth and proliferation^25,26^. It is also known to play a role in amino acid signalling which is of critical importance during the implantation phase of early pregnancy^27^. Administration of an mTOR inhibitor, rapamycin, to mice on the fourth day of pregnancy caused a significant decrease in the number of implantation sites within the uterus, and a decrease in the success of implantation of the embryos at these sites^28^. In an additional study, mTOR knock-out mouse embryos were found to be severely reduced in size suggesting further involvement of mTOR in conceptus growth, a role that has also been shown to be consistent with the mTOR signalling pathway in sheep^29,30^.

A number of interesting individual DEG were also identified during our analysis of the effect of *n-3* PUFA supplementation on pregnant heifers assigned a low plane of nutrition after insemination. Expression of Cathepsin V was 1.5 fold lower in heifers on the control diet compared to *n-3* PUFA supplemented heifers. Administration of leupeptin, an inhibitor of Cathepsin V*,* to rats on Day 4–5 embryos, completely blocked implantation of these embryos^31^. Cathepsin has also been demonstrated as having a potential role in the remodelling of endometrial cells in the porcine uterus and placenta^32^. Additionally PUFA supplementation increased the abundance of *UPK3BL* by almost threefold. This gene has been shown to be associated with epithelial cell differentiation, and its expression increased in the porcine uterus prior to implantation on Day 12, suggesting a role in implantation^33^. Guanylate binding protein 2 (*GBP2*) was also expressed at over fivefold higher levels in control heifers compared to those supplemented with *n-3* PUFA. This protein is known to inhibit the proliferation of endothelial cells, and its presence here at higher levels in control heifers could indicate a positive effect of *n-3* PUFA supplementation on cell proliferation within the uterus^34–36^.

The effect of *n-3* PUFA supplementation, combined with a high plane of nutrition after insemination, on gene expression in both pregnant and non-pregnant heifers was also analysed, leading to opposing gene expression patterns consistent with those observed between pregnant and non-pregnant heifers on restricted diets. The comparison for animals fed a higher dietary allowance after insemination led to 335 DEG being detected in the non-pregnant animals, with only one gene differing in this comparison for pregnant heifers. This indicates that *n-3* PUFA supplementation does not affect gene expression when animals are offered a higher plane of nutrition post-insemination. Only one gene, *GJB2,* also known as *Cx26,* which encodes a gap junction protein involved in cell–cell signalling, was 4.9 fold higher in control heifers^37^. This gene has been demonstrated to be expressed largely at gestational term, peaking as parturition approaches^38^. It's reduced expression here in the *n-3* PUFA-supplemented heifers may highlight a positive effect of *n-3* PUFA supplementation in aiding implantation and inhibition of luteolysis. Pathway analysis of control compared with *n-3* PUFA supplemented heifers highlighted a large number of differentially enriched networks. The most over-represented network was cellular function and metabolism, with the majority of genes present at lower levels of transcriptional abundance in control heifers. Lipid metabolism and molecular transport molecules were also present at lower levels of abundance in the non PUFA-supplemented animals, as were genes involved in the post-translational modification and cell–cell signalling network, which could possibly be due to processing by the animal of ingested *n-3* PUFA^39^. The non-specific effect of *n-3* PUFA supplementation on uterine gene expression appears to be largely involved with cellular function and general cellular metabolism, with many transcripts present at lower abundance in the control heifers (257 of the total 335 DEG). This highlights the potential effect of *n-3* PUFA supplementation to benefit the overall health and vitality of animals, directly affecting fertility^40–42^. Investigation of the transcription factors which were affected in the comparison of non-pregnant control and *n-3* PUFA supplemented heifers highlighted *PPARG* as being altered. Previous work by our group demonstrated similar altered expression of this transcription factor, when animals were supplemented with *n-3* PUFA^17^. Ligands of this transcription factor such as fatty acids, prostaglandin and eicosanoids are known to affect fertility. In this way, supplementation by *n-3* PUFA may regulate gene expression within the uterus.

Both antral follicle count^24^ and early embryo survival^24^ have been shown to be reduced in heifers exposed to a low plane of nutrition during the peri-breeding period. In order to analyse the effect of post-insemination plane of nutrition on endometrial gene expression, data from non-pregnant control heifers were first analysed, with animals on a high plane of post-insemination nutrition compared to those on a restricted diet. The comparison resulted in only 13 DEG, all of which were present at lower levels of abundance in heifers on the low dietary allowance. In the herein study, the abundance of a number of interferon-stimulated genes (ISG) present within this set of genes was reduced in the low plane of nutrition dietary group, including *MX1, MX2, IF1T1, IF1T3* and *RSAD.* These genes represent classic ISGs which are expressed within both endometrial and circulating immune cells from day 7 to day 21 of pregnancy^43–45^ and which can be further explored using metabolomic characterization and functional assays. Indeed the expression of ISGs in early pregnancy has been studied for use as an indicator of early pregnancy^43,44^. Four ISG identified as differentially expressed within the current study were previously detected by Spencer et al.^46^, during an investigation of differentially expressed genes in the uterus of day 13 pregnant cows. The ISGs, *IF1T1* and *IF1T3,* are thought to be involved in the conceptus elongation process^47^ and ISGs have been reported to be expressed in response to conceptus derived *IFN-τ* and prostaglandin in ruminants^48^. In the pregnant control animals only two genes were differentially expressed between the two planes of post-insemination nutrition, both of which were reduced in the animals on the lower plane of nutrition. Interestingly, however, one of these genes was identified as the oxytocin receptor *OXTR,* exhibiting tenfold lower expression in heifers on the low plane of nutrition. The detection of conceptus-derived anti-luteolytic *IFN-τ* is thought to result in suppression of *OXTR*^47^. While consistent with higher systemic progesterone concentrations in these heifers, the lower level of expression in the dietary-restricted heifers in the current study is unclear, as there was no observed effect of diet on overall embryo survival and indeed embryo length at recovery was actually 2.7 fold lower for diet restricted heifers^20^.

There were 1123 DEG identified in endometrial tissue between *n-3* PUFA-supplemented heifers offered a low compared with a high plane of nutrition after insemination, compared to the 13 identified within the control group. Pathway analysis revealed a number of enriched networks in the non-pregnant *n-3* PUFA-supplemented heifers, including cell morphology, cell-to-cell signalling, and an embryonic development-associated network. The majority of the molecules within these networks were present at lower levels in heifers on the lower plane of nutrition, which could have negative connotations for the reproductive potential of these animals. A number of genes normally expressed during early pregnancy or during the implantation phases were present here at lower levels in heifers on the low diet. For example, *CA1* and *SLC13A5* have previously been shown to be up regulated around the time of implantation^24,49^. The reduced abundance of these genes seen here under dietary restriction could have an effect on the success of implantation of the conceptus^50^.

The *n-3* PUFA-supplemented pregnant animals were then compared to analyse the effect of *n-3* PUFA in combination with nutritional restriction. This comparison gave rise to 844 DEG, compared to the 2 DEG identified in the same comparison within control animals. A number of interesting networks were identified between the low and high plane of nutrition groups, including networks with functions involved in cell morphology and embryonic development. The majority of the genes associated with these networks were present at lower levels in animals that were switched from a high to a low dietary allowance after insemination, providing evidence for a possible negative effect of a restricted plane of nutrition even when animals are supplemented with *n-3* PUFA. *CA1* and *TNR* were both found at much lower levels of abundance in the *n-3* PUFA-supplemented heifers that were fed a low post-insemination diet. *CA1* was present at levels of over 18-fold lower in *n-3* PUFA-supplemented heifers subjected to a low plane of nutrition and was also present at decreased levels of abundance in the *n-3* PUFA supplemented non-pregnant heifers. *TNR,* an additional gene involved in implantation, was also present at reduced levels (over three fold lower) in the low plane of nutrition group^51^. *TNR* encodes for Tenascin, a protein which is highly expressed in early pregnancy and was found to be induced at implantation sites in the mouse uterus^52^. A large number of ribosomal genes were found to be upregulated in heifers that were on the low plane of nutrition, suggesting a possible increase in protein synthesis to account for the loss of nutrients during dietary restriction^53^. An additional group of genes sharing the same direction of change to their transcriptional abundance were a number of zinc finger associated genes. This group were all present at lower levels of abundance in the *n-3* PUFA-supplemented animals that had received a low plane of post-insemination nutrition. The reason for the altered abundance is not completely clear, but zinc as a mineral has long been suggested as playing an important role in mammalian fertility^54–56^. Firstly, all nuclear receptors for steroids are zinc finger proteins, and a range of steroid metabolites are known to play an important role in conceptus driven maternal recognition of pregnancy^57,58^. Additionally, zinc is considered crucial in the formation of prostaglandin. Thus, zinc finger proteins may enhance conceptus development, and their presence here in the animals on the higher plane of nutrition suggests that maintaining the higher diet throughout pregnancy may be beneficial towards successful live birth^59^.

Pregnant heifers in the *n-3* PUFA supplemented group on both post-insemination diets were compared to control heifers on both levels of post-insemination nutrition, in order to assess whether or not there was an interaction between *n-3* PUFA supplementation and post-breeding diet. A total of 1919 genes were identified as being differentially expressed in response to diet depending on *n-3* PUFA supplementation in pregnant animals. From this list of genes, network analysis was performed, highlighting a number of enriched networks in the interaction of diet and pregnancy. A network involved in cell signalling and embryonic development was one such network, where the majority of the component genes involved were expressed at lower levels. An additional network with functions including reproductive system disease was also found to be enriched in the interaction of *n-3* PUFA supplementation and peri-insemination diet, with molecules exhibiting a lower transcriptional abundance in the *n-3* PUFA-supplemented group suggesting an overall improved reproductive health. Separating the DEG involved in the interaction into their biological function categories resulted in embryonic development highlighted as the second most enriched biological function in the interaction of diet and *n-3* PUFA in pregnant heifers. This analysis highlighted a lower expression of a number of genes involved in important embryonic developmental processes in the control heifers, such as increase in size during growth and cardiogenesis. There was also a small increase to a subset of genes involved in embryonic mortality, although the increase was only marginal in the control group. The interaction of *n-3* PUFA and plane of nutrition resulted in a marked alteration in abundance of transcripts for a number of interesting genes, including a greater than six fold increase in the level of *OXTR* expression. This gene, as previously mentioned, is usually down regulated during pregnancy to inhibit oxytocin receptor activity and potential luteolysis and pregnancy loss^17,50^. A number of zinc finger binding proteins were also found to be decreased relative to the overall interaction of *n-3* PUFA and plane of nutrition in the pregnant heifers, described in the previous comparison of pregnant *n-3* PUFA supplemented heifers on restricted and unrestricted plane of nutrition post insemination, as having a potential role in successful conceptus development^59^. *GBP2* was decreased in abundance relative to the interaction of diet and *n-3* PUFA, by a level of almost 7.5 fold. This ISG is a GTPase and has been shown as a marker of uterine receptivity for implantation^60^. *CTAGE5* was also decreased in abundance by a level of over three fold. This is a proliferation marker and is normally expressed at high levels in early stage pregnancy. The lower expression here in the *n-3* PUFA-supplemented group may highlight possible beneficial outcomes of supplementation by *n-3* PUFA.

Pregnancy status is known to affect uterine endometrial gene expression, with alterations during the critical peri-implantation period postulated to affect receptivity and advancement of conceptus development^2^. Previous work by other groups has indicated that uterine gene expression at this time is altered between pregnant and cyclic heifers, with many of the genes detectable at higher levels of abundance being members of the interferon-stimulated group of genes^17^. Understanding the pregnancy-related transcriptomic changes within uterine tissue during the critical implantation period is crucial to understanding mechanisms of failure and to investigate any positive or negative associated effects of diet.

Within the control heifers, when analysed by pregnancy status for the two different post-insemination dietary groups, 81 DEG were evident in those on the low plane of nutrition, but there was no statistically significant variation in gene expression levels in heifers on the higher plane of nutrition. Network analysis of the DEG in the heifers on the lower plane of nutrition highlighted that an antimicrobial response network was highly enriched, likely due to the number of ISGs altered in abundance due to *IFN-τ* secreted by the developing conceptus^61,62^, consistent with other studies^19^. The expression of ISGs possibly plays a role in conceptus implantation and elongation, and the extra abundance in the transcript levels of these genes in the pregnant heifers is expected^19^. A number of genes present at lower levels of abundance in the non-pregnant heifers have also been shown to exhibit similar patterns of expression in other studies. *AGRN* was present at levels of almost three fold lower in the uterine tissue of non-pregnant heifers compared to those that were pregnant. *AGRN* encodes a heparin sulphate proteoglycan, Agrin, and is involved in cell adhesion through formation of synapse of the neuromuscular junction and immunological synapses^19^. It is thought to play a role in the general formation of very close cell contacts and the subsequent signalling between them. A number of genes involved in cell binding with a possible function during the implantation process were also present at reduced levels of abundance in the non-pregnant heifers compared to the expression levels in the pregnant heifers. *LGALS9,* which encoded the protein Galectin 9 has previously been shown to mediate cell aggregation and adhesion, and due to its appearance as an ISG, has also been linked with implantation^19^. Galectin 3, encoded by *LGALS3B9,* was also present at reduced levels of abundance in the non-pregnant uterine tissue, at levels of over 2.5 fold. Galectin 15 has previously been demonstrated to be involved in ovine endometrial epithelium to regulate trophoblast migration, adhesion and differentiation, and it is possible that the Galectins present at higher levels of abundance in the pregnant heifers here also play a similar role during early pregnancy^19^.

*n-3* PUFA-supplemented heifers on both the lower and higher plane of post-insemination diets were also compared within the treatment groups in order to establish the effect of pregnancy status on uterine gene expression. Comparison of pregnant and non-pregnant *n-3* PUFA supplemented heifers on the low plane of nutrition resulted in 828 DEG, involved in 25 molecular or biological networks of interest, highlighted by pathway analysis. One of the networks highlighted, embryonic and organismal tissue development, had 28 focus molecules involved from the list of DEG, all of which, not surprisingly, were present at lower levels of abundance in non-pregnant animals. A similar result was observed in the antimicrobial response, inflammatory response, cell to cell signalling and interaction network, which contains a number of ISGs. The majority of molecules in this network were present at higher levels of transcriptional abundance in the pregnant heifers, presumably stimulated by *IFN-τ* from the conceptus. During early pregnancy, the conceptus not only needs to block luteal regression, but also, due to its shared paternally-derived genetic composition, needs to manipulate the dams immune response in order to establish successful pregnancy and prevent destruction^19^. One of these ISGs, *ISG20,* was determined to be present at 44 fold lower abundance in the non-pregnant heifers, with an additional gene, *ISG15* decreased by over 28 fold.

Comparison of pregnancy status between *n-3* PUFA-fed heifers maintained on the higher plane of post-insemination nutrition resulted in a total of 493 DEG, and highlighted 25 enriched networks during pathway analysis. One enriched network, involved in developmental disorder, hereditary disorder and metabolic disease, included genes such as *EBAGT* and *ESRRA* which are involved in estrogen binding. The higher expression of these genes in non-pregnant heifers is to be expected as during early pregnancy, estrogen receptor expression is inhibited^19^. Two additional genes present at higher levels in the endometrium of non-pregnant heifers, *SLC13A5* and *ANPEP,* were also reported by Forde et al*.*^63^ when comparing uterine gene expression patterns of cyclic and pregnant heifers. In that study the expression of these genes was not altered after Day 7 of pregnancy and their expression was independent of pregnancy status.

**Figure 9:**
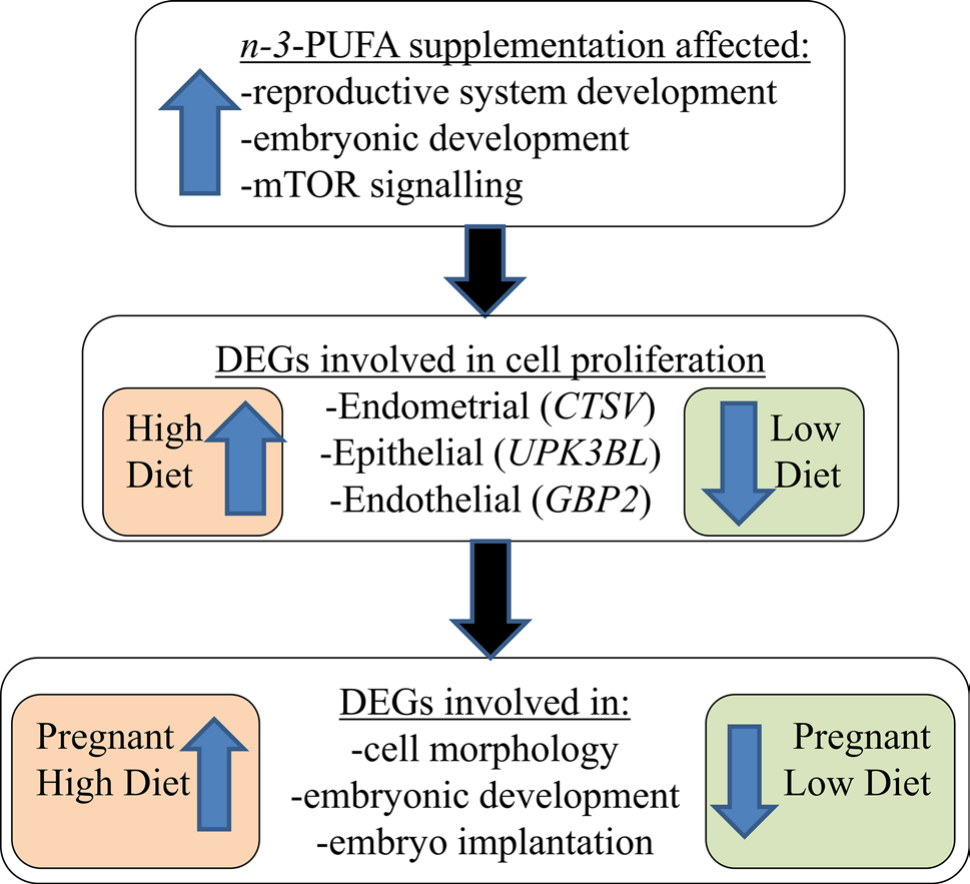
Summary of the main observed effects of n-3 PUFA supplementation and post-insemination diet on endometrial gene expression.

While a precursor to this study^20^ suggested limited effects of the dietary regimens employed here on early embryo survival and/or development, nonetheless, in the current study we observed interesting changes in the expression of many key reproductive genes across the various treatment comparisons, suggesting potential novel interactions between dietary *n-3* PUFA consumption and post breeding metabolic status in pregnant heifers. The effect of *n-3* PUFA supplementation in pregnant heifers offered a higher plane of post-insemination nutrition is in contrast to that observed for their non-pregnant contemporaries, where differential expression of genes involved in estrogen binding as well as in key biological processes including cellular function and maintenance and embryonic development were evident. This highlights the importance of a comprehensive examination of potential interacting metabolic factors that may affect the interpretation of biochemical processes influencing reproductive function. Overall results indicate that *n-3* PUFA supplementation does not appreciably affect gene expression of uterine endometrial tissue when animals are maintained on an adequate feed allowance. However, when animals undergo significant fluctuation in nutrient supply there is evidence that many biochemical processes are altered, some of which may have implications for embryo survival (Fig. 9). This study provides a novel insight into the effects of both n-3-PUFA supplementation and post-insemination plane of nutrition on the endometrial tissue. However, further validation of transcriptional results presented in this study are warranted, for example through the utilisation of functional assays or global proteomics to fully characterise the molecular response of endometrial tissue to n-3 PUFA supplementation and post-insemination plane of nutrition.

## Supplementary information

Supplementary Tables.

## Data Availability

The dataset analysed during the current study is available in the NCBI Gene Expression Omnibus https://www.ncbi.nlm.nih.gov/geo/ under accession number GSE141937.
