# Supplementary material for: Effects of dietary n-3-PUFA supplementation, post-insemination plane of nutrition and pregnancy status on the endometrial transcriptome of beef heifers
Source: Sci Rep. 2020 Nov 27;10:20798. doi: 10.1038/s41598-020-77604-y (PMC7695717; doi:10.1038/s41598-020-77604-y)
Supplement: Supplementary file 1 — Supplementary Tables. [file 41598_2020_77604_MOESM1_ESM.pdf]

Supplementary information

Effects of dietary *n-3*-PUFA supplementation, post-insemination plane of nutrition and pregnancy status on the endometrial transcriptome of beef heifers

Authors: Carla Surlis[1], Paul Cormican[1], Sinead M. Waters[1], Patrick Lonergan[2], Kate Keogh[1], David N. Doyle[1] and David A. Kenny[1,2*].

[1]Animal and Grassland Research and Innovation Centre, Teagasc, Grange, Dunsany, Co. Meath. Ireland.

[2]School of Agriculture and Food Science, University College Dublin, Belfield, Dublin 4, Ireland.

*Corresponding Author: Prof. David Kenny;

email: david.kenny@teagasc.ie

Table S1. Differentially expressed genes in Control, pregnant, low diet versus PUFA, pregnant, low diet

Symbol	Entrez Gene Name	p-value	Fold Change[1]
ABCA9	ATP binding cassette subfamily A member 9	0.0086	1.567
ABCB1	ATP binding cassette subfamily B member 1	0.00006	1.84
ABCC10	ATP binding cassette subfamily C member 10	0.00265	1.577
ABHD11	Abhydrolase domain containing 11	0.000487	-1.51
ABHD2	Abhydrolase domain containing 2	0.000377	-1.642
Acan	Aggrecan	0.00216	2.231
ADAMTS10	ADAM metallopeptidase with thrombospondin type 1 motif 10	0.0144	1.597
ADAMTS2	ADAM metallopeptidase with thrombospondin type 1 motif 2	0.000776	1.621
ADAMTSL1	ADAMTS like 1	0.00761	1.589
ADGRF5	Adhesion G protein-coupled receptor F5	0.00043	1.522
ADGRL3	Adhesion G protein-coupled receptor L3	0.00878	1.773
AFAP1L1	Actin filament associated protein 1 like 1	0.00876	1.717
AGR2	Anterior gradient 2, protein disulphide isomerase family member	0.00218	-1.927
AHSG	Alpha 2-HS glycoprotein	0.0111	-2.526
ALMS1	ALMS1, centrosome and basal body associated protein	0.00489	1.522
ANGPT2	Angiopoietin 2	0.000261	1.652
ANKRD12	Ankyrin repeat domain 12	0.000888	1.569
ANKRD26	Ankyrin repeat domain 26	0.000032	1.983
ANKRD6	Ankyrin repeat domain 6	0.000988	1.574
ANO8	Anoctamin 8	0.00379	1.497
APC	APC, WNT signaling pathway regulator	0.000229	1.701
ARG2	Arginase 2	0.00933	-1.59
ARHGAP21	Rho gtpase activating protein 21	0.00171	1.52
ARHGAP44	Rho gtpase activating protein 44	0.00237	1.645
ARHGEF10	Rho guanine nucleotide exchange factor 10	0.00127	1.517
ARID4A	AT-rich interaction domain 4A	0.000898	1.562
ARID4B	AT-rich interaction domain 4B	0.000866	1.538
ARPC3	Actin related protein 2/3 complex subunit 3	0.00008	-1.555
ASNS	Asparagine synthetase (glutamine-hydrolyzing)	0.000442	-1.567
ATAD5	Atpase family, AAA domain containing 5	0.00221	1.528
ATP5J2	ATP synthase, H+ transporting, mitochondrial Fo complex subunit F2	0.00104	-1.509
ATP6V0A2	Atpase H+ transporting V0 subunit a2	0.0189	-1.504

B2M	Beta-2-microglobulin	0.0115	-1.561
B3GALNT1	Beta-1,3-N-acetylgalactosaminyltransferase 1 (globoside blood group)	0.000255	-2.516
BACE2	Beta-site APP-cleaving enzyme 2	0.00633	-1.59
BAZ2B	Bromodomain adjacent to zinc finger domain 2B	0.000081	1.816
BBX	BBX, HMG-box containing	0.00577	1.5
BEX5	Brain expressed X-linked 5	0.000047	-1.853
BPTF	Bromodomain PHD finger transcription factor	0.00217	1.575
BRCA2	BRCA2, DNA repair associated	0.0107	1.613
BTBD8	BTB domain containing 8	0.000434	1.585
BTF3	Basic transcription factor 3	0.0000001	-1.621
BTF3L4	Basic transcription factor 3 like 4	0.000007	-1.507
C14orf1	Chromosome 14 open reading frame 1	0.000126	-1.716
C14orf166	Chromosome 14 open reading frame 166	0.000224	-1.595
C5orf30	Chromosome 5 open reading frame 30	0.00106	-1.778
CASP8	Caspase 8	0.00393	-1.75
CATSPERD	Cation channel sperm associated auxiliary subunit delta	0.0087	1.794
CATSPERG	Cation channel sperm associated auxiliary subunit gamma	0.00238	1.783
CCDC150	Coiled-coil domain containing 150	0.00001	1.982
CCDC158	Coiled-coil domain containing 158	0.000085	1.707
CCDC186	Coiled-coil domain containing 186	0.00356	1.537
CCDC39	Coiled-coil domain containing 39	0.0125	1.874
CCDC57	Coiled-coil domain containing 57	0.00885	1.523
CD48	CD48 molecule	0.000065	-1.974
CD59	CD59 molecule	0.00106	-1.681
CD9	CD9 molecule	0.000062	-1.496
CDH17	Cadherin 17	0.00019	-2.041
CDH3	Cadherin 3	0.000314	1.618
CDK3	Cyclin dependent kinase 3	0.0157	1.629
CDKN1A	Cyclin dependent kinase inhibitor 1A	0.00127	-1.585
CENPJ	Centromere protein J	0.00202	1.544
CEP135	Centrosomal protein 135	0.00498	1.526
CEP162	Centrosomal protein 162	0.0015	1.645
CEP170	Centrosomal protein 170	0.000105	1.629
CEP350	Centrosomal protein 350	0.00497	1.628
CFB	Complement factor B	0.000947	-2.59

CHAD	Chondroadherin	0.00652	1.56
CHD7	Chromodomain helicase DNA binding protein 7	0.00841	1.539
CHD9	Chromodomain helicase DNA binding protein 9	0.00424	1.58
CHL1	Cell adhesion molecule L1 like	0.014	1.729
CHST11	Carbohydrate (chondroitin 4) sulfotransferase 11	0.00664	1.505
CHST7	Carbohydrate sulfotransferase 7	0.0103	1.581
CIRBP	Cold inducible RNA binding protein	0.000154	-1.569
CLCN2	Chloride voltage-gated channel 2	0.00357	1.538
CLIC1	Chloride intracellular channel 1	0.000839	-1.528
CLIP1	CAP-Gly domain containing linker protein 1	0.000004	1.877
CNIH4	Cornichon family AMPA receptor auxiliary protein 4	0.0002	-1.505
CNTLN	Centlein	0.0011	1.591
CNTRL	Centriolin	0.00546	1.564
COCH	Cochlin	0.00418	2.466
COL27A1	Collagen type XXVII alpha 1	0.01760	1.595
COLEC12	Collectin subfamily member 12	0.000151	1.557
COX7A2	Cytochrome c oxidase subunit 7A2	0.000403	-1.547
COX7A2L	Cytochrome c oxidase subunit 7A2 like	0.000038	-1.613
CP	Ceruloplasmin (ferroxidase)	0.00169	1.59
CRYBB1	Crystallin beta B1	0.0162	-1.542
CSPP1	Centrosome and spindle pole associated protein 1	0.000556	1.682
CTSV	Cathepsin V	0.00207	-1.512
CTTNBP2	Cortactin binding protein 2	0.0012	1.528
CWH43	Cell wall biogenesis 43 C-terminal homolog	0.00426	-2.008
CYP20A1	Cytochrome P450 family 20 subfamily A member 1	0.000167	-1.523
CYP2R1	Cytochrome P450 family 2 subfamily R member 1	0.000108	-1.516
DCDC1	Doublecortin domain containing 1	0.0167	1.867
DGKI	Diacylglycerol kinase iota	0.0179	2.017
DGKQ	Diacylglycerol kinase theta	0.000352	1.599
DGUOK	Deoxyguanosine kinase	0.000016	-1.565
DLL1	Delta like canonical Notch ligand 1	0.00165	1.602
DMGDH	Dimethylglycine dehydrogenase	0.00182	1.712
DNAH12	Dynein axonemal heavy chain 12	0.0145	1.626
DOK6	Docking protein 6	0.0185	1.763
DST	Dystonin	0.00764	1.534

DUSP4	Dual specificity phosphatase 4	0.0108	-1.599
DUT	Deoxyuridine triphosphatase	0.000499	-1.573
DZIP3	DAZ interacting zinc finger protein 3	0.000073	1.544
EEA1	Early endosome antigen 1	0.00386	1.667
EEF1A1	Eukaryotic translation elongation factor 1 alpha 1	0.000268	-1.553
EEF1B2	Eukaryotic translation elongation factor 1 beta 2	0.000081	-1.718
EEF1D	Eukaryotic translation elongation factor 1 delta	0.000052	-1.548
EEF1G	Eukaryotic translation elongation factor 1 gamma	0.000031	-1.565
EGFL7	EGF like domain multiple 7	0.00136	1.669
EIF2S3	Eukaryotic translation initiation factor 2 subunit gamma	0.0004	-1.538
EIF3E	Eukaryotic translation initiation factor 3 subunit E	0.00016	-1.653
EIF3I	Eukaryotic translation initiation factor 3 subunit I	0.0003	-1.506
EIF3M	Eukaryotic translation initiation factor 3 subunit M	0.000305	-1.659
EIF4G3	Eukaryotic translation initiation factor 4 gamma 3	0.000213	1.547
ELK3	ELK3, ETS transcription factor	0.000102	1.631
EMB	Embigin	0.008430	-1.792
ERCC6L2	ERCC excision repair 6 like 2	0.000019	1.562
ERICH2	Glutamate rich 2	0.017	-2.074
ESCO1	Establishment of sister chromatid cohesion N-acetyltransferase 1	0.000139	1.733
FABP7	Fatty acid binding protein 7	0.00233	-1.715
FAM13C	Family with sequence similarity 13 member C	0.00339	1.548
FAM193B	Family with sequence similarity 193 member B	0.000777	1.624
FAM214A	Family with sequence similarity 214 member A	0.000036	1.575
FAM229B	Family with sequence similarity 229 member B	0.00582	-1.668
FAM65C	Family with sequence similarity 65 member C	0.00431	1.6
FBXO10	F-box protein 10	0.00243	1.506
FGF13	Fibroblast growth factor 13	0.00448	-1.613
FILIP1L	Filamin A interacting protein 1 like	0.0016	1.514
FNBP4	Formin binding protein 4	0.000054	2.053
FSD2	Fibronectin type III and SPRY domain containing 2	0.00261	1.596
GANC	Glucosidase alpha, neutral C	0.00283	1.569
GBP2	Guanylate binding protein 2	0.00865	5.189
GCC2	GRIP and coiled-coil domain containing 2	0.000404	1.672
GDPD2	Glycerophosphodiester phosphodiesterase domain containing 2	0.00923	-1.87
GEMIN2	Gem nuclear organelle associated protein 2	0.0001	-1.573

GGT5	Gamma-glutamyltransferase 5	0.0148	1.521
GK	Glycerol kinase	0.00448	-1.536
GLIS2	GLIS family zinc finger 2	0.00489	1.505
GNAL	G protein subunit alpha L	0.00212	1.838
GOLGA4	Golgin A4	0.00138	1.599
GOLGB1	Golgin B1	0.000634	1.572
GOT1	Glutamic-oxaloacetic transaminase 1	0.00282	-1.541
GPRC5A	G protein-coupled receptor class C group 5 member A	0.00339	-2.157
GRIK1	Glutamate ionotropic receptor kainate type subunit 1	0.00258	2.123
GRK4	G protein-coupled receptor kinase 4	0.00157	1.701
GZMK	Granzyme K	0.0111	-2.404
HACD3	3-hydroxyacyl-coa dehydratase 3	0.000241	-1.505
HACD4	3-hydroxyacyl-coa dehydratase 4	0.00684	-1.721
HAS3	Hyaluronan synthase 3	0.00775	1.653
HEXB	Hexosaminidase subunit beta	0.00546	-2.481
HIGD1A	HIG1 hypoxia inducible domain family member 1A	0.00111	-2.114
HINT1	Histidine triad nucleotide binding protein 1	0.000678	-1.56
HIP1	Huntingtin interacting protein 1	0.00544	1.512
HIST1H4J	Histone cluster 1, h4j	0.00801	-1.583
HLA-B	Major histocompatibility complex, class I, B	0.00844	-1.55
HSPE1	Heat shock protein family E (Hsp10) member 1	0.00135	-1.497
IGBP1	Immunoglobulin (CD79A) binding protein 1	0.000012	-1.513
IGSF9	Immunoglobulin superfamily member 9	0.0178	1.606
IL33	Interleukin 33	0.00112	1.836
ISM1	Isthmin 1, angiogenesis inhibitor	0.00878	1.57
ITGA2B	Integrin subunit alpha 2b	0.00573	1.884
ITGA7	Integrin subunit alpha 7	0.0015	1.604
JCHAIN	Joining chain of multimeric iga and igm	0.00479	4.709
KAT6B	Lysine acetyltransferase 6B	0.00354	1.528
KCNMA1	Potassium calcium-activated channel subfamily M alpha 1	0.00632	1.519
KIAA1211	Kiaa1211	0.000206	1.927
KIF20B	Kinesin family member 20B	0.00827	1.58
KIF27	Kinesin family member 27	0.00018	1.825
KIFC3	Kinesin family member C3	0.00404	1.536
KRBA1	KRAB-A domain containing 1	0.00227	1.885

KRTCAP3	Keratinocyte associated protein 3	0.000408	-1.509
LENG8	Leukocyte receptor cluster member 8	0.00635	1.522
LGALS3	Lectin, galactoside binding soluble 3	0.000028	-1.691
LIPT1	Lipoyltransferase 1	0.000131	-1.55
LRRC32	Leucine rich repeat containing 32	0.000068	1.594
LRRCC1	Leucine rich repeat and coiled-coil centrosomal protein 1	0.000061	1.563
LSM3	LSM3 homolog, U6 small nuclear RNA and mrna degradation associated	0.000096	-1.519
LSM8	LSM8 homolog, U6 small nuclear RNA associated	0.000026	-1.53
LTBP4	Latent transforming growth factor beta binding protein 4	0.000766	1.505
MACF1	Microtubule-actin crosslinking factor 1	0.00107	1.628
MAGI1	Membrane associated guanylate kinase, WW and PDZ domain containing 1	0.00195	1.544
MAGOH	Mago homolog, exon junction complex core component	0.000148	-1.612
MAP3K13	Mitogen-activated protein kinase kinase kinase 13	0.00141	1.555
MAP3K14	Mitogen-activated protein kinase kinase kinase 14	0.000657	1.787
MAP7D3	MAP7 domain containing 3	0.000041	1.729
MARCH3	Membrane associated ring-CH-type finger 3	0.0157	-1.563
MAT1A	Methionine adenosyltransferase 1A	0.0133	1.76
MATN2	Matrilin 2	0.00203	1.6
MCM10	Minichromosome maintenance 10 replication initiation factor	0.00377	2.017
MEGF8	Multiple EGF like domains 8	0.00303	1.537
MEOX2	Mesenchyme homeobox 2	0.000695	1.569
METTL12	Methyltransferase like 12	0.00072	-1.813
MGST3	Microsomal glutathione S-transferase 3	0.000006	-1.646
MPHOSPH9	M-phase phosphoprotein 9	0.000063	1.531
MPZL2	Myelin protein zero like 2	0.0093	-1.644
MRPL3	Mitochondrial ribosomal protein L3	0.000092	-1.579
MRPL32	Mitochondrial ribosomal protein L32	0.000225	-1.568
MRPS35	Mitochondrial ribosomal protein S35	0.000016	-1.507
MS4A8	Membrane spanning 4-domains A8	0.00313	-1.965
MT-ATP6	ATP synthase F0 subunit 6	0.000486	-2.003
MT-CO1	Cytochrome c oxidase subunit I	0.000024	-2.026
MT-CO2	Cytochrome c oxidase subunit II	0.000132	-2.13
MT-CO3	Cytochrome c oxidase III	0.000166	-2.086
MT-CYB	Cytochrome b	0.000124	-2.29
MT-ND1	NADH dehydrogenase, subunit 1 (complex I)	0.000323	-2.166

MT-ND2	Mtnd2	0.00203	-1.943
MT-ND3	NADH dehydrogenase, subunit 3 (complex I)	0.0015	-1.978
MT-ND4	NADH dehydrogenase, subunit 4 (complex I)	0.00237	-1.912
MT-ND4L	NADH dehydrogenase, subunit 4L (complex I)	0.000224	-2.218
MT-ND5	NADH dehydrogenase, subunit 5 (complex I)	0.00151	-1.938
MT-ND6	NADH dehydrogenase, subunit 6 (complex I)	0.0166	-1.712
MYCBP2	MYC binding protein 2, E3 ubiquitin protein ligase	0.00137	1.495
MYH11	Myosin heavy chain 11	0.00589	1.668
MYL3	Myosin light chain 3	0.00526	-1.891
MYOZ1	Myozenin 1	0.000003	-1.886
MZF1	Myeloid zinc finger 1	0.000235	1.669
NAPB	NSF attachment protein beta	0.000674	1.664
NAV3	Neuron navigator 3	0.00765	1.704
NDRG4	NDRG family member 4	0.0175	1.575
NDUFA1	NADH:ubiquinone oxidoreductase subunit A1	0.000117	-1.505
NDUFA12	NADH:ubiquinone oxidoreductase subunit A12	0.000012	-1.669
NDUFA4	NDUFA4, mitochondrial complex associated	0.000098	-1.516
NDUFB1	NADH:ubiquinone oxidoreductase subunit B1	0.00159	-1.533
NDUFB3	NADH:ubiquinone oxidoreductase subunit B3	0.000035	-1.558
NDUFB6	NADH:ubiquinone oxidoreductase subunit B6	0.000165	-1.516
NDUFC1	NADH:ubiquinone oxidoreductase subunit C1	0.000270	-1.559
NEMF	Nuclear export mediator factor	0.000941	1.517
NETO2	Neuropilin and tolloid like 2	0.008230	-1.815
NEURL4	Neuralized E3 ubiquitin protein ligase 4	0.000196	1.529
NFKBIZ	NFKB inhibitor zeta	0.000037	2.055
NIN	Ninein	0.002530	1.64
NIPBL	NIPBL, cohesin loading factor	0.000428	1.516
NKPD1	Ntpase, KAP family P-loop domain containing 1	0.004890	2.056
NKTR	Natural killer cell triggering receptor	0.000583	1.658
NMB	Neuromedin B	0.002490	-1.875
NPAT	Nuclear protein, coactivator of histone transcription	0.001640	1.541
NPM1	Nucleophosmin	0.000001	-1.727
NTN4	Netrin 4	0.002080	1.612
NUP93	Nucleoporin 93	0.002700	-1.692
NYAP1	Neuronal tyrosine phosphorylated phosphoinositide-3-kinase adaptor 1	0.010500	1.495

OLFML2B	Olfactomedin like 2B	0.001120	1.529
ORMDL2	ORMDL sphingolipid biosynthesis regulator 2	0.000003	-1.548
OSBPL8	Oxysterol binding protein like 8	0.004200	1.513
PAXBP1	PAX3 and PAX7 binding protein 1	0.000029	1.588
PCDH12	Protocadherin 12	0.000829	2.355
PCF11	PCF11 cleavage and polyadenylation factor subunit	0.000390	1.551
PCM1	Pericentriolar material 1	0.000444	1.509
PDE6D	Phosphodiesterase 6D	0.000091	-1.782
PFDN1	Prefoldin subunit 1	0.000024	-1.53
PFDN4	Prefoldin subunit 4	0.000154	-1.552
PFDN5	Prefoldin subunit 5	0.000030	-1.641
PGF	Placental growth factor	0.000273	1.642
PGM2	Phosphoglucomutase 2	0.001040	1.717
PHF19	PHD finger protein 19	0.002560	1.519
PHF3	PHD finger protein 3	0.000081	1.623
PHOSPHO2	Phosphatase, orphan 2	0.000483	-1.496
PIGP	Phosphatidylinositol glycan anchor biosynthesis class P	0.000031	-1.565
PLA2G7	Phospholipase A2 group VII	0.000204	-1.514
PLCB4	Phospholipase C beta 4	0.002940	1.509
PLEKHD1	Pleckstrin homology and coiled-coil domain containing D1	0.000722	2.309
PLEKHH3	Pleckstrin homology, myth4 and FERM domain containing H3	0.000036	1.594
PLIN2	Perilipin 2	0.003300	-1.521
PLK1	Polo like kinase 1	0.003570	1.592
Pln	Phospholamban	0.005930	1.587
PLP2	Proteolipid protein 2	0.000018	-1.568
PLS1	Plastin 1	0.007840	-1.569
PLXDC1	Plexin domain containing 1	0.000507	1.554
POLR2K	RNA polymerase II subunit K	0.000327	-1.509
POP5	POP5 homolog, ribonuclease P/MRP subunit	0.000039	-1.522
PPM1L	Protein phosphatase, Mg2+/Mn2+ dependent 1L	0.007420	-1.555
PQLC3	PQ loop repeat containing 3	0.015300	-1.611
PRDX1	Peroxiredoxin 1	0.000027	-1.589
PRSS23	Protease, serine 23	0.000038	-1.996
PSMB4	Proteasome subunit beta 4	0.000396	-1.52
PSMG4	Proteasome assembly chaperone 4	0.000021	-1.67

PSPH	Phosphoserine phosphatase	0.005560	-2.464
PTK2B	Protein tyrosine kinase 2 beta	0.002000	1.509
RAB27B	RAB27B, member RAS oncogene family	0.002920	-1.772
RACK1	Receptor for activated C kinase 1	0.000005	-1.728
RAP2A	RAP2A, member of RAS oncogene family	0.000459	-1.63
RAPGEF4	Rap guanine nucleotide exchange factor 4	0.000422	2.005
RASGRP1	RAS guanyl releasing protein 1	0.009380	1.592
RASGRP3	RAS guanyl releasing protein 3	0.003500	1.529
RBBP6	RB binding protein 6, ubiquitin ligase	0.001810	1.531
RBM3	RNA binding motif (RNP1, RRM) protein 3	0.000080	-1.829
REV3L	REV3 like, DNA directed polymerase zeta catalytic subunit	0.000456	1.55
RGS6	Regulator of G-protein signaling 6	0.014800	1.607
RHEB	Ras homolog enriched in brain	0.000199	-1.528
RPGRIP1L	RPGRIP1 like	0.003120	1.541
RPL10	Ribosomal protein L10	0.000161	-1.503
RPL10A	Ribosomal protein l10a	0.000119	-1.577
RPL11	Ribosomal protein L11	0.000257	-1.504
RPL13	Ribosomal protein L13	0.000054	-1.577
RPL13A	Ribosomal protein l13a	0.000104	-1.644
RPL17	Ribosomal protein L17	0.000068	-1.682
RPL21	Ribosomal protein L21	0.000053	-1.659
RPL22	Ribosomal protein L22	0.000018	-1.725
RPL23	Ribosomal protein L23	0.000059	-1.746
RPL26	Ribosomal protein L26	0.000014	-1.756
RPL27	Ribosomal protein L27	0.000007	-1.852
RPL27A	Ribosomal protein l27a	0.000311	-1.617
RPL3	Ribosomal protein L3	0.000225	-1.557
RPL30	Ribosomal protein L30	0.000013	-1.798
RPL31	Ribosomal protein L31	0.000181	-1.667
RPL32	Ribosomal protein L32	0.000007	-1.76
RPL35A	Ribosomal protein l35a	0.000015	-1.877
RPL36	Ribosomal protein L36	0.000031	-1.525
RPL36A	Ribosomal protein l36a	0.000000	-2.23
RPL37	Ribosomal protein L37	0.000006	-1.974
RPL37A	Ribosomal protein l37a	0.000021	-1.752

RPL5	Ribosomal protein L5	0.000045	-1.567
RPL8	Ribosomal protein L8	0.000054	-1.575
RPLP0	Ribosomal protein lateral stalk subunit P0	0.000002	-1.762
RPLP1	Ribosomal protein lateral stalk subunit P1	0.000000	-1.878
RPLP2	Ribosomal protein lateral stalk subunit P2	0.000002	-1.908
RPS10	Ribosomal protein S10	0.000094	-1.634
RPS11	Ribosomal protein S11	0.000091	-1.605
RPS12	Ribosomal protein S12	0.000014	-1.793
RPS13	Ribosomal protein S13	0.000003	-1.65
RPS14	Ribosomal protein S14	0.000104	-1.647
RPS17	Ribosomal protein S17	0.000019	-1.893
RPS18	Ribosomal protein S18	0.000019	-1.676
RPS19	Ribosomal protein S19	0.000015	-1.706
RPS2	Ribosomal protein S2	0.000432	-1.512
RPS20	Ribosomal protein S20	0.000007	-1.839
RPS21	Ribosomal protein S21	0.000006	-1.998
RPS24	Ribosomal protein S24	0.000004	-1.861
RPS25	Ribosomal protein S25	0.000001	-1.935
RPS26	Ribosomal protein S26	0.003370	-1.533
RPS27	Ribosomal protein S27	0.000002	-1.878
RPS27A	Ribosomal protein s27a	0.000111	-1.681
RPS28	Ribosomal protein S28	0.000065	-1.616
RPS3	Ribosomal protein S3	0.000006	-1.798
RPS3A	Ribosomal protein S3A	0.001680	-1.527
RPS4X	Ribosomal protein S4, X-linked	0.001190	-1.596
RPS5	Ribosomal protein S5	0.000215	-1.53
RPS7	Ribosomal protein S7	0.000772	-1.551
RPS8	Ribosomal protein S8	0.000756	-1.521
RPSA	Ribosomal protein SA	0.000146	-1.539
RSF1	Remodeling and spacing factor 1	0.001400	1.537
RWDD1	RWD domain containing 1	0.000102	-1.527
S100A2	S100 calcium binding protein A2	0.017100	-1.615
SATB1	SATB homeobox 1	0.005160	1.504
SCAF11	SR-related CTD associated factor 11	0.000935	1.52
SCAPER	S-phase cyclin A associated protein in the ER	0.000017	1.6

SCG3	Secretogranin III	0.007440	-2.06
SDHAF4	Succinate dehydrogenase complex assembly factor 4	0.000007	-1.557
SDPR	Serum deprivation response	0.001960	1.607
SEC11A	SEC11 homolog A, signal peptidase complex subunit	0.000195	-1.561
SEC11C	SEC11 homolog C, signal peptidase complex subunit	0.003870	-1.521
SEMA3F	Semaphorin 3F	0.000313	1.646
SENP6	SUMO1/sentrin specific peptidase 6	0.000017	1.503
SERPINA11	Serpin family A member 11	0.018200	-1.632
SERPINB1	Serpin family B member 1	0.000584	-1.496
SETD2	SET domain containing 2	0.000184	1.524
SETD9	SET domain containing 9	0.002450	-1.52
SETX	Senataxin	0.000371	1.829
SLC16A1	Solute carrier family 16 member 1	0.000197	-2.176
SLC1A5	Solute carrier family 1 member 5	0.000617	-1.76
SLC25A20	Solute carrier family 25 member 20	0.000654	-1.515
SLC39A2	Solute carrier family 39 member 2	0.017600	-2.291
SLC40A1	Solute carrier family 40 member 1	0.000536	-1.939
SLC45A2	Solute carrier family 45 member 2	0.007520	-1.885
SLC6A1	Solute carrier family 6 member 1	0.013800	2.045
SLCO1A2	Solute carrier organic anion transporter family member 1A2	0.003950	1.851
SLIRP	SRA stem-loop interacting RNA binding protein	0.000258	-1.602
SMC4	Structural maintenance of chromosomes 4	0.000020	1.78
SMG1	SMG1, nonsense mediated mrna decay associated PI3K related kinase	0.017600	1.563
SMIM8	Small integral membrane protein 8	0.001130	-1.52
SMPDL3A	Sphingomyelin phosphodiesterase acid like 3A	0.018600	-1.604
SNRPF	Small nuclear ribonucleoprotein polypeptide F	0.001300	-1.518
SNRPG	Small nuclear ribonucleoprotein polypeptide G	0.000003	-2.134
SOAT1	Sterol O-acyltransferase 1	0.000015	-2.756
SOSTDC1	Sclerostin domain containing 1	0.006780	-3.213
SOX6	SRY-box 6	0.013100	1.714
SPARCL1	SPARC like 1	0.000522	1.568
SPEG	SPEG complex locus	0.003520	1.796
SPTB	Spectrin beta, erythrocytic	0.017600	1.856
SRPRB	SRP receptor beta subunit	0.000276	-1.517
SSR2	Signal sequence receptor subunit 2	0.000029	-1.594

SSR4	Signal sequence receptor subunit 4	0.000401	-1.555
STK31	Serine/threonine kinase 31	0.017100	1.597
SUSD5	Sushi domain containing 5	0.002350	1.631
SYNE2	Spectrin repeat containing nuclear envelope protein 2	0.007070	1.537
SZT2	Seizure threshold 2 homolog (mouse)	0.005070	1.585
TAOK2	TAO kinase 2	0.000034	1.526
TATDN1	Tatd dnase domain containing 1	0.000026	-1.834
TBC1D16	TBC1 domain family member 16	0.002730	1.569
TCF20	Transcription factor 20	0.002460	1.567
TCTN2	Tectonic family member 2	0.000319	-1.644
TENM3	Teneurin transmembrane protein 3	0.001830	1.739
TET2	Tet methylcytosine dioxygenase 2	0.012500	1.541
TM6SF2	Transmembrane 6 superfamily member 2	0.017000	-1.607
TMED8	Transmembrane p24 trafficking protein family member 8	0.001540	-1.606
TMEM205	Transmembrane protein 205	0.000226	-1.53
TMEM208	Transmembrane protein 208	0.000042	-1.604
TMEM256	Transmembrane protein 256	0.000091	-1.546
TMEM60	Transmembrane protein 60	0.000277	-1.531
TMEM64	Transmembrane protein 64	0.000213	-1.864
TNFRSF11B	TNF receptor superfamily member 11b	0.004140	1.822
TNFRSF4	TNF receptor superfamily member 4	0.003040	2.106
TNFSF10	Tumor necrosis factor superfamily member 10	0.014900	1.609
TNRC6A	Trinucleotide repeat containing 6A	0.001580	1.557
TOMM7	Translocase of outer mitochondrial membrane 7	0.000005	-1.634
TPT1	Tumor protein, translationally-controlled 1	0.000003	-1.723
TRIP11	Thyroid hormone receptor interactor 11	0.009170	1.67
TRMT112	Trna methyltransferase 11-2 homolog (S. Cerevisiae)	0.000075	-1.517
TRMT44	Trna methyltransferase 44 homolog (S. Cerevisiae)	0.003190	1.514
TTC36	Tetratricopeptide repeat domain 36	0.014000	-1.602
TUBA1C	Tubulin alpha 1c	0.011200	-1.826
UACA	Uveal autoantigen with coiled-coil domains and ankyrin repeats	0.000072	1.821
UBL5	Ubiquitin like 5	0.000001	-1.646
UPK3BL	Uroplakin 3B-like	0.000765	-2.921
UQCRH	Ubiquinol-cytochrome c reductase hinge protein	0.002450	-1.562
UXT	Ubiquitously expressed prefoldin like chaperone	0.003090	-1.508

VTI1A	Vesicle transport through interaction with t-snares 1A	0.000092	-1.501
WDR12	WD repeat domain 12	0.000567	-1.518
ZC3H11A	Zinc finger CCCH-type containing 11A	0.000026	1.522
ZC3H12A	Zinc finger CCCH-type containing 12A	0.009710	1.666
ZEB1	Zinc finger E-box binding homeobox 1	0.000319	1.573
ZHX1	Zinc fingers and homeoboxes 1	0.000001	1.549
ZNF182	Zinc finger protein 182	0.000743	1.568
ZNF318	Zinc finger protein 318	0.000131	1.617
ZNF41	Zinc finger protein 41	0.001480	1.504
ZNF605	Zinc finger protein 605	0.000446	1.725
ZNF608	Zinc finger protein 608	0.000442	1.687
ZNF644	Zinc finger protein 644	0.000004	1.508

[1] fold changes are up or down in control fed animals compared to PUFA supplemented

Table S2. Differentially expressed genes in Control, non-pregnant, high diet versus PUFA, non-pregnant, high diet

Symbol	Entrez gene name	p-value	Fold Change[1]
ABCD3	ATP binding cassette subfamily D member 3	9.56E-05	-2.083
ABHD14B	Abhydrolase domain containing 14B	0.00154	-1.81
ABRACL	ABRA C-terminal like	2.64E-06	-2.58
ACER2	Alkaline ceramidase 2	0.00156	2.143
ACER3	Alkaline ceramidase 3	0.000397	-1.946
ACO1	Aconitase 1	0.000464	-1.575
ACP5	Acid phosphatase 5, tartrate resistant	3.77E-05	-3.679
ACSL3	Acyl-coa synthetase long-chain family member 3	7.48E-06	-2.006
ACSL4	Acyl-coa synthetase long-chain family member 4	2.68E-06	-4.047
ACY1	Aminoacylase 1	0.00273	-1.691
ADCK5	Aarf domain containing kinase 5	0.00214	1.774
ADIPOR2	Adiponectin receptor 2	0.00191	-1.77
ADSS	Adenylosuccinate synthase	1.21E-05	-2.17
AFMID	Arylformamidase	0.00223	-3.454
AIM1	Absent in melanoma 1	0.000175	-2.26
AKAP6	A-kinase anchoring protein 6	0.000603	-4.235
AKIRIN1	Akirin 1	0.00156	-1.596
AKR1B1	Aldo-keto reductase family 1 member B	5.09E-05	-3.19

ALDH18A1	Aldehyde dehydrogenase 18 family member A1	3.58E-05	-2.425
ALDH3A2	Aldehyde dehydrogenase 3 family member A2	0.00033	-1.934
ALG8	ALG8, alpha-1,3-glucosyltransferase	4.21E-05	-1.771
AMDHD2	Amidohydrolase domain containing 2	0.000656	-1.917
ANKIB1	Ankyrin repeat and IBR domain containing 1	7.8E-06	-2.175
ANKRD37	Ankyrin repeat domain 37	0.00169	-4.002
ANPEP	Alanyl aminopeptidase, membrane	3.74E-09	-11.771
AP1S2	Adaptor related protein complex 1 sigma 2 subunit	0.000233	-2.159
AP3B1	Adaptor related protein complex 3 beta 1 subunit	0.000146	-1.523
AP3M1	Adaptor related protein complex 3 mu 1 subunit	0.000115	-1.783
AP3S2	Adaptor related protein complex 3 sigma 2 subunit	0.000759	-1.524
APBB3	Amyloid beta precursor protein binding family B member 3	0.00108	2.348
ARAF	A-Raf proto-oncogene, serine/threonine kinase	0.000108	-1.904
ARHGEF15	Rho guanine nucleotide exchange factor 15	0.000611	1.998
ARHGEF16	Rho guanine nucleotide exchange factor 16	9.15E-06	2.111
ARMT1	Acidic residue methyltransferase 1	0.00035	-1.983
ARSJ	Arylsulfatase family member J	0.00192	-1.784
ASGR2	Asialoglycoprotein receptor 2	8.52E-06	-4.926
ASPN	Asporin	0.00244	-2.366

ASRGL1	Asparaginase like 1	5.57E-06	-2.958
ATP1B3	Atpase Na+/K+ transporting subunit beta 3	0.000378	-1.996
ATP6V1A	Atpase H+ transporting V1 subunit A	0.000743	-1.575
ATP6V1H	Atpase H+ transporting V1 subunit H	0.000266	-1.54
B4GALNT2	Beta-1,4-N-acetyl-galactosaminyltransferase 2	0.000721	-2.095
BCKDHB	Branched chain keto acid dehydrogenase E1 subunit beta	0.000462	-2.101
BCO2	Beta-carotene oxygenase 2	0.000338	-2.314
BCOR	BCL6 corepressor	0.00236	1.684
BDH1	3-hydroxybutyrate dehydrogenase, type 1	0.00153	-3.235
BSPRY	B-box and SPRY domain containing	0.000693	-1.826
C1orf21	Chromosome 1 open reading frame 21	1.16E-05	-2.474
C2orf76	Chromosome 2 open reading frame 76	0.000859	-2.075
C4orf32	Chromosome 4 open reading frame 32	6.12E-05	-2.445
CACNG4	Calcium voltage-gated channel auxiliary subunit gamma 4	0.000515	2.729
CAD	Carbamoyl-phosphate synthetase 2, aspartate transcarbamylase, and dihydroorotase	0.000324	1.7
CAMK2B	Calcium/calmodulin dependent protein kinase II beta	0.000375	-3.614
CANX	Calnexin	0.00253	-1.542
CAPZA2	Capping actin protein of muscle Z-line alpha subunit 2	0.00078	-1.646
CCDC150	Coiled-coil domain containing 150	0.00171	2.279

CCNB1	Cyclin B1	0.000355	-4.96
CDC123	Cell division cycle 123	0.000646	-1.555
CDC42	Cell division cycle 42	0.00178	-1.657
CFAP36	Cilia and flagella associated protein 36	0.000131	-1.695
CGNL1	Cingulin like 1	0.000785	2.094
CHD5	Chromodomain helicase DNA binding protein 5	0.00227	2.824
CKS2	CDC28 protein kinase regulatory subunit 2	0.000849	-2.295
CLDN11	Claudin 11	0.00077	3.585
CMBL	Carboxymethylenebutenolidase homolog	6.16E-05	-2.887
CNKSR3	CNKSR family member 3	0.000411	-1.697
COPS9	COP9 signalosome subunit 9	0.000904	-1.756
COQ10A	Coenzyme Q10A	0.000136	-2.125
COX11	COX11, cytochrome c oxidase copper chaperone	0.000504	-1.825
CROCC	Ciliary rootlet coiled-coil, rootletin	0.000532	2.62
CRTC2	CREB regulated transcription coactivator 2	0.000712	1.524
CSTB	Cystatin B	0.000828	-1.994
CTC1	CTS telomere maintenance complex component 1	0.00161	2.069
CX3CL1	C-X3-C motif chemokine ligand 1	0.00148	1.732
CYB5B	Cytochrome b5 type B	4.77E-08	-3.199

CYB5R4	Cytochrome b5 reductase 4	9.54E-05	-1.843
DBI	Diazepam binding inhibitor, acyl-coa binding protein	2.99E-07	-2.788
DDT	D-dopachrome tautomerase	0.00212	-1.965
DENND1A	DENN domain containing 1A	0.000666	1.652
DFFA	DNA fragmentation factor subunit alpha	0.00099	-1.602
DGAT2	Diacylglycerol O-acyltransferase 2	0.000398	-4.103
DGKI	Diacylglycerol kinase iota	4.93E-06	-13.433
DLL4	Delta like canonical Notch ligand 4	0.000338	1.75
DMPK	Dystrophia myotonica protein kinase	0.000609	-3.061
DNAJA2	Dnaj heat shock protein family (Hsp40) member A2	0.00104	-1.405
DNAJC16	Dnaj heat shock protein family (Hsp40) member C16	0.00025	-1.671
DPY19L4	Dpy-19 like 4 (C. Elegans)	0.00146	-2.353
EBAG9	Estrogen receptor binding site associated, antigen, 9	0.00165	-1.593
ECHDC1	Ethylmalonyl-coa decarboxylase 1	0.0013	-1.528
ELOVL5	ELOVL fatty acid elongase 5	2.18E-06	-2.312
ENDOG	Endonuclease G	0.00191	-1.742
ENPP1	Ectonucleotide pyrophosphatase/phosphodiesterase 1	5.09E-05	-2.248
ENPP3	Ectonucleotide pyrophosphatase/phosphodiesterase 3	0.000015	-4.781
ENPP4	Ectonucleotide pyrophosphatase/phosphodiesterase 4 (putative)	0.00253	-2.021

ENPP5	Ectonucleotide pyrophosphatase/phosphodiesterase 5 (putative)	0.000302	-2.089
ENTPD2	Ectonucleoside triphosphate diphosphohydrolase 2	0.000169	-3.417
EPHX2	Epoxide hydrolase 2	0.0015	-2.533
EPN3	Epsin 3	0.000836	-2.65
ERGIC2	ERGIC and golgi 2	0.00117	-1.704
ERO1A	Endoplasmic reticulum oxidoreductase 1 alpha	8.1E-07	-2.741
EVC2	Evc ciliary complex subunit 2	0.00177	1.99
EXOC6	Exocyst complex component 6	0.000177	-1.568
FAM162A	Family with sequence similarity 162 member A	2.85E-08	-4.478
FAM20A	Family with sequence similarity 20 member A	0.000802	2.122
FAM3C	Family with sequence similarity 3 member C	0.0015	-1.641
FAM43A	Family with sequence similarity 43 member A	0.000322	-3.128
FAM84A	Family with sequence similarity 84 member A	0.000253	-2.796
FBP1	Fructose-bisphosphatase 1	0.0017	-5.771
FDX1	Ferredoxin 1	0.000477	-1.64
FGD5	FYVE, rhogef and PH domain containing 5	0.00232	1.794
FGL1	Fibrinogen like 1	0.00223	2.307
FKBP5	FK506 binding protein 5	0.00021	-2.073
FOXRED2	FAD dependent oxidoreductase domain containing 2	0.000001	-8.906

FUCA1	Fucosidase, alpha-L- 1, tissue	0.000737	-1.673
GBE1	Glucan (1,4-alpha-), branching enzyme 1	0.00137	-1.494
GBGT1	Globoside alpha-1,3-N-acetylgalactosaminyltransferase 1	2.18E-06	-2.86
GDAP1	Ganglioside induced differentiation associated protein 1	0.00261	-1.821
GDPD1	Glycerophosphodiester phosphodiesterase domain containing 1	1.42E-07	-3.901
GGT5	Gamma-glutamyltransferase 5	0.00203	2.496
GJB1	Gap junction protein beta 1	0.000163	-3.851
GJB3	Gap junction protein beta 3	2.06E-05	-3.202
GJB5	Gap junction protein beta 5	0.000211	-6.514
GK5	Glycerol kinase 5 (putative)	7.67E-11	-5.79
GLOD4	Glyoxalase domain containing 4	0.00245	-1.532
GLRX	Glutaredoxin	0.00094	-2.153
GLUD1	Glutamate dehydrogenase 1	0.00161	-1.441
GM2A	GM2 ganglioside activator	0.000024	-4.031
GNG10	G protein subunit gamma 10	0.00194	-2.29
GNMT	Glycine N-methyltransferase	0.000793	-2.51
GNPNAT1	Glucosamine-phosphate N-acetyltransferase 1	0.00104	-1.807
GNS	Glucosamine (N-acetyl)-6-sulfatase	0.000233	-1.628
GOLPH3	Golgi phosphoprotein 3	0.000126	-1.613

GRIN1	Glutamate ionotropic receptor NMDA type subunit 1	0.000326	-2.928
Gstt1	Glutathione S-transferase, theta 1	0.0017	-1.922
Gstt3	Glutathione S-transferase, theta 3	1.71E-07	-8.322
HAUS5	HAUS augmin like complex subunit 5	0.000611	1.529
HDAC7	Histone deacetylase 7	0.00172	1.535
HENMT1	HEN1 methyltransferase homolog 1	0.00112	-3.288
HK2	Hexokinase 2	0.000447	-4.172
HOMER1	Homer scaffolding protein 1	7.95E-05	-2.109
HSD17B4	Hydroxysteroid 17-beta dehydrogenase 4	0.000178	-1.806
IGHMBP2	Immunoglobulin mu binding protein 2	0.00228	1.898
IKBKAP	Inhibitor of kappa light polypeptide gene enhancer in B-cells, kinase complex-associated protein	0.000304	-1.901
IKBKB	Inhibitor of kappa light polypeptide gene enhancer in B-cells, kinase beta	7.79E-05	1.819
IL17RC	Interleukin 17 receptor C	5.49E-06	1.905
ILVBL	Ilvb acetolactate synthase like	1.34E-05	-2.466
IPO8	Importin 8	0.0013	-1.456
IST1	IST1, ESCRT-III associated factor	0.00128	-1.687
ITGA2B	Integrin subunit alpha 2b	0.000687	3.807
JAK3	Janus kinase 3	1.19E-08	-4.718
JARID2	Jumonji and AT-rich interaction domain containing 2	5.55E-06	-3.461

Kdm1b	Lysine (K)-specific demethylase 1B	0.00283	-1.6
KIAA0513	Kiaa0513	0.00126	1.968
KIAA2013	Kiaa2013	0.00279	-1.41
KIFC3	Kinesin family member C3	0.00193	2.224
KLHL7	Kelch like family member 7	0.000825	-1.916
KRT24	Keratin 24	0.000436	-4.738
LDHA	Lactate dehydrogenase A	0.000314	-2.783
LENG8	Leukocyte receptor cluster member 8	7.62E-05	2.869
LIMK2	LIM domain kinase 2	0.00162	-1.75
LINS1	Lines homolog 1	6.34E-05	-2.119
LMBR1L	Limb development membrane protein 1 like	0.000969	1.782
LPL	Lipoprotein lipase	0.000521	-6.634
LRRC71	Leucine rich repeat containing 71	0.00221	2.336
LRRK1	Leucine rich repeat kinase 1	0.00233	1.709
LTF	Lactotransferrin	2.13E-05	-8.868
LYRM2	LYR motif containing 2	0.000215	-1.996
MAPKAPK2	Mitogen-activated protein kinase-activated protein kinase 2	0.00232	-1.432
MAST2	Microtubule associated serine/threonine kinase 2	0.000783	1.771
MBOAT2	Membrane bound O-acyltransferase domain containing 2	8.44E-07	-2.712

MCCC2	Methylcrotonoyl-coa carboxylase 2	1.03E-06	-3.492
MED25	Mediator complex subunit 25	0.000973	1.685
MEMO1	Mediator of cell motility 1	0.0015	-1.724
MESDC1	Mesoderm development candidate 1	2.67E-05	-1.952
METAP1	Methionyl aminopeptidase 1	0.00024	-1.856
MICAL2	Microtubule associated monooxygenase, calponin and LIM domain containing 2	0.00281	-2.225
MINPP1	Multiple inositol-polyphosphate phosphatase 1	0.000409	-1.69
MKNK1	MAP kinase interacting serine/threonine kinase 1	0.000609	-1.605
MKS1	Meckel syndrome, type 1	0.00277	1.687
MLH1	Mutl homolog 1	0.00255	1.58
MOG	Myelin oligodendrocyte glycoprotein	0.00179	1.771
MOXD1	Monooxygenase DBH like 1	0.00015	-4.396
MPC1	Mitochondrial pyruvate carrier 1	0.00258	-1.825
MPP7	Membrane palmitoylated protein 7	7.39E-08	-4.03
MRFAP1	Morf4 family associated protein 1	4.98E-05	-1.866
MRPL19	Mitochondrial ribosomal protein L19	0.000171	-1.676
MRPS36	Mitochondrial ribosomal protein S36	2.76E-12	-13.115
MSX1	Msh homeobox 1	0.000547	-2.503
MYO1B	Myosin IB	0.000244	-1.928

MYRIP	Myosin VIIA and Rab interacting protein	0.00109	2.458
NCAPH2	Non-SMC condensin II complex subunit H2	0.00257	1.422
NDFIP2	Nedd4 family interacting protein 2	0.00149	-1.951
NEBL	Nebulette	0.00135	-2.517
NLK	Nemo like kinase	0.000224	-1.687
NOL10	Nucleolar protein 10	0.00254	-1.523
NSDHL	NAD(P) dependent steroid dehydrogenase-like	0.000391	-1.819
NUDCD3	Nudc domain containing 3	2.86E-05	-1.844
NUP205	Nucleoporin 205	0.00107	-1.519
NUP88	Nucleoporin 88	0.000418	-1.74
NXNL2	Nucleoredoxin-like 2	8.39E-10	-7.045
NXT2	Nuclear transport factor 2 like export factor 2	0.00248	-1.844
ODC1	Ornithine decarboxylase 1	6.19E-10	-4.728
OGDH	Oxoglutarate dehydrogenase	9.14E-08	-3.402
OSBPL7	Oxysterol binding protein like 7	0.00244	1.804
OSTM1	Osteopetrosis associated transmembrane protein 1	3.41E-07	-2.599
P2RX3	Purinergic receptor P2X 3	0.00165	-3.3
PACSIN1	Protein kinase C and casein kinase substrate in neurons 1	0.00229	-3.274
PAN2	PAN2 poly(A) specific ribonuclease subunit	0.000973	1.585

PAQR8	Progestin and adipoq receptor family member 8	9.78E-05	-2.497
PCCA	Propionyl-coa carboxylase alpha subunit	0.000809	-1.731
PDCD4	Programmed cell death 4 (neoplastic transformation inhibitor)	2.15E-05	-1.907
PDXK	Pyridoxal (pyridoxine, vitamin B6) kinase	0.000057	-2.763
PDZD8	PDZ domain containing 8	0.00146	-1.618
PDZK1	PDZ domain containing 1	1.88E-05	-4.019
PDZK1IP1	PDZK1 interacting protein 1	3.86E-06	-8.471
PEPD	Peptidase D	2.87E-06	-4.28
PEX1	Peroxisomal biogenesis factor 1	8.36E-06	-1.938
PFKFB2	6-phosphofructo-2-kinase/fructose-2,6-biphosphatase 2	8.16E-06	-3.781
PHGDH	Phosphoglycerate dehydrogenase	0.000389	-4.881
PHLDB2	Pleckstrin homology like domain family B member 2	0.00248	1.847
PIK3AP1	Phosphoinositide-3-kinase adaptor protein 1	0.00148	-1.927
PKD1	Polycystin 1, transient receptor potential channel interacting	0.00161	2.35
PLEKHG1	Pleckstrin homology and rhogef domain containing G1	0.0024	-2.385
PLLP	Plasmolipin	3.96E-05	-2.986
PNPLA6	Patatin like phospholipase domain containing 6	0.0021	1.62
POLE2	DNA polymerase epsilon 2, accessory subunit	0.00114	-2.248
POLG	DNA polymerase gamma, catalytic subunit	0.00152	1.734

PPM1G	Protein phosphatase, Mg2+/Mn2+ dependent 1G	0.00151	-1.438
PPM1M	Protein phosphatase, Mg2+/Mn2+ dependent 1M	0.0028	-1.574
PPP2CB	Protein phosphatase 2 catalytic subunit beta	0.000349	-1.525
PRKAG2	Protein kinase AMP-activated non-catalytic subunit gamma 2	0.000733	-1.585
PRNP	Prion protein	0.00196	-1.626
PRPSAP1	Phosphoribosyl pyrophosphate synthetase associated protein 1	0.000452	-1.769
PSAT1	Phosphoserine aminotransferase 1	1.37E-05	-7.934
PSRC1	Proline and serine rich coiled-coil 1	0.00122	1.753
PTGER4	Prostaglandin E receptor 4	0.00125	-2.491
PTK2B	Protein tyrosine kinase 2 beta	0.00223	2.02
PTP4A1	Protein tyrosine phosphatase type IVA, member 1	0.00123	-1.463
PTPN1	Protein tyrosine phosphatase, non-receptor type 1	5.72E-05	-1.999
PTPN23	Protein tyrosine phosphatase, non-receptor type 23	0.00124	1.637
RAB10	RAB10, member RAS oncogene family	0.00117	-1.512
RAB8B	RAB8B, member RAS oncogene family	0.00122	-1.94
RABGEF1	RAB guanine nucleotide exchange factor 1	1.19E-06	-1.682
RCN1	Reticulocalbin 1	6.95E-07	-3.291
RFWD2	Ring finger and WD repeat domain 2	0.00184	-1.459
RHOBTB3	Rho related BTB domain containing 3	0.000529	-2.053

RIDA	Reactive intermediate imine deaminase A homolog	1.57E-06	-2.15
RNF11	Ring finger protein 11	0.000894	-1.464
RNF115	Ring finger protein 115	0.00283	-1.481
RNF122	Ring finger protein 122	0.000128	-3.013
RNF14	Ring finger protein 14	0.0023	-1.515
RNF180	Ring finger protein 180	0.000164	-2.012
RNF185	Ring finger protein 185	0.00139	-1.388
RORC	RAR related orphan receptor C	0.00126	2.262
RSPRY1	Ring finger and SPRY domain containing 1	2.3E-06	-2.295
RTCA	RNA 3'-terminal phosphate cyclase	0.0019	-1.561
RTKN	Rhotekin	0.00211	1.677
RUBCN	RUN and cysteine rich domain containing beclin 1 interacting protein	0.00087	1.985
RWDD4	RWD domain containing 4	8.52E-07	-2.311
S100A10	S100 calcium binding protein A10	0.000327	-1.842
SAA1	Serum amyloid A1	6.57E-06	-27.764
SAT1	Spermidine/spermine N1-acetyltransferase 1	0.000959	-1.925
SCFD1	Sec1 family domain containing 1	0.00234	-1.43
SEC31A	SEC31 homolog A, COPII coat complex component	0.000148	-1.911
SEMA3G	Semaphorin 3G	0.000366	2.862

SEPT4	Septin 4	0.00192	-1.925
SH2D3A	SH2 domain containing 3A	0.00165	1.962
SH3BP2	SH3 domain binding protein 2	0.00161	1.79
SH3GLB2	SH3 domain containing GRB2 like endophilin B2	0.00244	1.594
SIDT1	SID1 transmembrane family member 1	0.00172	3.183
SLC13A5	Solute carrier family 13 member 5	1.99E-06	-51.242
SLC28A3	Solute carrier family 28 member 3	0.000538	-3.486
SLC30A5	Solute carrier family 30 member 5	0.00099	-1.95
SLC31A1	Solute carrier family 31 member 1	0.00146	-2.03
SLC31A2	Solute carrier family 31 member 2	0.000052	-2.712
SLC37A4	Solute carrier family 37 member 4	3.2E-06	-2.775
SLC38A3	Solute carrier family 38 member 3	0.00271	2.825
SLC43A3	Solute carrier family 43 member 3	0.000159	-3.057
SLC50A1	Solute carrier family 50 member 1	5.38E-06	-2.683
SMAD3	SMAD family member 3	0.00114	1.622
SMARCE1	SWI/SNF related, matrix associated, actin dependent regulator of chromatin, subfamily e, member 1	0.00106	-1.5
SMG9	SMG9, nonsense mediated mrna decay factor	0.00163	1.705
SMIM14	Small integral membrane protein 14	0.00173	-1.828
SMOX	Spermine oxidase	0.00101	-2.291

SMS	Spermine synthase	0.000947	-2.648
SMTNL2	Smoothelin like 2	3.92E-06	-2.567
SNAPC4	Small nuclear RNA activating complex polypeptide 4	0.00012	2.439
SNPH	Syntaphilin	0.000696	2.337
SPC24	SPC24, NDC80 kinetochore complex component	2.96E-05	-4.719
SPDEF	SAM pointed domain containing ETS transcription factor	0.00227	-5.382
SPHK1	Sphingosine kinase 1	0.000156	-3.036
SPIN1	Spindlin 1	3.74E-05	-1.988
SPIRE1	Spire type actin nucleation factor 1	0.00176	1.548
SPOPL	Speckle type BTB/POZ protein like	0.00283	-1.941
SPOUT1	SPOUT domain containing methyltransferase 1	0.000257	-1.948
SPPL3	Signal peptide peptidase like 3	0.000426	-1.794
SRPX	Sushi repeat containing protein, X-linked	0.00114	-2.55
SRSF9	Serine and arginine rich splicing factor 9	4.31E-06	-1.862
SS18L1	SS18L1, nbaf chromatin remodeling complex subunit	3.69E-06	-3.485
ST5	Suppression of tumorigenicity 5	0.00022	1.746
STAT6	Signal transducer and activator of transcription 6	0.00232	1.563
STX1A	Syntaxin 1A	0.00267	2.306
Sult1a1	Sulfotransferase family 1A, phenol-preferring, member 1	2.52E-06	-3.45

SYMPK	Symplekin	0.00121	1.498
SYNE4	Spectrin repeat containing nuclear envelope family member 4	0.00191	-2.339
SYNJ1	Synaptojanin 1	0.000993	1.799
TBC1D30	TBC1 domain family member 30	0.00169	-2.116
TBKBP1	TBK1 binding protein 1	0.00216	1.769
TCAF2	TRPM8 channel associated factor 2	7.14E-10	-3.477
TDGF1	Teratocarcinoma-derived growth factor 1	0.00102	-14.059
TES	Testin LIM domain protein	0.00181	-1.63
THSD1	Thrombospondin type 1 domain containing 1	0.000493	2.021
TK1	Thymidine kinase 1	0.000082	-2.124
TM9SF3	Transmembrane 9 superfamily member 3	2.41E-05	-1.959
TMED3	Transmembrane p24 trafficking protein 3	0.000217	-1.763
TNFRSF11B	TNF receptor superfamily member 11b	0.000323	3.706
TNFRSF4	TNF receptor superfamily member 4	4.44E-05	5.97
TNKS1BP1	Tankyrase 1 binding protein 1	0.000496	1.678
TNS2	Tensin 2	5.04E-05	1.974
TOR1B	Torsin family 1 member B	0.000119	-1.923
TPMT	Thiopurine S-methyltransferase	0.00014	-1.991
TRIM9	Tripartite motif containing 9	4.75E-07	-36.052

TST	Thiosulfate sulfurtransferase	0.000177	-3.209
TTC22	Tetratricopeptide repeat domain 22	0.00261	-2.373
TXNRD1	Thioredoxin reductase 1	5.86E-10	-5.802
UCP2	Uncoupling protein 2	0.000574	-2.617
UPK1B	Uroplakin 1B	1.43E-05	-14.555
USP46	Ubiquitin specific peptidase 46	0.000818	-1.725
VMA21	VMA21 vacuolar H+-atpase homolog (S. Cerevisiae)	8.36E-05	-1.748
VNN2	Vanin 2	0.000384	-7.754
WNT11	Wnt family member 11	1.67E-05	-4.396
WSB2	WD repeat and SOCS box containing 2	0.000153	-1.501
YIPF4	Yip1 domain family member 4	0.000904	-1.614
ZC3H12A	Zinc finger CCCH-type containing 12A	0.000785	3.124
ZFP57	ZFP57 zinc finger protein	0.00181	1.943

[1] Fold changes are up or down in control animals compared with PUFA supplemented animals

Table S3. Differentially expressed genes in Control, pregnant, high diet versus PUFA, pregnant, high diet.

Symbol	Entrez gene name	p- value	Fold Change[1]
GJB2	gap junction protein beta 2	1.38E-06	4.90

[1] Fold changes are up or down in control fed animals compared to PUFA

Table S4. Differentially expressed genes in Control, non-pregnant, low diet versus Control, non-pregnant, high diet

Symbol	Entrez Gene Name	p-value	Fold Change[1]
MX2	MX dynamin like gtpase 2	5.13E-05	-77.525
RSAD2	Radical S-adenosyl methionine domain containing 2	4.64E-05	-56.008
ISG15	ISG15 ubiquitin-like modifier	3.58E-05	-55.347
IFIT1	Interferon induced protein with tetratricopeptide repeats 1	6.16E-05	-38.724
IFIT3	Interferon induced protein with tetratricopeptide repeats 3	6.38E-05	-31.198
MX1	MX dynamin like gtpase 1	6.93E-05	-17.734
ZBP1	Z-DNA binding protein 1	8.93E-05	-17.531
USP18	Ubiquitin specific peptidase 18	4.45E-05	-16.521
SLFN11	Schlafen family member 11	2.91E-05	-14.608
CMPK2	Cytidine/uridine monophosphate kinase 2	7.22E-05	-13.452
OAS1	2'-5'-oligoadenylate synthetase 1	0.000122	-10.905
HERC6	HECT and RLD domain containing E3 ubiquitin protein ligase family member 6	8.78E-05	-10.41
HERC5	HECT and RLD domain containing E3 ubiquitin protein ligase 5	0.000092	-5.624

[1] Fold changes are up or down in control, non-pregnant, low diet compared to control, non-pregnant, high diet

Table S5. Differentially expressed genes in Control, pregnant, low diet versus Control, pregnant, high diet

Symbol	Entrez gene name	p- value	Fold Change[1]
OXTR	Oxytocin receptor	2.46E-06	-10.26
GJB2	Gap junction protein beta 2	8.64E-06	-4.71

[1] Fold changes are up or down in control, pregnant, low diet compared to control, pregnant, high diet

Table S6. Differentially expressed genes in PUFA, not-pregnant, low diet verses PUFA, not-pregnant, high diet

Symbol	Entrez gene name	p-value	Fold Change[1]
ABAT	4-aminobutyrate aminotransferase	0.009	-1.952
ABCA3	ATP binding cassette subfamily A member 3	0.002	-1.556
ABCC1	ATP binding cassette subfamily C member 1	0.000	-3.544
ABCC3	ATP binding cassette subfamily C member 3	0.004	-2.157
ABCD3	ATP binding cassette subfamily D member 3	0.000	-2.351
ABCF2	ATP binding cassette subfamily F member 2	0.001	-1.559
ABCG2	ATP binding cassette subfamily G member 2 (Junior blood group)	0.001	2.105
ABHD17B	Abhydrolase domain containing 17B	0.000	-1.622
ABHD6	Abhydrolase domain containing 6	0.000	-1.733
ABHD8	Abhydrolase domain containing 8	0.012	1.530
ABLIM2	Actin binding LIM protein family member 2	0.010	-1.810
ABRACL	ABRA C-terminal like	0.000	-2.338
ACBD6	Acyl-coa binding domain containing 6	0.005	1.581
ACE	Angiotensin I converting enzyme	0.003	1.811
ACER2	Alkaline ceramidase 2	0.005	1.843
ACER3	Alkaline ceramidase 3	0.002	-1.650
ACKR4	Atypical chemokine receptor 4	0.005	-5.051
ACOX2	Acyl-coa oxidase 2	0.000	2.225
ACP2	Acid phosphatase 2, lysosomal	0.000	-1.842
ACP5	Acid phosphatase 5, tartrate resistant	0.000	-2.748
ACPP	Acid phosphatase, prostate	0.007	-2.014
ACSL3	Acyl-coa synthetase long-chain family member 3	0.000	-2.081
ACSL4	Acyl-coa synthetase long-chain family member 4	0.000	-6.554
ACSM1	Acyl-coa synthetase medium-chain family member 1	0.006	5.234
ACSM3	Acyl-coa synthetase medium-chain family member 3	0.005	2.559
ACSS2	Acyl-coa synthetase short-chain family member 2	0.000	-3.946
ACTR3B	ARP3 actin related protein 3 homolog B	0.003	-1.855
ADAL	Adenosine deaminase like	0.001	1.639
ADAM9	ADAM metallopeptidase domain 9	0.009	-1.653
ADAR	Adenosine deaminase, RNA specific	0.011	-2.158
ADCY9	Adenylate cyclase 9	0.000	-2.166
ADIPOR2	Adiponectin receptor 2	0.002	-1.637

ADIRF	Adipogenesis regulatory factor	0.004	1.956
ADK	Adenosine kinase	0.003	-1.574
ADSS	Adenylosuccinate synthase	0.000	-2.392
AEBP2	AE binding protein 2	0.001	-1.505
AFMID	Arylformamidase	0.000	-4.154
AGTPBP1	ATP/GTP binding protein 1	0.012	-1.640
AIDA	Axin interactor, dorsalization associated	0.001	-1.765
AIFM1	Apoptosis inducing factor, mitochondria associated 1	0.002	-1.745
AIM1	Absent in melanoma 1	0.000	-2.362
AKAP4	A-kinase anchoring protein 4	0.003	4.981
AKAP6	A-kinase anchoring protein 6	0.000	-6.766
AKR1B1	Aldo-keto reductase family 1 member B	0.000	-2.482
ALAD	Aminolevulinate dehydratase	0.003	1.544
ALAS1	5'-aminolevulinate synthase 1	0.001	-2.318
ALDH18A1	Aldehyde dehydrogenase 18 family member A1	0.000	-2.193
ALDH3A2	Aldehyde dehydrogenase 3 family member A2	0.002	-1.643
ALG3	ALG3, alpha-1,3- mannosyltransferase	0.002	-1.759
ALG8	ALG8, alpha-1,3-glucosyltransferase	0.000	-1.651
ALOX12	Arachidonate 12-lipoxygenase, 12S type	0.000	-4.207
ALPL	Alkaline phosphatase, liver/bone/kidney	0.010	-1.531
AMACR	Alpha-methylacyl-coa racemase	0.000	-1.899
AMD1	Adenosylmethionine decarboxylase 1	0.000	-1.544
AMDHD2	Amidohydrolase domain containing 2	0.000	-1.933
AMIGO1	Adhesion molecule with Ig like domain 1	0.000	1.884
AMMECR1	Alport syndrome, mental retardation, midface hypoplasia and elliptocytosis chromosomal region gene 1	0.002	-1.866
AMPD3	Adenosine monophosphate deaminase 3	0.000	-3.784
ANKIB1	Ankyrin repeat and IBR domain containing 1	0.000	-2.542
ANKMY2	Ankyrin repeat and MYND domain containing 2	0.004	-1.527
ANPEP	Alanyl aminopeptidase, membrane	0.000	-15.379
AP1S2	Adaptor related protein complex 1 sigma 2 subunit	0.009	-1.586
AP3M1	Adaptor related protein complex 3 mu 1 subunit	0.001	-1.514
APBB1	Amyloid beta precursor protein binding family B member 1	0.000	1.619
APOA1	Apolipoprotein A1	0.001	2.831
AQP8	Aquaporin 8	0.001	-57.378
ARAF	A-Raf proto-oncogene, serine/threonine kinase	0.000	-2.019

ARAP2	Arfgap with rhogap domain, ankyrin repeat and PH domain 2	0.000	-2.514
ARG2	Arginase 2	0.002	-2.330
ARHGAP18	Rho gtpase activating protein 18	0.008	-1.586
ARHGAP26	Rho gtpase activating protein 26	0.000	-2.734
ARHGEF37	Rho guanine nucleotide exchange factor 37	0.000	-2.490
ARL3	ADP ribosylation factor like gtpase 3	0.000	2.089
ARL6IP4	ADP ribosylation factor like gtpase 6 interacting protein 4	0.002	1.529
ARMCX6	Armadillo repeat containing, X-linked 6	0.006	1.582
ARMT1	Acidic residue methyltransferase 1	0.001	-1.703
ARPC1A	Actin related protein 2/3 complex subunit 1A	0.000	1.563
ARPC5L	Actin related protein 2/3 complex subunit 5 like	0.007	-1.540
ARRDC4	Arrestin domain containing 4	0.000	-1.637
ARSJ	Arylsulfatase family member J	0.000	-1.943
ASGR1	Asialoglycoprotein receptor 1	0.012	2.115
ASGR2	Asialoglycoprotein receptor 2	0.000	-4.309
ASPN	Asporin	0.007	-1.907
ASRGL1	Asparaginase like 1	0.000	-2.387
ATAD1	Atpase family, AAA domain containing 1	0.002	-1.898
ATG4D	Autophagy related 4D cysteine peptidase	0.002	-1.549
ATP13A5	Atpase 13A5	0.006	-2.065
ATP1B3	Atpase Na+/K+ transporting subunit beta 3	0.008	-1.556
ATP2A2	Atpase sarcoplasmic/endoplasmic reticulum Ca2+ transporting 2	0.002	-1.496
ATP6AP2	Atpase H+ transporting accessory protein 2	0.000	-1.896
ATP6V0A2	Atpase H+ transporting V0 subunit a2	0.002	-2.316
ATP6V0A4	Atpase H+ transporting V0 subunit a4	0.002	-3.408
ATP6V1E1	Atpase H+ transporting V1 subunit E1	0.001	-1.568
ATP6V1H	Atpase H+ transporting V1 subunit H	0.000	-1.572
ATP8A1	Atpase phospholipid transporting 8A1	0.000	-2.197
ATP8B4	Atpase phospholipid transporting 8B4 (putative)	0.000	-5.893
ATPAF2	ATP synthase mitochondrial F1 complex assembly factor 2	0.003	-1.515
B3GNT2	UDP-glcnac:betagal beta-1,3-N-acetylglucosaminyltransferase 2	0.006	-1.565
B4GALNT2	Beta-1,4-N-acetyl-galactosaminyltransferase 2	0.000	-2.354
B4GALT3	Beta-1,4-galactosyltransferase 3	0.000	-1.525
B9D2	B9 protein domain 2	0.002	1.787
BAIAP2L1	BAI1 associated protein 2 like 1	0.000	-2.008

BATF2	Basic leucine zipper ATF-like transcription factor 2	0.006	-2.987
BAZ1A	Bromodomain adjacent to zinc finger domain 1A	0.001	-1.596
BCAS1	Breast carcinoma amplified sequence 1	0.003	1.756
BCAS4	Breast carcinoma amplified sequence 4	0.009	1.679
BCAT1	Branched chain amino acid transaminase 1	0.000	-2.012
BCL2L1	BCL2 like 1	0.006	-1.516
BCL7C	BCL tumor suppressor 7C	0.000	1.766
BCO2	Beta-carotene oxygenase 2	0.001	-1.889
BDH1	3-hydroxybutyrate dehydrogenase, type 1	0.000	-5.883
BDKRB2	Bradykinin receptor B2	0.000	-2.844
BFAR	Bifunctional apoptosis regulator	0.004	1.603
BICDL1	BICD family like cargo adaptor 1	0.000	-3.171
BOC	BOC cell adhesion associated, oncogene regulated	0.002	-2.504
BPI	Bactericidal/permeability-increasing protein	0.006	-4.557
BRSK1	BR serine/threonine kinase 1	0.000	-2.313
BSPRY	B-box and SPRY domain containing	0.000	-1.990
BTBD19	BTB domain containing 19	0.008	1.640
BTF3	Basic transcription factor 3	0.000	1.660
BYSL	Bystin like	0.000	-2.203
BZW2	Basic leucine zipper and W2 domains 2	0.000	-2.890
C10orf54	Chromosome 10 open reading frame 54	0.000	1.820
C11orf42	Chromosome 11 open reading frame 42	0.011	1.724
C12orf57	Chromosome 12 open reading frame 57	0.003	2.034
C14orf1	Chromosome 14 open reading frame 1	0.003	-1.913
C1orf115	Chromosome 1 open reading frame 115	0.006	2.048
C1orf122	Chromosome 1 open reading frame 122	0.003	-1.801
C1orf21	Chromosome 1 open reading frame 21	0.000	-1.890
C1orf52	Chromosome 1 open reading frame 52	0.012	-1.497
C1QL2	Complement C1q like 2	0.005	-11.263
C4orf32	Chromosome 4 open reading frame 32	0.000	-4.534
C7orf43	Chromosome 7 open reading frame 43	0.000	-1.704
C9orf43	Chromosome 9 open reading frame 43	0.000	2.557
CA1	Carbonic anhydrase 1	0.000	-219.879
CA13	Carbonic anhydrase 13	0.003	-3.069
CABIN1	Calcineurin binding protein 1	0.010	-1.573

CACNA1S	Calcium voltage-gated channel subunit alpha1 S	0.001	2.771
CALHM2	Calcium homeostasis modulator 2	0.007	1.630
CAMK2B	Calcium/calmodulin dependent protein kinase II beta	0.000	-4.004
CASP3	Caspase 3	0.001	-1.524
CASP4	Caspase 4	0.002	-2.842
CASP8	Caspase 8	0.007	-2.229
CBX7	Chromobox 7	0.009	1.597
CCDC167	Coiled-coil domain containing 167	0.007	1.691
CCDC17	Coiled-coil domain containing 17	0.007	1.582
CCDC6	Coiled-coil domain containing 6	0.000	-1.925
CCL16	C-C motif chemokine ligand 16	0.002	2.389
CCNB1	Cyclin B1	0.001	-3.573
CCNI	Cyclin I	0.001	1.750
CCR7	C-C motif chemokine receptor 7	0.002	-4.975
CD274	CD274 molecule	0.005	-4.918
CD300LG	CD300 molecule like family member g	0.010	2.171
CDADC1	Cytidine and dcmp deaminase domain containing 1	0.001	-1.997
CDC45	Cell division cycle 45	0.001	1.931
CDH1	Cadherin 1	0.008	-1.647
CDH17	Cadherin 17	0.002	-2.505
CDIP1	Cell death-inducing p53 target 1	0.000	1.850
CDKN2AIP	CDKN2A interacting protein	0.003	-1.972
CDR2	Cerebellar degeneration related protein 2	0.007	-1.516
CEP85	Centrosomal protein 85	0.003	-1.609
CERS6	Ceramide synthase 6	0.000	-2.442
CFAP20	Cilia and flagella associated protein 20	0.000	1.633
CFD	Complement factor D	0.000	1.870
CHAC2	Chac cation transport regulator homolog 2	0.002	-1.986
CHCHD6	Coiled-coil-helix-coiled-coil-helix domain containing 6	0.000	2.714
CHKA	Choline kinase alpha	0.008	-1.567
CHMP2B	Charged multivesicular body protein 2B	0.000	-1.590
CHORDC1	Cysteine and histidine rich domain containing 1	0.000	-2.372
CHTF18	Chromosome transmission fidelity factor 18	0.011	1.874
CINP	Cyclin dependent kinase 2 interacting protein	0.005	1.581
CIRBP	Cold inducible RNA binding protein	0.013	1.584

CISD2	CDGSH iron sulfur domain 2	0.001	-1.634
CITED2	Cbp/p300 interacting transactivator with Glu/Asp rich carboxy-terminal domain 2	0.004	1.496
CKS2	CDC28 protein kinase regulatory subunit 2	0.000	-2.115
CLDN11	Claudin 11	0.008	2.497
CLDN5	Claudin 5	0.000	2.374
CLEC10A	C-type lectin domain family 10 member A	0.000	-5.017
CLEC3B	C-type lectin domain family 3 member B	0.009	1.578
CLEC4F	C-type lectin domain family 4 member F	0.001	-20.859
CLIP1	CAP-Gly domain containing linker protein 1	0.003	-1.837
CMBL	Carboxymethylenebutenolidase homolog	0.000	-2.371
CMPK2	Cytidine/uridine monophosphate kinase 2	0.000	-6.343
CMTM7	CKLF like MARVEL transmembrane domain containing 7	0.000	-1.971
CNIH1	Cornichon family AMPA receptor auxiliary protein 1	0.013	-1.523
CNKSR3	CNKSR family member 3	0.000	-1.821
CNN2	Calponin 2	0.000	1.674
CNOT1	CCR4-NOT transcription complex subunit 1	0.001	-1.909
CNRIP1	Cannabinoid receptor interacting protein 1	0.003	1.731
CNTFR	Ciliary neurotrophic factor receptor	0.005	2.805
COBL	Cordon-bleu WH2 repeat protein	0.000	-3.033
COL22A1	Collagen type XXII alpha 1	0.003	-4.321
COL4A3	Collagen type IV alpha 3 chain	0.007	-2.663
COL7A1	Collagen type VII alpha 1	0.012	-1.783
COMMD8	COMM domain containing 8	0.001	-1.653
COMTD1	Catechol-O-methyltransferase domain containing 1	0.002	-1.725
COPS9	COP9 signalosome subunit 9	0.000	-2.051
COQ10A	Coenzyme Q10A	0.000	-1.813
COQ10B	Coenzyme Q10B	0.000	-1.807
COQ8A	Coenzyme Q8A	0.000	1.921
CORO2A	Coronin 2A	0.001	-1.868
COX11	COX11, cytochrome c oxidase copper chaperone	0.002	-1.581
COX5A	Cytochrome c oxidase subunit 5A	0.007	-1.579
CPAMD8	C3 and PZP like, alpha-2-macroglobulin domain containing 8	0.001	2.356
CPEB4	Cytoplasmic polyadenylation element binding protein 4	0.000	-1.799
CPM	Carboxypeptidase M	0.000	-5.465
CRCP	CGRP receptor component	0.000	-1.761

CRYBB1	Crystallin beta B1	0.001	2.674
CRYGS	Crystallin gamma S	0.007	-6.525
CRYM	Crystallin mu	0.011	-3.203
CS	Citrate synthase	0.000	-1.590
CSPG5	Chondroitin sulfate proteoglycan 5	0.011	2.110
CSRP1	Cysteine and glycine rich protein 1	0.001	1.863
CSRP2	Cysteine and glycine rich protein 2	0.003	1.809
CSTB	Cystatin B	0.001	-1.791
CTBS	Chitobiase	0.004	2.747
CWH43	Cell wall biogenesis 43 C-terminal homolog	0.000	-6.712
CX3CR1	C-X3-C motif chemokine receptor 1	0.009	2.004
CXorf38	Chromosome X open reading frame 38	0.000	-1.793
CYB5B	Cytochrome b5 type B	0.000	-3.100
CYB5R4	Cytochrome b5 reductase 4	0.002	-1.518
CYP20A1	Cytochrome P450 family 20 subfamily A member 1	0.013	1.540
CYP24A1	Cytochrome P450 family 24 subfamily A member 1	0.002	-5.068
CYR61	Cysteine rich angiogenic inducer 61	0.002	2.948
DAGLA	Diacylglycerol lipase alpha	0.008	-1.886
DAPP1	Dual adaptor of phosphotyrosine and 3-phosphoinositides 1	0.005	-1.652
DBI	Diazepam binding inhibitor, acyl-coa binding protein	0.000	-2.644
DBNDD2	Dysbindin domain containing 2	0.005	1.608
DCAF17	DDB1 and CUL4 associated factor 17	0.000	-1.843
DCK	Deoxycytidine kinase	0.002	-1.663
DDB2	Damage specific DNA binding protein 2	0.000	-1.684
DDX58	DEXD/H-box helicase 58	0.000	-7.274
DENND1B	DENN domain containing 1B	0.000	-2.084
DENND1C	DENN domain containing 1C	0.001	-1.565
DEPTOR	DEP domain containing MTOR-interacting protein	0.008	2.046
DES	Desmin	0.010	1.850
DFFA	DNA fragmentation factor subunit alpha	0.001	-1.524
DGAT2	Diacylglycerol O-acyltransferase 2	0.000	-3.820
DGKA	Diacylglycerol kinase alpha	0.000	-1.892
DGKI	Diacylglycerol kinase iota	0.000	-9.482
DHCR24	24-dehydrocholesterol reductase	0.001	-1.985
DHFR	Dihydrofolate reductase	0.000	-1.745

DHTKD1	Dehydrogenase E1 and transketolase domain containing 1	0.000	1.863
DHX36	DEAH-box helicase 36	0.000	-1.557
DHX58	DEXH-box helicase 58	0.002	-3.436
DIMT1	DIM1 dimethyladenosine transferase 1 homolog	0.000	-2.358
DIP2B	Disco interacting protein 2 homolog B	0.000	-1.589
DIRAS3	DIRAS family gtpase 3	0.010	3.218
DLAT	Dihydrolipoamide S-acetyltransferase	0.001	-1.804
DLD	Dihydrolipoamide dehydrogenase	0.001	-1.497
DLG5	Discs large MAGUK scaffold protein 5	0.000	-2.049
DMPK	Dystrophia myotonica protein kinase	0.000	-2.936
DMTN	Dematin actin binding protein	0.001	-1.920
DNAJA1	Dnaj heat shock protein family (Hsp40) member A1	0.000	-2.410
DNAJB11	Dnaj heat shock protein family (Hsp40) member B11	0.002	-1.811
DNAJB14	Dnaj heat shock protein family (Hsp40) member B14	0.008	-1.561
DNAJB9	Dnaj heat shock protein family (Hsp40) member B9	0.002	-1.660
DNAJC1	Dnaj heat shock protein family (Hsp40) member C1	0.011	-1.499
DNAJC16	Dnaj heat shock protein family (Hsp40) member C16	0.000	-1.867
DNAJC25	Dnaj heat shock protein family (Hsp40) member C25	0.000	-2.265
DOCK9	Dedicator of cytokinesis 9	0.000	-1.870
DOPEY1	Dopey family member 1	0.003	-1.500
DPP3	Dipeptidyl peptidase 3	0.000	-1.988
DPY19L4	Dpy-19 like 4 (C. Elegans)	0.000	-2.286
DRAM1	DNA damage regulated autophagy modulator 1	0.000	-2.395
DRAM2	DNA damage regulated autophagy modulator 2	0.008	-1.524
DTX3L	Deltex E3 ubiquitin ligase 3L	0.000	-3.733
DUSP26	Dual specificity phosphatase 26 (putative)	0.000	4.763
DUSP4	Dual specificity phosphatase 4	0.007	-2.184
DYNC1I1	Dynein cytoplasmic 1 intermediate chain 1	0.006	-1.749
DYNC2LI1	Dynein cytoplasmic 2 light intermediate chain 1	0.003	1.602
DYNLRB2	Dynein light chain roadblock-type 2	0.010	1.884
EBAG9	Estrogen receptor binding site associated, antigen, 9	0.000	-1.748
ECSCR	Endothelial cell surface expressed chemotaxis and apoptosis regulator	0.003	1.815
EEF1B2	Eukaryotic translation elongation factor 1 beta 2	0.004	1.846
EEF1D	Eukaryotic translation elongation factor 1 delta	0.001	1.772
EEF1E1	Eukaryotic translation elongation factor 1 epsilon 1	0.012	-1.567

EFEMP1	EGF containing fibulin like extracellular matrix protein 1	0.003	1.855
EHD2	EH domain containing 2	0.013	1.544
EHD4	EH domain containing 4	0.006	-1.957
EIF2AK2	Eukaryotic translation initiation factor 2 alpha kinase 2	0.000	-4.387
EIF4E	Eukaryotic translation initiation factor 4E	0.000	-1.687
EIF4G3	Eukaryotic translation initiation factor 4 gamma 3	0.002	-1.737
ELOVL5	ELOVL fatty acid elongase 5	0.000	-2.002
ELOVL6	ELOVL fatty acid elongase 6	0.000	-2.108
EMB	Embigin	0.009	-2.416
EML2	Echinoderm microtubule associated protein like 2	0.004	-1.526
EMP1	Epithelial membrane protein 1	0.012	-2.112
ENDOG	Endonuclease G	0.003	-1.568
ENPP1	Ectonucleotide pyrophosphatase/phosphodiesterase 1	0.000	-3.111
ENPP3	Ectonucleotide pyrophosphatase/phosphodiesterase 3	0.000	-7.692
ENTPD2	Ectonucleoside triphosphate diphosphohydrolase 2	0.000	-3.268
EPB41L4A	Erythrocyte membrane protein band 4.1 like 4A	0.012	-1.496
EPHA1	EPH receptor A1	0.007	-1.726
EPN3	Epsin 3	0.000	-5.310
EPS8L1	EPS8 like 1	0.001	-1.727
EPSTI1	Epithelial stromal interaction 1 (breast)	0.001	-4.670
ERBB2	Erb-b2 receptor tyrosine kinase 2	0.003	1.625
ERBB3	Erb-b2 receptor tyrosine kinase 3	0.004	1.543
ERGIC2	ERGIC and golgi 2	0.001	-1.599
ERN1	Endoplasmic reticulum to nucleus signaling 1	0.004	-1.856
ERO1A	Endoplasmic reticulum oxidoreductase 1 alpha	0.000	-3.101
ESAM	Endothelial cell adhesion molecule	0.008	1.693
ESF1	ESF1 nucleolar pre-rrna processing protein homolog	0.000	-1.905
Esrra	Estrogen related receptor, alpha	0.001	-1.580
ETV5	ETS variant 5	0.003	1.747
EXOC6	Exocyst complex component 6	0.000	-1.612
EZH1	Enhancer of zeste 1 polycomb repressive complex 2 subunit	0.002	1.514
EZR	Ezrin	0.001	-1.655
F3	Coagulation factor III, tissue factor	0.004	-1.767
F5	Coagulation factor V	0.010	2.200
FABP3	Fatty acid binding protein 3	0.000	-6.594

FAHD2A	Fumarylacetoacetate hydrolase domain containing 2A	0.000	2.113
FAM110D	Family with sequence similarity 110 member D	0.010	1.705
FAM111B	Family with sequence similarity 111 member B	0.006	-1.755
FAM162A	Family with sequence similarity 162 member A	0.000	-2.828
FAM171A2	Family with sequence similarity 171 member A2	0.004	1.961
FAM189A2	Family with sequence similarity 189 member A2	0.005	-1.694
FAM210B	Family with sequence similarity 210 member B	0.000	1.975
FAM214B	Family with sequence similarity 214 member B	0.000	-1.619
FAM229B	Family with sequence similarity 229 member B	0.002	2.485
FAM3B	Family with sequence similarity 3 member B	0.002	-2.576
FAM3C	Family with sequence similarity 3 member C	0.000	-1.925
FAM43A	Family with sequence similarity 43 member A	0.001	-2.383
FAM63B	Family with sequence similarity 63 member B	0.001	-1.666
FAM69A	Family with sequence similarity 69 member A	0.000	-2.355
FAM84A	Family with sequence similarity 84 member A	0.000	-2.442
FASN	Fatty acid synthase	0.000	-1.962
FBLN5	Fibulin 5	0.000	1.835
FBP1	Fructose-bisphosphatase 1	0.006	-3.475
FBXL21	F-box and leucine rich repeat protein 21 (gene/pseudogene)	0.011	1.916
FBXL4	F-box and leucine rich repeat protein 4	0.006	1.512
FBXO32	F-box protein 32	0.006	1.686
FBXO33	F-box protein 33	0.003	-1.715
FDFT1	Farnesyl-diphosphate farnesyltransferase 1	0.002	-1.538
FERMT1	Fermitin family member 1	0.002	-1.652
FGFBP1	Fibroblast growth factor binding protein 1	0.000	-10.656
FGL1	Fibrinogen like 1	0.000	2.927
FHL1	Four and a half LIM domains 1	0.000	1.741
FIS1	Fission, mitochondrial 1	0.001	1.612
FKBP5	FK506 binding protein 5	0.000	-2.030
FKBP9	FK506 binding protein 9	0.005	-1.566
FLNC	Filamin C	0.005	1.696
FLVCR2	Feline leukemia virus subgroup C cellular receptor family member 2	0.009	-1.629
FMR1	Fragile X mental retardation 1	0.004	-1.638
FNDC4	Fibronectin type III domain containing 4	0.001	2.360
FOSL1	FOS like 1, AP-1 transcription factor subunit	0.010	-3.495

FOXN4	Forkhead box N4	0.000	-1.512
FOXO4	Forkhead box O4	0.000	1.931
Foxp1	Forkhead box P1	0.006	1.594
FOXRED2	FAD dependent oxidoreductase domain containing 2	0.000	-11.624
FRK	Fyn related Src family tyrosine kinase	0.001	-1.950
FST	Follistatin	0.002	-2.739
FUT1	Fucosyltransferase 1 (H blood group)	0.002	-4.001
FUT6	Fucosyltransferase 6	0.006	-2.370
FUZ	Fuzzy planar cell polarity protein	0.000	1.915
G6PD	Glucose-6-phosphate dehydrogenase	0.001	-2.000
GAB2	GRB2 associated binding protein 2	0.004	-1.645
GALE	UDP-galactose-4-epimerase	0.001	-2.117
GALNT15	Polypeptide N-acetylgalactosaminyltransferase 15	0.003	2.770
GALNT6	Polypeptide N-acetylgalactosaminyltransferase 6	0.008	1.872
GATA2	GATA binding protein 2	0.001	-1.891
GBGT1	Globoside alpha-1,3-N-acetylgalactosaminyltransferase 1	0.000	-2.924
GBP1	Guanylate binding protein 1	0.001	-4.828
GBP2	Guanylate binding protein 2	0.000	-9.067
GBP5	Guanylate binding protein 5	0.003	-4.830
GCA	Grancalcin	0.006	-2.095
GCK	Glucokinase	0.000	1.940
GCLC	Glutamate-cysteine ligase catalytic subunit	0.000	-3.116
GDA	Guanine deaminase	0.000	-3.894
GDAP1	Ganglioside induced differentiation associated protein 1	0.006	-1.599
GDAP2	Ganglioside induced differentiation associated protein 2	0.011	-1.695
GDE1	Glycerophosphodiester phosphodiesterase 1	0.000	-1.639
GDPD1	Glycerophosphodiester phosphodiesterase domain containing 1	0.000	-3.508
GDPGP1	GDP-D-glucose phosphorylase 1	0.002	-1.943
GIPC2	GIPC PDZ domain containing family member 2	0.002	-1.743
GJB1	Gap junction protein beta 1	0.000	-3.732
GJB3	Gap junction protein beta 3	0.000	-3.201
GJB5	Gap junction protein beta 5	0.000	-5.483
GK5	Glycerol kinase 5 (putative)	0.000	-5.618
GLRX	Glutaredoxin	0.002	-1.830
GLTSCR2	Glioma tumor suppressor candidate region gene 2	0.001	1.558

GLUD1	Glutamate dehydrogenase 1	0.000	-1.579
GM2A	GM2 ganglioside activator	0.000	-3.145
GMFB	Glia maturation factor beta	0.000	-2.008
GMNN	Geminin, DNA replication inhibitor	0.000	1.829
GNG7	G protein subunit gamma 7	0.002	1.583
GNMT	Glycine N-methyltransferase	0.000	-2.388
GNPNAT1	Glucosamine-phosphate N-acetyltransferase 1	0.003	-1.590
GOT1	Glutamic-oxaloacetic transaminase 1	0.000	-2.273
GPHN	Gephyrin	0.001	-1.723
GPLD1	Glycosylphosphatidylinositol specific phospholipase D1	0.001	-5.948
GPRC5A	G protein-coupled receptor class C group 5 member A	0.001	-3.710
GPX2	Glutathione peroxidase 2	0.000	-3.315
GRASP	General receptor for phosphoinositides 1 associated scaffold protein	0.010	1.595
GRB7	Growth factor receptor bound protein 7	0.001	1.860
GRHL2	Grainyhead like transcription factor 2	0.010	-1.626
GRIN1	Glutamate ionotropic receptor NMDA type subunit 1	0.000	-3.086
GRIP2	Glutamate receptor interacting protein 2	0.003	-4.421
GSAP	Gamma-secretase activating protein	0.006	1.681
GSPT1	G1 to S phase transition 1	0.005	-1.501
GSR	Glutathione-disulfide reductase	0.003	-1.622
GSS	Glutathione synthetase	0.001	-2.337
Gstt3	Glutathione S-transferase, theta 3	0.000	-5.064
GSTZ1	Glutathione S-transferase zeta 1	0.010	1.593
GUSB	Glucuronidase beta	0.000	-1.736
H2-T24	Histocompatibility 2, T region locus 24	0.005	-2.668
HACD2	3-hydroxyacyl-coa dehydratase 2	0.000	-1.586
HDAC11	Histone deacetylase 11	0.007	1.569
HDAC5	Histone deacetylase 5	0.000	1.565
HEATR5B	HEAT repeat containing 5B	0.000	-1.746
HEBP1	Heme binding protein 1	0.004	1.804
HENMT1	HEN1 methyltransferase homolog 1	0.006	-2.306
HERC3	HECT and RLD domain containing E3 ubiquitin protein ligase 3	0.000	-2.213
HERC5	HECT and RLD domain containing E3 ubiquitin protein ligase 5	0.000	-3.923
HERC6	HECT and RLD domain containing E3 ubiquitin protein ligase family member 6	0.000	-5.850
HIF1A	Hypoxia inducible factor 1 alpha subunit	0.013	-1.745

HIGD1A	HIG1 hypoxia inducible domain family member 1A	0.004	-2.774
HIST1H4J	Histone cluster 1, h4j	0.001	2.500
HK2	Hexokinase 2	0.000	-5.895
HMGB2	High mobility group box 2	0.001	1.639
HMGB3	High mobility group box 3	0.000	2.069
HMGCR	3-hydroxy-3-methylglutaryl-coa reductase	0.000	-2.015
HOMER1	Homer scaffolding protein 1	0.000	-2.124
HSD17B11	Hydroxysteroid 17-beta dehydrogenase 11	0.009	1.510
HSD17B12	Hydroxysteroid 17-beta dehydrogenase 12	0.000	-3.137
HSD17B4	Hydroxysteroid 17-beta dehydrogenase 4	0.001	-1.565
HSDL2	Hydroxysteroid dehydrogenase like 2	0.004	1.600
HSPA6	Heat shock protein family A (Hsp70) member 6	0.001	4.237
IDE	Insulin degrading enzyme	0.001	-1.693
IDH2	Isocitrate dehydrogenase (NADP(+)) 2, mitochondrial	0.000	-1.764
IDI1	Isopentenyl-diphosphate delta isomerase 1	0.005	-1.753
IDO1	Indoleamine 2,3-dioxygenase 1	0.000	-14.434
IFI44	Interferon induced protein 44	0.002	-5.558
IFI44L	Interferon induced protein 44 like	0.000	-8.004
IFI6	Interferon alpha inducible protein 6	0.004	-4.338
IFIH1	Interferon induced with helicase C domain 1	0.001	-4.160
IFIT1	Interferon induced protein with tetratricopeptide repeats 1	0.000	-14.697
IFIT2	Interferon induced protein with tetratricopeptide repeats 2	0.000	-21.958
IFIT3	Interferon induced protein with tetratricopeptide repeats 3	0.000	-12.683
IFIT5	Interferon induced protein with tetratricopeptide repeats 5	0.000	-3.492
IFITM1	Interferon induced transmembrane protein 1	0.011	-2.985
IFT27	Intraflagellar transport 27	0.001	1.814
IGF2	Insulin like growth factor 2	0.008	1.884
IGSF8	Immunoglobulin superfamily member 8	0.006	1.606
IHH	Indian hedgehog	0.005	-2.237
IKBKAP	Inhibitor of kappa light polypeptide gene enhancer in B-cells, kinase complex-associated protein	0.000	-1.865
IL1RAP	Interleukin 1 receptor accessory protein	0.008	-1.574
IL1RL1	Interleukin 1 receptor like 1	0.003	-5.769
ILF2	Interleukin enhancer binding factor 2	0.009	-1.522
ILVBL	Ilvb acetolactate synthase like	0.000	-2.198
IMPAD1	Inositol monophosphatase domain containing 1	0.000	-1.754

IMPDH2	Inosine monophosphate dehydrogenase 2	0.002	1.580
INHBB	Inhibin beta B subunit	0.011	2.042
INPP5K	Inositol polyphosphate-5-phosphatase K	0.002	1.551
INSIG1	Insulin induced gene 1	0.006	-1.689
IRAK1BP1	Interleukin 1 receptor associated kinase 1 binding protein 1	0.001	1.859
IRF6	Interferon regulatory factor 6	0.006	-1.825
IRF7	Interferon regulatory factor 7	0.001	-3.793
IRF9	Interferon regulatory factor 9	0.001	-3.449
ISG15	ISG15 ubiquitin-like modifier	0.000	-15.603
ISG20	Interferon stimulated exonuclease gene 20	0.000	-31.903
ISLR2	Immunoglobulin superfamily containing leucine rich repeat 2	0.002	4.549
IST1	IST1, ESCRT-III associated factor	0.003	-1.517
ITGA2	Integrin subunit alpha 2	0.001	-1.937
ITGA6	Integrin subunit alpha 6	0.001	-1.632
ITPR3	Inositol 1,4,5-trisphosphate receptor type 3	0.004	-2.015
ITPRIPL1	Inositol 1,4,5-trisphosphate receptor interacting protein-like 1	0.012	-1.636
JAK3	Janus kinase 3	0.000	-5.583
JARID2	Jumonji and AT-rich interaction domain containing 2	0.000	-5.632
JMJD6	Arginine demethylase and lysine hydroxylase	0.005	-1.537
JPH1	Junctophilin 1	0.000	-2.509
JTB	Jumping translocation breakpoint	0.003	1.532
KANK1	KN motif and ankyrin repeat domains 1	0.000	-1.820
KCNAB3	Potassium voltage-gated channel subfamily A regulatory beta subunit 3	0.006	2.302
KCNK1	Potassium two pore domain channel subfamily K member 1	0.000	-2.224
KCNMB2	Potassium calcium-activated channel subfamily M regulatory beta subunit 2	0.000	3.002
KDF1	Keratinocyte differentiation factor 1	0.000	-2.109
KIAA0226L	KIAA0226 like	0.003	-2.483
KIAA1147	Kiaa1147	0.011	1.583
KIAA1551	Kiaa1551	0.005	-1.730
KIF17	Kinesin family member 17	0.000	2.624
KIF21A	Kinesin family member 21A	0.000	-2.153
KIF2A	Kinesin family member 2A	0.000	-1.727
KIF5B	Kinesin family member 5B	0.006	-1.507
KIF5C	Kinesin family member 5C	0.003	-3.915
KIT	KIT proto-oncogene receptor tyrosine kinase	0.005	2.260

KLC2	Kinesin light chain 2	0.000	-1.770
KLC3	Kinesin light chain 3	0.009	2.421
KLHDC8B	Kelch domain containing 8B	0.011	1.526
KLHL2	Kelch like family member 2	0.000	-1.531
KLHL22	Kelch like family member 22	0.003	1.550
KLHL42	Kelch like family member 42	0.000	-2.178
KNSTRN	Kinetochore localized astrin/SPAG5 binding protein	0.000	1.739
KPNA2	Karyopherin subunit alpha 2	0.000	-2.214
KRT17	Keratin 17	0.000	-38.995
KRT24	Keratin 24	0.000	-10.820
LACE1	Lactation elevated 1	0.003	-1.578
LARP1B	La ribonucleoprotein domain family member 1B	0.002	-1.901
LARP4	La ribonucleoprotein domain family member 4	0.000	-1.934
LARP6	La ribonucleoprotein domain family member 6	0.006	1.552
LDHA	Lactate dehydrogenase A	0.001	-2.276
LDLR	Low density lipoprotein receptor	0.000	-2.166
LDLRAD4	Low density lipoprotein receptor class A domain containing 4	0.013	-1.682
LEO1	LEO1 homolog, Paf1/RNA polymerase II complex component	0.000	-1.576
LETM1	Leucine zipper and EF-hand containing transmembrane protein 1	0.000	-1.663
LIG1	DNA ligase 1	0.010	1.508
LIMK2	LIM domain kinase 2	0.000	-2.053
LIMS2	LIM zinc finger domain containing 2	0.006	1.619
LIN7C	Lin-7 homolog C, crumbs cell polarity complex component	0.001	-1.543
LLGL1	LLGL1, scribble cell polarity complex component	0.002	1.504
LMCD1	LIM and cysteine rich domains 1	0.000	2.331
LNX1	Ligand of numb-protein X 1	0.003	-1.852
LPAR6	Lysophosphatidic acid receptor 6	0.001	2.151
LPL	Lipoprotein lipase	0.000	-7.519
LRAT	Lecithin retinol acyltransferase (phosphatidylcholine--retinol O-acyltransferase)	0.001	-3.768
LRCH1	Leucine rich repeats and calponin homology domain containing 1	0.000	-1.868
LRFN3	Leucine rich repeat and fibronectin type III domain containing 3	0.006	1.640
LRP12	LDL receptor related protein 12	0.001	-1.608
LRP8	LDL receptor related protein 8	0.000	-3.206
LRRC38	Leucine rich repeat containing 38	0.000	2.283
LSS	Lanosterol synthase (2,3-oxidosqualene-lanosterol cyclase)	0.000	-1.738

LTBP3	Latent transforming growth factor beta binding protein 3	0.000	1.729
LTF	Lactotransferrin	0.000	-7.342
LTV1	LTV1 ribosome biogenesis factor	0.011	-1.526
LY6E	Lymphocyte antigen 6 complex, locus E	0.000	-3.645
LY6G6C	Lymphocyte antigen 6 complex, locus G6C	0.012	-2.578
LYPD6	LY6/PLAUR domain containing 6	0.001	-2.550
LYPLA1	Lysophospholipase I	0.003	-1.506
LZTS1	Leucine zipper tumor suppressor 1	0.011	-1.975
MACROD1	MACRO domain containing 1	0.002	1.814
MAGED2	MAGE family member D2	0.000	2.065
MALL	Mal, T-cell differentiation protein like	0.000	2.085
MAP1LC3A	Microtubule associated protein 1 light chain 3 alpha	0.001	-1.801
MAP3K8	Mitogen-activated protein kinase kinase kinase 8	0.009	-2.205
MAPK6	Mitogen-activated protein kinase 6	0.000	-1.802
MARK1	Microtubule affinity regulating kinase 1	0.000	-1.829
MARK3	Microtubule affinity regulating kinase 3	0.000	-1.603
MARVELD1	MARVEL domain containing 1	0.002	1.500
MATN4	Matrilin 4	0.004	-3.200
MB21D1	Mab-21 domain containing 1	0.000	-3.645
MBOAT2	Membrane bound O-acyltransferase domain containing 2	0.000	-2.543
MBP	Myelin basic protein	0.006	1.659
MCCC2	Methylcrotonoyl-coa carboxylase 2	0.000	-3.740
MCEE	Methylmalonyl-coa epimerase	0.006	-1.613
MCM7	Minichromosome maintenance complex component 7	0.000	1.703
MCOLN2	Mucolipin 2	0.007	1.871
MCRIP2	MAPK regulated corepressor interacting protein 2	0.005	-1.857
MCUR1	Mitochondrial calcium uniporter regulator 1	0.006	-1.645
MESDC1	Mesoderm development candidate 1	0.000	-1.771
METAP1	Methionyl aminopeptidase 1	0.001	-1.646
METTL13	Methyltransferase like 13	0.000	-2.177
METTL5	Methyltransferase like 5	0.005	1.595
MFGE8	Milk fat globule-EGF factor 8 protein	0.001	1.502
MGAT4A	Mannosyl (alpha-1,3-)-glycoprotein beta-1,4-N-acetylglucosaminyltransferase, isozyme A	0.003	-3.064
MIA3	MIA family member 3, ER export factor	0.000	-1.599
MICAL2	Microtubule associated monooxygenase, calponin and LIM domain containing 2	0.000	-3.011

MICB	MHC class I polypeptide-related sequence B	0.008	-3.319
MKS1	Meckel syndrome, type 1	0.001	1.676
MMP15	Matrix metallopeptidase 15	0.001	2.470
MOB1A	MOB kinase activator 1A	0.002	-1.586
MOCOS	Molybdenum cofactor sulfurase	0.000	-2.287
MORC3	MORC family CW-type zinc finger 3	0.002	-1.949
MORN1	MORN repeat containing 1	0.011	1.769
MOXD1	Monooxygenase DBH like 1	0.003	-2.595
MPG	N-methylpurine DNA glycosylase	0.006	1.545
MPP7	Membrane palmitoylated protein 7	0.000	-4.497
MPV17L2	MPV17 mitochondrial inner membrane protein like 2	0.000	-2.691
MPZ	Myelin protein zero	0.012	2.362
MRPL52	Mitochondrial ribosomal protein L52	0.000	1.620
MRPS25	Mitochondrial ribosomal protein S25	0.001	-1.665
MRPS36	Mitochondrial ribosomal protein S36	0.000	-11.446
MRS2	MRS2, magnesium transporter	0.000	-1.850
MSMO1	Methylsterol monooxygenase 1	0.009	-1.671
MSRB2	Methionine sulfoxide reductase B2	0.000	1.714
MSX1	Msh homeobox 1	0.000	-2.287
MSX2	Msh homeobox 2	0.000	-2.556
MTCL1	Microtubule crosslinking factor 1	0.000	-2.866
MTHFD1L	Methylenetetrahydrofolate dehydrogenase (NADP+ dependent) 1-like	0.001	-2.396
MTHFD2	Methylenetetrahydrofolate dehydrogenase (NADP+ dependent) 2, methenyltetrahydrofolate cyclohydrolase	0.000	-2.312
MTHFD2L	Methylenetetrahydrofolate dehydrogenase (NADP+ dependent) 2-like	0.002	-2.237
MTMR12	Myotubularin related protein 12	0.002	-1.717
MTR	5-methyltetrahydrofolate-homocysteine methyltransferase	0.011	-1.608
MTUS1	Microtubule associated tumor suppressor 1	0.008	-1.777
MUM1L1	MUM1 like 1	0.010	-1.867
MUSTN1	Musculoskeletal, embryonic nuclear protein 1	0.004	1.596
MVD	Mevalonate diphosphate decarboxylase	0.001	-1.637
MX1	MX dynamin like gtpase 1	0.000	-7.296
MX2	MX dynamin like gtpase 2	0.000	-17.761
MYCT1	Myc target 1	0.001	2.667
MYH14	Myosin, heavy chain 14, non-muscle	0.012	-1.764

MYO10	Myosin X	0.011	-1.544
MYO1B	Myosin IB	0.000	-1.986
MYO5B	Myosin VB	0.006	-1.715
MYO6	Myosin VI	0.000	-2.184
MYOF	Myoferlin	0.001	-1.757
MYRIP	Myosin VIIA and Rab interacting protein	0.013	1.858
NAA40	N(alpha)-acetyltransferase 40, natd catalytic subunit	0.009	-1.505
NAALAD2	N-acetylated alpha-linked acidic dipeptidase 2	0.000	-3.913
NAMPT	Nicotinamide phosphoribosyltransferase	0.000	-2.320
NAP1L1	Nucleosome assembly protein 1 like 1	0.009	1.534
NDFIP2	Nedd4 family interacting protein 2	0.000	-3.006
NDP	NDP, norrin cystine knot growth factor	0.012	1.512
NDRG1	N-myc downstream regulated 1	0.000	1.858
NDRG2	NDRG family member 2	0.006	1.762
NDUFA5	NADH:ubiquinone oxidoreductase subunit A5	0.003	-1.655
NDUFAF5	NADH:ubiquinone oxidoreductase complex assembly factor 5	0.003	-2.167
NEBL	Nebulette	0.000	-2.387
NECTIN1	Nectin cell adhesion molecule 1	0.012	-1.687
NEDD4L	Neural precursor cell expressed, developmentally down-regulated 4-like, E3 ubiquitin protein ligase	0.001	-2.312
NETO2	Neuropilin and tolloid like 2	0.000	-4.429
NEURL1B	Neuralized E3 ubiquitin protein ligase 1B	0.005	2.339
NFKBIL1	NFKB inhibitor like 1	0.003	1.539
NINJ1	Ninjurin 1	0.004	1.588
NIP7	NIP7, nucleolar pre-rrna processing protein	0.005	-1.664
NKD2	Naked cuticle homolog 2	0.001	1.875
NLK	Nemo like kinase	0.000	-1.832
NLRX1	NLR family member X1	0.002	1.554
NME3	NME/NM23 nucleoside diphosphate kinase 3	0.002	1.824
NOA1	Nitric oxide associated 1	0.000	1.707
NOCT	Nocturnin	0.011	-1.926
NOSIP	Nitric oxide synthase interacting protein	0.000	1.563
NPL	N-acetylneuraminate pyruvate lyase	0.009	1.891
NPM1	Nucleophosmin	0.011	1.531
NPPC	Natriuretic peptide C	0.009	-5.206
Nradd	Neurotrophin receptor associated death domain	0.005	1.628

NSDHL	NAD(P) dependent steroid dehydrogenase-like	0.000	-2.134
NSG1	Neuron specific gene family member 1	0.002	-1.832
NSMCE1	NSE1 homolog, SMC5-SMC6 complex component	0.001	1.839
NTHL1	Nth-like DNA glycosylase 1	0.000	1.734
NUDCD3	Nudc domain containing 3	0.000	-1.680
NUDT1	Nudix hydrolase 1	0.002	1.730
NUP160	Nucleoporin 160	0.002	-1.527
NUP205	Nucleoporin 205	0.000	-1.648
NUP210L	Nucleoporin 210 like	0.002	1.614
NUP88	Nucleoporin 88	0.001	-1.575
NUS1	NUS1 dehydrodolichyl diphosphate synthase subunit	0.001	-1.642
NXNL2	Nucleoredoxin-like 2	0.000	-6.312
NXPE2	Neurexophilin and PC-esterase domain family member 2	0.001	4.334
NXT2	Nuclear transport factor 2 like export factor 2	0.000	-1.953
OAS1	2'-5'-oligoadenylate synthetase 1	0.000	-5.335
OAS2	2'-5'-oligoadenylate synthetase 2	0.000	-22.734
ODC1	Ornithine decarboxylase 1	0.000	-4.475
OGDH	Oxoglutarate dehydrogenase	0.000	-4.623
OGG1	8-oxoguanine DNA glycosylase	0.002	1.607
OSBPL10	Oxysterol binding protein like 10	0.000	-1.605
OSTM1	Osteopetrosis associated transmembrane protein 1	0.000	-2.535
OVOL1	Ovo like transcriptional repressor 1	0.002	-2.130
OXCT1	3-oxoacid coa-transferase 1	0.000	-1.806
OXSR1	Oxidative stress responsive 1	0.001	-1.498
P2RX3	Purinergic receptor P2X 3	0.000	-3.608
P2RY2	Purinergic receptor P2Y2	0.000	-1.924
PABPC1	Poly(A) binding protein cytoplasmic 1	0.000	1.788
PACSIN1	Protein kinase C and casein kinase substrate in neurons 1	0.000	-4.402
PAFAH2	Platelet activating factor acetylhydrolase 2	0.006	-1.863
PALM	Paralemmin	0.004	1.539
PANK1	Pantothenate kinase 1	0.002	-1.958
PANK3	Pantothenate kinase 3	0.001	-2.084
PAQR5	Progestin and adipoq receptor family member 5	0.001	2.329
PAQR8	Progestin and adipoq receptor family member 8	0.005	-1.735
PARP12	Poly(ADP-ribose) polymerase family member 12	0.004	-2.766

PARP14	Poly(ADP-ribose) polymerase family member 14	0.000	-4.604
PARP9	Poly(ADP-ribose) polymerase family member 9	0.002	-2.638
PATL1	PAT1 homolog 1, processing body mrna decay factor	0.000	-2.372
PAX2	Paired box 2	0.006	-2.012
PC	Pyruvate carboxylase	0.004	-1.729
PCCA	Propionyl-coa carboxylase alpha subunit	0.000	-1.760
PCED1A	PC-esterase domain containing 1A	0.000	1.739
PCK2	Phosphoenolpyruvate carboxykinase 2, mitochondrial	0.004	-2.168
PCSK7	Proprotein convertase subtilisin/kexin type 7	0.002	-1.520
PDCD4	Programmed cell death 4 (neoplastic transformation inhibitor)	0.000	-1.749
PDE12	Phosphodiesterase 12	0.000	-2.778
PDGFB	Platelet derived growth factor subunit B	0.001	1.684
PDHX	Pyruvate dehydrogenase complex component X	0.000	-1.619
PDK2	Pyruvate dehydrogenase kinase 2	0.000	1.814
PDSS1	Prenyl (decaprenyl) diphosphate synthase, subunit 1	0.008	-1.861
PDXK	Pyridoxal (pyridoxine, vitamin B6) kinase	0.000	-3.246
PDZD8	PDZ domain containing 8	0.000	-1.779
PDZK1	PDZ domain containing 1	0.000	-6.248
PDZK1IP1	PDZK1 interacting protein 1	0.000	-8.014
PELI1	Pellino E3 ubiquitin protein ligase 1	0.000	-1.680
PEPD	Peptidase D	0.000	-3.337
PEX1	Peroxisomal biogenesis factor 1	0.000	-1.913
PFDN5	Prefoldin subunit 5	0.000	1.991
PFKFB2	6-phosphofructo-2-kinase/fructose-2,6-biphosphatase 2	0.000	-4.724
PGP	Phosphoglycolate phosphatase	0.000	-2.157
PHF1	PHD finger protein 1	0.000	1.635
PHF10	PHD finger protein 10	0.002	1.647
PHGDH	Phosphoglycerate dehydrogenase	0.000	-4.463
PIGF	Phosphatidylinositol glycan anchor biosynthesis class F	0.005	-1.559
PIGR	Polymeric immunoglobulin receptor	0.004	3.287
PIK3AP1	Phosphoinositide-3-kinase adaptor protein 1	0.000	-2.336
PIK3CB	Phosphatidylinositol-4,5-bisphosphate 3-kinase catalytic subunit beta	0.000	-1.800
PIK3IP1	Phosphoinositide-3-kinase interacting protein 1	0.001	1.885
PITPNB	Phosphatidylinositol transfer protein beta	0.000	-1.498
PKIA	Protein kinase (camp-dependent, catalytic) inhibitor alpha	0.001	-2.197

PLA2G10	Phospholipase A2 group X	0.009	2.059
PLA2G3	Phospholipase A2 group III	0.000	-34.600
PLEKHA4	Pleckstrin homology domain containing A4	0.006	-2.799
PLEKHG1	Pleckstrin homology and rhogef domain containing G1	0.000	-3.911
PLET1	Placenta expressed transcript 1	0.000	-42.211
PLIN2	Perilipin 2	0.000	-2.161
PLK5	Polo like kinase 5	0.000	-17.735
PLLP	Plasmolipin	0.000	-2.942
PLPP3	Phospholipid phosphatase 3	0.002	1.573
PLS1	Plastin 1	0.000	-3.135
PLXDC1	Plexin domain containing 1	0.013	-1.614
PMVK	Phosphomevalonate kinase	0.004	-1.921
PNMAL1	Paraneoplastic Ma antigen family like 1	0.001	2.449
PNPT1	Polyribonucleotide nucleotidyltransferase 1	0.000	-3.931
POLD1	DAN polymerase delta 1, catalytic subunit	0.001	1.642
POLH	DNA polymerase eta	0.009	-1.517
POLK	Polymerase (DNA) kappa	0.009	-1.877
POLR3A	RNA polymerase III subunit A	0.010	-1.504
POLR3E	RNA polymerase III subunit E	0.000	-1.529
POP1	POP1 homolog, ribonuclease P/MRP subunit	0.004	-1.850
PPA1	Pyrophosphatase (inorganic) 1	0.001	-3.003
PPARA	Peroxisome proliferator activated receptor alpha	0.002	-1.600
PPARD	Peroxisome proliferator activated receptor delta	0.002	-1.608
PPARGC1B	PPARG coactivator 1 beta	0.000	-4.231
PPDPF	Pancreatic progenitor cell differentiation and proliferation factor	0.003	1.522
PPM1H	Protein phosphatase, Mg2+/Mn2+ dependent 1H	0.000	-1.984
PPM1M	Protein phosphatase, Mg2+/Mn2+ dependent 1M	0.000	-1.762
PPP2R3A	Protein phosphatase 2 regulatory subunit b"alpha	0.000	-1.754
PREB	Prolactin regulatory element binding	0.007	-1.687
PRKAB2	Protein kinase AMP-activated non-catalytic subunit beta 2	0.002	-1.658
PRKAG2	Protein kinase AMP-activated non-catalytic subunit gamma 2	0.000	-1.764
PRKCDBP	Protein kinase C delta binding protein	0.008	1.668
PRKCE	Protein kinase C epsilon	0.008	1.500
PRKCQ	Protein kinase C theta	0.000	-4.126
PROCA1	Protein interacting with cyclin A1	0.000	1.911

PROCR	Protein C receptor	0.002	-1.796
PRPSAP1	Phosphoribosyl pyrophosphate synthetase associated protein 1	0.000	-1.744
PRRG2	Proline rich and Gla domain 2	0.001	-1.699
PRSS23	Protease, serine 23	0.010	-1.926
PRSS35	Protease, serine 35	0.002	-3.682
PSAT1	Phosphoserine aminotransferase 1	0.000	-12.421
PSMC6	Proteasome 26S subunit, atpase 6	0.000	-1.598
PSME4	Proteasome activator subunit 4	0.000	-1.746
PSMG4	Proteasome assembly chaperone 4	0.000	2.063
PSRC1	Proline and serine rich coiled-coil 1	0.001	1.690
PTCH2	Patched 2	0.002	-2.458
PTGER4	Prostaglandin E receptor 4	0.002	-2.077
PTGES2	Prostaglandin E synthase 2	0.011	-1.559
PTOV1	Prostate tumor overexpressed 1	0.000	1.549
PTPN1	Protein tyrosine phosphatase, non-receptor type 1	0.000	-2.135
PTPRE	Protein tyrosine phosphatase, receptor type E	0.002	-2.956
PYROXD2	Pyridine nucleotide-disulphide oxidoreductase domain 2	0.002	2.086
RAB33B	RAB33B, member RAS oncogene family	0.001	-1.506
RAB8B	RAB8B, member RAS oncogene family	0.000	-2.313
RABGEF1	RAB guanine nucleotide exchange factor 1	0.000	-1.670
RACK1	Receptor for activated C kinase 1	0.011	1.604
RANBP17	RAN binding protein 17	0.008	-1.787
RAP1GAP2	RAP1 gtpase activating protein 2	0.007	-1.890
RASA4	RAS p21 protein activator 4	0.005	1.793
RASD1	Ras related dexamethasone induced 1	0.004	1.820
RASEF	RAS and EF-hand domain containing	0.000	-4.111
RASL11A	RAS like family 11 member A	0.000	2.053
RBP4	Retinol binding protein 4	0.012	1.664
RBPMS	RNA binding protein with multiple splicing	0.009	1.521
RCN1	Reticulocalbin 1	0.000	-2.727
RDH11	Retinol dehydrogenase 11 (all-trans/9-cis/11-cis)	0.006	-1.713
RDH16	Retinol dehydrogenase 16 (all-trans)	0.000	4.536
REEP5	Receptor accessory protein 5	0.007	-1.885
RELL2	RELT like 2	0.001	1.754
RFC4	Replication factor C subunit 4	0.003	1.653

RFFL	Ring finger and FYVE-like domain containing E3 ubiquitin protein ligase	0.000	-2.011
RFNG	RFNG O-fucosylpeptide 3-beta-N-acetylglucosaminyltransferase	0.001	1.529
RFX5	Regulatory factor X5	0.002	-1.648
RGS14	Regulator of G-protein signaling 14	0.000	-2.745
RHBG	Rh family B glycoprotein (gene/pseudogene)	0.002	-3.673
RHCG	Rh family C glycoprotein	0.005	5.086
RHOBTB3	Rho related BTB domain containing 3	0.000	-2.409
RIDA	Reactive intermediate imine deaminase A homolog	0.000	-2.214
RITA1	RBPJ interacting and tubulin associated 1	0.012	-1.517
RNF122	Ring finger protein 122	0.000	-3.540
RNF128	Ring finger protein 128, E3 ubiquitin protein ligase	0.002	-1.783
RNF180	Ring finger protein 180	0.001	-1.693
RNF183	Ring finger protein 183	0.000	-2.152
RNF187	Ring finger protein 187	0.001	1.594
ROGDI	Rogdi homolog	0.002	-1.570
RORC	RAR related orphan receptor C	0.000	2.433
RP2	Retinitis pigmentosa 2 (X-linked recessive)	0.000	-1.733
RPE	Ribulose-5-phosphate-3-epimerase	0.000	-1.815
RPL10	Ribosomal protein L10	0.005	1.601
RPL10A	Ribosomal protein l10a	0.003	1.726
RPL11	Ribosomal protein L11	0.004	1.650
RPL13	Ribosomal protein L13	0.001	1.841
RPL13A	Ribosomal protein l13a	0.006	1.734
RPL14	Ribosomal protein L14	0.005	1.630
RPL17	Ribosomal protein L17	0.000	2.188
RPL18A	Ribosomal protein l18a	0.006	1.565
RPL21	Ribosomal protein L21	0.002	1.847
RPL22	Ribosomal protein L22	0.000	2.025
RPL23	Ribosomal protein L23	0.003	1.893
RPL24	Ribosomal protein L24	0.006	1.691
RPL26	Ribosomal protein L26	0.001	1.986
RPL27	Ribosomal protein L27	0.006	1.802
RPL27A	Ribosomal protein l27a	0.004	1.810
RPL29	Ribosomal protein L29	0.001	1.750
RPL30	Ribosomal protein L30	0.001	2.043

RPL31	Ribosomal protein L31	0.002	1.922
RPL32	Ribosomal protein L32	0.000	2.109
RPL35	Ribosomal protein L35	0.001	1.896
RPL35A	Ribosomal protein l35a	0.001	2.068
RPL36	Ribosomal protein L36	0.001	1.690
RPL36A	Ribosomal protein l36a	0.004	1.881
RPL37	Ribosomal protein L37	0.001	2.269
RPL37A	Ribosomal protein l37a	0.008	1.730
RPL38	Ribosomal protein L38	0.001	1.748
RPL4	Ribosomal protein L4	0.003	1.613
RPL5	Ribosomal protein L5	0.004	1.641
RPL6	Ribosomal protein L6	0.002	1.599
RPL7	Ribosomal protein L7	0.001	1.811
RPL8	Ribosomal protein L8	0.006	1.610
RPLP1	Ribosomal protein lateral stalk subunit P1	0.001	1.816
RPLP2	Ribosomal protein lateral stalk subunit P2	0.005	1.827
RPS11	Ribosomal protein S11	0.008	1.646
RPS12	Ribosomal protein S12	0.005	1.790
RPS13	Ribosomal protein S13	0.002	1.678
RPS14	Ribosomal protein S14	0.013	1.646
RPS15	Ribosomal protein S15	0.006	1.710
RPS16	Ribosomal protein S16	0.001	1.669
RPS18	Ribosomal protein S18	0.004	1.709
RPS19	Ribosomal protein S19	0.000	2.135
RPS20	Ribosomal protein S20	0.002	1.906
RPS21	Ribosomal protein S21	0.001	2.189
RPS23	Ribosomal protein S23	0.001	2.287
RPS24	Ribosomal protein S24	0.001	2.050
RPS25	Ribosomal protein S25	0.000	2.363
RPS27	Ribosomal protein S27	0.000	2.059
RPS27A	Ribosomal protein s27a	0.008	1.738
RPS28	Ribosomal protein S28	0.003	1.736
RPS3	Ribosomal protein S3	0.004	1.796
RPS3A	Ribosomal protein S3A	0.013	1.687
RPS4X	Ribosomal protein S4, X-linked	0.005	1.887

RPS5	Ribosomal protein S5	0.002	1.722
RPS6	Ribosomal protein S6	0.006	1.642
RPS6KA1	Ribosomal protein S6 kinase A1	0.005	-1.507
RPS7	Ribosomal protein S7	0.004	1.807
RPS8	Ribosomal protein S8	0.001	1.858
RPS9	Ribosomal protein S9	0.007	1.624
RSAD2	Radical S-adenosyl methionine domain containing 2	0.000	-16.269
RSPRY1	Ring finger and SPRY domain containing 1	0.000	-2.249
RTCA	RNA 3'-terminal phosphate cyclase	0.001	-1.526
RTKN	Rhotekin	0.000	2.111
RTN1	Reticulon 1	0.000	-3.216
RTP3	Receptor transporter protein 3	0.000	-2.488
RTP4	Receptor transporter protein 4	0.001	-4.380
RWDD4	RWD domain containing 4	0.000	-2.170
RXRA	Retinoid X receptor alpha	0.006	1.524
S100A12	S100 calcium binding protein A12	0.007	-3.345
S100A13	S100 calcium binding protein A13	0.001	1.891
S100A16	S100 calcium binding protein A16	0.005	1.596
S100A2	S100 calcium binding protein A2	0.000	-3.376
S1PR3	Sphingosine-1-phosphate receptor 3	0.012	1.638
SAA1	Serum amyloid A1	0.000	-55.789
SAMD9	Sterile alpha motif domain containing 9	0.000	-7.615
SAR1B	Secretion associated Ras related gtpase 1B	0.006	-1.579
SASS6	SAS-6 centriolar assembly protein	0.009	-2.013
SBSPON	Somatomedin B and thrombospondin type 1 domain containing	0.003	-2.049
SCD	Stearoyl-coa desaturase	0.000	-2.928
SDS	Serine dehydratase	0.000	-3.763
SEC14L1	SEC14 like lipid binding 1	0.001	-1.573
SEC31A	SEC31 homolog A, COPII coat complex component	0.003	-1.548
SEL1L3	SEL1L family member 3	0.001	-1.621
SELENBP1	Selenium binding protein 1	0.010	1.763
SELT	Selenoprotein T	0.001	-1.657
SEMA4C	Semaphorin 4C	0.003	1.500
SEMA4G	Semaphorin 4G	0.013	1.664
SEPT4	Septin 4	0.005	-1.660

SERF2	Small EDRK-rich factor 2	0.004	1.646
SERINC4	Serine incorporator 4	0.002	1.517
SERPINA5	Serpin family A member 5	0.006	-1.807
SETD6	SET domain containing 6	0.000	-1.770
SFR1	SWI5 dependent homologous recombination repair protein 1	0.012	1.722
SGMS2	Sphingomyelin synthase 2	0.011	-1.533
SGPP1	Sphingosine-1-phosphate phosphatase 1	0.004	-1.730
SH3GLB2	SH3 domain containing GRB2 like endophilin B2	0.000	1.716
SH3PXD2B	SH3 and PX domains 2B	0.009	-1.725
SHC2	SHC adaptor protein 2	0.001	2.326
SHISA2	Shisa family member 2	0.007	-4.494
SHISA5	Shisa family member 5	0.012	-2.046
SHMT1	Serine hydroxymethyltransferase 1	0.012	1.645
SIGLEC1	Sialic acid binding Ig like lectin 1	0.004	-4.358
SIKE1	Suppressor of IKBKE 1	0.007	-1.504
SKAP1	Src kinase associated phosphoprotein 1	0.000	1.810
SLC13A5	Solute carrier family 13 member 5	0.000	-75.678
SLC16A1	Solute carrier family 16 member 1	0.012	-2.233
SLC16A11	Solute carrier family 16 member 11	0.011	1.556
SLC22A17	Solute carrier family 22 member 17	0.002	1.906
SLC22A4	Solute carrier family 22 member 4	0.001	2.147
SLC22A5	Solute carrier family 22 member 5	0.004	1.608
SLC23A1	Solute carrier family 23 member 1	0.008	-1.783
SLC25A13	Solute carrier family 25 member 13	0.001	-1.573
SLC25A15	Solute carrier family 25 member 15	0.000	-2.618
SLC25A22	Solute carrier family 25 member 22	0.001	-1.727
SLC25A25	Solute carrier family 25 member 25	0.007	-1.871
SLC25A26	Solute carrier family 25 member 26	0.005	1.503
SLC25A29	Solute carrier family 25 member 29	0.001	-1.848
SLC25A3	Solute carrier family 25 member 3	0.007	-1.581
SLC25A33	Solute carrier family 25 member 33	0.000	-2.500
SLC25A5	Solute carrier family 25 member 5	0.000	-3.003
SLC27A4	Solute carrier family 27 member 4	0.000	-2.416
SLC28A3	Solute carrier family 28 member 3	0.000	-3.113
SLC29A2	Solute carrier family 29 member 2	0.000	-1.901

SLC2A1	Solute carrier family 2 member 1	0.004	-2.563
SLC30A4	Solute carrier family 30 member 4	0.000	-2.031
SLC30A5	Solute carrier family 30 member 5	0.007	-1.602
SLC30A6	Solute carrier family 30 member 6	0.010	-1.503
SLC30A7	Solute carrier family 30 member 7	0.001	-1.715
SLC31A2	Solute carrier family 31 member 2	0.000	-2.387
SLC34A2	Solute carrier family 34 member 2	0.004	-2.587
SLC35B1	Solute carrier family 35 member B1	0.012	-1.524
SLC35C1	Solute carrier family 35 member C1	0.000	-2.082
SLC35F3	Solute carrier family 35 member F3	0.013	1.899
SLC37A3	Solute carrier family 37 member 3	0.007	-1.496
SLC37A4	Solute carrier family 37 member 4	0.000	-2.161
SLC38A1	Solute carrier family 38 member 1	0.000	-2.685
SLC38A3	Solute carrier family 38 member 3	0.004	2.498
SLC38A7	Solute carrier family 38 member 7	0.005	-1.587
SLC43A3	Solute carrier family 43 member 3	0.000	-2.884
SLC4A11	Solute carrier family 4 member 11	0.010	-2.350
SLC50A1	Solute carrier family 50 member 1	0.000	-2.163
SLC5A8	Solute carrier family 5 member 8	0.000	-4.885
SLC6A9	Solute carrier family 6 member 9	0.000	-1.893
SLC8B1	Solute carrier family 8 member B1	0.003	-1.676
SLCO4C1	Solute carrier organic anion transporter family member 4C1	0.000	-3.410
SLFN11	Schlafen family member 11	0.000	-6.081
SMARCA4	SWI/SNF related, matrix associated, actin dependent regulator of chromatin, subfamily a, member 4	0.004	-1.696
SMARCD3	SWI/SNF related, matrix associated, actin dependent regulator of chromatin, subfamily d, member 3	0.012	1.540
SMIM11A	Small integral membrane protein 11A	0.002	1.573
SMIM14	Small integral membrane protein 14	0.002	-1.666
SMOX	Spermine oxidase	0.000	-2.686
SMPD3	Sphingomyelin phosphodiesterase 3	0.006	-2.827
SMTNL2	Smoothelin like 2	0.000	-2.927
SNAP29	Synaptosome associated protein 29	0.001	-1.506
SNPH	Syntaphilin	0.012	1.772
SNX16	Sorting nexin 16	0.004	-1.498
SNX19	Sorting nexin 19	0.000	-1.528
SOCS4	Suppressor of cytokine signaling 4	0.000	-1.864

SOD2	Superoxide dismutase 2, mitochondrial	0.004	1.520
SPAG1	Sperm associated antigen 1	0.010	-1.609
SPAG9	Sperm associated antigen 9	0.000	-1.823
SPC24	SPC24, NDC80 kinetochore complex component	0.000	-3.252
SPDEF	SAM pointed domain containing ETS transcription factor	0.006	-3.403
SPDYC	Speedy/RINGO cell cycle regulator family member C	0.003	-2.037
SPHK1	Sphingosine kinase 1	0.000	-2.905
SPOUT1	SPOUT domain containing methyltransferase 1	0.000	-1.974
SPP1	Secreted phosphoprotein 1	0.012	-3.309
SPPL3	Signal peptide peptidase like 3	0.000	-1.663
SPSB3	Spla/ryanodine receptor domain and SOCS box containing 3	0.002	1.499
SREBF1	Sterol regulatory element binding transcription factor 1	0.003	-1.518
SRPX	Sushi repeat containing protein, X-linked	0.000	-2.436
SRSF9	Serine and arginine rich splicing factor 9	0.000	-1.713
SRXN1	Sulfiredoxin 1	0.000	-3.677
SS18L1	SS18L1, nbaf chromatin remodeling complex subunit	0.000	-6.859
SSBP4	Single stranded DNA binding protein 4	0.002	1.552
SSFA2	Sperm specific antigen 2	0.005	-1.712
SSR2	Signal sequence receptor subunit 2	0.008	1.585
SSUH2	Ssu-2 homolog (C. Elegans)	0.002	-5.792
SSX2IP	SSX family member 2 interacting protein	0.002	-1.857
ST3GAL2	ST3 beta-galactoside alpha-2,3-sialyltransferase 2	0.000	1.779
ST3GAL3	ST3 beta-galactoside alpha-2,3-sialyltransferase 3	0.005	1.596
ST5	Suppression of tumorigenicity 5	0.000	1.864
STAT1	Signal transducer and activator of transcription 1	0.007	-2.416
STAT2	Signal transducer and activator of transcription 2	0.004	-2.113
STK17B	Serine/threonine kinase 17b	0.011	-2.141
STK26	Serine/threonine protein kinase 26	0.000	-2.596
STK3	Serine/threonine kinase 3	0.000	-1.538
STK38L	Serine/threonine kinase 38 like	0.003	-1.537
STX3	Syntaxin 3	0.000	-2.146
STYK1	Serine/threonine/tyrosine kinase 1	0.002	-2.564
STYXL1	Serine/threonine/tyrosine interacting-like 1	0.007	1.778
SUCO	SUN domain containing ossification factor	0.000	-1.669
Sult1a1	Sulfotransferase family 1A, phenol-preferring, member 1	0.000	-3.056

SUV39H1	Suppressor of variegation 3-9 homolog 1	0.003	1.573
SVBP	Small vasohibin binding protein	0.003	1.620
SVEP1	Sushi, von Willebrand factor type A, EGF and pentraxin domain containing 1	0.000	3.302
SVOPL	SVOP like	0.002	2.643
SWI5	SWI5 homologous recombination repair protein	0.000	1.757
SYK	Spleen associated tyrosine kinase	0.001	-2.113
SYNE3	Spectrin repeat containing nuclear envelope family member 3	0.011	1.635
SYNE4	Spectrin repeat containing nuclear envelope family member 4	0.001	-2.146
TAF13	TATA-box binding protein associated factor 13	0.009	-1.513
TAF7	TATA-box binding protein associated factor 7	0.008	1.509
TAP1	Transporter 1, ATP binding cassette subfamily B member	0.001	-2.490
TATDN1	Tatd dnase domain containing 1	0.003	1.954
TBC1D30	TBC1 domain family member 30	0.000	-2.282
TCAF2	TRPM8 channel associated factor 2	0.000	-5.027
TCEB1	Transcription elongation factor B subunit 1	0.001	-1.577
TCERG1	Transcription elongation regulator 1	0.008	-1.524
TCP11L1	T-complex 11 like 1	0.001	1.546
TCP11L2	T-complex 11 like 2	0.008	1.523
TDGF1	Teratocarcinoma-derived growth factor 1	0.000	-18.426
TDRD7	Tudor domain containing 7	0.003	-2.016
TDRKH	Tudor and KH domain containing	0.006	-1.550
TFAP4	Transcription factor AP-4 (activating enhancer binding protein 4)	0.001	1.621
TFG	TRK-fused gene	0.001	-1.540
TFRC	Transferrin receptor	0.001	-2.654
TGFB1I1	Transforming growth factor beta 1 induced transcript 1	0.001	1.757
TGM5	Transglutaminase 5	0.000	-42.438
THAP3	THAP domain containing 3	0.000	1.645
THRA	Thyroid hormone receptor, alpha	0.000	1.899
THSD1	Thrombospondin type 1 domain containing 1	0.009	1.605
TIA1	TIA1 cytotoxic granule-associated RNA binding protein	0.011	-1.570
TIFA	TRAF interacting protein with forkhead associated domain	0.000	-4.573
TIGAR	TP53 induced glycolysis regulatory phosphatase	0.000	-4.080
TK1	Thymidine kinase 1	0.000	-1.886
TM7SF2	Transmembrane 7 superfamily member 2	0.006	-1.673

TM9SF3	Transmembrane 9 superfamily member 3	0.000	-1.652
TMCC3	Transmembrane and coiled-coil domain family 3	0.005	-2.069
TMED3	Transmembrane p24 trafficking protein 3	0.000	-1.814
TMED5	Transmembrane p24 trafficking protein 5	0.000	-1.957
TMEM109	Transmembrane protein 109	0.000	1.582
TMEM120A	Transmembrane protein 120A	0.012	-1.516
TMEM132A	Transmembrane protein 132A	0.003	1.636
TMEM165	Transmembrane protein 165	0.000	-1.781
TMEM184A	Transmembrane protein 184A	0.009	1.597
TMEM2	Transmembrane protein 2	0.006	-1.527
TMEM218	Transmembrane protein 218	0.000	1.616
TMEM229B	Transmembrane protein 229B	0.000	-1.987
TMEM268	Transmembrane protein 268	0.001	-1.959
TMEM38B	Transmembrane protein 38B	0.003	-1.547
TMEM40	Transmembrane protein 40	0.000	-17.404
TMEM41B	Transmembrane protein 41B	0.000	-2.140
TMEM64	Transmembrane protein 64	0.002	-2.228
TMEM79	Transmembrane protein 79	0.002	-1.632
TMEM8A	Transmembrane protein 8A	0.001	-1.598
TMEM98	Transmembrane protein 98	0.000	2.181
TMPRSS6	Transmembrane protease, serine 6	0.007	1.935
TMTC1	Transmembrane and tetratricopeptide repeat containing 1	0.006	-1.880
TNFRSF11B	TNF receptor superfamily member 11b	0.000	3.296
TNFRSF1B	TNF receptor superfamily member 1B	0.000	-1.840
TNFRSF6B	TNF receptor superfamily member 6b	0.001	-7.441
TNKS1BP1	Tankyrase 1 binding protein 1	0.002	1.516
TNS2	Tensin 2	0.000	1.739
TOM1L2	Target of myb1 like 2 membrane trafficking protein	0.010	-1.592
TOMM34	Translocase of outer mitochondrial membrane 34	0.000	-2.048
TOMM7	Translocase of outer mitochondrial membrane 7	0.007	1.577
TOP1	Topoisomerase (DNA) I	0.002	-1.692
TOP2A	Topoisomerase (DNA) II alpha	0.002	-3.072
TOR1AIP2	Torsin 1A interacting protein 2	0.001	-1.806
TOR1B	Torsin family 1 member B	0.000	-2.174
TP53I11	Tumor protein p53 inducible protein 11	0.002	-2.026

TP53I13	Tumor protein p53 inducible protein 13	0.001	1.627
TPCN1	Two pore segment channel 1	0.000	-3.504
TPGS1	Tubulin polyglutamylase complex subunit 1	0.007	1.631
TPMT	Thiopurine S-methyltransferase	0.000	-1.896
TPT1	Tumor protein, translationally-controlled 1	0.000	1.905
TREX1	Three prime repair exonuclease 1	0.009	-2.345
TRIM21	Tripartite motif containing 21	0.002	-2.091
TRIM26	Tripartite motif containing 26	0.001	-1.658
TRIM47	Tripartite motif containing 47	0.004	1.606
TRIM5	Tripartite motif containing 5	0.000	-3.158
TRIM65	Tripartite motif containing 65	0.007	1.536
TRIM9	Tripartite motif containing 9	0.000	-22.906
TRIT1	Trna isopentenyltransferase 1	0.001	-1.811
TRMT112	Trna methyltransferase 11-2 homolog (S. Cerevisiae)	0.013	1.503
TRMT13	Trna methyltransferase 13 homolog	0.002	-1.998
TRMT6	Trna methyltransferase 6	0.000	-1.864
TSNAX	Translin associated factor X	0.000	-2.064
TSPAN33	Tetraspanin 33	0.011	1.898
TSPAN7	Tetraspanin 7	0.004	1.890
TST	Thiosulfate sulfurtransferase	0.000	-2.779
TSTD1	Thiosulfate sulfurtransferase like domain containing 1	0.001	-1.772
TTC13	Tetratricopeptide repeat domain 13	0.001	-1.808
TTC22	Tetratricopeptide repeat domain 22	0.000	-3.055
TTC39B	Tetratricopeptide repeat domain 39B	0.000	-2.817
TUBA1A	Tubulin alpha 1a	0.004	1.968
TUBB4A	Tubulin beta 4A class iva	0.007	-2.214
TXNRD1	Thioredoxin reductase 1	0.000	-6.111
UBA2	Ubiquitin like modifier activating enzyme 2	0.000	-1.517
UBA7	Ubiquitin like modifier activating enzyme 7	0.001	-5.214
UBE2J1	Ubiquitin conjugating enzyme E2 J1	0.000	-1.804
UBE2T	Ubiquitin conjugating enzyme E2 T	0.000	1.531
UBIAD1	Ubia prenyltransferase domain containing 1	0.000	-1.805
UCP2	Uncoupling protein 2	0.001	-2.184
UEVLD	UEV and lactate/malate dehyrogenase domains	0.000	-1.825
UGGT1	UDP-glucose glycoprotein glucosyltransferase 1	0.012	-1.519

UNC93A	Unc-93 homolog A (C. Elegans)	0.000	-5.414
UPK1B	Uroplakin 1B	0.000	-12.362
UQCRB	Ubiquinol-cytochrome c reductase binding protein	0.006	-1.508
UQCRFS1	Ubiquinol-cytochrome c reductase, Rieske iron-sulfur polypeptide 1	0.011	-1.587
USHBP1	USH1 protein network component harmonin binding protein 1	0.007	1.746
USP14	Ubiquitin specific peptidase 14	0.001	-1.512
USP18	Ubiquitin specific peptidase 18	0.000	-7.970
USP25	Ubiquitin specific peptidase 25	0.009	-1.655
USP31	Ubiquitin specific peptidase 31	0.002	-2.038
USP43	Ubiquitin specific peptidase 43	0.001	-2.415
UTP18	UTP18, small subunit processome component	0.011	1.622
UTP20	UTP20, small subunit processome component	0.000	-2.152
UXS1	UDP-glucuronate decarboxylase 1	0.004	-1.629
VGLL1	Vestigial like family member 1	0.005	-4.700
VKORC1	Vitamin K epoxide reductase complex subunit 1	0.010	1.512
VMA21	VMA21 vacuolar H+-atpase homolog (S. Cerevisiae)	0.000	-1.659
VNN2	Vanin 2	0.000	-12.739
VSIG1	V-set and immunoglobulin domain containing 1	0.012	2.963
WBSCR17	Williams-Beuren syndrome chromosome region 17	0.000	-7.995
Wfdc21	WAP four-disulfide core domain 21	0.012	-6.273
WISP2	WNT1 inducible signaling pathway protein 2	0.002	2.612
WNT11	Wnt family member 11	0.000	-5.143
WRB	Tryptophan rich basic protein	0.007	1.501
WSB2	WD repeat and SOCS box containing 2	0.000	-1.548
XAF1	XIAP associated factor 1	0.000	-4.736
XPNPEP1	X-prolyl aminopeptidase 1	0.000	-1.812
XPO1	Exportin 1	0.002	-1.687
YARS	Tyrosyl-trna synthetase	0.007	-1.819
YEATS2	YEATS domain containing 2	0.000	-1.919
YIPF4	Yip1 domain family member 4	0.001	-1.512
YTHDC2	YTH domain containing 2	0.000	-2.132
ZBP1	Z-DNA binding protein 1	0.000	-8.056
ZFAND2B	Zinc finger AN1-type containing 2B	0.000	1.528
ZFP36L2	ZFP36 ring finger protein like 2	0.003	1.742
ZHX1	Zinc fingers and homeoboxes 1	0.002	-1.500

ZNF395	Zinc finger protein 395	0.004	1.659
ZNF521	Zinc finger protein 521	0.009	1.855
ZNF70	Zinc finger protein 70	0.008	1.637
ZNF750	Zinc finger protein 750	0.000	-3.143
ZNFX1	Zinc finger NFX1-type containing 1	0.002	-3.269
ZNRF2	Zinc and ring finger 2, E3 ubiquitin protein ligase	0.007	-1.548
ZYX	Zyxin	0.001	1.591

[1] Fold changes are up or down in PUFA, not-pregnant, low diet compared to PUFA, not-pregnant, high diet

Table S7. Differentially expressed genes in PUFA, Pregnant, Low diet versus PUFA, Pregnant, High diet

Symbol	Entrez Gene Name	p-value	Fold Change[1]
A2M	Alpha-2-macroglobulin	0.009	-1.536
ABAT	4-aminobutyrate aminotransferase	0.012	-1.502
ABCA2	ATP binding cassette subfamily A member 2	0.004	-1.509
ABCA9	ATP binding cassette subfamily A member 9	0.009	-1.540
ABCB1	ATP binding cassette subfamily B member 1	0.001	-1.645
ABCC10	ATP binding cassette subfamily C member 10	0.002	-1.574
ABCC9	ATP binding cassette subfamily C member 9	0.001	-1.600
ABHD1	Abhydrolase domain containing 1	0.026	1.535
Acan	Aggrecan	0.022	-1.784
ACKR1	Atypical chemokine receptor 1 (Duffy blood group)	0.000	2.230
ADAM19	ADAM metallopeptidase domain 19	0.002	-1.520
ADAMTS12	ADAM metallopeptidase with thrombospondin type 1 motif 12	0.022	-1.788
ADAMTS2	ADAM metallopeptidase with thrombospondin type 1 motif 2	0.000	-1.867
ADAMTS9	ADAM metallopeptidase with thrombospondin type 1 motif 9	0.010	-1.583
ADAMTSL1	ADAMTS like 1	0.001	-1.793
ADCY5	Adenylate cyclase 5	0.002	-1.548
ADCYAP1R1	ADCYAP receptor type I	0.015	-2.510
ADD3	Adducin 3	0.000	-1.515
ADGB	Androglobin	0.005	-1.982
ADGRG6	Adhesion G protein-coupled receptor G6	0.025	-1.635
ADGRL3	Adhesion G protein-coupled receptor L3	0.008	-1.756
ADIRF	Adipogenesis regulatory factor	0.000	1.706
AFF4	AF4/FMR2 family member 4	0.007	-1.503
AFMID	Arylformamidase	0.020	-1.657
AK9	Adenylate kinase 9	0.003	-2.033
AKAP12	A-kinase anchoring protein 12	0.001	-1.642
AKAP4	A-kinase anchoring protein 4	0.005	2.423
AKAP5	A-kinase anchoring protein 5	0.005	-1.496
AKAP8	A-kinase anchoring protein 8	0.000	-1.540
AKAP9	A-kinase anchoring protein 9	0.005	-1.503
AKR1A1	Aldo-keto reductase family 1 member A1	0.000	1.602
ALG5	ALG5, dolichyl-phosphate beta-glucosyltransferase	0.000	1.497

ALMS1	ALMS1, centrosome and basal body associated protein	0.000	-1.778
ALOX5AP	Arachidonate 5-lipoxygenase activating protein	0.012	1.778
ALPK3	Alpha kinase 3	0.002	-1.967
ANKRD11	Ankyrin repeat domain 11	0.001	-1.563
ANKRD12	Ankyrin repeat domain 12	0.000	-1.784
ANKRD26	Ankyrin repeat domain 26	0.000	-2.008
ANKRD6	Ankyrin repeat domain 6	0.000	-1.640
ANO4	Anoctamin 4	0.009	-1.838
ANXA1	Annexin A1	0.018	1.573
APBB3	Amyloid beta precursor protein binding family B member 3	0.005	-1.516
APC	APC, WNT signaling pathway regulator	0.000	-1.937
APIP	APAF1 interacting protein	0.000	1.535
APOA1	Apolipoprotein A1	0.029	1.500
APOM	Apolipoprotein M	0.000	1.514
AR	Androgen receptor	0.012	-1.610
ARAP2	Arfgap with rhogap domain, ankyrin repeat and PH domain 2	0.000	-1.770
ARG2	Arginase 2	0.004	1.632
ARHGAP20	Rho gtpase activating protein 20	0.019	-1.498
ARHGAP21	Rho gtpase activating protein 21	0.000	-1.667
ARHGAP44	Rho gtpase activating protein 44	0.008	-1.526
ARHGEF28	Rho guanine nucleotide exchange factor 28	0.000	-1.527
ARHGEF38	Rho guanine nucleotide exchange factor 38	0.004	-1.509
ARHGEF4	Rho guanine nucleotide exchange factor 4	0.031	-1.504
ARID1B	AT-rich interaction domain 1B	0.001	-1.512
ARID4A	AT-rich interaction domain 4A	0.000	-1.696
ARID4B	AT-rich interaction domain 4B	0.000	-1.762
ARL14EP	ADP ribosylation factor like gtpase 14 effector protein	0.000	1.524
ARL6	ADP ribosylation factor like gtpase 6	0.000	-1.532
ARPC3	Actin related protein 2/3 complex subunit 3	0.000	1.546
ASH1L	ASH1 like histone lysine methyltransferase	0.000	-1.575
ASNS	Asparagine synthetase (glutamine-hydrolyzing)	0.000	1.707
ASPN	Asporin	0.004	-1.554
ATAD2B	Atpase family, AAA domain containing 2B	0.000	-1.645
ATAD5	Atpase family, AAA domain containing 5	0.000	-1.692
ATG2B	Autophagy related 2B	0.000	-1.507

ATM	ATM serine/threonine kinase	0.001	-1.542
ATP5G3	ATP synthase, H+ transporting, mitochondrial Fo complex subunit C3 (subunit 9)	0.000	1.525
ATP5H	ATP synthase, H+ transporting, mitochondrial Fo complex subunit D	0.000	1.505
ATP5J	ATP synthase, H+ transporting, mitochondrial Fo complex subunit F6	0.000	1.631
ATP5J2	ATP synthase, H+ transporting, mitochondrial Fo complex subunit F2	0.000	1.718
ATP5O	ATP synthase, H+ transporting, mitochondrial F1 complex, O subunit	0.000	1.610
ATPIF1	Atpase inhibitory factor 1	0.000	1.507
ATRAID	All-trans retinoic acid induced differentiation factor	0.000	1.496
ATRNL1	Attractin like 1	0.001	-1.807
ATRX	ATRX, chromatin remodeler	0.000	-1.656
B2M	Beta-2-microglobulin	0.004	1.618
BAZ2B	Bromodomain adjacent to zinc finger domain 2B	0.000	-2.068
BBX	BBX, HMG-box containing	0.000	-1.741
BCL2L15	BCL2 like 15	0.018	1.760
BCLAF1	BCL2 associated transcription factor 1	0.000	-1.576
BDP1	B double prime 1, subunit of RNA polymerase III transcription initiation factor IIIB	0.001	-1.718
BEX5	Brain expressed X-linked 5	0.000	2.087
BICC1	Bicc family RNA binding protein 1	0.002	-1.561
BICD2	BICD cargo adaptor 2	0.000	-1.528
BICDL1	BICD family like cargo adaptor 1	0.003	-1.497
BOD1L1	Biorientation of chromosomes in cell division 1 like 1	0.000	-1.629
BPIFB1	BPI fold containing family B member 1	0.007	-1.797
BPTF	Bromodomain PHD finger transcription factor	0.000	-1.802
BRCA2	BRCA2, DNA repair associated	0.005	-1.665
BRWD1	Bromodomain and WD repeat domain containing 1	0.000	-1.614
BTBD8	BTB domain containing 8	0.000	-1.863
BTF3	Basic transcription factor 3	0.000	1.699
C12orf57	Chromosome 12 open reading frame 57	0.002	1.573
C14orf166	Chromosome 14 open reading frame 166	0.000	1.719
C19orf66	Chromosome 19 open reading frame 66	0.004	1.507
C19orf70	Chromosome 19 open reading frame 70	0.000	1.548
C1orf54	Chromosome 1 open reading frame 54	0.001	1.678
C1QTNF7	C1q and tumor necrosis factor related protein 7	0.003	1.522
CA1	Carbonic anhydrase 1	0.000	-18.491
CA4	Carbonic anhydrase 4	0.002	1.735

CACNA1D	Calcium voltage-gated channel subunit alpha1 D	0.001	-1.813
CACNA2D1	Calcium voltage-gated channel auxiliary subunit alpha2delta 1	0.001	-1.769
CAMSAP1	Calmodulin regulated spectrin associated protein 1	0.000	-1.573
CAMTA1	Calmodulin binding transcription activator 1	0.000	-1.710
CASP8	Caspase 8	0.010	1.614
CASP8AP2	Caspase 8 associated protein 2	0.000	-1.646
CATSPERD	Cation channel sperm associated auxiliary subunit delta	0.023	-1.635
CATSPERG	Cation channel sperm associated auxiliary subunit gamma	0.006	-1.666
CC2D2A	Coiled-coil and C2 domain containing 2A	0.001	-1.659
CCDC13	Coiled-coil domain containing 13	0.009	-1.902
CCDC141	Coiled-coil domain containing 141	0.029	-1.654
CCDC142	Coiled-coil domain containing 142	0.002	-1.518
CCDC146	Coiled-coil domain containing 146	0.012	-1.802
CCDC150	Coiled-coil domain containing 150	0.001	-1.672
CCDC158	Coiled-coil domain containing 158	0.000	-1.795
CCDC170	Coiled-coil domain containing 170	0.000	-1.974
CCDC180	Coiled-coil domain containing 180	0.013	-1.878
CCDC186	Coiled-coil domain containing 186	0.000	-1.817
CCDC30	Coiled-coil domain containing 30	0.016	-1.546
CCDC39	Coiled-coil domain containing 39	0.000	-2.394
CCDC40	Coiled-coil domain containing 40	0.009	-1.817
CCDC58	Coiled-coil domain containing 58	0.000	1.584
CCDC88A	Coiled-coil domain containing 88A	0.002	-1.686
CCNT2	Cyclin T2	0.000	-1.497
CCP110	Centriolar coiled-coil protein 110	0.000	-1.518
CCPG1	Cell cycle progression 1	0.000	-1.580
CCRL2	C-C motif chemokine receptor like 2	0.000	2.159
CD163	CD163 molecule	0.016	-1.941
CD1E	CD1e molecule	0.017	1.554
CD48	CD48 molecule	0.000	1.950
CD59	CD59 molecule	0.001	1.687
CD63	CD63 molecule	0.000	1.567
CD9	CD9 molecule	0.000	1.523
CDH17	Cadherin 17	0.009	1.613
CDK8	Cyclin dependent kinase 8	0.001	-1.498

CDKN2AIPNL	CDKN2A interacting protein N-terminal like	0.000	1.578
CENPJ	Centromere protein J	0.001	-1.576
CEP104	Centrosomal protein 104	0.000	-1.613
CEP112	Centrosomal protein 112	0.001	-1.609
CEP120	Centrosomal protein 120	0.000	-1.650
CEP128	Centrosomal protein 128	0.001	-1.576
CEP135	Centrosomal protein 135	0.000	-1.798
CEP162	Centrosomal protein 162	0.001	-1.678
CEP170	Centrosomal protein 170	0.000	-1.716
CEP250	Centrosomal protein 250	0.005	-1.518
CEP295	Centrosomal protein 295	0.000	-1.604
CEP350	Centrosomal protein 350	0.000	-2.086
CEP83	Centrosomal protein 83	0.000	-1.553
CEP85L	Centrosomal protein 85 like	0.002	-1.612
CEP89	Centrosomal protein 89	0.000	-1.583
CFAP100	Cilia and flagella associated protein 100	0.006	-1.788
CFAP44	Cilia and flagella associated protein 44	0.004	-1.933
CFAP69	Cilia and flagella associated protein 69	0.002	-1.651
CFH	Complement factor H	0.014	-1.541
CH25H	Cholesterol 25-hydroxylase	0.018	1.578
CHAD	Chondroadherin	0.002	-1.631
CHD1	Chromodomain helicase DNA binding protein 1	0.000	-1.552
CHD6	Chromodomain helicase DNA binding protein 6	0.005	-1.541
CHD7	Chromodomain helicase DNA binding protein 7	0.003	-1.598
CHD9	Chromodomain helicase DNA binding protein 9	0.000	-1.909
CHST11	Carbohydrate (chondroitin 4) sulfotransferase 11	0.000	-1.743
CIRBP	Cold inducible RNA binding protein	0.000	1.699
CKAP2	Cytoskeleton associated protein 2	0.008	-1.540
CKAP5	Cytoskeleton associated protein 5	0.000	-1.531
CLIC1	Chloride intracellular channel 1	0.000	1.613
CLIP1	CAP-Gly domain containing linker protein 1	0.000	-2.091
CMSS1	Cms1 ribosomal small subunit homolog (yeast)	0.000	1.536
CNIH4	Cornichon family AMPA receptor auxiliary protein 4	0.000	1.569
CNTLN	Centlein	0.000	-1.671

CNTN4	Contactin 4	0.018	-1.776
CNTNAP1	Contactin associated protein 1	0.021	-1.629
CNTRL	Centriolin	0.000	-1.788
COL23A1	Collagen type XXIII alpha 1 chain	0.002	-1.646
COL27A1	Collagen type XXVII alpha 1	0.024	-1.536
COMMD1	Copper metabolism domain containing 1	0.000	1.612
COX5B	Cytochrome c oxidase subunit 5B	0.000	1.551
COX6B1	Cytochrome c oxidase subunit 6B1	0.000	1.535
COX7A2	Cytochrome c oxidase subunit 7A2	0.001	1.505
COX7A2L	Cytochrome c oxidase subunit 7A2 like	0.000	1.689
Cox7c	Cytochrome c oxidase subunit viic	0.000	2.647
CPED1	Cadherin like and PC-esterase domain containing 1	0.000	-1.535
CPXM1	Carboxypeptidase X (M14 family), member 1	0.000	-1.638
CR2	Complement component 3d receptor 2	0.000	-2.739
CRAMP1	Cramped chromatin regulator homolog 1	0.001	-1.537
CRYBB1	Crystallin beta B1	0.000	1.829
CRYM	Crystallin mu	0.018	1.947
CSPP1	Centrosome and spindle pole associated protein 1	0.000	-1.820
CTAGE5	CTAGE family member 5, ER export factor	0.008	-2.337
CTTNBP2	Cortactin binding protein 2	0.000	-1.563
CYCS	Cytochrome c, somatic	0.000	1.821
CYP20A1	Cytochrome P450 family 20 subfamily A member 1	0.000	1.523
CYTH3	Cytohesin 3	0.000	-1.511
DAD1	Defender against cell death 1	0.000	1.546
DCDC1	Doublecortin domain containing 1	0.001	-2.272
DCLK2	Doublecortin like kinase 2	0.011	-1.687
DEGS1	Delta 4-desaturase, sphingolipid 1	0.002	1.496
DGKH	Diacylglycerol kinase eta	0.004	-2.022
DGUOK	Deoxyguanosine kinase	0.000	1.605
DKK1	Dickkopf WNT signaling pathway inhibitor 1	0.025	1.835
DLC1	DLC1 Rho gtpase activating protein	0.001	-1.512
DMGDH	Dimethylglycine dehydrogenase	0.004	-1.615
DNAH12	Dynein axonemal heavy chain 12	0.000	-2.003
DNAH2	Dynein axonemal heavy chain 2	0.003	-2.125
DNAH5	Dynein axonemal heavy chain 5	0.002	-2.077

DNAH7	Dynein axonemal heavy chain 7	0.005	-1.687
DNALI1	Dynein axonemal light intermediate chain 1	0.018	-1.627
DNASE2	Deoxyribonuclease II, lysosomal	0.003	1.655
DNMT3A	DNA methyltransferase 3 alpha	0.003	-1.523
DOK6	Docking protein 6	0.001	-2.210
DPM3	Dolichyl-phosphate mannosyltransferase subunit 3	0.001	1.560
DST	Dystonin	0.000	-1.831
DUT	Deoxyuridine triphosphatase	0.001	1.535
DYNLRB1	Dynein light chain roadblock-type 1	0.000	1.507
DZIP3	DAZ interacting zinc finger protein 3	0.000	-1.688
EEA1	Early endosome antigen 1	0.000	-2.045
EEF1B2	Eukaryotic translation elongation factor 1 beta 2	0.000	1.864
EEF1D	Eukaryotic translation elongation factor 1 delta	0.000	1.697
EEF1G	Eukaryotic translation elongation factor 1 gamma	0.000	1.668
EEF2K	Eukaryotic elongation factor 2 kinase	0.000	-1.641
EFCAB12	EF-hand calcium binding domain 12	0.030	-1.712
EFCAB6	EF-hand calcium binding domain 6	0.033	-1.574
EGFR	Epidermal growth factor receptor	0.000	-1.581
EIF1AD	Eukaryotic translation initiation factor 1A domain containing	0.000	1.528
EIF2S3	Eukaryotic translation initiation factor 2 subunit gamma	0.000	1.580
EIF3E	Eukaryotic translation initiation factor 3 subunit E	0.000	1.787
EIF3H	Eukaryotic translation initiation factor 3 subunit H	0.000	1.532
EIF3I	Eukaryotic translation initiation factor 3 subunit I	0.000	1.590
EIF3K	Eukaryotic translation initiation factor 3 subunit K	0.000	1.531
EIF3M	Eukaryotic translation initiation factor 3 subunit M	0.000	1.990
EIF4G3	Eukaryotic translation initiation factor 4 gamma 3	0.000	-1.768
ELK3	ELK3, ETS transcription factor	0.000	-1.590
EMILIN2	Elastin microfibril interfacer 2	0.004	-1.534
EML5	Echinoderm microtubule associated protein like 5	0.000	-1.999
ENPP6	Ectonucleotide pyrophosphatase/phosphodiesterase 6	0.000	-1.915
EPC1	Enhancer of polycomb homolog 1	0.000	-1.552
ERCC6L2	ERCC excision repair 6 like 2	0.000	-1.655
ERICH3	Glutamate rich 3	0.009	-1.961
ERN2	Endoplasmic reticulum to nucleus signaling 2	0.010	-1.611
ESCO1	Establishment of sister chromatid cohesion N-acetyltransferase 1	0.000	-1.883

ESR2	Estrogen receptor 2	0.008	-1.669
ETV3	ETS variant 3	0.000	-1.564
F8	Coagulation factor VIII	0.001	-1.505
FABP7	Fatty acid binding protein 7	0.000	1.914
FAM161A	Family with sequence similarity 161 member A	0.002	-1.537
FAM169A	Family with sequence similarity 169 member A	0.001	-1.652
FAM171B	Family with sequence similarity 171 member B	0.014	-1.513
FAM185A	Family with sequence similarity 185 member A	0.001	-1.602
FAM193B	Family with sequence similarity 193 member B	0.002	-1.533
FAM214A	Family with sequence similarity 214 member A	0.000	-1.846
FAM65C	Family with sequence similarity 65 member C	0.009	-1.516
FBL	Fibrillarin	0.000	1.511
FBN1	Fibrillin 1	0.000	-1.568
FCER2	Fc fragment of ige receptor II	0.023	2.118
FCF1	FCF1 rrna-processing protein	0.000	1.654
FGF1	Fibroblast growth factor 1	0.002	-1.603
FGFBP1	Fibroblast growth factor binding protein 1	0.031	2.145
FHAD1	Forkhead associated phosphopeptide binding domain 1	0.001	-2.270
FILIP1	Filamin A interacting protein 1	0.029	-1.501
FILIP1L	Filamin A interacting protein 1 like	0.001	-1.533
FKBP11	FK506 binding protein 11	0.000	1.633
FKBP7	FK506 binding protein 7	0.001	1.534
FMNL2	Formin like 2	0.000	-1.572
FMO4	Flavin containing monooxygenase 4	0.006	-1.504
FNBP4	Formin binding protein 4	0.000	-2.198
FRMPD2	FERM and PDZ domain containing 2	0.005	-2.195
FRY	FRY microtubule binding protein	0.004	-1.521
FSD2	Fibronectin type III and SPRY domain containing 2	0.006	-1.509
FSIP1	Fibrous sheath interacting protein 1	0.032	-1.698
GBP2	Guanylate binding protein 2	0.032	-3.624
GCC2	GRIP and coiled-coil domain containing 2	0.000	-1.849
GDF7	Growth differentiation factor 7	0.006	-1.776
GEMIN2	Gem nuclear organelle associated protein 2	0.000	1.627
GLIPR1	GLI pathogenesis related 1	0.000	1.530
GLRX	Glutaredoxin	0.000	1.601

GLRX2	Glutaredoxin 2	0.000	1.562
GLRX3	Glutaredoxin 3	0.000	1.550
GNAL	G protein subunit alpha L	0.005	-1.719
GNPTAB	N-acetylglucosamine-1-phosphate transferase alpha and beta subunits	0.000	-1.596
GOLGA3	Golgin A3	0.000	-1.605
GOLGA4	Golgin A4	0.000	-1.773
GOLGB1	Golgin B1	0.000	-1.667
GPATCH8	G-patch domain containing 8	0.000	-1.652
GPR176	G protein-coupled receptor 176	0.016	-1.503
GRAMD1B	GRAM domain containing 1B	0.012	-1.531
GREB1	Growth regulation by estrogen in breast cancer 1	0.012	-1.815
GRHPR	Glyoxylate reductase/hydroxypyruvate reductase	0.000	1.613
GRK4	G protein-coupled receptor kinase 4	0.000	-2.022
GRN	Granulin	0.003	1.509
GSTO1	Glutathione S-transferase omega 1	0.002	1.558
GTF2B	General transcription factor IIB	0.006	1.580
GUCY1A2	Guanylate cyclase 1 soluble subunit alpha 2	0.009	-1.573
GUCY1A3	Guanylate cyclase 1 soluble subunit alpha	0.000	-1.518
HAX1	HCLS1 associated protein X-1	0.000	1.557
HEBP1	Heme binding protein 1	0.000	1.632
HEBP2	Heme binding protein 2	0.000	1.496
HERC2	HECT and RLD domain containing E3 ubiquitin protein ligase 2	0.000	-1.532
HEXB	Hexosaminidase subunit beta	0.003	2.521
HHIP	Hedgehog interacting protein	0.022	-1.756
HIGD1A	HIG1 hypoxia inducible domain family member 1A	0.002	1.982
HIKESHI	Hikeshi, heat shock protein nuclear import factor	0.000	1.517
HINT1	Histidine triad nucleotide binding protein 1	0.000	1.717
HIP1	Huntingtin interacting protein 1	0.004	-1.522
HIST1H2BD	Histone cluster 1, h2bd	0.010	1.500
HIST1H4J	Histone cluster 1, h4j	0.000	2.083
HLA-DMA	Major histocompatibility complex, class II, DM alpha	0.003	1.592
HMCN1	Hemicentin 1	0.002	-2.141
HMGN5	High mobility group nucleosome binding domain 5	0.001	1.598
HMMR	Hyaluronan mediated motility receptor	0.000	-1.559
HOOK1	Hook microtubule-tethering protein 1	0.001	-1.527

HR	Hair growth associated	0.004	-1.590
HSPA12A	Heat shock protein family A (Hsp70) member 12A	0.004	-1.526
HSPB11	Heat shock protein family B (small) member 11	0.000	1.535
HSPE1	Heat shock protein family E (Hsp10) member 1	0.000	1.674
IDNK	IDNK, gluconokinase	0.006	1.627
IFI35	Interferon induced protein 35	0.012	1.537
IFITM1	Interferon induced transmembrane protein 1	0.013	1.944
IGF2	Insulin like growth factor 2	0.002	1.558
IGSF10	Immunoglobulin superfamily member 10	0.012	-1.683
IL1RN	Interleukin 1 receptor antagonist	0.017	1.566
INO80	INO80 complex subunit	0.000	-1.524
INPP4A	Inositol polyphosphate-4-phosphatase type I A	0.000	-1.501
IPMK	Inositol polyphosphate multikinase	0.000	-1.606
IQCA1	IQ motif containing with AAA domain 1	0.015	-1.564
IQCE	IQ motif containing E	0.001	-1.527
IQCG	IQ motif containing G	0.007	-1.683
IQGAP2	IQ motif containing gtpase activating protein 2	0.000	-1.580
ISM1	Isthmin 1, angiogenesis inhibitor	0.001	-1.766
ITGA4	Integrin subunit alpha 4	0.002	-1.710
ITPR1	Inositol 1,4,5-trisphosphate receptor type 1	0.000	-1.603
ITPR2	Inositol 1,4,5-trisphosphate receptor type 2	0.003	-1.638
ITSN2	Intersectin 2	0.000	-1.569
JCHAIN	Joining chain of multimeric iga and igm	0.013	3.465
JMJD1C	Jumonji domain containing 1C	0.000	-1.639
KAT6B	Lysine acetyltransferase 6B	0.000	-1.763
KCNK7	Potassium two pore domain channel subfamily K member 7	0.012	1.879
KCNRG	Potassium channel regulator	0.008	-2.120
KDELC2	KDEL motif containing 2	0.000	-1.577
KDM4B	Lysine demethylase 4B	0.000	-1.544
KDM5A	Lysine demethylase 5A	0.001	-1.575
KIAA0226L	KIAA0226 like	0.005	-1.736
KIAA0753	Kiaa0753	0.000	-1.511
KIAA1211	Kiaa1211	0.000	-2.327
KIAA1456	Kiaa1456	0.013	-1.670
KIAA1551	Kiaa1551	0.000	-1.581

KIAA2012	Kiaa2012	0.001	-2.276
KIDINS220	Kinase D-interacting substrate 220kda	0.000	-1.528
KIF13A	Kinesin family member 13A	0.000	-1.629
KIF20B	Kinesin family member 20B	0.006	-1.591
KIF21A	Kinesin family member 21A	0.001	-1.531
KIF27	Kinesin family member 27	0.000	-2.035
KIF3A	Kinesin family member 3A	0.000	-1.523
KLHL32	Kelch like family member 32	0.017	-1.546
KMT2C	Lysine methyltransferase 2C	0.002	-1.548
KMT2E	Lysine methyltransferase 2E	0.000	-1.583
KMT5A	Lysine methyltransferase 5A	0.000	-1.508
KRTCAP2	Keratinocyte associated protein 2	0.000	1.529
KRTCAP3	Keratinocyte associated protein 3	0.000	1.588
Ktn1	Kinectin 1	0.000	-1.532
LACC1	Laccase domain containing 1	0.000	-1.620
LAMA4	Laminin subunit alpha 4	0.000	-1.613
LAMB1	Laminin subunit beta 1	0.001	-1.617
LAMTOR2	Late endosomal/lysosomal adaptor, MAPK and MTOR activator 2	0.000	1.575
LCORL	Ligand dependent nuclear receptor corepressor like	0.000	-1.595
LDLRAD4	Low density lipoprotein receptor class A domain containing 4	0.001	-1.583
LECT1	Leukocyte cell derived chemotaxin 1	0.022	-2.147
LGALS1	Galectin 1	0.000	1.732
LGALS3	Lectin, galactoside binding soluble 3	0.000	1.636
LGALS4	Galectin 4	0.001	1.522
LIPT1	Lipoyltransferase 1	0.000	1.540
LLPH	LLP homolog, long-term synaptic facilitation	0.000	1.540
LOX	Lysyl oxidase	0.004	-1.578
LRP2	LDL receptor related protein 2	0.001	-2.052
LRP4	LDL receptor related protein 4	0.001	-1.534
LRRC43	Leucine rich repeat containing 43	0.029	-1.692
LRRCC1	Leucine rich repeat and coiled-coil centrosomal protein 1	0.000	-1.662
LRRIQ1	Leucine rich repeats and IQ motif containing 1	0.002	-2.105
LSM3	LSM3 homolog, U6 small nuclear RNA and mrna degradation associated	0.000	1.690
LSM8	LSM8 homolog, U6 small nuclear RNA associated	0.000	1.713
LTF	Lactotransferrin	0.031	1.709

LUM	Lumican	0.001	1.534
LY96	Lymphocyte antigen 96	0.000	1.635
LYST	Lysosomal trafficking regulator	0.001	-1.519
MACF1	Microtubule-actin crosslinking factor 1	0.000	-1.891
MAF	MAF bzip transcription factor	0.002	-1.543
MAGI1	Membrane associated guanylate kinase, WW and PDZ domain containing 1	0.001	-1.595
MAGI3	Membrane associated guanylate kinase, WW and PDZ domain containing 3	0.000	-1.561
MAGOH	Mago homolog, exon junction complex core component	0.000	1.780
MAK	Male germ cell associated kinase	0.033	-1.612
MAP3K13	Mitogen-activated protein kinase kinase kinase 13	0.000	-1.740
MAP3K14	Mitogen-activated protein kinase kinase kinase 14	0.003	-1.634
MAP4K4	Mitogen-activated protein kinase kinase kinase kinase 4	0.000	-1.497
MAP7D3	MAP7 domain containing 3	0.000	-1.789
MAP9	Microtubule associated protein 9	0.006	-1.647
MAST4	Microtubule associated serine/threonine kinase family member 4	0.009	-1.546
MAT1A	Methionine adenosyltransferase 1A	0.002	-1.961
MCEE	Methylmalonyl-coa epimerase	0.000	1.502
MCTP2	Multiple C2 and transmembrane domain containing 2	0.001	-1.571
MCTS1	MCTS1, re-initiation and release factor	0.000	1.553
MDN1	Midasin AAA atpase 1	0.000	-1.850
ME3	Malic enzyme 3	0.030	1.534
MED12L	Mediator complex subunit 12 like	0.019	-1.495
METTL12	Methyltransferase like 12	0.003	1.654
MFSD4A	Major facilitator superfamily domain containing 4A	0.008	-1.600
MGA	MGA, MAX dimerization protein	0.001	-1.680
MGAT4C	MGAT4 family member C	0.010	-1.630
MGST3	Microsomal glutathione S-transferase 3	0.000	1.656
MICAL3	Microtubule associated monooxygenase, calponin and LIM domain containing 3	0.002	-1.512
MKI67	Marker of proliferation Ki-67	0.001	-3.003
MMP2	Matrix metallopeptidase 2	0.000	-1.516
MNS1	Meiosis specific nuclear structural 1	0.026	-1.569
MPDZ	Multiple PDZ domain crumbs cell polarity complex component	0.000	-1.593
MPHOSPH9	M-phase phosphoprotein 9	0.000	-1.665
MRI1	Methylthioribose-1-phosphate isomerase 1	0.001	1.500
MRPL10	Mitochondrial ribosomal protein L10	0.000	1.497

MRPL3	Mitochondrial ribosomal protein L3	0.000	1.697
MRPL30	Mitochondrial ribosomal protein L30	0.000	1.522
MRPL32	Mitochondrial ribosomal protein L32	0.000	1.606
MRPL34	Mitochondrial ribosomal protein L34	0.000	1.532
MRPL42	Mitochondrial ribosomal protein L42	0.000	1.597
MRPL48	Mitochondrial ribosomal protein L48	0.000	1.539
MRPS14	Mitochondrial ribosomal protein S14	0.000	1.609
MS4A8	Membrane spanning 4-domains A8	0.003	1.895
MSI2	Musashi RNA binding protein 2	0.001	-1.620
MT-ATP6	ATP synthase F0 subunit 6	0.000	2.003
MT-CO1	Cytochrome c oxidase subunit I	0.000	1.841
MT-CO2	Cytochrome c oxidase subunit II	0.000	2.095
MT-CO3	Cytochrome c oxidase III	0.000	2.072
MT-CYB	Cytochrome b	0.001	1.929
MTHFD2	Methylenetetrahydrofolate dehydrogenase (NADP+ dependent) 2, methenyltetrahydrofolate cyclohydrolase	0.002	1.501
MT-ND1	NADH dehydrogenase, subunit 1 (complex I)	0.000	2.071
MT-ND2	Mtnd2	0.004	1.818
MT-ND3	NADH dehydrogenase, subunit 3 (complex I)	0.000	2.200
MT-ND4	NADH dehydrogenase, subunit 4 (complex I)	0.004	1.786
MT-ND4L	NADH dehydrogenase, subunit 4L (complex I)	0.001	1.988
MT-ND5	NADH dehydrogenase, subunit 5 (complex I)	0.021	1.584
MTR	5-methyltetrahydrofolate-homocysteine methyltransferase	0.000	-1.735
MYB	MYB proto-oncogene, transcription factor	0.033	-1.588
MYCBP2	MYC binding protein 2, E3 ubiquitin protein ligase	0.000	-1.756
MYH10	Myosin, heavy chain 10, non-muscle	0.001	-1.580
MYL3	Myosin light chain 3	0.006	1.828
MYL6	Myosin light chain 6	0.000	1.591
MYO9A	Myosin IXA	0.002	-1.639
MYOZ1	Myozenin 1	0.000	1.841
MZF1	Myeloid zinc finger 1	0.001	-1.557
N4BP2	NEDD4 binding protein 2	0.009	-1.517
NAPB	NSF attachment protein beta	0.003	-1.535
NAV3	Neuron navigator 3	0.005	-1.714
NBEAL1	Neurobeachin like 1	0.017	-1.762

NDUFA1	NADH:ubiquinone oxidoreductase subunit A1	0.000	1.551
NDUFA12	NADH:ubiquinone oxidoreductase subunit A12	0.000	1.845
NDUFA13	NADH:ubiquinone oxidoreductase subunit A13	0.000	1.582
NDUFA4	NDUFA4, mitochondrial complex associated	0.000	1.696
NDUFA5	NADH:ubiquinone oxidoreductase subunit A5	0.000	1.543
NDUFA6	NADH:ubiquinone oxidoreductase subunit A6	0.000	1.571
NDUFB11	NADH:ubiquinone oxidoreductase subunit B11	0.000	1.568
NDUFB3	NADH:ubiquinone oxidoreductase subunit B3	0.000	1.741
NDUFB4	NADH:ubiquinone oxidoreductase subunit B4	0.000	1.565
NDUFB6	NADH:ubiquinone oxidoreductase subunit B6	0.000	1.692
NDUFB9	NADH:ubiquinone oxidoreductase subunit B9	0.000	1.557
NDUFC1	NADH:ubiquinone oxidoreductase subunit C1	0.000	1.679
NDUFC2	NADH:ubiquinone oxidoreductase subunit C2	0.000	1.585
NEK5	NIMA related kinase 5	0.011	-1.903
NETO2	Neuropilin and tolloid like 2	0.019	1.654
NEURL4	Neuralized E3 ubiquitin protein ligase 4	0.000	-1.516
NFKBIZ	NFKB inhibitor zeta	0.000	-2.123
NHS	NHS actin remodeling regulator	0.006	-1.535
NIN	Ninein	0.000	-1.984
NIPBL	NIPBL, cohesin loading factor	0.000	-1.679
NKTR	Natural killer cell triggering receptor	0.000	-1.892
NMB	Neuromedin B	0.018	1.598
NOL3	Nucleolar protein 3	0.001	1.517
NOTCH4	Notch 4	0.014	-1.499
NPAT	Nuclear protein, coactivator of histone transcription	0.000	-1.782
NPM1	Nucleophosmin	0.000	1.706
NPM3	Nucleophosmin/nucleoplasmin 3	0.001	1.505
NQO2	NAD(P)H quinone dehydrogenase 2	0.001	1.522
NR2C2	Nuclear receptor subfamily 2 group C member 2	0.000	-1.516
NR5A2	Nuclear receptor subfamily 5 group A member 2	0.019	-1.582
NRIP1	Nuclear receptor interacting protein 1	0.000	-1.644
NSMCE1	NSE1 homolog, SMC5-SMC6 complex component	0.000	1.614
NTN4	Netrin 4	0.000	-1.772
NUP93	Nucleoporin 93	0.006	1.590
NWD1	NACHT and WD repeat domain containing 1	0.008	-2.822

ODF2L	Outer dense fiber of sperm tails 2 like	0.000	-1.572
OLFML2B	Olfactomedin like 2B	0.000	-1.740
OOEP	Oocyte expressed protein	0.012	1.633
ORMDL2	ORMDL sphingolipid biosynthesis regulator 2	0.000	1.609
OSBPL8	Oxysterol binding protein like 8	0.000	-1.730
OSTC	Oligosaccharyltransferase complex non-catalytic subunit	0.000	1.509
PAFAH1B3	Platelet activating factor acetylhydrolase 1b catalytic subunit 3	0.000	1.608
PAPPA	Pappalysin 1	0.001	-2.208
PAXBP1	PAX3 and PAX7 binding protein 1	0.000	-1.664
PCF11	PCF11 cleavage and polyadenylation factor subunit	0.000	-1.700
PCM1	Pericentriolar material 1	0.000	-1.699
PDE3B	Phosphodiesterase 3B	0.002	-1.554
PDE4DIP	Phosphodiesterase 4D interacting protein	0.000	-1.600
PDE6D	Phosphodiesterase 6D	0.000	1.639
PDZD2	PDZ domain containing 2	0.001	-1.673
PER1	Period circadian clock 1	0.001	-1.514
PFDN1	Prefoldin subunit 1	0.000	1.557
PFDN4	Prefoldin subunit 4	0.000	1.617
PFDN5	Prefoldin subunit 5	0.000	1.834
PFDN6	Prefoldin subunit 6	0.000	1.528
PFN2	Profilin 2	0.000	1.506
PGM2	Phosphoglucomutase 2	0.000	-1.999
PHF20L1	PHD finger protein 20-like 1	0.000	-1.558
PHF3	PHD finger protein 3	0.000	-1.730
PHIP	Pleckstrin homology domain interacting protein	0.003	-1.866
PIEZO2	Piezo type mechanosensitive ion channel component 2	0.030	-1.528
PIGP	Phosphatidylinositol glycan anchor biosynthesis class P	0.000	1.757
PIK3C2A	Phosphatidylinositol-4-phosphate 3-kinase catalytic subunit type 2 alpha	0.030	-1.558
PIN4	Peptidylprolyl cis/trans isomerase, NIMA-interacting 4	0.000	1.504
PKIB	Protein kinase (camp-dependent, catalytic) inhibitor beta	0.003	-1.551
PLA2R1	Phospholipase A2 receptor 1	0.007	-1.630
PLCB1	Phospholipase C beta 1	0.012	-1.505
PLCB4	Phospholipase C beta 4	0.002	-1.501
PLCG1	Phospholipase C gamma 1	0.000	-1.529
PLEKHD1	Pleckstrin homology and coiled-coil domain containing D1	0.020	-1.746

PLEKHG1	Pleckstrin homology and rhogef domain containing G1	0.001	-1.681
PLGRKT	Plasminogen receptor with a C-terminal lysine	0.000	1.517
PLK1	Polo like kinase 1	0.001	-1.652
PLOD2	Procollagen-lysine,2-oxoglutarate 5-dioxygenase 2	0.004	-1.638
PLP2	Proteolipid protein 2	0.000	1.683
PLXNC1	Plexin C1	0.001	-1.559
PODN	Podocan	0.000	-1.641
POLR2H	RNA polymerase II subunit H	0.000	1.502
POLR2K	RNA polymerase II subunit K	0.000	1.595
POP5	POP5 homolog, ribonuclease P/MRP subunit	0.000	1.645
POP7	POP7 homolog, ribonuclease P/MRP subunit	0.000	1.530
PPA1	Pyrophosphatase (inorganic) 1	0.016	1.675
PPIL3	Peptidylprolyl isomerase like 3	0.000	1.666
PQLC3	PQ loop repeat containing 3	0.017	1.564
PRDX1	Peroxiredoxin 1	0.000	1.602
PREX2	Phosphatidylinositol-3,4,5-trisphosphate dependent Rac exchange factor 2	0.017	-1.655
PRICKLE2	Prickle planar cell polarity protein 2	0.020	-1.538
Prorsd1	Prolyl-trna synthetase domain containing 1	0.002	1.530
PRSS23	Protease, serine 23	0.009	1.520
PRSS35	Protease, serine 35	0.001	-2.594
PRX	Periaxin	0.003	-1.853
PSD3	Pleckstrin and Sec7 domain containing 3	0.008	-1.567
PSMA1	Proteasome subunit alpha 1	0.000	1.505
PSMA2	Proteasome subunit alpha 2	0.000	1.609
PSMA3	Proteasome subunit alpha 3	0.000	1.567
PSMA6	Proteasome subunit alpha 6	0.000	1.594
PSMB1	Proteasome subunit beta 1	0.000	1.597
PSMB3	Proteasome subunit beta 3	0.000	1.634
PSMB4	Proteasome subunit beta 4	0.000	1.670
PSMB6	Proteasome subunit beta 6	0.000	1.577
PSMB7	Proteasome subunit beta 7	0.000	1.506
PSMB8	Proteasome subunit beta 8	0.002	1.513
PSMB9	Proteasome subunit beta 9	0.008	1.509
PSME2	Proteasome activator subunit 2	0.000	1.606
PSMG2	Proteasome assembly chaperone 2	0.000	1.612

PSMG4	Proteasome assembly chaperone 4	0.000	1.833
PSPH	Phosphoserine phosphatase	0.025	1.996
PTCH2	Patched 2	0.004	-1.692
PTPN11	Protein tyrosine phosphatase, non-receptor type 11	0.000	-1.534
PTPN13	Protein tyrosine phosphatase, non-receptor type 13	0.000	-1.733
PTPRD	Protein tyrosine phosphatase, receptor type D	0.003	-1.638
RAB11FIP2	RAB11 family interacting protein 2	0.000	-1.509
RAB38	RAB38, member RAS oncogene family	0.019	1.539
RABEP1	Rabaptin, RAB gtpase binding effector protein 1	0.001	-1.621
RACK1	Receptor for activated C kinase 1	0.000	1.940
RAD51B	RAD51 paralog B	0.000	1.522
RAPGEF4	Rap guanine nucleotide exchange factor 4	0.007	-1.672
RASGRF2	Ras protein specific guanine nucleotide releasing factor 2	0.002	-1.580
RASGRP1	RAS guanyl releasing protein 1	0.001	-1.741
RBBP6	RB binding protein 6, ubiquitin ligase	0.001	-1.555
RBM3	RNA binding motif (RNP1, RRM) protein 3	0.000	1.842
RBM5	RNA binding motif protein 5	0.000	-1.525
REV3L	REV3 like, DNA directed polymerase zeta catalytic subunit	0.000	-1.796
RFC4	Replication factor C subunit 4	0.000	1.593
RFX7	Regulatory factor X7	0.000	-1.608
RGS6	Regulator of G-protein signaling 6	0.008	-1.643
RHBG	Rh family B glycoprotein (gene/pseudogene)	0.027	1.780
RHEB	Ras homolog enriched in brain	0.000	1.516
RICTOR	RPTOR independent companion of MTOR complex 2	0.000	-1.580
RIF1	Replication timing regulatory factor 1	0.000	-1.741
RNF168	Ring finger protein 168	0.000	-1.603
RNF6	Ring finger protein 6	0.000	-1.557
ROBO2	Roundabout guidance receptor 2	0.014	-1.513
ROCK1	Rho associated coiled-coil containing protein kinase 1	0.000	-1.593
ROCK2	Rho associated coiled-coil containing protein kinase 2	0.000	-1.633
ROMO1	Reactive oxygen species modulator 1	0.000	1.577
RPGRIP1L	RPGRIP1 like	0.000	-1.820
RPL10	Ribosomal protein L10	0.000	1.692
RPL10A	Ribosomal protein l10a	0.000	1.769
RPL11	Ribosomal protein L11	0.000	1.696

RPL13	Ribosomal protein L13	0.000	1.685
RPL13A	Ribosomal protein l13a	0.000	1.825
RPL14	Ribosomal protein L14	0.000	1.614
RPL15	Ribosomal protein L15	0.000	1.596
RPL17	Ribosomal protein L17	0.000	1.908
RPL18	Ribosomal protein L18	0.000	1.583
RPL18A	Ribosomal protein l18a	0.000	1.605
RPL21	Ribosomal protein L21	0.000	1.904
RPL22	Ribosomal protein L22	0.000	1.864
RPL23	Ribosomal protein L23	0.000	2.062
RPL24	Ribosomal protein L24	0.000	1.667
RPL26	Ribosomal protein L26	0.000	2.021
RPL27	Ribosomal protein L27	0.000	2.107
RPL27A	Ribosomal protein l27a	0.000	1.879
RPL29	Ribosomal protein L29	0.000	1.678
RPL3	Ribosomal protein L3	0.000	1.669
RPL30	Ribosomal protein L30	0.000	2.070
RPL31	Ribosomal protein L31	0.000	1.961
RPL32	Ribosomal protein L32	0.000	2.054
RPL35	Ribosomal protein L35	0.000	1.719
RPL35A	Ribosomal protein l35a	0.000	2.138
RPL36	Ribosomal protein L36	0.000	1.733
RPL36A	Ribosomal protein l36a	0.000	2.512
RPL37	Ribosomal protein L37	0.000	2.276
RPL37A	Ribosomal protein l37a	0.000	2.032
RPL38	Ribosomal protein L38	0.000	1.712
RPL5	Ribosomal protein L5	0.000	1.719
RPL6	Ribosomal protein L6	0.000	1.548
RPL7	Ribosomal protein L7	0.000	1.592
RPL7A	Ribosomal protein l7a	0.000	1.670
RPL8	Ribosomal protein L8	0.000	1.764
RPLP0	Ribosomal protein lateral stalk subunit P0	0.000	1.918
RPLP1	Ribosomal protein lateral stalk subunit P1	0.000	2.040
RPLP2	Ribosomal protein lateral stalk subunit P2	0.000	2.069
RPS10	Ribosomal protein S10	0.000	1.828

RPS11	Ribosomal protein S11	0.000	1.763
RPS12	Ribosomal protein S12	0.000	1.938
RPS13	Ribosomal protein S13	0.000	1.846
RPS14	Ribosomal protein S14	0.000	1.856
RPS15	Ribosomal protein S15	0.000	1.691
RPS16	Ribosomal protein S16	0.000	1.632
RPS17	Ribosomal protein S17	0.000	2.018
RPS18	Ribosomal protein S18	0.000	1.845
RPS19	Ribosomal protein S19	0.000	2.044
RPS2	Ribosomal protein S2	0.000	1.645
RPS20	Ribosomal protein S20	0.000	2.115
RPS21	Ribosomal protein S21	0.000	2.354
RPS23	Ribosomal protein S23	0.000	1.727
RPS24	Ribosomal protein S24	0.000	2.100
RPS25	Ribosomal protein S25	0.000	2.389
RPS26	Ribosomal protein S26	0.000	2.115
RPS27	Ribosomal protein S27	0.000	2.089
RPS27A	Ribosomal protein s27a	0.000	1.916
RPS28	Ribosomal protein S28	0.000	1.739
RPS3	Ribosomal protein S3	0.000	2.035
RPS3A	Ribosomal protein S3A	0.000	1.713
RPS4X	Ribosomal protein S4, X-linked	0.000	1.748
RPS5	Ribosomal protein S5	0.000	1.814
RPS7	Ribosomal protein S7	0.000	1.675
RPS8	Ribosomal protein S8	0.000	1.664
RPS9	Ribosomal protein S9	0.000	1.596
RPSA	Ribosomal protein SA	0.000	1.725
RSF1	Remodeling and spacing factor 1	0.000	-1.800
RYR2	Ryanodine receptor 2	0.021	-1.613
S100A13	S100 calcium binding protein A13	0.000	1.599
S100A2	S100 calcium binding protein A2	0.002	1.839
SACS	Sacsin molecular chaperone	0.006	-1.592
SAMD15	Sterile alpha motif domain containing 15	0.012	-1.769
SATB1	SATB homeobox 1	0.001	-1.634
SCAF11	SR-related CTD associated factor 11	0.000	-1.631

SCAPER	S-phase cyclin A associated protein in the ER	0.000	-1.655
SCARA5	Scavenger receptor class A member 5	0.000	-1.532
SDHD	Succinate dehydrogenase complex subunit D	0.000	1.521
SEC11A	SEC11 homolog A, signal peptidase complex subunit	0.000	1.599
SEC11C	SEC11 homolog C, signal peptidase complex subunit	0.000	1.675
SEC14L3	SEC14 like lipid binding 3	0.009	-3.055
SEC61B	Sec61 translocon beta subunit	0.000	1.568
SELK	Selenoprotein K	0.000	1.555
SEMA3C	Semaphorin 3C	0.000	-1.623
SENP6	SUMO1/sentrin specific peptidase 6	0.000	-1.647
SERGEF	Secretion regulating guanine nucleotide exchange factor	0.000	1.564
SERPINB8	Serpin family B member 8	0.010	1.557
SETBP1	SET binding protein 1	0.002	-1.644
SETD2	SET domain containing 2	0.000	-1.643
SETD5	SET domain containing 5	0.000	-1.612
SETD9	SET domain containing 9	0.000	1.615
SETX	Senataxin	0.000	-1.890
SFR1	SWI5 dependent homologous recombination repair protein 1	0.002	1.514
SFXN4	Sideroflexin 4	0.000	1.503
SH3PXD2A	SH3 and PX domains 2A	0.002	-1.501
SH3PXD2B	SH3 and PX domains 2B	0.000	-1.628
SHANK2	SH3 and multiple ankyrin repeat domains 2	0.007	-1.634
SLC1A5	Solute carrier family 1 member 5	0.000	1.754
SLC25A5	Solute carrier family 25 member 5	0.009	1.564
SLC38A5	Solute carrier family 38 member 5	0.005	1.631
SLC39A2	Solute carrier family 39 member 2	0.015	2.241
SLC45A2	Solute carrier family 45 member 2	0.000	2.535
SLC50A1	Solute carrier family 50 member 1	0.000	1.511
SLCO1A2	Solute carrier organic anion transporter family member 1A2	0.014	-1.660
SLIRP	SRA stem-loop interacting RNA binding protein	0.000	1.761
SLIT3	Slit guidance ligand 3	0.000	-1.505
SLK	STE20 like kinase	0.002	-1.501
SLTM	SAFB like transcription modulator	0.000	-1.502
SMC1A	Structural maintenance of chromosomes 1A	0.000	-1.585
SMC2	Structural maintenance of chromosomes 2	0.000	-1.634

SMC4	Structural maintenance of chromosomes 4	0.000	-1.928
SMC5	Structural maintenance of chromosomes 5	0.000	-1.618
SMC6	Structural maintenance of chromosomes 6	0.000	-1.589
SMG1	SMG1, nonsense mediated mrna decay associated PI3K related kinase	0.000	-1.982
SMIM20	Small integral membrane protein 20	0.000	1.502
SMIM8	Small integral membrane protein 8	0.001	1.531
SMPDL3A	Sphingomyelin phosphodiesterase acid like 3A	0.016	1.587
SNRPB2	Small nuclear ribonucleoprotein polypeptide B2	0.000	1.509
SNRPD2	Small nuclear ribonucleoprotein D2 polypeptide	0.000	1.573
SNRPF	Small nuclear ribonucleoprotein polypeptide F	0.000	1.615
SNRPG	Small nuclear ribonucleoprotein polypeptide G	0.000	2.145
SOAT1	Sterol O-acyltransferase 1	0.007	1.808
SORBS1	Sorbin and SH3 domain containing 1	0.000	-1.533
SOX6	SRY-box 6	0.027	-1.594
SPAG9	Sperm associated antigen 9	0.000	-1.504
SPATA18	Spermatogenesis associated 18	0.022	-1.647
SPECC1L	Sperm antigen with calponin homology and coiled-coil domains 1 like	0.000	-1.495
SPEF2	Sperm flagellar 2	0.003	-2.020
SPEG	SPEG complex locus	0.018	-1.581
SPEN	Spen family transcriptional repressor	0.002	-1.668
SPP1	Secreted phosphoprotein 1	0.004	-2.428
SPTBN1	Spectrin beta, non-erythrocytic 1	0.001	-1.517
SRGAP3	SLIT-ROBO Rho gtpase activating protein 3	0.008	-1.747
SRP14	Signal recognition particle 14	0.000	1.535
SRPK2	SRSF protein kinase 2	0.000	-1.521
SRPRB	SRP receptor beta subunit	0.000	1.508
SSR2	Signal sequence receptor subunit 2	0.000	1.724
SSR4	Signal sequence receptor subunit 4	0.000	1.733
SSUH2	Ssu-2 homolog (C. Elegans)	0.003	-2.878
STAB1	Stabilin 1	0.005	-1.623
STARD13	Star related lipid transfer domain containing 13	0.000	-1.496
STK31	Serine/threonine kinase 31	0.011	-1.622
STK33	Serine/threonine kinase 33	0.027	-1.606
STRADB	STE20-related kinase adaptor beta	0.000	1.508
SULF1	Sulfatase 1	0.000	-1.784

SULF2	Sulfatase 2	0.000	-1.505
SYNE2	Spectrin repeat containing nuclear envelope protein 2	0.002	-1.607
SZT2	Seizure threshold 2 homolog (mouse)	0.003	-1.610
TAF1	TATA-box binding protein associated factor 1	0.000	-1.641
TANC1	Tetratricopeptide repeat, ankyrin repeat and coiled-coil containing 1	0.000	-1.537
TAOK2	TAO kinase 2	0.000	-1.512
TATDN1	Tatd dnase domain containing 1	0.000	2.053
TAX1BP1	Tax1 binding protein 1	0.000	-1.497
TBC1D16	TBC1 domain family member 16	0.001	-1.628
TCAIM	T-cell activation inhibitor, mitochondrial	0.030	-1.551
TCF20	Transcription factor 20	0.000	-1.694
TEKT3	Tektin 3	0.012	-1.616
TENM3	Teneurin transmembrane protein 3	0.000	-1.980
TET2	Tet methylcytosine dioxygenase 2	0.001	-1.795
THEM4	Thioesterase superfamily member 4	0.001	1.512
TIA1	TIA1 cytotoxic granule-associated RNA binding protein	0.000	-1.567
TM4SF1	Transmembrane 4 L six family member 1	0.021	1.507
TM6SF2	Transmembrane 6 superfamily member 2	0.004	1.720
TMEM205	Transmembrane protein 205	0.000	1.673
TMEM208	Transmembrane protein 208	0.000	1.714
TMEM232	Transmembrane protein 232	0.010	-1.505
TMEM256	Transmembrane protein 256	0.000	1.694
TMEM40	Transmembrane protein 40	0.009	2.873
TMEM60	Transmembrane protein 60	0.000	1.633
TMEM64	Transmembrane protein 64	0.005	1.571
TMF1	TATA element modulatory factor 1	0.000	-1.624
TNFRSF4	TNF receptor superfamily member 4	0.018	-1.783
TNMD	Tenomodulin	0.005	1.530
TNR	Tenascin R	0.002	-3.009
TNRC6A	Trinucleotide repeat containing 6A	0.000	-1.759
TNRC6C	Trinucleotide repeat containing 6C	0.001	-1.665
TOMM7	Translocase of outer mitochondrial membrane 7	0.000	1.796
TOP2A	Topoisomerase (DNA) II alpha	0.003	-1.991
TP53BP1	Tumor protein p53 binding protein 1	0.000	-1.516
TPCN1	Two pore segment channel 1	0.019	-1.621

TPT1	Tumor protein, translationally-controlled 1	0.000	1.866
TRAF3	TNF receptor associated factor 3	0.001	-1.515
TRAF3IP1	TRAF3 interacting protein 1	0.000	-1.585
TRIM44	Tripartite motif containing 44	0.000	-1.532
TRIP11	Thyroid hormone receptor interactor 11	0.000	-2.162
TRMT112	Trna methyltransferase 11-2 homolog (S. Cerevisiae)	0.000	1.621
TRMT44	Trna methyltransferase 44 homolog (S. Cerevisiae)	0.001	-1.573
TRPC1	Transient receptor potential cation channel subfamily C member 1	0.000	-1.582
TRPM6	Transient receptor potential cation channel subfamily M member 6	0.012	-1.582
TRPS1	Transcriptional repressor GATA binding 1	0.002	-1.600
TSC1	Tuberous sclerosis 1	0.000	-1.746
TSGA10	Testis specific 10	0.004	-1.656
TSHZ2	Teashirt zinc finger homeobox 2	0.001	-1.553
TSPAN6	Tetraspanin 6	0.001	1.528
TSPO	Translocator protein	0.000	1.563
TTBK2	Tau tubulin kinase 2	0.004	-1.601
TTC21A	Tetratricopeptide repeat domain 21A	0.024	-1.649
TTC3	Tetratricopeptide repeat domain 3	0.000	-1.528
TTLL7	Tubulin tyrosine ligase like 7	0.001	-1.910
TXNDC12	Thioredoxin domain containing 12	0.000	1.518
TXNDC16	Thioredoxin domain containing 16	0.002	-1.599
UACA	Uveal autoantigen with coiled-coil domains and ankyrin repeats	0.000	-1.785
UBA52	Ubiquitin A-52 residue ribosomal protein fusion product 1	0.000	1.618
UBL5	Ubiquitin like 5	0.000	1.694
UBR2	Ubiquitin protein ligase E3 component n-recognin 2	0.000	-1.561
UBR5	Ubiquitin protein ligase E3 component n-recognin 5	0.000	-1.518
UCHL3	Ubiquitin C-terminal hydrolase L3	0.004	1.507
UGGT2	UDP-glucose glycoprotein glucosyltransferase 2	0.002	-1.539
UHRF1BP1	UHRF1 binding protein 1	0.001	-1.504
UHRF1BP1L	UHRF1 binding protein 1 like	0.000	-1.496
UNC45B	Unc-45 myosin chaperone B	0.033	1.731
UQCR10	Ubiquinol-cytochrome c reductase, complex III subunit X	0.000	1.537
UQCR11	Ubiquinol-cytochrome c reductase, complex III subunit XI	0.000	1.579
UQCRH	Ubiquinol-cytochrome c reductase hinge protein	0.000	1.754
USP34	Ubiquitin specific peptidase 34	0.000	-1.519

UTRN	Utrophin	0.000	-1.736
UXT	Ubiquitously expressed prefoldin like chaperone	0.000	1.693
VPS13D	Vacuolar protein sorting 13 homolog D	0.000	-1.528
VPS29	VPS29, retromer complex component	0.000	1.504
VWA3A	Von Willebrand factor A domain containing 3A	0.032	-1.715
VWA3B	Von Willebrand factor A domain containing 3B	0.022	-1.729
WDFY3	WD repeat and FYVE domain containing 3	0.000	-1.582
WDR78	WD repeat domain 78	0.023	-1.622
WHSC1	Wolf-Hirschhorn syndrome candidate 1	0.001	-1.593
WNT11	Wnt family member 11	0.020	-1.523
WRN	Werner syndrome recq like helicase	0.000	-1.516
WWC2	WW and C2 domain containing 2	0.000	-1.616
WWC3	WWC family member 3	0.000	-1.589
XRN1	5'-3' exoribonuclease 1	0.008	-1.592
YEATS2	YEATS domain containing 2	0.000	-1.610
ZAK	Sterile alpha motif and leucine zipper containing kinase AZK	0.000	-1.593
ZBED6	Zinc finger BED-type containing 6	0.032	-1.914
ZC3H11A	Zinc finger CCCH-type containing 11A	0.000	-1.597
ZC3H12A	Zinc finger CCCH-type containing 12A	0.012	-1.614
ZC3H12C	Zinc finger CCCH-type containing 12C	0.006	-1.624
ZC3H6	Zinc finger CCCH-type containing 6	0.000	-1.586
ZCCHC6	Zinc finger CCHC-type containing 6	0.000	-1.509
ZEB1	Zinc finger E-box binding homeobox 1	0.000	-1.661
ZFP62	ZFP62 zinc finger protein	0.002	-1.507
ZFYVE16	Zinc finger FYVE-type containing 16	0.001	-1.635
ZHX1	Zinc fingers and homeoboxes 1	0.000	-1.643
ZKSCAN1	Zinc finger with KRAB and SCAN domains 1	0.000	-1.843
ZMYND8	Zinc finger MYND-type containing 8	0.001	-1.529
ZNF182	Zinc finger protein 182	0.000	-1.568
ZNF197	Zinc finger protein 197	0.000	-1.625
ZNF202	Zinc finger protein 202	0.000	-1.596
ZNF214	Zinc finger protein 214	0.002	-1.700
ZNF235	Zinc finger protein 235	0.001	-1.528
ZNF236	Zinc finger protein 236	0.001	-1.605
ZNF292	Zinc finger protein 292	0.000	-1.737

ZNF318	Zinc finger protein 318	0.000	-1.763
ZNF41	Zinc finger protein 41	0.001	-1.536
ZNF507	Zinc finger protein 507	0.000	-1.549
ZNF605	Zinc finger protein 605	0.000	-1.874
ZNF608	Zinc finger protein 608	0.000	-1.676
ZNF638	Zinc finger protein 638	0.000	-1.568
ZNF644	Zinc finger protein 644	0.000	-1.588
ZNF677	Zinc finger protein 677	0.000	-1.529
ZSCAN29	Zinc finger and SCAN domain containing 29	0.000	-1.597

[1] Fold changes are up or down in PUFA, pregnant, low diet compared with PUFA, pregnant, High diet

Table S8. Differentially expressed genes that respond differently to diet dependent on PUFA supplementation in pregnant animals

Symbol	Entrez Gene Name	p-value	Fold Change[1]
A2M	Alpha-2-macroglobulin	0.0188	-1.722
AAMDC	Adipogenesis associated Mth938 domain containing	0.00213	1.598
AASS	Aminoadipate-semialdehyde synthase	0.00305	-1.53
ABAT	4-aminobutyrate aminotransferase	0.00822	-1.836
ABCA2	ATP binding cassette subfamily A member 2	0.0213	-1.589
ABCA9	ATP binding cassette subfamily A member 9	0.00601	-1.896
ABCB1	ATP binding cassette subfamily B member 1	0.00103	-1.971
ABCC10	ATP binding cassette subfamily C member 10	0.000866	-1.989
ABCG1	ATP binding cassette subfamily G member 1	0.0236	-1.682
ABHD10	Abhydrolase domain containing 10	0.000132	1.623
ABHD11	Abhydrolase domain containing 11	0.000519	1.753
ABL2	ABL proto-oncogene 2, non-receptor tyrosine kinase	0.0239	-1.561
ACACA	Acetyl-coa carboxylase alpha	0.000281	-1.68
ACAD10	Acyl-coa dehydrogenase family member 10	0.000361	-1.626
Acan	Aggrecan	0.00272	-2.901
ACAP3	Arfgap with coiled-coil, ankyrin repeat and PH domains 3	0.0101	-1.512
ACIN1	Apoptotic chromatin condensation inducer 1	0.000122	-1.81
ACKR1	Atypical chemokine receptor 1 (Duffy blood group)	0.000393	2.638
ACOT9	Acyl-coa thioesterase 9	0.000083	1.636
ACOX3	Acyl-coa oxidase 3, pristanoyl	0.00859	-1.592
ACTL6A	Actin like 6A	0.000152	1.575
ACTR3B	ARP3 actin related protein 3 homolog B	0.0207	-1.531
ACVR1B	Activin A receptor type 1B	0.00505	-1.61
ACYP1	Acylphosphatase 1	0.00361	1.564
ADAM19	ADAM metallopeptidase domain 19	0.00514	-1.69
ADAMTS2	ADAM metallopeptidase with thrombospondin type 1 motif 2	0.00499	-1.733
ADAMTSL1	ADAMTS like 1	0.00828	-1.865

ADAP2	Arfgap with dual PH domains 2	0.00776	-1.717
ADCY5	Adenylate cyclase 5	0.0117	-1.665
ADCY6	Adenylate cyclase 6	7.54E-05	-1.635
ADCY9	Adenylate cyclase 9	0.0191	-1.54
ADGB	Androglobin	0.0232	-2.164
ADGRF5	Adhesion G protein-coupled receptor F5	0.00228	-1.645
ADGRL3	Adhesion G protein-coupled receptor L3	0.00836	-2.19
ADH5	Alcohol dehydrogenase 5 (class III), chi polypeptide	0.000649	1.6
ADIRF	Adipogenesis regulatory factor	0.00131	1.935
ADM	Adrenomedullin	0.0108	1.613
AFAP1L2	Actin filament associated protein 1 like 2	0.001	-1.607
AFDN	Afadin, adherens junction formation factor	0.0062	-1.527
AFF3	AF4/FMR2 family member 3	0.00547	-1.558
AFF4	AF4/FMR2 family member 4	0.0111	-1.715
AFMID	Arylformamidase	0.0206	-2.03
AGAP3	Arfgap with gtpase domain, ankyrin repeat and PH domain 3	0.00218	-1.497
AGGF1	Angiogenic factor with G-patch and FHA domains 1	0.000505	-1.528
AGTPBP1	ATP/GTP binding protein 1	0.0113	-1.564
AIM1	Absent in melanoma 1	0.00336	-1.628
AIM1L	Absent in melanoma 1-like	0.00715	-2.684
AK2	Adenylate kinase 2	0.00578	1.639
AK9	Adenylate kinase 9	0.0221	-2.154
AKAP12	A-kinase anchoring protein 12	0.00133	-1.986
AKAP4	A-kinase anchoring protein 4	0.00368	3.685
AKAP8	A-kinase anchoring protein 8	0.000102	-1.728
AKAP9	A-kinase anchoring protein 9	0.00188	-1.884
AKNA	AT-hook transcription factor	0.0238	-1.556
AKR1A1	Aldo-keto reductase family 1 member A1	0.000332	1.996
ALG5	ALG5, dolichyl-phosphate beta-glucosyltransferase	6.74E-06	1.908
ALKBH7	Alkb homolog 7	0.0065	1.532

ALMS1	ALMS1, centrosome and basal body associated protein	0.000461	-2.045
ALOX5AP	Arachidonate 5-lipoxygenase activating protein	0.0237	2.103
ALPK1	Alpha kinase 1	0.000667	-1.675
ALPK3	Alpha kinase 3	0.0115	-2.178
ALS2CL	ALS2 C-terminal like	0.00189	-1.66
AMBRA1	Autophagy and beclin 1 regulator 1	0.000349	-1.54
AMOTL2	Angiomotin like 2	0.00457	-1.551
ANAPC10	Anaphase promoting complex subunit 10	0.00944	1.508
ANKRD11	Ankyrin repeat domain 11	0.000466	-2
ANKRD12	Ankyrin repeat domain 12	7.03E-05	-2.089
ANKRD17	Ankyrin repeat domain 17	9.99E-05	-1.671
ANKRD22	Ankyrin repeat domain 22	0.0314	1.59
ANKRD26	Ankyrin repeat domain 26	1.29E-05	-2.656
ANKRD28	Ankyrin repeat domain 28	0.00272	-1.523
ANKRD6	Ankyrin repeat domain 6	0.000538	-1.918
ANKS1A	Ankyrin repeat and sterile alpha motif domain containing 1A	0.000317	-1.648
ANO8	Anoctamin 8	0.00867	-1.644
ANXA1	Annexin A1	0.0161	1.936
AP1S2	Adaptor related protein complex 1 sigma 2 subunit	0.00806	1.529
APBA1	Amyloid beta precursor protein binding family A member 1	0.00378	-2.026
APBB3	Amyloid beta precursor protein binding family B member 3	0.012	-1.685
APC	APC, WNT signaling pathway regulator	3.46E-06	-2.5
APEX1	Apurinic/apyrimidinic endodeoxyribonuclease 1	0.000394	1.649
APIP	APAF1 interacting protein	0.000113	1.629
APOM	Apolipoprotein M	0.00429	1.57
APRT	Adenine phosphoribosyltransferase	0.00704	1.646
ARAP2	Arfgap with rhogap domain, ankyrin repeat and PH domain 2	0.000609	-1.9
ARFGEF2	ADP ribosylation factor guanine nucleotide exchange factor 2	0.00088	-1.679
ARG2	Arginase 2	0.019	1.776
ARHGAP12	Rho gtpase activating protein 12	0.000245	-1.514

ARHGAP18	Rho gtpase activating protein 18	0.00444	-1.562
ARHGAP21	Rho gtpase activating protein 21	5.21E-05	-2.096
ARHGAP31	Rho gtpase activating protein 31	0.0141	-1.516
ARHGAP32	Rho gtpase activating protein 32	0.00155	-1.815
ARHGAP35	Rho gtpase activating protein 35	0.000609	-1.882
ARHGAP39	Rho gtpase activating protein 39	0.00515	-1.746
ARHGAP44	Rho gtpase activating protein 44	0.00494	-1.872
ARHGAP5	Rho gtpase activating protein 5	0.00313	-1.6
ARHGEF11	Rho guanine nucleotide exchange factor 11	0.00202	-1.675
ARHGEF12	Rho guanine nucleotide exchange factor 12	0.0138	-1.51
ARHGEF18	Rho/Rac guanine nucleotide exchange factor 18	0.00203	-1.523
ARHGEF28	Rho guanine nucleotide exchange factor 28	0.000206	-1.814
ARHGEF38	Rho guanine nucleotide exchange factor 38	0.0115	-1.675
ARHGEF7	Rho guanine nucleotide exchange factor 7	0.000526	-1.495
ARID1A	AT-rich interaction domain 1A	0.00556	-1.596
ARID1B	AT-rich interaction domain 1B	0.000205	-1.877
ARID2	AT-rich interaction domain 2	0.00157	-1.518
ARID4A	AT-rich interaction domain 4A	5.79E-05	-2.093
ARID4B	AT-rich interaction domain 4B	2.33E-05	-2.115
ARL14EP	ADP ribosylation factor like gtpase 14 effector protein	2.44E-05	1.808
ARL3	ADP ribosylation factor like gtpase 3	0.000721	1.645
ARL6IP5	ADP ribosylation factor like gtpase 6 interacting protein 5	0.00115	1.495
ARMCX2	Armadillo repeat containing, X-linked 2	0.0107	1.545
ARPC3	Actin related protein 2/3 complex subunit 3	1.72E-05	1.933
ARPC4	Actin related protein 2/3 complex subunit 4	9.05E-05	1.623
ASAP2	Arfgap with SH3 domain, ankyrin repeat and PH domain 2	0.000334	-1.641
ASF1A	Anti-silencing function 1A histone chaperone	0.000034	1.654
ASH1L	ASH1 like histone lysine methyltransferase	9.85E-05	-1.846
ASNA1	Arsa arsenite transporter, ATP-binding, homolog 1 (bacterial)	0.000126	1.556
ASNS	Asparagine synthetase (glutamine-hydrolyzing)	2.16E-05	2.104

ASS1	Argininosuccinate synthase 1	0.00651	1.543
ASXL1	Additional sex combs like 1, transcriptional regulator	0.00179	-1.587
ASXL2	Additional sex combs like 2, transcriptional regulator	0.00439	-1.593
ATAD2B	Atpase family, AAA domain containing 2B	0.00178	-1.741
ATAD5	Atpase family, AAA domain containing 5	0.000174	-2.026
ATG2A	Autophagy related 2A	0.00147	-1.689
ATG2B	Autophagy related 2B	0.00216	-1.649
ATIC	5-aminoimidazole-4-carboxamide ribonucleotide formyltransferase/IMP cyclohydrolase	0.00124	1.664
ATM	ATM serine/threonine kinase	0.0172	-1.532
ATOX1	Antioxidant 1 copper chaperone	7.36E-05	1.783
ATP11C	Atpase phospholipid transporting 11C	0.00181	-1.555
ATP13A2	Atpase 13A2	0.00381	-1.587
ATP23	ATP23 metallopeptidase and ATP synthase assembly factor homolog	0.000205	1.823
ATP5A1	ATP synthase, H+ transporting, mitochondrial F1 complex, alpha subunit 1, cardiac muscle	0.000177	1.608
ATP5F1	ATP synthase, H+ transporting, mitochondrial Fo complex subunit B1	1.59E-05	1.737
ATP5G1	ATP synthase, H+ transporting, mitochondrial Fo complex subunit C1 (subunit 9)	0.000471	1.801
ATP5G3	ATP synthase, H+ transporting, mitochondrial Fo complex subunit C3 (subunit 9)	0.000177	1.901
ATP5H	ATP synthase, H+ transporting, mitochondrial Fo complex subunit D	3.23E-05	1.854
ATP5J	ATP synthase, H+ transporting, mitochondrial Fo complex subunit F6	2.83E-06	1.999
ATP5J2	ATP synthase, H+ transporting, mitochondrial Fo complex subunit F2	5.28E-06	2.185
ATP5L	ATP synthase, H+ transporting, mitochondrial Fo complex subunit G	0.00734	1.519
ATP5O	ATP synthase, H+ transporting, mitochondrial F1 complex, O subunit	5.46E-06	2.003
ATP6V0B	Atpase H+ transporting V0 subunit b	1.97E-05	1.684
ATP6V1D	Atpase H+ transporting V1 subunit D	4.85E-05	1.513
ATP6V1F	Atpase H+ transporting V1 subunit F	0.000449	1.641
ATP6V1G1	Atpase H+ transporting V1 subunit G1	0.000864	1.5
ATP7B	Atpase copper transporting beta	0.000792	-1.72
ATP8B1	Atpase phospholipid transporting 8B1	0.00113	-1.55
ATPIF1	Atpase inhibitory factor 1	0.00171	1.658
ATRAID	All-trans retinoic acid induced differentiation factor	7.81E-05	1.556

ATRX	ATRX, chromatin remodeler	0.00104	-1.872
ATXN2	Ataxin 2	0.000254	-1.573
ATXN7	Ataxin 7	0.00508	-1.546
AURKAIP1	Aurora kinase A interacting protein 1	0.00192	1.538
B2M	Beta-2-microglobulin	0.00577	1.946
B3GALNT1	Beta-1,3-N-acetylgalactosaminyltransferase 1 (globoside blood group)	0.00394	2.682
B9D1	B9 domain containing 1	0.00345	1.641
B9D2	B9 protein domain 2	0.0061	1.577
BAZ1B	Bromodomain adjacent to zinc finger domain 1B	6.17E-05	-1.657
BAZ2B	Bromodomain adjacent to zinc finger domain 2B	5.67E-06	-2.556
BBX	BBX, HMG-box containing	0.000808	-1.961
BCAP31	B-cell receptor-associated protein 31	0.000924	1.623
BCAS2	Breast carcinoma amplified sequence 2	0.00652	1.524
BCCIP	BRCA2 and CDKN1A interacting protein	0.00021	1.651
BCKDHB	Branched chain keto acid dehydrogenase E1 subunit beta	0.013	1.502
BCL2L15	BCL2 like 15	0.00903	2.436
BCL7A	BCL tumor suppressor 7A	0.024	-1.514
BCL7C	BCL tumor suppressor 7C	0.00288	1.516
BCLAF1	BCL2 associated transcription factor 1	9.58E-05	-1.746
BCOR	BCL6 corepressor	0.00216	-1.517
BCS1L	BCS1 homolog, ubiquinol-cytochrome c reductase complex chaperone	0.00369	1.52
BDP1	B double prime 1, subunit of RNA polymerase III transcription initiation factor IIIB	0.00206	-2.02
BET1	Bet1 golgi vesicular membrane trafficking protein	6.44E-05	1.675
BEX5	Brain expressed X-linked 5	1.75E-06	2.695
BICC1	Bicc family RNA binding protein 1	0.0196	-1.608
BICD2	BICD cargo adaptor 2	0.00062	-1.608
BICDL1	BICD family like cargo adaptor 1	0.00201	-1.81
BLM	Bloom syndrome recq like helicase	0.0108	-1.502
BLVRB	Biliverdin reductase B	0.000838	1.715
BOD1L1	Biorientation of chromosomes in cell division 1 like 1	0.000233	-2.051

BOLA1	Bola family member 1	0.000906	1.508
BPTF	Bromodomain PHD finger transcription factor	4.97E-05	-2.278
BRCA1	BRCA1, DNA repair associated	0.0134	-1.498
BRCA2	BRCA2, DNA repair associated	0.00699	-1.989
BRD1	Bromodomain containing 1	0.00439	-1.504
BRD2	Bromodomain containing 2	0.00337	-1.498
BRD3	Bromodomain containing 3	0.000228	-1.717
BRE	Brain and reproductive organ-expressed (TNFRSF1A modulator)	0.0003	1.506
BRF1	BRF1, RNA polymerase III transcription initiation factor 90 kda subunit	0.00335	-1.516
BRK1	BRICK1, SCAR/WAVE actin nucleating complex subunit	7.77E-05	1.643
BRPF3	Bromodomain and PHD finger containing 3	0.00369	-1.506
BRSK1	BR serine/threonine kinase 1	0.0138	-1.653
BRWD1	Bromodomain and WD repeat domain containing 1	0.000542	-1.742
BTBD8	BTB domain containing 8	0.00051	-1.852
BTF3	Basic transcription factor 3	1.42E-07	1.941
BTF3L4	Basic transcription factor 3 like 4	1.12E-07	1.937
BUD31	BUD31 homolog	0.000124	1.527
C12orf57	Chromosome 12 open reading frame 57	0.00144	1.959
C14orf1	Chromosome 14 open reading frame 1	0.00674	1.684
C14orf119	Chromosome 14 open reading frame 119	3.08E-05	1.829
C14orf166	Chromosome 14 open reading frame 166	9.17E-06	2.156
C19orf47	Chromosome 19 open reading frame 47	0.0277	-1.521
C1orf122	Chromosome 1 open reading frame 122	0.0116	1.566
C1orf27	Chromosome 1 open reading frame 27	0.000562	1.508
C1orf54	Chromosome 1 open reading frame 54	0.0148	1.697
C1QBP	Complement C1q binding protein	3.86E-05	1.633
C1QTNF7	C1q and tumor necrosis factor related protein 7	0.00423	1.781
C21orf62	Chromosome 21 open reading frame 62	0.0146	1.653
C7	Complement component 7	0.00637	-2.61
C8orf33	Chromosome 8 open reading frame 33	0.000323	1.619

C9orf16	Chromosome 9 open reading frame 16	0.00101	1.738
CA4	Carbonic anhydrase 4	0.0206	1.799
CABIN1	Calcineurin binding protein 1	0.00203	-1.628
CACNA1H	Calcium voltage-gated channel subunit alpha1 H	0.00367	-1.606
CAMK2G	Calcium/calmodulin dependent protein kinase II gamma	0.0063	-1.516
CAMSAP1	Calmodulin regulated spectrin associated protein 1	0.000178	-1.866
CAMSAP3	Calmodulin regulated spectrin associated protein family member 3	0.00343	-1.54
CAMTA1	Calmodulin binding transcription activator 1	0.000367	-2.008
CAMTA2	Calmodulin binding transcription activator 2	0.00118	-1.512
CAP1	Adenylate cyclase associated protein 1	0.00261	1.57
CAPG	Capping actin protein, gelsolin like	0.0083	1.548
CARMIL1	Capping protein regulator and myosin 1 linker 1	0.000679	-1.673
CASP8	Caspase 8	0.0235	1.824
CASP8AP2	Caspase 8 associated protein 2	0.00012	-1.79
CASZ1	Castor zinc finger 1	0.00864	-1.823
CATSPERG	Cation channel sperm associated auxiliary subunit gamma	0.00231	-2.204
CBX4	Chromobox 4	0.00289	-1.626
CC2D1B	Coiled-coil and C2 domain containing 1B	0.000219	-1.654
CC2D2A	Coiled-coil and C2 domain containing 2A	0.00589	-1.769
CCDC107	Coiled-coil domain containing 107	0.00237	1.504
CCDC14	Coiled-coil domain containing 14	0.00216	-1.591
CCDC142	Coiled-coil domain containing 142	0.00357	-1.743
CCDC150	Coiled-coil domain containing 150	0.000198	-2.175
CCDC158	Coiled-coil domain containing 158	5.18E-06	-2.329
CCDC167	Coiled-coil domain containing 167	0.0137	1.527
CCDC170	Coiled-coil domain containing 170	0.006	-2.088
CCDC186	Coiled-coil domain containing 186	0.000135	-2.159
CCDC191	Coiled-coil domain containing 191	0.0183	-1.559
CCDC30	Coiled-coil domain containing 30	0.0288	-1.745
CCDC39	Coiled-coil domain containing 39	0.00874	-2.455

CCDC40	Coiled-coil domain containing 40	0.0199	-2.106
CCDC53	Coiled-coil domain containing 53	0.000114	1.687
CCDC57	Coiled-coil domain containing 57	0.0106	-1.751
CCDC58	Coiled-coil domain containing 58	3.24E-05	1.875
CCDC59	Coiled-coil domain containing 59	0.000171	1.496
CCDC66	Coiled-coil domain containing 66	0.000235	-1.58
CCDC77	Coiled-coil domain containing 77	0.0161	-1.532
CCDC88A	Coiled-coil domain containing 88A	0.0266	-1.698
CCNG1	Cyclin G1	0.0016	1.769
CCNH	Cyclin H	0.000954	1.551
CCNT2	Cyclin T2	0.000386	-1.533
CCP110	Centriolar coiled-coil protein 110	0.00935	-1.55
CCPG1	Cell cycle progression 1	4.19E-05	-1.701
CCRL2	C-C motif chemokine receptor like 2	0.000931	2.296
CCT2	Chaperonin containing TCP1 subunit 2	0.000512	1.583
CCT3	Chaperonin containing TCP1 subunit 3	0.000693	1.56
CCT4	Chaperonin containing TCP1 subunit 4	6.45E-05	1.631
CCT6A	Chaperonin containing TCP1 subunit 6A	0.00888	1.577
CCT7	Chaperonin containing TCP1 subunit 7	0.000101	1.525
CD1E	CD1e molecule	0.017	1.877
CD2AP	CD2 associated protein	0.0102	-1.505
CD3G	CD3g molecule	0.0166	1.87
CD48	CD48 molecule	0.000386	2.283
CD59	CD59 molecule	0.00054	2.119
CD63	CD63 molecule	1.64E-05	1.958
CD9	CD9 molecule	4.76E-05	1.75
CDAN1	Codanin 1	0.00345	-1.547
CDC26	Cell division cycle 26	8.57E-05	1.59
CDC42	Cell division cycle 42	0.00018	1.594
CDC42BPB	CDC42 binding protein kinase beta	0.00069	-1.587

CDC42BPG	CDC42 binding protein kinase gamma	3.65E-05	-1.845
CDCP1	CUB domain containing protein 1	0.00332	-1.636
CDH17	Cadherin 17	0.0154	1.88
CDH3	Cadherin 3	0.011	-1.587
CDK13	Cyclin dependent kinase 13	0.000759	-1.572
CDK3	Cyclin dependent kinase 3	0.00925	-2.051
CDK4	Cyclin dependent kinase 4	7.70E-06	1.641
CDK5RAP2	CDK5 regulatory subunit associated protein 2	0.000389	-1.787
CDK8	Cyclin dependent kinase 8	0.00196	-1.672
CDKN2AIPNL	CDKN2A interacting protein N-terminal like	3.70E-06	1.938
CDON	Cell adhesion associated, oncogene regulated	0.0301	-1.516
CENPC	Centromere protein C	0.000963	-1.512
CENPJ	Centromere protein J	0.00114	-1.861
CEP104	Centrosomal protein 104	0.000255	-1.75
CEP112	Centrosomal protein 112	0.0142	-1.629
CEP120	Centrosomal protein 120	6.06E-06	-1.644
CEP128	Centrosomal protein 128	0.00744	-1.706
CEP135	Centrosomal protein 135	0.00292	-1.834
CEP162	Centrosomal protein 162	0.00196	-1.938
CEP164	Centrosomal protein 164	0.0121	-1.647
CEP170	Centrosomal protein 170	8.13E-05	-1.964
CEP170B	Centrosomal protein 170B	0.00103	-1.777
CEP250	Centrosomal protein 250	0.000701	-2.041
CEP295	Centrosomal protein 295	0.00122	-1.717
CEP350	Centrosomal protein 350	0.000446	-2.301
CEP85L	Centrosomal protein 85 like	0.0236	-1.654
CEP89	Centrosomal protein 89	0.000066	-1.766
CEP95	Centrosomal protein 95	0.000481	-1.671
CEPT1	Choline/ethanolamine phosphotransferase 1	3.63E-05	1.655
CETN2	Centrin 2	0.00576	1.552

CFAP69	Cilia and flagella associated protein 69	0.0233	-1.658
CGNL1	Cingulin like 1	0.000558	-1.827
CGRRF1	Cell growth regulator with ring finger domain 1	0.00252	1.509
CH25H	Cholesterol 25-hydroxylase	0.00463	2.187
CHAD	Chondroadherin	0.00675	-1.822
CHAF1A	Chromatin assembly factor 1 subunit A	0.00537	-1.508
CHCHD2	Coiled-coil-helix-coiled-coil-helix domain containing 2	0.00115	1.678
CHCHD4	Coiled-coil-helix-coiled-coil-helix domain containing 4	0.000336	1.521
CHCHD7	Coiled-coil-helix-coiled-coil-helix domain containing 7	0.000143	1.535
CHD1	Chromodomain helicase DNA binding protein 1	0.000108	-1.702
CHD3	Chromodomain helicase DNA binding protein 3	0.0128	-1.508
CHD6	Chromodomain helicase DNA binding protein 6	0.000563	-2.137
CHD7	Chromodomain helicase DNA binding protein 7	0.000766	-2.125
CHD8	Chromodomain helicase DNA binding protein 8	0.00071	-1.709
CHD9	Chromodomain helicase DNA binding protein 9	0.00209	-1.96
CHIC2	Cysteine rich hydrophobic domain 2	0.00108	1.542
CHML	CHM like, Rab escort protein 2	0.0293	-1.514
CHODL	Chondrolectin	0.0115	2.716
CHST11	Carbohydrate (chondroitin 4) sulfotransferase 11	0.0147	-1.638
CIB1	Calcium and integrin binding 1	0.00466	1.545
CIRBP	Cold inducible RNA binding protein	0.000152	1.854
CKAP2	Cytoskeleton associated protein 2	0.0303	-1.648
CKAP5	Cytoskeleton associated protein 5	9.53E-06	-1.721
CKS1B	CDC28 protein kinase regulatory subunit 1B	0.00157	1.744
CKS2	CDC28 protein kinase regulatory subunit 2	0.0249	1.531
CLCN2	Chloride voltage-gated channel 2	0.00671	-1.721
CLCN6	Chloride voltage-gated channel 6	0.00055	-1.79
CLDN10	Claudin 10	0.00537	2.569
CLDND1	Claudin domain containing 1	0.0023	1.536
CLEC16A	C-type lectin domain family 16 member A	0.00761	-1.499

CLIC1	Chloride intracellular channel 1	0.000352	1.86
CLIP1	CAP-Gly domain containing linker protein 1	1.21E-06	-2.474
CLNS1A	Chloride nucleotide-sensitive channel 1A	0.00032	1.538
CLTA	Clathrin light chain A	0.000168	1.53
CMC1	C-X9-C motif containing 1	0.000515	1.565
CMIP	C-Maf inducing protein	0.00518	-1.529
CMPK1	Cytidine/uridine monophosphate kinase 1	0.000116	1.761
CMSS1	Cms1 ribosomal small subunit homolog (yeast)	0.00159	1.72
CMTR2	Cap methyltransferase 2	0.033	1.505
CNEP1R1	CTD nuclear envelope phosphatase 1 regulatory subunit 1	0.000261	1.597
CNIH4	Cornichon family AMPA receptor auxiliary protein 4	3.83E-06	2.004
CNN2	Calponin 2	5.04E-05	1.55
CNNM2	Cyclin and CBS domain divalent metal cation transport mediator 2	0.00207	-1.623
CNOT1	CCR4-NOT transcription complex subunit 1	0.0218	-1.499
CNPY2	Canopy FGF signaling regulator 2	2.33E-05	1.812
CNRIP1	Cannabinoid receptor interacting protein 1	0.0115	1.512
CNTLN	Centlein	0.00132	-1.863
CNTRL	Centriolin	0.00116	-2.045
COA3	Cytochrome c oxidase assembly factor 3	0.000433	1.532
COBL	Cordon-bleu WH2 repeat protein	0.0235	-1.589
COBLL1	Cordon-bleu WH2 repeat protein like 1	0.000757	-1.844
COL12A1	Collagen type XII alpha 1 chain	0.00641	-1.774
COLEC12	Collectin subfamily member 12	0.00578	-1.552
COLQ	Collagen like tail subunit of asymmetric acetylcholinesterase	0.0223	-1.66
COMMD1	Copper metabolism domain containing 1	1.43E-05	1.948
COMMD3	COMM domain containing 3	0.000958	1.644
COMMD4	COMM domain containing 4	5.36E-05	1.68
COMMD5	COMM domain containing 5	0.00053	1.621
COPRS	Coordinator of PRMT5 and differentiation stimulator	0.00123	1.724
COPS5	COP9 signalosome subunit 5	0.000203	1.526

COQ3	Coenzyme Q3, methyltransferase	0.00188	1.648
COQ5	Coenzyme Q5, methyltransferase	0.000587	1.517
COX14	COX14, cytochrome c oxidase assembly factor	8.07E-05	1.649
COX16	COX16, cytochrome c oxidase assembly homolog	5.24E-05	1.635
COX17	COX17, cytochrome c oxidase copper chaperone	0.0015	1.619
COX4I1	Cytochrome c oxidase subunit 4I1	0.000187	1.64
COX5B	Cytochrome c oxidase subunit 5B	1.49E-05	1.994
COX6B1	Cytochrome c oxidase subunit 6B1	0.000109	1.831
Cox6c	Cytochrome c oxidase subunit vic	0.000168	1.898
COX7A1	Cytochrome c oxidase subunit 7A1	0.00681	1.637
COX7A2	Cytochrome c oxidase subunit 7A2	0.000218	1.867
COX7A2L	Cytochrome c oxidase subunit 7A2 like	4.13E-06	2.077
Cox7c	Cytochrome c oxidase subunit viic	0.000162	3.049
COX8A	Cytochrome c oxidase subunit 8A	0.00072	1.5
CPED1	Cadherin like and PC-esterase domain containing 1	0.0119	-1.502
CPQ	Carboxypeptidase Q	0.0131	1.587
CPXM1	Carboxypeptidase X (M14 family), member 1	0.00744	-1.6
CRAMP1	Cramped chromatin regulator homolog 1	0.000446	-1.923
CREBRF	CREB3 regulatory factor	0.00956	-1.569
CRIPT	CXXC repeat containing interactor of PDZ3 domain	4.96E-05	1.781
CRYBB1	Crystallin beta B1	0.00385	2.047
CRYZ	Crystallin zeta	2.65E-05	1.645
CRYZL1	Crystallin zeta like 1	0.00014	1.652
CSF2RA	Colony stimulating factor 2 receptor alpha subunit	0.0137	-1.609
CSPP1	Centrosome and spindle pole associated protein 1	0.000368	-2.078
CST3	Cystatin C	0.0128	1.572
CSTB	Cystatin B	0.000177	1.806
CTAGE5	CTAGE family member 5, ER export factor	0.0119	-3.09
CTSL	Cathepsin L	0.0207	2.259
CTSV	Cathepsin V	0.000254	1.958

CTTNBP2	Cortactin binding protein 2	6.52E-05	-2.045
CUL7	Cullin 7	0.00179	-1.55
CUL9	Cullin 9	0.00809	-1.54
CUTC	Cutc copper transporter	3.21E-05	1.725
CXCL16	C-X-C motif chemokine ligand 16	0.000499	1.636
CXorf56	Chromosome X open reading frame 56	0.00176	1.556
CYBA	Cytochrome b-245 alpha chain	0.00101	1.518
CYCS	Cytochrome c, somatic	0.00295	1.956
CYP20A1	Cytochrome P450 family 20 subfamily A member 1	2.63E-05	1.903
CYP2R1	Cytochrome P450 family 2 subfamily R member 1	0.000105	1.768
DAD1	Defender against cell death 1	1.18E-05	1.991
DAGLA	Diacylglycerol lipase alpha	0.00958	-1.764
DAGLB	Diacylglycerol lipase beta	0.00146	-1.689
DARS	Aspartyl-trna synthetase	0.000245	1.586
DBI	Diazepam binding inhibitor, acyl-coa binding protein	0.000231	1.738
DCDC1	Doublecortin domain containing 1	0.0068	-2.611
DCLK2	Doublecortin like kinase 2	0.0114	-2.072
DCTN5	Dynactin subunit 5	1.66E-06	1.994
DCTPP1	Dctp pyrophosphatase 1	0.00014	1.866
DDT	D-dopachrome tautomerase	0.000692	1.773
DDX47	DEAD-box helicase 47	3.68E-05	1.609
DEGS1	Delta 4-desaturase, sphingolipid 1	8.71E-05	2.052
DEPTOR	DEP domain containing MTOR-interacting protein	0.0102	-1.859
DERA	Deoxyribose-phosphate aldolase	0.000128	1.759
DERL1	Derlin 1	1.44E-05	1.756
DERL2	Derlin 2	8.90E-06	1.667
DGKD	Diacylglycerol kinase delta	0.00293	-1.516
DGKQ	Diacylglycerol kinase theta	0.000385	-1.89
DGUOK	Deoxyguanosine kinase	6.89E-07	2.027
DHDH	Dihydrodiol dehydrogenase	0.0159	1.682

DHRS1	Dehydrogenase/reductase 1	5.01E-06	1.756
DHX34	DEAH-box helicase 34	0.00163	-1.561
DIABLO	Diablo IAP-binding mitochondrial protein	1.83E-05	1.603
DIDO1	Death inducer-obliterator 1	0.000673	-1.538
DIMT1	DIM1 dimethyladenosine transferase 1 homolog	0.00156	1.753
DIP2A	Disco interacting protein 2 homolog A	0.00522	-1.558
DIRC2	Disrupted in renal carcinoma 2	0.0137	1.556
DKKL1	Dickkopf like acrosomal protein 1	0.00368	1.669
DLC1	DLC1 Rho gtpase activating protein	0.00252	-1.674
DLL1	Delta like canonical Notch ligand 1	0.00166	-1.903
DLX6	Distal-less homeobox 6	0.0105	-1.592
Dmd	Dystrophin	0.000245	-1.877
DMXL1	Dmx like 1	0.00781	-1.643
DNAH12	Dynein axonemal heavy chain 12	0.00374	-2.196
DNAH2	Dynein axonemal heavy chain 2	0.0106	-2.455
DNAH5	Dynein axonemal heavy chain 5	0.00834	-2.439
DNAJB11	Dnaj heat shock protein family (Hsp40) member B11	0.00554	1.59
DNAJB4	Dnaj heat shock protein family (Hsp40) member B4	0.00229	1.536
DNAJC19	Dnaj heat shock protein family (Hsp40) member C19	0.00205	1.627
DNASE2	Deoxyribonuclease II, lysosomal	0.0119	1.836
DNMT1	DNA (cytosine-5-)-methyltransferase 1	0.000964	-1.529
DNMT3A	DNA methyltransferase 3 alpha	0.000802	-1.952
DOCK5	Dedicator of cytokinesis 5	0.00556	-1.673
DOCK8	Dedicator of cytokinesis 8	0.000374	-2.012
DOPEY1	Dopey family member 1	0.000485	-1.534
DPH6	Diphthamine biosynthesis 6	7.73E-05	1.767
DPM3	Dolichyl-phosphate mannosyltransferase subunit 3	0.000257	2.063
DPY30	Dpy-30, histone methyltransferase complex regulatory subunit	2.08E-05	1.753
DSP	Desmoplakin	0.00452	-1.707
DST	Dystonin	0.0155	-1.7

DSTYK	Dual serine/threonine and tyrosine protein kinase	7.35E-05	-1.518
DTYMK	Deoxythymidylate kinase	0.0171	1.559
DUSP23	Dual specificity phosphatase 23	0.0161	1.669
DUSP5	Dual specificity phosphatase 5	0.0162	1.813
DUT	Deoxyuridine triphosphatase	0.000017	2.153
DYNC1H1	Dynein cytoplasmic 1 heavy chain 1	0.00209	-1.615
DYNC2LI1	Dynein cytoplasmic 2 light intermediate chain 1	0.00267	1.517
DYNLRB1	Dynein light chain roadblock-type 1	0.000014	1.823
DZIP1	DAZ interacting zinc finger protein 1	0.00278	-1.558
DZIP3	DAZ interacting zinc finger protein 3	5.08E-06	-1.971
ECSCR	Endothelial cell surface expressed chemotaxis and apoptosis regulator	0.00356	1.66
EDEM3	ER degradation enhancing alpha-mannosidase like protein 3	0.0059	-1.547
EEA1	Early endosome antigen 1	0.000486	-2.323
EEF1A1	Eukaryotic translation elongation factor 1 alpha 1	0.000676	1.754
EEF1B2	Eukaryotic translation elongation factor 1 beta 2	2.37E-06	2.425
EEF1D	Eukaryotic translation elongation factor 1 delta	4.85E-06	1.965
EEF1E1	Eukaryotic translation elongation factor 1 epsilon 1	0.000214	1.81
EEF1G	Eukaryotic translation elongation factor 1 gamma	1.39E-06	2.035
EEF2K	Eukaryotic elongation factor 2 kinase	6.41E-05	-1.968
EFCAB11	EF-hand calcium binding domain 11	0.019	1.639
EGFR	Epidermal growth factor receptor	0.00164	-1.76
EHBP1	EH domain binding protein 1	0.00022	-1.569
EHHADH	Enoyl-coa, hydratase/3-hydroxyacyl coa dehydrogenase	0.0264	-1.507
EHMT1	Euchromatic histone lysine methyltransferase 1	2.92E-05	-1.614
EID2	EP300 interacting inhibitor of differentiation 2	0.00721	1.739
EIF1AD	Eukaryotic translation initiation factor 1A domain containing	0.000148	1.774
EIF1AX	Eukaryotic translation initiation factor 1A, X-linked	3.69E-06	1.952
EIF2B1	Eukaryotic translation initiation factor 2B subunit alpha	0.000279	1.524
EIF2S3	Eukaryotic translation initiation factor 2 subunit gamma	5.02E-05	1.965
EIF3E	Eukaryotic translation initiation factor 3 subunit E	2.13E-05	2.168

EIF3G	Eukaryotic translation initiation factor 3 subunit G	0.000075	1.561
EIF3H	Eukaryotic translation initiation factor 3 subunit H	5.64E-05	1.675
EIF3I	Eukaryotic translation initiation factor 3 subunit I	4.91E-05	1.876
EIF3K	Eukaryotic translation initiation factor 3 subunit K	1.08E-05	1.752
EIF3M	Eukaryotic translation initiation factor 3 subunit M	3.38E-06	2.44
EIF4A1	Eukaryotic translation initiation factor 4A1	0.0127	1.704
EIF4A3	Eukaryotic translation initiation factor 4A3	0.000085	1.543
EIF4G3	Eukaryotic translation initiation factor 4 gamma 3	3.38E-05	-1.951
EIF6	Eukaryotic translation initiation factor 6	0.000396	1.533
ELK3	ELK3, ETS transcription factor	0.00698	-1.587
ELP4	Elongator acetyltransferase complex subunit 4	0.0023	1.505
EMC4	ER membrane protein complex subunit 4	0.000057	1.642
EMC6	ER membrane protein complex subunit 6	1.79E-05	1.682
EMG1	EMG1, N1-specific pseudouridine methyltransferase	0.000199	1.659
EML1	Echinoderm microtubule associated protein like 1	0.00145	-1.513
EML5	Echinoderm microtubule associated protein like 5	0.000865	-2.136
EMP3	Epithelial membrane protein 3	0.00503	1.691
EMSY	EMSY, BRCA2 interacting transcriptional repressor	0.00102	-1.63
ENO1	Enolase 1	0.00104	1.56
ENO2	Enolase 2	0.00542	1.581
ENOPH1	Enolase-phosphatase 1	0.000436	1.659
ENPP6	Ectonucleotide pyrophosphatase/phosphodiesterase 6	0.000592	-2.151
ENY2	ENY2, transcription and export complex 2 subunit	0.000982	1.563
EP400	E1A binding protein p400	0.00249	-1.607
EPC1	Enhancer of polycomb homolog 1	0.00027	-1.724
EPHB1	EPH receptor B1	0.0279	1.818
EPHB4	EPH receptor B4	0.00413	-1.534
ERBIN	Erbb2 interacting protein	0.000193	-1.568
ERCC6	ERCC excision repair 6, chromatin remodeling factor	0.00341	-1.556
ERCC6L2	ERCC excision repair 6 like 2	0.000015	-1.854

ERGIC3	ERGIC and golgi 3	8.48E-05	1.672
ERH	Enhancer of rudimentary homolog (Drosophila)	5.73E-05	1.807
ERICH3	Glutamate rich 3	0.0253	-2.247
ERN1	Endoplasmic reticulum to nucleus signaling 1	0.0141	-1.607
ERN2	Endoplasmic reticulum to nucleus signaling 2	0.0161	-1.872
ERP29	Endoplasmic reticulum protein 29	2.01E-05	1.688
ESCO1	Establishment of sister chromatid cohesion N-acetyltransferase 1	0.000134	-2.115
ESD	Esterase D	0.000556	1.496
ESR2	Estrogen receptor 2	0.00778	-2.06
ETAA1	Ewing tumor associated antigen 1	1.42E-05	-1.526
ETFA	Electron transfer flavoprotein alpha subunit	1.71E-05	1.772
ETHE1	ETHE1, persulfide dioxygenase	0.00378	1.551
ETV3	ETS variant 3	0.000359	-1.626
EVC	Evc ciliary complex subunit 1	0.000836	-1.585
EXOSC1	Exosome component 1	0.000103	1.771
EXOSC4	Exosome component 4	0.000282	1.79
EXOSC8	Exosome component 8	0.000613	1.582
EXPH5	Exophilin 5	0.0245	-1.7
F5	Coagulation factor V	0.00929	-2.012
F8	Coagulation factor VIII	0.00931	-1.569
FABP7	Fatty acid binding protein 7	0.000478	2.333
FAM103A1	Family with sequence similarity 103 member A1	0.00185	1.548
FAM161A	Family with sequence similarity 161 member A	0.0103	-1.665
FAM184A	Family with sequence similarity 184 member A	0.0234	-1.546
FAM185A	Family with sequence similarity 185 member A	0.00136	-1.904
FAM193A	Family with sequence similarity 193 member A	0.000343	-1.752
FAM193B	Family with sequence similarity 193 member B	0.00123	-1.89
FAM214A	Family with sequence similarity 214 member A	3.71E-06	-2.001
FAM229B	Family with sequence similarity 229 member B	0.00669	1.99
FAM65C	Family with sequence similarity 65 member C	0.00718	-1.827

FAM69A	Family with sequence similarity 69 member A	0.0251	1.589
FAM96B	Family with sequence similarity 96 member B	0.00232	1.534
FANCC	Fanconi anemia complementation group C	0.0013	1.529
FASTKD2	FAST kinase domains 2	0.000598	1.552
FAT1	FAT atypical cadherin 1	0.0211	-1.527
FBF1	Fas binding factor 1	0.00427	-1.589
FBL	Fibrillarin	0.000057	1.909
FBN1	Fibrillin 1	0.00202	-1.63
FBRSL1	Fibrosin like 1	0.00166	-1.641
FBXL5	F-box and leucine rich repeat protein 5	0.000274	1.613
FBXO10	F-box protein 10	0.00461	-1.681
FBXO17	F-box protein 17	0.0167	1.527
FBXO31	F-box protein 31	0.00129	-1.774
FBXW7	F-box and WD repeat domain containing 7	0.000239	-1.674
FCF1	FCF1 rrna-processing protein	3.89E-06	1.941
FDPS	Farnesyl diphosphate synthase	0.00654	1.546
FDX1L	Ferredoxin 1 like	0.00042	1.579
FER1L5	Fer-1 like family member 5	0.000643	-1.916
FGD4	FYVE, rhogef and PH domain containing 4	0.0133	-1.7
FGF13	Fibroblast growth factor 13	0.0186	1.719
FGFRL1	Fibroblast growth factor receptor-like 1	0.0169	-1.766
FHAD1	Forkhead associated phosphopeptide binding domain 1	0.00583	-2.526
FHDC1	FH2 domain containing 1	0.0224	-1.667
FILIP1	Filamin A interacting protein 1	0.00456	-2.109
FILIP1L	Filamin A interacting protein 1 like	0.000265	-1.927
FIS1	Fission, mitochondrial 1	0.000456	1.556
FKBP11	FK506 binding protein 11	0.000013	1.97
FKBP15	FK506 binding protein 15	0.000252	-1.719
FKBP1A	FK506 binding protein 1A	0.000495	1.577
FKBP2	FK506 binding protein 2	0.00187	1.5

FKBP3	FK506 binding protein 3	8.91E-05	1.667
FKBP7	FK506 binding protein 7	0.000899	1.827
FLNB	Filamin B	0.0011	-1.677
FLYWCH1	FLYWCH-type zinc finger 1	0.0247	-1.524
FMNL2	Formin like 2	0.00024	-1.819
FMO4	Flavin containing monooxygenase 4	0.00119	-1.982
FMR1	Fragile X mental retardation 1	0.00383	-1.555
FNBP4	Formin binding protein 4	3.15E-05	-2.747
FNDC3A	Fibronectin type III domain containing 3A	0.00197	-1.557
FNTA	Farnesyltransferase, CAAX box, alpha	3.25E-05	1.612
FOLR1	Folate receptor 1	0.0113	2.248
FOXO3	Forkhead box O3	0.0202	-1.545
Foxp1	Forkhead box P1	0.00123	-1.618
FREM2	FRAS1 related extracellular matrix protein 2	0.0278	-2.223
FRMD4A	FERM domain containing 4A	0.000812	-1.698
FRMPD2	FERM and PDZ domain containing 2	0.0028	-3.226
FRRS1	Ferric chelate reductase 1	0.00539	1.598
FRY	FRY microtubule binding protein	0.000314	-2.123
FRYL	FRY like transcription coactivator	0.00846	-1.779
FSD2	Fibronectin type III and SPRY domain containing 2	0.000522	-2.083
FUNDC1	FUN14 domain containing 1	0.000406	1.711
FURIN	Furin, paired basic amino acid cleaving enzyme	0.00434	-1.525
FYCO1	FYVE and coiled-coil domain containing 1	0.000468	-1.773
FZD5	Frizzled class receptor 5	0.0179	-1.509
G0S2	G0/G1 switch 2	0.0083	1.747
G3BP1	G3BP stress granule assembly factor 1	5.22E-05	1.678
GABARAPL2	GABA type A receptor associated protein like 2	0.000373	1.772
GALNT12	Polypeptide N-acetylgalactosaminyltransferase 12	0.00708	1.593
GALNT6	Polypeptide N-acetylgalactosaminyltransferase 6	0.0296	-1.567
GANC	Glucosidase alpha, neutral C	0.00805	-1.722

GAPDH	Glyceraldehyde-3-phosphate dehydrogenase	0.00144	1.554
GAREM1	GRB2 associated regulator of MAPK1 subtype 1	0.00717	-1.554
GATA2	GATA binding protein 2	0.0101	-1.561
GBP2	Guanylate binding protein 2	0.016	-7.43
GCC2	GRIP and coiled-coil domain containing 2	3.60E-06	-2.512
GCH1	GTP cyclohydrolase 1	0.0108	1.714
GEMIN2	Gem nuclear organelle associated protein 2	0.000011	2.018
GEMIN6	Gem nuclear organelle associated protein 6	0.000434	1.694
GGH	Gamma-glutamyl hydrolase	0.0173	1.714
GGT5	Gamma-glutamyltransferase 5	0.00722	-1.882
GIGYF1	GRB10 interacting GYF protein 1	0.00819	-1.87
GIGYF2	GRB10 interacting GYF protein 2	6.74E-05	-1.647
GIT1	GIT arfgap 1	0.000121	-1.624
GJB2	Gap junction protein beta 2	1.77E-05	7.674
GK	Glycerol kinase	0.0201	1.619
GLIPR2	GLI pathogenesis related 2	0.0163	1.599
GLIS2	GLIS family zinc finger 2	0.0229	-1.568
GLRX	Glutaredoxin	0.000465	1.854
GLRX2	Glutaredoxin 2	3.06E-05	2.015
GLRX3	Glutaredoxin 3	8.79E-05	1.727
GLTSCR1L	GLTSCR1 like	0.00348	-1.581
GNAL	G protein subunit alpha L	0.00262	-2.246
GNAT2	G protein subunit alpha transducin 2	0.00274	-1.642
GNG11	G protein subunit gamma 11	0.00922	1.6
GNG5	G protein subunit gamma 5	0.000045	1.521
GNPTAB	N-acetylglucosamine-1-phosphate transferase alpha and beta subunits	0.000233	-1.697
GOLGA1	Golgin A1	0.00014	-1.604
GOLGA3	Golgin A3	7.68E-05	-1.857
GOLGA4	Golgin A4	2.03E-05	-2.354
GOLGB1	Golgin B1	3.26E-05	-2.124

GOLT1B	Golgi transport 1B	4.53E-05	1.806
GON4L	Gon-4 like	3.99E-05	-1.731
GPATCH8	G-patch domain containing 8	0.000178	-1.92
GPT	Glutamic--pyruvic transaminase	0.0161	-1.798
GPX8	Glutathione peroxidase 8 (putative)	0.0012	1.827
GRAMD1B	GRAM domain containing 1B	0.00411	-1.995
GRB10	Growth factor receptor bound protein 10	0.00373	-1.537
GRHL1	Grainyhead like transcription factor 1	0.0044	-1.692
GRHL2	Grainyhead like transcription factor 2	0.00103	-1.755
GRHPR	Glyoxylate reductase/hydroxypyruvate reductase	1.21E-05	1.856
GRK3	G protein-coupled receptor kinase 3	0.00754	-1.553
GRK4	G protein-coupled receptor kinase 4	0.000284	-2.282
GRPEL1	Grpe like 1, mitochondrial	7.44E-06	1.641
GSAP	Gamma-secretase activating protein	0.0128	-1.52
GSE1	Gse1 coiled-coil protein	0.00331	-1.663
GSR	Glutathione-disulfide reductase	0.00113	1.601
GSTA4	Glutathione S-transferase alpha 4	0.00426	1.53
GSTK1	Glutathione S-transferase kappa 1	0.000857	1.514
GSTO1	Glutathione S-transferase omega 1	0.00553	1.782
GSTP1	Glutathione S-transferase pi 1	0.000688	1.54
GTF2A2	General transcription factor IIA subunit 2	1.76E-05	1.671
GTF2B	General transcription factor IIB	0.0142	1.79
GTF2H2	General transcription factor IIH subunit 2	0.00165	1.55
GTF2H3	General transcription factor IIH subunit 3	0.000486	1.648
GTF2H5	General transcription factor IIH subunit 5	0.0009	1.602
GTF3C1	General transcription factor IIIC subunit 1	0.000734	-1.69
GTPBP10	GTP binding protein 10	0.000489	1.542
GUCY1A2	Guanylate cyclase 1 soluble subunit alpha 2	0.00749	-1.925
GUCY1A3	Guanylate cyclase 1 soluble subunit alpha	0.000783	-1.758
GZMK	Granzyme K	0.00381	3.901

H2AFJ	H2A histone family member J	0.000841	1.536
H2AFX	H2A histone family member X	0.00384	1.615
HACD3	3-hydroxyacyl-coa dehydratase 3	0.00047	1.704
HACD4	3-hydroxyacyl-coa dehydratase 4	0.00195	2.346
HAT1	Histone acetyltransferase 1	1.93E-05	1.551
HAUS2	HAUS augmin like complex subunit 2	0.000932	1.51
HAX1	HCLS1 associated protein X-1	3.90E-06	1.89
HEBP1	Heme binding protein 1	2.04E-05	2.138
HEBP2	Heme binding protein 2	8.05E-05	1.75
HECTD1	HECT domain E3 ubiquitin protein ligase 1	4.19E-05	-1.658
HERC2	HECT and RLD domain containing E3 ubiquitin protein ligase 2	6.25E-05	-1.772
HEXB	Hexosaminidase subunit beta	0.00182	4.013
HIGD1A	HIG1 hypoxia inducible domain family member 1A	0.0048	2.417
HIKESHI	Hikeshi, heat shock protein nuclear import factor	1.54E-05	1.943
HINT1	Histidine triad nucleotide binding protein 1	2.64E-05	2.124
HIP1	Huntingtin interacting protein 1	0.00399	-1.795
HIRA	Histone cell cycle regulator	0.000784	-1.516
HIST1H2AC	Histone cluster 1, h2ac	0.00119	2.168
HIST1H2BD	Histone cluster 1, h2bd	0.00704	1.826
HIST1H4J	Histone cluster 1, h4j	9.76E-05	2.528
HIVEP1	Human immunodeficiency virus type I enhancer binding protein 1	0.0146	-1.634
HMCN1	Hemicentin 1	0.0143	-2.293
HMGXB4	HMG-box containing 4	0.00238	-1.5
HMMR	Hyaluronan mediated motility receptor	0.00404	-1.631
HN1	Hematological and neurological expressed 1	0.000211	1.76
HNRNPUL1	Heterogeneous nuclear ribonucleoprotein U like 1	0.000982	-1.498
HOOK1	Hook microtubule-tethering protein 1	0.00786	-1.587
HOXB3	Homeobox B3	0.00734	-1.537
HOXB9	Homeobox B9	0.0181	-1.567
HSD17B10	Hydroxysteroid 17-beta dehydrogenase 10	0.000142	1.691

HSPA12A	Heat shock protein family A (Hsp70) member 12A	0.0104	-1.706
HSPA14	Heat shock protein family A (Hsp70) member 14	0.000107	1.693
HSPB11	Heat shock protein family B (small) member 11	0.00273	1.558
HSPE1	Heat shock protein family E (Hsp10) member 1	0.000018	2.095
HTRA3	Htra serine peptidase 3	0.0175	-1.641
HTT	Huntingtin	0.00197	-1.531
HYPK	Huntingtin interacting protein K	9.57E-05	1.74
ICE1	Interactor of little elongation complex ELL subunit 1	0.00011	-1.794
ID1	Inhibitor of DNA binding 1, HLH protein	0.000536	2.145
ID3	Inhibitor of DNA binding 3, HLH protein	0.00783	1.687
IDH1	Isocitrate dehydrogenase (NADP(+)) 1, cytosolic	0.0287	1.54
IDNK	IDNK, gluconokinase	0.00144	2.243
IER3IP1	Immediate early response 3 interacting protein 1	0.000493	1.712
IFT122	Intraflagellar transport 122	0.00951	-1.719
IFT172	Intraflagellar transport 172	0.0113	-1.656
IFT20	Intraflagellar transport 20	5.98E-06	1.799
IFT74	Intraflagellar transport 74	0.00689	-1.516
IGBP1	Immunoglobulin (CD79A) binding protein 1	2.37E-05	1.729
IGF2	Insulin like growth factor 2	0.0139	1.674
IGFBP6	Insulin like growth factor binding protein 6	0.0322	1.526
IGHMBP2	Immunoglobulin mu binding protein 2	0.00593	-1.593
IGSF3	Immunoglobulin superfamily member 3	0.00451	-1.77
IKBKB	Inhibitor of kappa light polypeptide gene enhancer in B-cells, kinase beta	0.000128	-1.584
IL6R	Interleukin 6 receptor	0.00746	-1.616
ILDR1	Immunoglobulin like domain containing receptor 1	0.0148	-1.522
ILF2	Interleukin enhancer binding factor 2	0.00301	1.534
ILVBL	Ilvb acetolactate synthase like	0.0101	1.499
IMMP2L	Inner mitochondrial membrane peptidase subunit 2	0.00554	1.595
IMPDH2	Inosine monophosphate dehydrogenase 2	2.35E-05	1.737
IMPG2	Interphotoreceptor matrix proteoglycan 2	0.0196	-1.825

INIP	INTS3 and NABP interacting protein	0.000139	1.569
INO80	INO80 complex subunit	0.00025	-1.769
INPP4A	Inositol polyphosphate-4-phosphatase type I A	5.25E-05	-1.691
INSR	Insulin receptor	0.000508	-1.756
INTU	Inturned planar cell polarity protein	0.0152	-1.549
INVS	Inversin	0.000117	-1.585
IQCA1	IQ motif containing with AAA domain 1	0.0105	-1.937
IQCE	IQ motif containing E	0.000401	-1.93
IQGAP2	IQ motif containing gtpase activating protein 2	5.09E-05	-1.993
ISM1	Isthmin 1, angiogenesis inhibitor	0.00604	-1.898
ISOC1	Isochorismatase domain containing 1	0.00271	1.706
ITGA2	Integrin subunit alpha 2	0.00214	-1.742
ITGA4	Integrin subunit alpha 4	0.00682	-1.954
ITGA7	Integrin subunit alpha 7	0.00737	-1.724
ITGB4	Integrin subunit beta 4	0.00737	-1.639
ITIH5	Inter-alpha-trypsin inhibitor heavy chain family member 5	0.0156	-1.635
ITPR1	Inositol 1,4,5-trisphosphate receptor type 1	0.00824	-1.631
ITPR2	Inositol 1,4,5-trisphosphate receptor type 2	0.00151	-2.131
ITSN1	Intersectin 1	0.00479	-1.517
ITSN2	Intersectin 2	0.000021	-1.934
JAG1	Jagged 1	0.00239	-1.599
JAG2	Jagged 2	0.0164	-1.66
JMJD1C	Jumonji domain containing 1C	0.000389	-1.748
KALRN	Kalirin, rhogef kinase	0.000993	-1.812
KANK1	KN motif and ankyrin repeat domains 1	0.00113	-1.58
KANK2	KN motif and ankyrin repeat domains 2	0.00089	-1.578
KANSL1	KAT8 regulatory NSL complex subunit 1	0.00309	-1.521
KAT6A	Lysine acetyltransferase 6A	0.0017	-1.625
KAT6B	Lysine acetyltransferase 6B	0.000983	-1.926
KAZN	Kazrin, periplakin interacting protein	0.0108	-1.604

KCNK7	Potassium two pore domain channel subfamily K member 7	0.032	2.161
KCNMA1	Potassium calcium-activated channel subfamily M alpha 1	0.0146	-1.667
KDELC2	KDEL motif containing 2	3.18E-05	-1.665
KDELR3	KDEL endoplasmic reticulum protein retention receptor 3	0.00229	1.762
KDM2A	Lysine demethylase 2A	0.000597	-1.714
KDM2B	Lysine demethylase 2B	0.00453	-1.499
KDM4B	Lysine demethylase 4B	3.64E-06	-1.853
KDM5A	Lysine demethylase 5A	0.000266	-1.991
KDM6B	Lysine demethylase 6B	0.00832	-1.658
KIAA0226L	KIAA0226 like	0.0136	-1.992
KIAA0232	Kiaa0232	0.00048	-1.855
KIAA0319L	KIAA0319 like	0.00409	-1.5
KIAA0368	Kiaa0368	5.16E-05	-1.512
KIAA0430	Kiaa0430	0.00367	-1.531
KIAA0753	Kiaa0753	0.00136	-1.641
KIAA0922	Kiaa0922	0.000824	-1.66
KIAA1211	Kiaa1211	2.25E-06	-3.131
KIAA1217	Kiaa1217	0.000134	-1.797
KIAA1462	Kiaa1462	0.0226	-1.602
KIAA1551	Kiaa1551	0.00464	-1.642
KIAA1804	Mixed lineage kinase 4	0.0223	-1.537
KIAA2012	Kiaa2012	0.0161	-2.307
KIDINS220	Kinase D-interacting substrate 220kda	0.00167	-1.498
KIF13A	Kinesin family member 13A	6.79E-05	-1.97
KIF17	Kinesin family member 17	0.0163	-1.581
KIF20B	Kinesin family member 20B	0.00156	-2.108
KIF21A	Kinesin family member 21A	0.00123	-1.782
KIF24	Kinesin family member 24	0.0302	-1.502
KIF26A	Kinesin family member 26A	0.00758	-1.795
KIF27	Kinesin family member 27	2.17E-05	-2.537

KIF3A	Kinesin family member 3A	0.000625	-1.577
KIF3B	Kinesin family member 3B	1.18E-05	-1.564
KLF15	Kruppel like factor 15	0.0077	-1.598
KLHL14	Kelch like family member 14	0.0312	-1.668
KMT2A	Lysine methyltransferase 2A	0.00977	-1.901
KMT2B	Lysine methyltransferase 2B	0.00281	-1.679
KMT2C	Lysine methyltransferase 2C	0.00244	-1.816
KMT2E	Lysine methyltransferase 2E	0.000343	-1.806
KMT5A	Lysine methyltransferase 5A	0.000818	-1.67
KMT5C	Lysine methyltransferase 5C	0.0282	-1.515
KREMEN1	Kringle containing transmembrane protein 1	0.000714	-1.691
KRTCAP2	Keratinocyte associated protein 2	2.29E-05	1.72
KRTCAP3	Keratinocyte associated protein 3	6.93E-05	1.886
KSR2	Kinase suppressor of ras 2	0.00663	-1.851
Ktn1	Kinectin 1	7.48E-06	-1.918
KYAT3	Kynurenine aminotransferase 3	0.000379	1.687
LACC1	Laccase domain containing 1	1.27E-05	-1.819
LACTB2	Lactamase beta 2	0.000399	1.6
LAMA2	Laminin subunit alpha 2	0.0121	-1.522
LAMA4	Laminin subunit alpha 4	0.00884	-1.647
LAMTOR2	Late endosomal/lysosomal adaptor, MAPK and MTOR activator 2	4.93E-06	1.889
LAMTOR3	Late endosomal/lysosomal adaptor, MAPK and MTOR activator 3	3.37E-05	1.598
LAMTOR5	Late endosomal/lysosomal adaptor, MAPK and MTOR activator 5	4.66E-06	1.825
LAPTM4A	Lysosomal protein transmembrane 4 alpha	0.000052	1.783
LARP1	La ribonucleoprotein domain family member 1	0.00342	-1.651
LDHA	Lactate dehydrogenase A	0.00487	1.829
LDHB	Lactate dehydrogenase B	0.00213	1.518
LDLRAD4	Low density lipoprotein receptor class A domain containing 4	0.0187	-1.562
LENG8	Leukocyte receptor cluster member 8	0.009	-1.732
LEPROTL1	Leptin receptor overlapping transcript-like 1	0.000077	1.501

LGALS1	Galectin 1	0.000155	2.084
LGALS3	Lectin, galactoside binding soluble 3	0.000653	1.795
LGR4	Leucine rich repeat containing G protein-coupled receptor 4	0.00699	-1.568
LIPT1	Lipoyltransferase 1	1.18E-05	1.991
LLPH	LLP homolog, long-term synaptic facilitation	2.64E-05	1.779
LMO4	LIM domain only 4	0.00493	1.495
LMO7	LIM domain 7	0.00594	-1.495
LMTK2	Lemur tyrosine kinase 2	0.0122	-1.526
LOC728392	Uncharacterized LOC728392	0.0234	1.827
LPIN3	Lipin 3	0.00611	-1.531
LRIG1	Leucine rich repeats and immunoglobulin like domains 1	0.000297	-1.682
LRP2	LDL receptor related protein 2	0.00343	-2.39
LRP4	LDL receptor related protein 4	0.00596	-1.684
LRP5	LDL receptor related protein 5	0.000636	-1.619
LRRCC1	Leucine rich repeat and coiled-coil centrosomal protein 1	0.000117	-1.798
LRRFIP1	LRR binding FLII interacting protein 1	2.74E-05	-1.549
LRRIQ1	Leucine rich repeats and IQ motif containing 1	0.0139	-2.316
LRRK1	Leucine rich repeat kinase 1	0.00117	-1.572
LSM2	LSM2 homolog, U6 small nuclear RNA and mrna degradation associated	2.23E-05	1.637
LSM3	LSM3 homolog, U6 small nuclear RNA and mrna degradation associated	8.49E-07	2.06
LSM5	LSM5 homolog, U6 small nuclear RNA and mrna degradation associated	0.00466	1.614
LSM7	LSM7 homolog, U6 small nuclear RNA and mrna degradation associated	0.000108	1.724
LSM8	LSM8 homolog, U6 small nuclear RNA associated	1.51E-06	1.948
LTA4H	Leukotriene A4 hydrolase	0.000355	1.556
LTBP4	Latent transforming growth factor beta binding protein 4	0.00516	-1.591
LTF	Lactotransferrin	0.0242	2.233
LUC7L	LUC7 like	0.00244	-1.594
LUM	Lumican	0.00642	1.658
LUZP1	Leucine zipper protein 1	0.0202	-1.585
LXN	Latexin	0.0191	1.532

LY75	Lymphocyte antigen 75	0.0304	-1.586
LY96	Lymphocyte antigen 96	0.000125	1.922
LYRM5	LYR motif containing 5	0.0065	1.523
LYST	Lysosomal trafficking regulator	0.00579	-1.609
MACF1	Microtubule-actin crosslinking factor 1	0.000062	-2.264
MAGI1	Membrane associated guanylate kinase, WW and PDZ domain containing 1	8.53E-05	-2.128
MAGI3	Membrane associated guanylate kinase, WW and PDZ domain containing 3	0.000394	-1.71
MAGOH	Mago homolog, exon junction complex core component	0.000112	1.953
MAGOHB	Mago homolog B, exon junction complex core component	0.000206	1.838
MAL	Mal, T-cell differentiation protein	0.00145	1.674
MALT1	MALT1 paracaspase	0.000246	-1.581
MAML3	Mastermind like transcriptional coactivator 3	0.0258	-1.571
MAN2A2	Mannosidase alpha class 2A member 2	0.00169	-1.564
MANF	Mesencephalic astrocyte derived neurotrophic factor	0.00692	1.766
MAP10	Microtubule associated protein 10	0.00475	-1.503
MAP2K6	Mitogen-activated protein kinase kinase 6	0.00504	1.694
MAP3K13	Mitogen-activated protein kinase kinase kinase 13	8.44E-05	-2.099
MAP3K14	Mitogen-activated protein kinase kinase kinase 14	0.00117	-2.126
MAP3K3	Mitogen-activated protein kinase kinase kinase 3	0.00181	-1.627
MAP3K4	Mitogen-activated protein kinase kinase kinase 4	0.00224	-1.501
MAP4K4	Mitogen-activated protein kinase kinase kinase kinase 4	3.99E-05	-1.622
MAP7D3	MAP7 domain containing 3	0.000169	-1.978
MAPK8IP3	Mitogen-activated protein kinase 8 interacting protein 3	0.000466	-1.761
MAPKBP1	Mitogen-activated protein kinase binding protein 1	0.00477	-1.508
MARK1	Microtubule affinity regulating kinase 1	0.00126	-1.497
MARK3	Microtubule affinity regulating kinase 3	0.000057	-1.556
MASP2	Mannan binding lectin serine peptidase 2	0.0239	-1.524
MAST4	Microtubule associated serine/threonine kinase family member 4	0.0233	-1.704
MAT1A	Methionine adenosyltransferase 1A	0.00707	-2.311
MATN2	Matrilin 2	0.0266	-1.585

MBD6	Methyl-cpg binding domain protein 6	0.00686	-1.501
MBOAT2	Membrane bound O-acyltransferase domain containing 2	0.00634	1.517
MBTD1	Mbt domain containing 1	0.00199	-1.546
MBTPS1	Membrane bound transcription factor peptidase, site 1	0.0038	-1.558
MCEE	Methylmalonyl-coa epimerase	0.00121	1.654
MCTS1	MCTS1, re-initiation and release factor	1.37E-05	1.824
MDN1	Midasin AAA atpase 1	5.68E-05	-2.13
MDP1	Magnesium dependent phosphatase 1	0.000394	1.791
MED10	Mediator complex subunit 10	5.68E-06	1.709
MED13L	Mediator complex subunit 13 like	0.00153	-1.762
MED18	Mediator complex subunit 18	9.25E-06	1.676
MED21	Mediator complex subunit 21	0.000929	1.653
MED29	Mediator complex subunit 29	0.0011	1.557
MED30	Mediator complex subunit 30	0.000336	1.528
MED31	Mediator complex subunit 31	3.51E-05	1.66
MED6	Mediator complex subunit 6	0.000133	1.636
MEGF8	Multiple EGF like domains 8	0.000758	-1.951
MEGF9	Multiple EGF like domains 9	0.00863	-1.523
METTL1	Methyltransferase like 1	0.00341	1.789
METTL12	Methyltransferase like 12	0.000218	2.436
METTL21A	Methyltransferase like 21A	0.000738	1.663
METTL23	Methyltransferase like 23	0.000367	1.877
METTL5	Methyltransferase like 5	0.000755	1.647
MGA	MGA, MAX dimerization protein	0.00331	-1.869
MGST2	Microsomal glutathione S-transferase 2	0.00657	1.587
MGST3	Microsomal glutathione S-transferase 3	5.85E-07	2.116
MICAL2	Microtubule associated monooxygenase, calponin and LIM domain containing 2	0.0295	-1.566
MICAL3	Microtubule associated monooxygenase, calponin and LIM domain containing 3	0.00703	-1.655
MIS12	MIS12, kinetochore complex component	0.000595	1.576
MKI67	Marker of proliferation Ki-67	0.016	-3.024

MKL2	MKL1/myocardin like 2	0.00731	-1.577
MKNK2	MAP kinase interacting serine/threonine kinase 2	0.000781	-1.551
MLLT11	Myeloid/lymphoid or mixed-lineage leukemia; translocated to, 11	0.00309	1.812
MLLT3	MLLT3, super elongation complex subunit	0.000728	-1.657
MMADHC	Methylmalonic aciduria and homocystinuria, cbld type	1.64E-05	1.789
MMGT1	Membrane magnesium transporter 1	0.00581	1.553
MMP15	Matrix metallopeptidase 15	0.00804	-1.881
MMP2	Matrix metallopeptidase 2	0.00982	-1.52
MOK	MOK protein kinase	0.0154	-2.085
MORF4L2	Mortality factor 4 like 2	0.00136	1.502
MPC1	Mitochondrial pyruvate carrier 1	0.000818	1.668
MPC2	Mitochondrial pyruvate carrier 2	9.73E-06	1.835
MPDU1	Mannose-P-dolichol utilization defect 1	1.57E-05	1.689
MPDZ	Multiple PDZ domain crumbs cell polarity complex component	0.000342	-1.751
MPHOSPH6	M-phase phosphoprotein 6	0.00243	1.508
MPHOSPH9	M-phase phosphoprotein 9	6.21E-05	-1.784
MPRIP	Myosin phosphatase Rho interacting protein	0.000166	-1.545
MPV17	MPV17, mitochondrial inner membrane protein	3.28E-05	1.682
MPZL2	Myelin protein zero like 2	0.0214	1.825
MRPL1	Mitochondrial ribosomal protein L1	0.000711	1.518
MRPL10	Mitochondrial ribosomal protein L10	1.63E-05	1.649
MRPL11	Mitochondrial ribosomal protein L11	9.42E-05	1.713
MRPL13	Mitochondrial ribosomal protein L13	3.58E-05	1.857
MRPL15	Mitochondrial ribosomal protein L15	1.41E-05	1.825
MRPL16	Mitochondrial ribosomal protein L16	0.0075	1.515
MRPL17	Mitochondrial ribosomal protein L17	0.000047	1.728
MRPL18	Mitochondrial ribosomal protein L18	0.000226	1.516
MRPL20	Mitochondrial ribosomal protein L20	4.64E-06	1.714
MRPL21	Mitochondrial ribosomal protein L21	0.000114	1.683
MRPL22	Mitochondrial ribosomal protein L22	0.000468	1.569

MRPL24	Mitochondrial ribosomal protein L24	0.000412	1.613
MRPL27	Mitochondrial ribosomal protein L27	0.00065	1.691
MRPL3	Mitochondrial ribosomal protein L3	2.59E-05	1.958
MRPL30	Mitochondrial ribosomal protein L30	9.89E-06	1.741
MRPL32	Mitochondrial ribosomal protein L32	1.16E-06	2.251
MRPL34	Mitochondrial ribosomal protein L34	4.26E-06	1.794
MRPL35	Mitochondrial ribosomal protein L35	9.21E-06	2.029
MRPL39	Mitochondrial ribosomal protein L39	0.000021	1.743
MRPL42	Mitochondrial ribosomal protein L42	1.61E-06	1.981
MRPL44	Mitochondrial ribosomal protein L44	0.000228	1.751
MRPL45	Mitochondrial ribosomal protein L45	7.95E-06	1.792
MRPL46	Mitochondrial ribosomal protein L46	2.05E-05	1.759
MRPL47	Mitochondrial ribosomal protein L47	0.000594	1.764
MRPL48	Mitochondrial ribosomal protein L48	0.000115	1.698
MRPL50	Mitochondrial ribosomal protein L50	3.38E-05	2.053
MRPL52	Mitochondrial ribosomal protein L52	0.000432	1.53
MRPL54	Mitochondrial ribosomal protein L54	1.75E-05	1.565
MRPS12	Mitochondrial ribosomal protein S12	0.000457	1.579
MRPS14	Mitochondrial ribosomal protein S14	1.18E-05	1.988
MRPS16	Mitochondrial ribosomal protein S16	9.67E-05	1.698
MRPS17	Mitochondrial ribosomal protein S17	0.0004	1.829
MRPS18C	Mitochondrial ribosomal protein S18C	0.000913	1.605
MRPS21	Mitochondrial ribosomal protein S21	1.22E-05	1.751
MRPS22	Mitochondrial ribosomal protein S22	2.36E-05	1.625
MRPS23	Mitochondrial ribosomal protein S23	5.61E-05	1.689
MRPS24	Mitochondrial ribosomal protein S24	0.000213	1.595
MRPS28	Mitochondrial ribosomal protein S28	4.12E-05	1.887
MRPS30	Mitochondrial ribosomal protein S30	0.000336	1.53
MRPS33	Mitochondrial ribosomal protein S33	0.000057	1.688
MRPS35	Mitochondrial ribosomal protein S35	1.25E-05	1.766

MS4A8	Membrane spanning 4-domains A8	0.0181	2.089
MSI2	Musashi RNA binding protein 2	0.00179	-1.917
MSMO1	Methylsterol monooxygenase 1	0.00856	1.59
MSS51	MSS51 mitochondrial translational activator	0.00792	-1.758
MT-ATP6	ATP synthase F0 subunit 6	0.000326	2.655
MTCH2	Mitochondrial carrier 2	0.000176	1.525
MT-CO1	Cytochrome c oxidase subunit I	4.05E-05	2.547
MT-CO2	Cytochrome c oxidase subunit II	0.000104	2.848
MT-CO3	Cytochrome c oxidase III	8.11E-05	2.856
MT-CYB	Cytochrome b	7.28E-05	3.21
MTERF3	Mitochondrial transcription termination factor 3	0.00552	1.514
MTHFD2	Methylenetetrahydrofolate dehydrogenase (NADP+ dependent) 2, methenyltetrahydrofolate cyclohydrolase	0.00174	1.822
MT-ND1	NADH dehydrogenase, subunit 1 (complex I)	0.000444	2.797
MT-ND2	Mtnd2	0.00336	2.367
MT-ND3	NADH dehydrogenase, subunit 3 (complex I)	0.000304	2.885
MT-ND4	NADH dehydrogenase, subunit 4 (complex I)	0.00114	2.579
MT-ND4L	NADH dehydrogenase, subunit 4L (complex I)	0.000541	2.766
MT-ND5	NADH dehydrogenase, subunit 5 (complex I)	0.0109	2.063
MTOR	Mechanistic target of rapamycin	0.000183	-1.737
MTR	5-methyltetrahydrofolate-homocysteine methyltransferase	0.00398	-1.615
MTSS1	Metastasis suppressor 1	0.000997	-1.561
MTX2	metaxin 2	3.55E-05	1.658
MYCBP2	MYC binding protein 2, E3 ubiquitin protein ligase	0.000286	-1.866
MYDGF	myeloid derived growth factor	0.00234	1.536
MYH10	myosin, heavy chain 10, non-muscle	0.0183	-1.563
MYH11	myosin heavy chain 11	0.000997	-2.311
MYH14	myosin, heavy chain 14, non-muscle	0.00368	-1.801
MYL12A	myosin light chain 12A	0.000277	1.513
MYL12B	myosin light chain 12B	5.51E-05	1.661

MYL3	myosin light chain 3	0.023	2.032
MYL6	myosin light chain 6	0.000145	1.921
MYO10	myosin X	0.00128	-1.644
MYO9A	myosin IXA	0.0176	-1.687
MYO9B	myosin IXB	0.0005	-1.646
MYOZ1	myozenin 1	6.85E-05	2.102
MYRF	myelin regulatory factor	0.000899	-1.801
MZF1	myeloid zinc finger 1	0.000111	-2.084
N4BP3	NEDD4 binding protein 3	0.00299	-1.559
NAA20	N(alpha)-acetyltransferase 20, NatB catalytic subunit	0.000175	1.616
NAAA	N-acylethanolamine acid amidase	0.00532	1.54
NAE1	NEDD8 activating enzyme E1 subunit 1	6.53E-05	1.741
NANS	N-acetylneuraminate synthase	0.00177	1.557
NAPB	NSF attachment protein beta	0.00619	-1.745
NAT9	N-acetyltransferase 9 (putative)	0.000744	-1.626
NAV3	neuron navigator 3	0.012	-1.975
NAXE	NAD(P)HX epimerase	0.000773	1.52
NCBP2	nuclear cap binding protein subunit 2	2.01E-05	1.685
NCOA1	nuclear receptor coactivator 1	0.000331	-1.68
NCOA2	nuclear receptor coactivator 2	0.00468	-1.693
NCOA6	nuclear receptor coactivator 6	0.00572	-1.514
NCOR1	nuclear receptor corepressor 1	0.000185	-1.726
NCOR2	nuclear receptor corepressor 2	0.00315	-1.64
NDUFA1	NADH:ubiquinone oxidoreductase subunit A1	1.93E-06	1.997
NDUFA11	NADH:ubiquinone oxidoreductase subunit A11	0.00156	1.572
NDUFA12	NADH:ubiquinone oxidoreductase subunit A12	7.91E-08	2.374
NDUFA13	NADH:ubiquinone oxidoreductase subunit A13	0.000015	1.812
NDUFA2	NADH:ubiquinone oxidoreductase subunit A2	0.0021	1.584
NDUFA3	NADH:ubiquinone oxidoreductase subunit A3	0.0114	1.522
NDUFA4	NDUFA4, mitochondrial complex associated	2.40E-06	1.994

NDUFA5	NADH:ubiquinone oxidoreductase subunit A5	1.13E-05	1.961
NDUFA6	NADH:ubiquinone oxidoreductase subunit A6	1.25E-05	1.848
NDUFA7	NADH:ubiquinone oxidoreductase subunit A7	0.00047	1.538
NDUFA8	NADH:ubiquinone oxidoreductase subunit A8	7.01E-05	1.602
NDUFAB1	NADH:ubiquinone oxidoreductase subunit AB1	1.65E-05	1.758
NDUFAF2	NADH:ubiquinone oxidoreductase complex assembly factor 2	0.00285	1.617
NDUFAF4	NADH:ubiquinone oxidoreductase complex assembly factor 4	0.00145	1.867
NDUFAF5	NADH:ubiquinone oxidoreductase complex assembly factor 5	0.0177	1.744
NDUFB1	NADH:ubiquinone oxidoreductase subunit B1	2.87E-05	2.169
NDUFB11	NADH:ubiquinone oxidoreductase subunit B11	0.000014	1.976
NDUFB3	NADH:ubiquinone oxidoreductase subunit B3	1.72E-07	2.155
NDUFB4	NADH:ubiquinone oxidoreductase subunit B4	2.67E-05	1.927
NDUFB5	NADH:ubiquinone oxidoreductase subunit B5	5.12E-05	1.93
NDUFB6	NADH:ubiquinone oxidoreductase subunit B6	7.32E-07	2.116
NDUFB7	NADH:ubiquinone oxidoreductase subunit B7	0.00086	1.524
NDUFB8	NADH:ubiquinone oxidoreductase subunit B8	0.00406	1.5
NDUFB9	NADH:ubiquinone oxidoreductase subunit B9	9.22E-06	1.892
NDUFC1	NADH:ubiquinone oxidoreductase subunit C1	3.79E-05	1.994
NDUFC2	NADH:ubiquinone oxidoreductase subunit C2	6.21E-06	1.906
NDUFS2	NADH:ubiquinone oxidoreductase core subunit S2	0.000671	1.502
NDUFS3	NADH:ubiquinone oxidoreductase core subunit S3	0.000039	1.612
NDUFS4	NADH:ubiquinone oxidoreductase subunit S4	0.00182	1.726
Ndufs5	NADH dehydrogenase (ubiquinone) Fe-S protein 5	0.00012	1.776
NDUFS6	NADH:ubiquinone oxidoreductase subunit S6	0.00392	1.522
NEK1	NIMA related kinase 1	0.000154	-1.613
NEK8	NIMA related kinase 8	0.0271	-1.612
NEMF	nuclear export mediator factor	0.000245	-1.88
NETO2	neuropilin and tolloid like 2	0.0312	1.949
NEURL4	neuralized E3 ubiquitin protein ligase 4	0.00011	-1.826
NFKBIZ	NFKB inhibitor zeta	3.56E-05	-2.665

NFRKB	nuclear factor related to kappaB binding protein	0.000686	-1.615
NFU1	NFU1 iron-sulfur cluster scaffold	0.00096	1.608
NFYB	nuclear transcription factor Y subunit beta	0.00554	1.62
NHP2	NHP2 ribonucleoprotein	1.97E-05	1.972
NHS	NHS actin remodeling regulator	0.00607	-1.835
NHSL1	NHS like 1	0.0192	-1.571
NIFK	nucleolar protein interacting with the FHA domain of MKI67	7.58E-06	1.706
NIN	ninein	0.000279	-2.254
NIPAL3	NIPA like domain containing 3	0.000751	-1.673
NIPBL	NIPBL, cohesin loading factor	2.31E-05	-1.982
NIPSNAP3A	nipsnap homolog 3A	0.000591	1.684
NKPD1	NTPase, KAP family P-loop domain containing 1	0.0152	-2.332
NKTR	natural killer cell triggering receptor	5.57E-05	-2.246
NMB	neuromedin B	0.00386	2.271
NOL3	nucleolar protein 3	0.00187	1.766
NOL4L	nucleolar protein 4 like	0.00105	-1.737
NOL8	nucleolar protein 8	0.000245	-1.545
NOP10	NOP10 ribonucleoprotein	1.26E-05	1.643
NOP16	NOP16 nucleolar protein	0.000112	1.71
NOTCH1	notch 1	0.00265	-1.824
NOTCH3	notch 3	9.82E-05	-1.806
NOTCH4	notch 4	0.0298	-1.657
NPAT	nuclear protein, coactivator of histone transcription	0.000022	-2.222
NPC2	NPC intracellular cholesterol transporter 2	0.000232	1.72
NPM1	nucleophosmin	2.90E-06	2.007
NPM3	nucleophosmin/nucleoplasmin 3	0.000221	1.948
NPY1R	neuropeptide Y receptor Y1	0.00201	1.998
NQO2	NAD(P)H quinone dehydrogenase 2	0.00067	1.888
NR2C2	nuclear receptor subfamily 2 group C member 2	0.0011	-1.609
NREP	neuronal regeneration related protein	0.0281	1.593

NRIP1	nuclear receptor interacting protein 1	0.00137	-1.829
NRP1	neuropilin 1	0.0254	-1.592
NSMCE1	NSE1 homolog, SMC5-SMC6 complex component	1.44E-05	1.954
NT5C	5', 3'-nucleotidase, cytosolic	0.0145	1.862
NTAN1	N-terminal asparagine amidase	0.000365	1.56
NTN4	netrin 4	0.00276	-1.882
NUDCD2	NudC domain containing 2	3.41E-05	1.648
NUDT1	nudix hydrolase 1	0.00546	1.523
NUDT2	nudix hydrolase 2	0.000493	1.563
NUMA1	nuclear mitotic apparatus protein 1	0.00313	-1.631
NUP210L	nucleoporin 210 like	0.000102	1.693
NUP35	nucleoporin 35	1.02E-05	1.612
NUP93	nucleoporin 93	0.00745	1.9
NUTF2	nuclear transport factor 2	1.89E-05	1.676
NYAP1	neuronal tyrosine phosphorylated phosphoinositide-3-kinase adaptor 1	0.0203	-1.643
OARD1	O-acyl-ADP-ribose deacylase 1	0.00011	1.55
OAT	ornithine aminotransferase	0.00585	1.712
OCIAD2	OCIA domain containing 2	0.00675	1.609
ODC1	ornithine decarboxylase 1	0.0157	1.555
ODF2L	outer dense fiber of sperm tails 2 like	0.0012	-1.582
OFD1	OFD1, centriole and centriolar satellite protein	4.07E-05	-1.785
OLA1	Obg-like ATPase 1	3.87E-06	1.869
OLFML2B	olfactomedin like 2B	0.00454	-1.656
OMA1	OMA1 zinc metallopeptidase	0.00062	1.525
ORC5	origin recognition complex subunit 5	0.000589	1.588
ORMDL2	ORMDL sphingolipid biosynthesis regulator 2	8.87E-07	1.874
OSBPL8	oxysterol binding protein like 8	8.45E-05	-2.179
OSTC	oligosaccharyltransferase complex non-catalytic subunit	3.80E-06	2.083
OTUD4	OTU deubiquitinase 4	0.00237	-1.513
OXTR	oxytocin receptor	0.00367	6.441

PACS2	phosphofurin acidic cluster sorting protein 2	0.000732	-1.559
PAFAH1B3	platelet activating factor acetylhydrolase 1b catalytic subunit 3	8.18E-06	1.736
PAICS	phosphoribosylaminoimidazole carboxylase; phosphoribosylaminoimidazolesuccinocarboxamide synthase	0.000103	1.548
PALB2	partner and localizer of BRCA2	0.00488	-1.535
PALM3	paralemmin 3	0.0107	-2.139
PARD3B	par-3 family cell polarity regulator beta	0.0123	-1.591
PARL	presenilin associated rhomboid like	1.66E-05	1.681
PATJ	PATJ, crumbs cell polarity complex component	0.00819	-1.555
PAX2	paired box 2	0.00528	-1.893
PAXBP1	PAX3 and PAX7 binding protein 1	0.000315	-1.722
PBDC1	polysaccharide biosynthesis domain containing 1	0.000509	1.614
PBRM1	polybromo 1	0.000925	-1.517
PC	pyruvate carboxylase	0.00147	-1.73
PCF11	PCF11 cleavage and polyadenylation factor subunit	3.52E-05	-2.015
PCM1	pericentriolar material 1	3.64E-06	-2.102
PCNA	proliferating cell nuclear antigen	4.43E-05	1.657
PCNT	pericentrin	0.00138	-1.724
PDCD10	programmed cell death 10	0.000151	1.554
PDCD5	programmed cell death 5	3.87E-05	1.827
PDCL3	phosducin like 3	6.96E-05	1.747
PDE3B	phosphodiesterase 3B	0.00429	-1.792
PDE4DIP	phosphodiesterase 4D interacting protein	0.000031	-1.845
PDE6D	phosphodiesterase 6D	0.00049	2.022
PDF	peptide deformylase (mitochondrial)	0.00119	1.528
PDZD11	PDZ domain containing 11	0.000121	1.533
PDZD2	PDZ domain containing 2	0.00154	-1.974
PECR	peroxisomal trans-2-enoyl-CoA reductase	0.00266	1.69
PER1	period circadian clock 1	0.00268	-1.714
PEX2	peroxisomal biogenesis factor 2	0.000117	1.639

PFDN1	prefoldin subunit 1	2.26E-06	1.919
PFDN4	prefoldin subunit 4	3.05E-06	2.102
PFDN5	prefoldin subunit 5	1.45E-05	2.022
PFDN6	prefoldin subunit 6	0.000645	1.547
PFKFB2	6-phosphofructo-2-kinase/fructose-2,6-biphosphatase 2	0.0308	-1.62
PFN2	profilin 2	5.21E-06	2.011
PGD	phosphogluconate dehydrogenase	0.00168	1.504
PGK1	phosphoglycerate kinase 1	0.00108	1.762
PGM2	phosphoglucomutase 2	4.01E-05	-2.524
PHACTR1	phosphatase and actin regulator 1	0.00845	-1.603
PHB	prohibitin	0.00211	1.628
PHF20L1	PHD finger protein 20-like 1	0.00115	-1.615
PHF21A	PHD finger protein 21A	0.00182	-1.698
PHF3	PHD finger protein 3	1.28E-05	-2.083
PHF5A	PHD finger protein 5A	0.000108	1.746
PHIP	pleckstrin homology domain interacting protein	0.0287	-1.921
PHLDB2	pleckstrin homology like domain family B member 2	0.00165	-1.659
PHOSPHO2	phosphatase, orphan 2	0.000215	1.796
PI4KA	phosphatidylinositol 4-kinase alpha	0.000787	-1.713
PIGB	phosphatidylinositol glycan anchor biosynthesis class B	0.000208	1.539
PIGH	phosphatidylinositol glycan anchor biosynthesis class H	0.00101	1.551
PIGK	phosphatidylinositol glycan anchor biosynthesis class K	0.000821	1.571
PIGP	phosphatidylinositol glycan anchor biosynthesis class P	2.04E-06	2.014
PIGU	phosphatidylinositol glycan anchor biosynthesis class U	0.000172	1.509
PIGW	phosphatidylinositol glycan anchor biosynthesis class W	0.000772	1.759
PIK3CD	phosphatidylinositol-4,5-bisphosphate 3-kinase catalytic subunit delta	0.0206	-1.694
PIK3R5	phosphoinositide-3-kinase regulatory subunit 5	0.0154	-1.768
PIKFYVE	phosphoinositide kinase, FYVE-type zinc finger containing	0.0235	-1.544
PIN4	peptidylprolyl cis/trans isomerase, NIMA-interacting 4	0.00081	1.577
PIP5K1C	phosphatidylinositol-4-phosphate 5-kinase type 1 gamma	0.00643	-1.513

PKD1	polycystin 1, transient receptor potential channel interacting	0.00713	-1.783
PKD1L3	polycystin 1 like 3, transient receptor potential channel interacting	0.00427	-1.506
PLA2R1	phospholipase A2 receptor 1	0.0109	-1.909
PLCB3	phospholipase C beta 3	0.000196	-1.675
PLCB4	phospholipase C beta 4	0.00184	-1.803
PLCG1	phospholipase C gamma 1	0.000498	-1.821
PLCG2	phospholipase C gamma 2	0.011	-1.614
PLCH1	phospholipase C eta 1	0.00389	-1.597
PLCL1	phospholipase C like 1	0.0297	-1.525
PLEC	plectin	0.00473	-1.623
PLEKHA7	pleckstrin homology domain containing A7	0.00869	-1.68
PLEKHB2	pleckstrin homology domain containing B2	0.000665	1.557
PLEKHG1	pleckstrin homology and RhoGEF domain containing G1	0.0139	-1.718
PLEKHG3	pleckstrin homology and RhoGEF domain containing G3	0.000319	-1.605
PLEKHG5	pleckstrin homology and RhoGEF domain containing G5	0.00961	-1.574
PLEKHH1	pleckstrin homology, MyTH4 and FERM domain containing H1	0.00481	-1.883
PLEKHH2	pleckstrin homology, MyTH4 and FERM domain containing H2	0.0329	-1.627
PLEKHH3	pleckstrin homology, MyTH4 and FERM domain containing H3	0.00039	-1.724
PLEKHM3	pleckstrin homology domain containing M3	0.0112	-1.642
PLGRKT	plasminogen receptor with a C-terminal lysine	4.03E-05	1.844
PLIN2	perilipin 2	0.00788	1.681
PLK1	polo like kinase 1	0.00073	-2.088
PLP2	proteolipid protein 2	9.78E-06	1.887
PLXDC1	plexin domain containing 1	0.00863	-1.576
PLXNB1	plexin B1	0.000843	-1.563
PLXNB2	plexin B2	0.00123	-1.673
PLXNC1	plexin C1	0.00105	-1.879
PNN	pinin, desmosome associated protein	0.000958	-1.501
PNO1	partner of NOB1 homolog	3.28E-05	1.901
PNPLA7	patatin like phospholipase domain containing 7	0.00466	-1.554

PODN	podocan	0.00557	-1.731
POGZ	pogo transposable element with ZNF domain	0.000684	-1.528
POLE2	DNA polymerase epsilon 2, accessory subunit	0.0241	1.538
POLG	DNA polymerase gamma, catalytic subunit	0.00265	-1.514
POLR1E	RNA polymerase I subunit E	4.26E-06	1.574
POLR2G	RNA polymerase II subunit G	2.94E-05	1.69
POLR2H	RNA polymerase II subunit H	1.31E-05	2.044
POLR2I	RNA polymerase II subunit I	0.000171	1.631
POLR2K	RNA polymerase II subunit K	3.09E-05	1.928
POLR3A	RNA polymerase III subunit A	0.00235	-1.536
POLR3K	RNA polymerase III subunit K	0.000727	1.595
POLRMT	RNA polymerase mitochondrial	0.00365	-1.499
POMP	proteasome maturation protein	0.000852	1.504
PON2	paraoxonase 2	0.000313	1.591
POP5	POP5 homolog, ribonuclease P/MRP subunit	8.34E-07	1.995
POP7	POP7 homolog, ribonuclease P/MRP subunit	0.000109	1.8
POPDC3	popeye domain containing 3	0.0242	1.601
PPA2	pyrophosphatase (inorganic) 2	1.47E-05	1.829
PPARD	peroxisome proliferator activated receptor delta	0.000865	-1.594
PPFIA1	PTPRF interacting protein alpha 1	0.000294	-1.539
PPIB	peptidylprolyl isomerase B	0.00006	1.787
PPID	peptidylprolyl isomerase D	0.000188	1.591
PPIE	peptidylprolyl isomerase E	0.000631	1.576
PPIG	peptidylprolyl isomerase G	0.000942	-1.498
PPIH	peptidylprolyl isomerase H	9.44E-05	1.762
PPIL1	peptidylprolyl isomerase like 1	1.27E-05	1.753
PPIL3	peptidylprolyl isomerase like 3	0.000448	1.761
PPL	periplakin	0.0103	-1.669
PPM1K	protein phosphatase, Mg2+/Mn2+ dependent 1K	0.000684	-1.824
PPP1CC	protein phosphatase 1 catalytic subunit gamma	0.00141	1.517

PPP1R13B	protein phosphatase 1 regulatory subunit 13B	0.00462	-1.588
PPP1R26	protein phosphatase 1 regulatory subunit 26	0.00129	-1.763
PPP6R2	protein phosphatase 6 regulatory subunit 2	0.00293	-1.519
PPT1	palmitoyl-protein thioesterase 1	0.000791	1.584
PRDM10	PR domain 10	0.000727	-1.844
PRDM15	PR domain 15	0.0135	-1.585
PRDM2	PR domain 2	0.000871	-1.823
PRDX1	peroxiredoxin 1	3.44E-06	2.017
PRDX2	peroxiredoxin 2	0.00118	1.515
PRDX4	peroxiredoxin 4	0.000106	1.648
PRELID1	PRELI domain containing 1	0.00303	1.592
PRELID3B	PRELI domain containing 3B	0.000155	1.567
PRICKLE1	prickle planar cell polarity protein 1	0.00528	-1.524
PRKAG1	protein kinase AMP-activated non-catalytic subunit gamma 1	5.26E-05	1.581
PRKDC	protein kinase, DNA-activated, catalytic polypeptide	0.00295	-1.527
PRPF40A	pre-mRNA processing factor 40 homolog A	0.00191	-1.5
PRPS1	phosphoribosyl pyrophosphate synthetase 1	0.00018	1.632
PRR14L	proline rich 14 like	0.000678	-1.783
PRRC2B	proline rich coiled-coil 2B	0.00963	-1.571
PRRC2C	proline rich coiled-coil 2C	0.00395	-1.883
PRSS23	protease, serine 23	0.00476	1.908
PRX	periaxin	0.00909	-2.16
PSD4	pleckstrin and Sec7 domain containing 4	0.00353	-1.586
PSENEN	presenilin enhancer gamma-secretase subunit	5.44E-05	1.574
PSMA1	proteasome subunit alpha 1	6.58E-05	1.887
PSMA2	proteasome subunit alpha 2	0.000289	1.988
PSMA3	proteasome subunit alpha 3	0.000034	1.795
PSMA4	proteasome subunit alpha 4	0.0013	1.555
PSMA5	proteasome subunit alpha 5	0.000431	1.69
PSMA6	proteasome subunit alpha 6	1.27E-05	1.914

PSMA7	proteasome subunit alpha 7	0.000379	1.687
PSMB1	proteasome subunit beta 1	1.31E-06	1.916
PSMB2	proteasome subunit beta 2	0.000219	1.7
PSMB3	proteasome subunit beta 3	1.09E-05	1.983
PSMB4	proteasome subunit beta 4	3.60E-06	2.118
PSMB5	proteasome subunit beta 5	0.00118	1.503
PSMB6	proteasome subunit beta 6	2.86E-05	1.971
PSMB7	proteasome subunit beta 7	8.52E-05	1.781
PSMB8	proteasome subunit beta 8	0.032	1.495
PSMD14	proteasome 26S subunit, non-ATPase 14	0.000666	1.753
PSMD5	proteasome 26S subunit, non-ATPase 5	0.00025	1.53
PSME1	proteasome activator subunit 1	0.0103	1.533
PSME2	proteasome activator subunit 2	0.00686	1.618
PSMG2	proteasome assembly chaperone 2	4.45E-06	2.003
PSMG4	proteasome assembly chaperone 4	1.77E-06	2.201
PSPH	phosphoserine phosphatase	0.0154	2.917
PTGES	prostaglandin E synthase	0.00136	2.461
PTGR1	prostaglandin reductase 1	0.0286	1.897
PTHLH	parathyroid hormone like hormone	0.0096	3.115
PTK2B	protein tyrosine kinase 2 beta	0.000398	-1.908
PTPMT1	protein tyrosine phosphatase, mitochondrial 1	0.000675	1.839
PTPN12	protein tyrosine phosphatase, non-receptor type 12	0.00018	-1.524
PTPN13	protein tyrosine phosphatase, non-receptor type 13	0.000872	-1.922
PTPN6	protein tyrosine phosphatase, non-receptor type 6	0.000267	-1.581
PTPRB	protein tyrosine phosphatase, receptor type B	0.0161	-1.664
PTPRF	protein tyrosine phosphatase, receptor type F	0.00224	-1.606
PTPRM	protein tyrosine phosphatase, receptor type M	0.00075	-1.684
PTRH2	peptidyl-tRNA hydrolase 2	0.000292	1.773
PTRHD1	peptidyl-tRNA hydrolase domain containing 1	0.00178	1.527
PUS7	pseudouridylate synthase 7 (putative)	0.00308	1.499

PWP1	PWP1 homolog, endonuclein	0.000018	1.655
R3HDM2	R3H domain containing 2	0.0015	-1.564
RAB11A	RAB11A, member RAS oncogene family	0.000122	1.533
RAB11FIP2	RAB11 family interacting protein 2	0.000763	-1.565
RAB25	RAB25, member RAS oncogene family	0.00125	1.5
RAB31	RAB31, member RAS oncogene family	0.00404	1.596
RABAC1	Rab acceptor 1	0.00014	1.866
RABEP1	rabaptin, RAB GTPase binding effector protein 1	0.00321	-1.81
RABGGTB	Rab geranylgeranyltransferase beta subunit	0.00076	1.541
RABL3	RAB, member of RAS oncogene family like 3	0.000197	1.581
RACK1	receptor for activated C kinase 1	1.11E-06	2.218
RAD50	RAD50 double strand break repair protein	9.34E-05	-1.717
RAD51B	RAD51 paralog B	0.00133	1.645
RAD54L2	RAD54-like 2 (S. cerevisiae)	0.00883	-1.499
RADIL	Rap associating with DIL domain	0.00695	-1.832
RAI1	retinoic acid induced 1	0.000935	-1.716
RAI14	retinoic acid induced 14	0.00906	-1.563
RALGDS	ral guanine nucleotide dissociation stimulator	0.0046	-1.521
RALGPS1	Ral GEF with PH domain and SH3 binding motif 1	0.00494	-1.729
RANBP17	RAN binding protein 17	0.0303	-1.534
RAP1B	RAP1B, member of RAS oncogene family	0.00113	1.511
RAPGEF1	Rap guanine nucleotide exchange factor 1	0.00576	-1.55
RAPGEF6	Rap guanine nucleotide exchange factor 6	0.0115	-1.546
RARS	arginyl-tRNA synthetase	0.000195	1.568
RASA4	RAS p21 protein activator 4	0.00248	-1.747
RASGRP1	RAS guanyl releasing protein 1	0.00315	-2.058
RASSF4	Ras association domain family member 4	0.00898	-1.746
RB1CC1	RB1 inducible coiled-coil 1	3.14E-06	-1.676
RBBP6	RB binding protein 6, ubiquitin ligase	0.000188	-2.009
RBBP8	RB binding protein 8, endonuclease	0.000155	-1.583

RBKS	ribokinase	8.09E-05	1.895
RBM11	RNA binding motif protein 11	0.0102	1.572
RBM25	RNA binding motif protein 25	0.000286	-1.809
RBM27	RNA binding motif protein 27	0.00119	-1.497
RBM3	RNA binding motif (RNP1, RRM) protein 3	0.000538	2.064
RBM5	RNA binding motif protein 5	0.000826	-1.667
RBM6	RNA binding motif protein 6	0.000512	-1.573
RBP4	retinol binding protein 4	0.00897	1.592
Rbx1	ring-box 1	0.000301	1.603
RCL1	RNA terminal phosphate cyclase like 1	0.000229	1.567
RCOR1	REST corepressor 1	0.00093	-1.646
RDH14	retinol dehydrogenase 14 (all-trans/9-cis/11-cis)	2.48E-05	1.614
RELL2	RELT like 2	0.00292	-1.53
RER1	retention in endoplasmic reticulum sorting receptor 1	7.80E-06	1.668
REV3L	REV3 like, DNA directed polymerase zeta catalytic subunit	0.000375	-1.836
REXO1	RNA exonuclease 1 homolog	0.000783	-1.562
REXO2	RNA exonuclease 2	0.00477	1.701
RFC1	replication factor C subunit 1	0.00198	-1.518
RFC3	replication factor C subunit 3	2.32E-05	1.849
RFC4	replication factor C subunit 4	6.21E-05	1.835
RFFL	ring finger and FYVE-like domain containing E3 ubiquitin protein ligase	0.00443	-1.602
RFK	riboflavin kinase	0.000355	1.528
RFX2	regulatory factor X2	0.0311	-1.645
RFX7	regulatory factor X7	0.00082	-1.635
RGS6	regulator of G-protein signaling 6	0.0166	-1.882
RHBDF2	rhomboid 5 homolog 2	0.0131	-1.511
RHEB	Ras homolog enriched in brain	0.000105	1.831
RHOC	ras homolog family member C	0.000534	1.575
RICTOR	RPTOR independent companion of MTOR complex 2	0.00188	-1.652
RIF1	replication timing regulatory factor 1	0.00348	-1.916

RMDN1	regulator of microtubule dynamics 1	0.00115	1.5
RNASE4	ribonuclease A family member 4	0.00551	1.547
RNF138	ring finger protein 138	0.000804	1.598
RNF168	ring finger protein 168	7.36E-05	-1.642
RNF181	ring finger protein 181	0.000185	1.66
RNF34	ring finger protein 34	0.000844	1.552
RNF43	ring finger protein 43	0.00856	-1.894
RNF6	ring finger protein 6	0.00173	-1.559
RNF7	ring finger protein 7	0.000734	1.562
ROCK1	Rho associated coiled-coil containing protein kinase 1	4.12E-05	-1.932
ROCK2	Rho associated coiled-coil containing protein kinase 2	0.00102	-1.788
ROMO1	reactive oxygen species modulator 1	1.28E-05	1.864
RORC	RAR related orphan receptor C	0.0197	-1.594
RPA3	replication protein A3	0.000689	1.665
RPF2	ribosome production factor 2 homolog	0.000539	1.673
RPGRIP1L	RPGRIP1 like	0.0011	-1.915
RPL10	ribosomal protein L10	0.00013	1.76
RPL10A	ribosomal protein L10a	4.03E-06	2.11
RPL11	ribosomal protein L11	1.06E-05	1.961
RPL13	ribosomal protein L13	9.21E-07	2.133
RPL13A	ribosomal protein L13a	7.84E-06	2.188
RPL14	ribosomal protein L14	2.75E-05	1.909
RPL15	ribosomal protein L15	5.59E-05	1.929
RPL17	ribosomal protein L17	3.19E-05	2.102
RPL18	ribosomal protein L18	0.000084	1.776
RPL18A	ribosomal protein L18a	3.97E-05	1.804
RPL21	ribosomal protein L21	1.37E-06	2.288
RPL22	ribosomal protein L22	1.47E-06	2.312
Rpl22l1	ribosomal protein L22 like 1	2.19E-05	1.843
RPL23	ribosomal protein L23	3.63E-06	2.408

RPL24	ribosomal protein L24	6.12E-05	1.961
RPL26	ribosomal protein L26	3.62E-06	2.271
RPL27	ribosomal protein L27	1.25E-07	2.694
RPL27A	ribosomal protein L27a	9.11E-06	2.247
RPL28	ribosomal protein L28	0.000198	1.629
RPL29	ribosomal protein L29	0.000034	1.806
RPL3	ribosomal protein L3	1.96E-05	2.015
RPL30	ribosomal protein L30	1.34E-06	2.432
RPL31	ribosomal protein L31	9.05E-06	2.291
RPL32	ribosomal protein L32	9.84E-07	2.316
RPL35	ribosomal protein L35	1.25E-05	2.029
RPL35A	ribosomal protein L35a	6.80E-06	2.447
RPL36	ribosomal protein L36	2.48E-06	1.921
RPL36A	ribosomal protein L36a	9.84E-08	2.768
RPL37	ribosomal protein L37	2.69E-07	2.866
RPL37A	ribosomal protein L37a	6.22E-06	2.257
RPL38	ribosomal protein L38	4.96E-06	1.997
RPL5	ribosomal protein L5	1.22E-05	1.933
RPL6	ribosomal protein L6	9.71E-05	1.691
RPL7	ribosomal protein L7	4.63E-05	1.868
RPL7A	ribosomal protein L7a	9.87E-06	1.939
RPL8	ribosomal protein L8	1.02E-06	2.122
RPLP0	ribosomal protein lateral stalk subunit P0	3.69E-07	2.274
RPLP1	ribosomal protein lateral stalk subunit P1	6.60E-08	2.398
RPLP2	ribosomal protein lateral stalk subunit P2	4.05E-07	2.575
RPP30	ribonuclease P/MRP subunit p30	0.00029	1.5
RPP38	ribonuclease P/MRP subunit p38	0.00311	1.503
RPS10	ribosomal protein S10	2.56E-06	2.246
RPS11	ribosomal protein S11	0.000011	2.069
RPS12	ribosomal protein S12	1.44E-06	2.424

RPS13	ribosomal protein S13	4.56E-05	1.821
RPS14	ribosomal protein S14	1.86E-05	2.122
RPS15	ribosomal protein S15	9.16E-05	1.953
RPS16	ribosomal protein S16	9.41E-07	1.975
RPS17	ribosomal protein S17	3.90E-06	2.561
RPS18	ribosomal protein S18	1.65E-06	2.206
RPS19	ribosomal protein S19	2.28E-06	2.22
RPS2	ribosomal protein S2	2.98E-05	1.956
RPS20	ribosomal protein S20	4.20E-07	2.556
RPS21	ribosomal protein S21	9.63E-07	2.781
RPS24	ribosomal protein S24	7.23E-07	2.49
RPS25	ribosomal protein S25	2.67E-07	2.586
RPS26	ribosomal protein S26	5.58E-05	2.236
RPS27	ribosomal protein S27	5.64E-07	2.451
RPS27A	ribosomal protein S27a	4.19E-06	2.329
RPS28	ribosomal protein S28	0.00001	2.068
RPS29	ribosomal protein S29	0.00253	2.005
RPS3	ribosomal protein S3	7.45E-07	2.406
RPS3A	ribosomal protein S3A	0.000306	1.947
RPS4X	ribosomal protein S4, X-linked	4.16E-05	2.247
RPS4Y2	ribosomal protein S4, Y-linked 2	2.14E-05	1.918
RPS5	ribosomal protein S5	1.44E-05	1.978
RPS6	ribosomal protein S6	0.000134	1.827
RPS6KA1	ribosomal protein S6 kinase A1	0.00149	-1.518
RPS6KA6	ribosomal protein S6 kinase A6	0.000355	-1.568
RPS7	ribosomal protein S7	2.76E-05	2.115
RPS8	ribosomal protein S8	3.53E-05	2.023
RPS9	ribosomal protein S9	7.62E-05	1.872
RPSA	ribosomal protein SA	3.43E-05	1.904
RRAGA	Ras related GTP binding A	7.83E-05	1.672

RREB1	ras responsive element binding protein 1	0.00508	-1.732
RSF1	remodeling and spacing factor 1	0.000129	-2.022
RSL24D1	ribosomal L24 domain containing 1	5.98E-05	1.585
RUBCN	RUN and cysteine rich domain containing beclin 1 interacting protein	0.00285	-1.63
RUSC2	RUN and SH3 domain containing 2	0.0056	-1.637
S100A11	S100 calcium binding protein A11	0.00288	1.584
S100A13	S100 calcium binding protein A13	0.000392	1.84
S100A2	S100 calcium binding protein A2	0.00494	2.166
S100A4	S100 calcium binding protein A4	0.0102	1.584
SAA1	serum amyloid A1	0.0103	-3.173
SACS	sacsin molecular chaperone	0.0278	-1.682
SAFB	scaffold attachment factor B	0.000117	-1.635
SAFB2	scaffold attachment factor B2	0.00715	-1.496
SALL1	spalt like transcription factor 1	0.00148	-1.786
SAMM50	SAMM50 sorting and assembly machinery component	1.93E-05	1.607
SAR1A	secretion associated Ras related GTPase 1A	0.00011	1.701
SATB1	SATB homeobox 1	0.00145	-1.887
SBF1	SET binding factor 1	0.00111	-1.676
SBNO2	strawberry notch homolog 2	0.00561	-1.505
SBSPON	somatomedin B and thrombospondin type 1 domain containing	0.0164	1.686
SCAF11	SR-related CTD associated factor 11	4.23E-05	-2.033
SCAND1	SCAN domain containing 1	5.77E-05	1.846
SCAPER	S-phase cyclin A associated protein in the ER	5.53E-06	-1.969
SCARA5	scavenger receptor class A member 5	0.00226	-1.654
SCCPDH	saccharopine dehydrogenase (putative)	1.17E-05	1.727
SCRN1	secernin 1	0.0147	-1.528
SCUBE1	signal peptide, CUB domain and EGF like domain containing 1	0.00932	-2.295
SDCBP	syndecan binding protein	7.61E-05	1.672
SDF2	stromal cell derived factor 2	0.000496	1.676
SDHAF2	succinate dehydrogenase complex assembly factor 2	0.000869	1.529

SDHAF3	succinate dehydrogenase complex assembly factor 3	0.00935	1.62
SDHC	succinate dehydrogenase complex subunit C	0.00641	1.638
SDHD	succinate dehydrogenase complex subunit D	1.75E-05	1.749
SDK1	sidekick cell adhesion molecule 1	7.47E-05	-1.995
SEC11A	SEC11 homolog A, signal peptidase complex subunit	1.33E-05	2.04
SEC11C	SEC11 homolog C, signal peptidase complex subunit	0.000107	2.161
SEC31B	SEC31 homolog B, COPII coat complex component	0.0134	-1.507
SEC61B	Sec61 translocon beta subunit	0.000005	1.939
SEC61G	Sec61 translocon gamma subunit	0.00286	1.692
SELENBP1	selenium binding protein 1	0.0179	1.58
SELK	selenoprotein K	2.43E-05	1.827
SEMA3C	semaphorin 3C	0.00969	-1.529
SEMA3F	semaphorin 3F	0.000554	-1.92
SEMA4G	semaphorin 4G	0.00777	-1.615
SENP6	SUMO1/sentrin specific peptidase 6	1.32E-05	-1.757
SEPW1	selenoprotein W, 1	0.00739	1.55
SERF2	small EDRK-rich factor 2	0.00104	1.658
SERGEF	secretion regulating guanine nucleotide exchange factor	0.000223	1.913
SERPINB8	serpin family B member 8	0.0129	1.842
SERTAD2	SERTA domain containing 2	0.029	-1.518
SETBP1	SET binding protein 1	0.0136	-1.759
SETD2	SET domain containing 2	1.09E-05	-1.972
SETD5	SET domain containing 5	0.00026	-1.811
SETD6	SET domain containing 6	0.00272	-1.542
SETD9	SET domain containing 9	0.00416	1.729
SETX	senataxin	3.22E-05	-2.623
SF3B5	splicing factor 3b subunit 5	0.000252	1.589
SF3B6	splicing factor 3b subunit 6	2.53E-06	1.893
SFI1	SFI1 centrin binding protein	0.0164	-1.572
SFR1	SWI5 dependent homologous recombination repair protein 1	0.000939	1.879

SFSWAP	splicing factor SWAP homolog	0.000561	-1.693
SFXN4	sideroflexin 4	0.000019	1.911
SGSM1	small G protein signaling modulator 1	0.00296	-1.794
SGSM2	small G protein signaling modulator 2	0.000551	-1.625
SH3PXD2A	SH3 and PX domains 2A	0.0198	-1.543
SH3PXD2B	SH3 and PX domains 2B	0.00462	-1.706
SH3TC1	SH3 domain and tetratricopeptide repeats 1	0.000221	-1.897
SHANK2	SH3 and multiple ankyrin repeat domains 2	0.0037	-2.094
SHPRH	SNF2 histone linker PHD RING helicase	0.000883	-1.563
SHTN1	shootin 1	0.00233	-1.522
SIK2	salt inducible kinase 2	0.00157	-1.643
SIN3A	SIN3 transcription regulator family member A	0.000204	-1.624
SIN3B	SIN3 transcription regulator family member B	0.000193	-1.693
SIPA1L1	signal induced proliferation associated 1 like 1	0.013	-1.53
SIPA1L2	signal induced proliferation associated 1 like 2	0.000273	-1.614
SKA2	spindle and kinetochore associated complex subunit 2	0.00137	1.603
SLC12A7	solute carrier family 12 member 7	0.00179	-1.546
SLC16A1	solute carrier family 16 member 1	0.0025	2.363
SLC16A12	solute carrier family 16 member 12	0.0299	-2.693
SLC1A5	solute carrier family 1 member 5	0.00381	1.922
SLC25A17	solute carrier family 25 member 17	3.08E-05	1.523
SLC25A20	solute carrier family 25 member 20	0.000732	1.757
SLC25A24	solute carrier family 25 member 24	0.00386	1.529
SLC25A3	solute carrier family 25 member 3	0.00358	1.556
SLC25A33	solute carrier family 25 member 33	0.0197	1.503
SLC25A40	solute carrier family 25 member 40	0.0095	-1.51
SLC25A5	solute carrier family 25 member 5	0.00315	2.05
SLC27A3	solute carrier family 27 member 3	0.00165	3.34
SLC34A2	solute carrier family 34 member 2	0.0201	-1.983
SLC35B1	solute carrier family 35 member B1	0.000383	1.707

SLC39A1	solute carrier family 39 member 1	0.000118	1.601
SLC40A1	solute carrier family 40 member 1	0.00869	1.983
SLC45A2	solute carrier family 45 member 2	7.55E-05	3.63
SLC47A1	solute carrier family 47 member 1	0.0108	-1.512
SLC50A1	solute carrier family 50 member 1	0.00148	1.684
SLC5A9	solute carrier family 5 member 9	0.018	-1.575
SLIRP	SRA stem-loop interacting RNA binding protein	2.27E-06	2.304
SLIT3	slit guidance ligand 3	0.000442	-1.699
SLITRK2	SLIT and NTRK like family member 2	0.00816	-1.932
SLK	STE20 like kinase	0.0017	-1.775
SLTM	SAFB like transcription modulator	0.000132	-1.725
SLX4	SLX4 structure-specific endonuclease subunit	0.00409	-1.651
SMARCA4	SWI/SNF related, matrix associated, actin dependent regulator of chromatin, subfamily a, member 4	0.00127	-1.709
SMARCC1	SWI/SNF related, matrix associated, actin dependent regulator of chromatin subfamily c member 1	0.00086	-1.602
SMC1A	structural maintenance of chromosomes 1A	0.000678	-1.789
SMC2	structural maintenance of chromosomes 2	0.000026	-1.947
SMC3	structural maintenance of chromosomes 3	0.000106	-1.645
SMC4	structural maintenance of chromosomes 4	5.73E-06	-2.303
SMC5	structural maintenance of chromosomes 5	0.00126	-1.668
SMC6	structural maintenance of chromosomes 6	3.16E-05	-1.686
SMDT1	single-pass membrane protein with aspartate rich tail 1	1.68E-05	1.729
SMG1	SMG1, nonsense mediated mRNA decay associated PI3K related kinase	0.00244	-2.185
SMG6	SMG6, nonsense mediated mRNA decay factor	0.000806	-1.627
SMIM20	small integral membrane protein 20	0.000708	1.715
SMIM4	small integral membrane protein 4	8.91E-05	1.861
SMIM7	small integral membrane protein 7	8.10E-07	1.713
SMIM8	small integral membrane protein 8	0.000115	1.975
SMPDL3A	sphingomyelin phosphodiesterase acid like 3A	0.0221	1.875
SMURF1	SMAD specific E3 ubiquitin protein ligase 1	0.0002	-1.718
SNAI2	snail family transcriptional repressor 2	0.0076	2.079

SNAPC4	small nuclear RNA activating complex polypeptide 4	0.00295	-1.728
SNRNP25	small nuclear ribonucleoprotein U11/U12 subunit 25	0.0112	1.534
SNRNP27	small nuclear ribonucleoprotein U4/U6.U5 subunit 27	0.00272	1.581
SNRPB	small nuclear ribonucleoprotein polypeptides B and B1	0.00045	1.577
SNRPB2	small nuclear ribonucleoprotein polypeptide B2	1.47E-06	1.876
Snrpc	U1 small nuclear ribonucleoprotein C	5.46E-05	1.553
SNRPD1	small nuclear ribonucleoprotein D1 polypeptide	4.13E-06	1.843
SNRPD2	small nuclear ribonucleoprotein D2 polypeptide	5.61E-06	1.838
SNRPD3	small nuclear ribonucleoprotein D3 polypeptide	0.0005	1.519
SNRPF	small nuclear ribonucleoprotein polypeptide F	1.37E-05	2.165
SNRPG	small nuclear ribonucleoprotein polypeptide G	1.47E-06	2.92
SNU13	SNU13 homolog, small nuclear ribonucleoprotein (U4/U6.U5)	3.47E-05	1.628
SNX7	sorting nexin 7	0.00954	1.51
SOAT1	sterol O-acyltransferase 1	0.00145	2.744
SOCS2	suppressor of cytokine signaling 2	0.00497	1.555
SON	SON DNA binding protein	0.00011	-1.504
SORBS1	sorbin and SH3 domain containing 1	0.000252	-1.87
SORL1	sortilin related receptor 1	0.00488	-1.618
SOS1	SOS Ras/Rac guanine nucleotide exchange factor 1	0.000867	-1.613
SOS2	SOS Ras/Rho guanine nucleotide exchange factor 2	0.000599	-1.561
SOX13	SRY-box 13	0.00534	-1.558
SOX6	SRY-box 6	0.0038	-2.36
SPAG9	sperm associated antigen 9	0.000527	-1.601
SPARCL1	SPARC like 1	0.00906	-1.587
SPCS1	signal peptidase complex subunit 1	4.99E-06	1.643
SPCS2	signal peptidase complex subunit 2	1.21E-05	1.717
SPECC1	sperm antigen with calponin homology and coiled-coil domains 1	0.0138	-1.552
SPECC1L	sperm antigen with calponin homology and coiled-coil domains 1 like	6.28E-05	-1.879
SPEF2	sperm flagellar 2	0.0074	-2.396
SPEG	SPEG complex locus	0.012	-1.984

SPEN	spen family transcriptional repressor	0.000567	-2.239
SPG21	spastic paraplegia 21 (autosomal recessive, Mast syndrome)	0.000111	1.649
SPOCK2	sparc/osteonectin, cwcv and kazal-like domains proteoglycan (testican) 2	0.0234	-1.519
SPTAN1	spectrin alpha, non-erythrocytic 1	0.00202	-1.618
SPTB	spectrin beta, erythrocytic	0.0173	-2.331
SPTBN1	spectrin beta, non-erythrocytic 1	0.000857	-1.789
SPTSSA	serine palmitoyltransferase small subunit A	0.00231	1.67
SPX	spexin hormone	0.000958	1.688
SRA1	steroid receptor RNA activator 1	0.00422	1.511
SRCAP	Snf2-related CREBBP activator protein	0.00551	-1.568
SRGAP2	SLIT-ROBO Rho GTPase activating protein 2	0.0025	-1.673
SRI	sorcin	0.00139	1.58
SRP14	signal recognition particle 14	2.36E-05	2.043
SRPK2	SRSF protein kinase 2	0.000397	-1.725
SRPRB	SRP receptor beta subunit	1.61E-06	2.124
SSBP1	single stranded DNA binding protein 1	0.000492	1.531
SSR2	signal sequence receptor subunit 2	1.25E-06	2.096
SSR3	signal sequence receptor subunit 3	0.000173	1.907
SSR4	signal sequence receptor subunit 4	1.53E-05	2.092
STAB1	stabilin 1	0.023	-1.739
STARD13	StAR related lipid transfer domain containing 13	0.00124	-1.55
STEAP1	six transmembrane epithelial antigen of the prostate 1	0.000413	2.346
STK31	serine/threonine kinase 31	0.00122	-2.387
STK36	serine/threonine kinase 36	0.00101	-1.659
STMN1	stathmin 1	0.000695	1.583
STRADB	STE20-related kinase adaptor beta	0.00047	1.717
STT3A	STT3A, catalytic subunit of the oligosaccharyltransferase complex	0.00111	1.505
STX8	syntaxin 8	0.000127	1.687
STXBP5	syntaxin binding protein 5	0.00702	-1.572
SUCLG1	succinate-CoA ligase alpha subunit	9.12E-05	1.613

SUCO	SUN domain containing ossification factor	0.00103	-1.521
SULF2	sulfatase 2	0.00309	-1.597
SUMO1	small ubiquitin-like modifier 1	0.000399	1.526
SUMO2	small ubiquitin-like modifier 2	0.000168	1.904
SUPT4H1	SPT4 homolog, DSIF elongation factor subunit	4.19E-05	1.583
SUPT6H	SPT6 homolog, histone chaperone	0.00106	-1.542
SUSD2	sushi domain containing 2	0.021	-2.273
SUSD5	sushi domain containing 5	0.0235	-1.642
SWI5	SWI5 homologous recombination repair protein	7.43E-05	1.669
SWT1	SWT1, RNA endoribonuclease homolog	0.00161	-1.503
SYDE2	synapse defective Rho GTPase homolog 2	0.0154	-1.58
SYNC	syncoilin, intermediate filament protein	0.0036	-1.543
SYNE2	spectrin repeat containing nuclear envelope protein 2	0.000698	-2.099
SYNE3	spectrin repeat containing nuclear envelope family member 3	0.00796	-1.572
SYNJ2	synaptojanin 2	0.0054	-1.83
SYNM	synemin	0.0128	-1.517
SYT7	synaptotagmin 7	0.00601	-1.774
SYTL2	synaptotagmin like 2	0.0104	-1.541
SZT2	seizure threshold 2 homolog (mouse)	0.000611	-2.161
TACC1	transforming acidic coiled-coil containing protein 1	0.00446	-1.678
TAF1	TATA-box binding protein associated factor 1	4.34E-05	-1.858
TAF5	TATA-box binding protein associated factor 5	0.00353	-1.536
TAF8	TATA-box binding protein associated factor 8	0.000195	-1.531
TAF9	TATA-box binding protein associated factor 9	0.000231	1.544
TAMM41	TAM41 mitochondrial translocator assembly and maintenance homolog	0.00329	1.522
TANC1	tetratricopeptide repeat, ankyrin repeat and coiled-coil containing 1	0.000463	-1.769
TANC2	tetratricopeptide repeat, ankyrin repeat and coiled-coil containing 2	0.0121	-1.689
TAOK2	TAO kinase 2	1.18E-05	-1.841
TATDN1	TatD DNase domain containing 1	7.91E-05	2.176
TAX1BP1	Tax1 binding protein 1	0.000283	-1.584

TBC1D16	TBC1 domain family member 16	0.000709	-2.003
TBC1D30	TBC1 domain family member 30	0.00583	-1.663
TBC1D7	TBC1 domain family member 7	0.00108	1.754
TBC1D9	TBC1 domain family member 9	0.00202	-1.605
TBCA	tubulin folding cofactor A	5.32E-05	1.676
TBCB	tubulin folding cofactor B	0.000105	1.656
TCAF1	TRPM8 channel associated factor 1	0.00676	-1.497
TCEB2	transcription elongation factor B subunit 2	0.00076	1.585
TCF20	transcription factor 20	0.000288	-2.087
TCOF1	treacle ribosome biogenesis factor 1	0.00369	-1.607
TCP1	t-complex 1	0.000222	1.559
TCTN2	tectonic family member 2	0.00525	1.695
TDP2	tyrosyl-DNA phosphodiesterase 2	0.00058	1.501
TECPR2	tectonin beta-propeller repeat containing 2	0.000369	-1.734
TELO2	telomere maintenance 2	0.0083	-1.676
TENM3	teneurin transmembrane protein 3	0.00161	-2.147
TEP1	telomerase associated protein 1	9.38E-05	-1.929
TET2	tet methylcytosine dioxygenase 2	0.00223	-2.066
TEX11	testis expressed 11	0.00137	1.693
TFB2M	transcription factor B2, mitochondrial	0.000154	1.731
THEM4	thioesterase superfamily member 4	0.00531	1.641
THNSL2	threonine synthase like 2	0.00401	-1.637
THOC2	THO complex 2	0.00295	-1.512
THOC3	THO complex 3	0.000413	1.547
THRAP3	thyroid hormone receptor associated protein 3	0.000188	-1.708
TIMM17A	translocase of inner mitochondrial membrane 17 homolog A (yeast)	0.00205	1.787
TIMM23	translocase of inner mitochondrial membrane 23	0.000627	1.678
TIMM8A	translocase of inner mitochondrial membrane 8 homolog A (yeast)	3.31E-05	1.901
TIMM8B	translocase of inner mitochondrial membrane 8 homolog B	0.000397	1.527
TLCD1	TLC domain containing 1	0.00471	1.581

TM2D1	TM2 domain containing 1	0.000199	1.622
TM4SF1	transmembrane 4 L six family member 1	0.0218	1.79
TM6SF2	transmembrane 6 superfamily member 2	0.00159	2.363
TMA16	translation machinery associated 16 homolog	0.000258	1.62
TMBIM4	transmembrane BAX inhibitor motif containing 4	0.00399	1.503
TMBIM6	transmembrane BAX inhibitor motif containing 6	0.000485	1.577
TMCC2	transmembrane and coiled-coil domain family 2	0.00243	-1.84
TMCO1	transmembrane and coiled-coil domains 1	2.63E-06	1.68
TMED2	transmembrane p24 trafficking protein 2	2.66E-06	1.835
TMEM100	transmembrane protein 100	0.00485	1.823
TMEM126A	transmembrane protein 126A	0.000182	1.854
TMEM126B	transmembrane protein 126B	0.00333	1.626
TMEM128	transmembrane protein 128	0.000264	1.635
TMEM147	transmembrane protein 147	0.000091	1.564
TMEM14A	transmembrane protein 14A	6.61E-05	1.823
TMEM14C	transmembrane protein 14C	6.01E-05	1.799
TMEM184C	transmembrane protein 184C	0.000657	1.518
TMEM208	transmembrane protein 208	6.49E-07	2.195
TMEM220	transmembrane protein 220	0.0201	1.659
TMEM230	transmembrane protein 230	8.24E-07	1.748
TMEM256	transmembrane protein 256	1.45E-06	2.085
TMEM258	transmembrane protein 258	0.000213	1.663
TMEM40	transmembrane protein 40	0.00588	4.853
TMEM50A	transmembrane protein 50A	0.000974	1.64
TMEM50B	transmembrane protein 50B	0.000631	1.684
TMEM60	transmembrane protein 60	0.000157	1.838
TMEM64	transmembrane protein 64	0.00492	1.908
TMEM70	transmembrane protein 70	4.86E-05	1.841
TMEM94	transmembrane protein 94	0.00248	-1.528
TMEM9B	TMEM9 domain family member B	6.59E-05	1.671

TMF1	TATA element modulatory factor 1	6.43E-06	-1.841
TMPRSS2	transmembrane protease, serine 2	0.0107	-1.733
TMPRSS6	transmembrane protease, serine 6	0.00998	-1.731
TNFRSF4	TNF receptor superfamily member 4	0.0283	-2.103
TNMD	tenomodulin	0.0218	1.64
TNRC18	trinucleotide repeat containing 18	0.00464	-1.644
TNRC6A	trinucleotide repeat containing 6A	0.000168	-2.057
TNRC6C	trinucleotide repeat containing 6C	0.00172	-1.913
TOM1L2	target of myb1 like 2 membrane trafficking protein	0.0116	-1.509
TOMM20	translocase of outer mitochondrial membrane 20	0.000352	1.543
TOMM22	translocase of outer mitochondrial membrane 22	3.27E-05	1.675
TOMM6	translocase of outer mitochondrial membrane 6	0.000203	1.605
TOMM7	translocase of outer mitochondrial membrane 7	1.35E-05	1.906
TOP2B	topoisomerase (DNA) II beta	0.000062	-1.653
TOPBP1	topoisomerase (DNA) II binding protein 1	8.98E-05	-1.531
TP53BP1	tumor protein p53 binding protein 1	4.53E-05	-1.675
TP53RK	TP53 regulating kinase	0.000123	1.548
TPCN1	two pore segment channel 1	0.0133	-2.051
TPI1	triosephosphate isomerase 1	0.000861	1.623
TPR	translocated promoter region, nuclear basket protein	0.000842	-1.736
TPRKB	TP53RK binding protein	7.95E-05	1.76
TPT1	tumor protein, translationally-controlled 1	3.29E-06	2.098
TRAF1	TNF receptor associated factor 1	0.000763	-1.6
TRAF3	TNF receptor associated factor 3	0.00718	-1.603
TRAF3IP1	TRAF3 interacting protein 1	0.00342	-1.669
TRAM1	translocation associated membrane protein 1	0.000117	1.676
TRAPPC5	trafficking protein particle complex 5	0.00331	1.565
TRIM44	tripartite motif containing 44	0.00492	-1.539
TRIP11	thyroid hormone receptor interactor 11	0.00038	-2.601
TRMT112	tRNA methyltransferase 11-2 homolog (S. cerevisiae)	2.45E-07	2.104

TRMT44	tRNA methyltransferase 44 homolog (S. cerevisiae)	0.000822	-1.898
TRMT5	tRNA methyltransferase 5	7.71E-06	1.745
TRPS1	transcriptional repressor GATA binding 1	0.0075	-1.771
TRPT1	tRNA phosphotransferase 1	0.0136	1.51
TRRAP	transformation/transcription domain associated protein	0.000281	-1.757
TSC1	tuberous sclerosis 1	0.00016	-2.118
TSHZ1	teashirt zinc finger homeobox 1	0.00202	-1.578
TSHZ2	teashirt zinc finger homeobox 2	0.00599	-1.652
TSN	translin	8.45E-06	1.664
TSPAN3	tetraspanin 3	0.00163	1.517
TSPAN31	tetraspanin 31	5.55E-05	1.799
TSPAN6	tetraspanin 6	0.000678	1.827
TSPAN7	tetraspanin 7	0.0236	1.547
TSPO	translocator protein	0.000351	1.764
TSTD1	thiosulfate sulfurtransferase like domain containing 1	0.00321	1.579
TTBK2	tau tubulin kinase 2	0.0213	-1.687
TTC17	tetratricopeptide repeat domain 17	0.000138	-1.632
TTC28	tetratricopeptide repeat domain 28	0.00937	-1.585
TTC3	tetratricopeptide repeat domain 3	0.000209	-1.707
TTC36	tetratricopeptide repeat domain 36	0.00585	2.066
TTLL4	tubulin tyrosine ligase like 4	0.00627	-1.537
TUBA1A	tubulin alpha 1a	0.00221	1.861
TUBA1B	tubulin alpha 1b	0.000781	1.909
TUBA1C	tubulin alpha 1c	0.0171	1.611
TUBB	tubulin beta class I	0.000716	1.806
TUBB2A	tubulin beta 2A class IIa	0.0057	1.776
TUBB6	tubulin beta 6 class V	0.00292	2.122
TUBGCP6	tubulin gamma complex associated protein 6	0.000839	-1.582
TWISTNB	TWIST neighbor	0.000236	1.763
TXN	thioredoxin	0.000564	2.046

TXNDC12	thioredoxin domain containing 12	6.96E-07	1.828
TXNDC16	thioredoxin domain containing 16	0.0219	-1.633
TXNDC17	thioredoxin domain containing 17	0.000156	1.682
TXNDC9	thioredoxin domain containing 9	2.02E-06	1.749
TYW3	tRNA-yW synthesizing protein 3 homolog	0.0014	1.6
U2af1l4	U2 small nuclear RNA auxiliary factor 1-like 4	0.0045	1.584
U2SURP	U2 snRNP associated SURP domain containing	0.00115	-1.533
UACA	uveal autoantigen with coiled-coil domains and ankyrin repeats	9.39E-05	-2.235
UBA3	ubiquitin like modifier activating enzyme 3	5.87E-06	1.851
UBA52	ubiquitin A-52 residue ribosomal protein fusion product 1	0.000116	1.723
UBC	ubiquitin C	0.0028	-1.796
UBE2B	ubiquitin conjugating enzyme E2 B	0.000215	1.605
UBE2N	ubiquitin conjugating enzyme E2 N	1.21E-06	1.957
UBE2O	ubiquitin conjugating enzyme E2 O	0.000249	-1.584
UBE2W	ubiquitin conjugating enzyme E2 W (putative)	0.000613	1.548
UBL5	ubiquitin like 5	1.80E-07	2.079
UBLCP1	ubiquitin like domain containing CTD phosphatase 1	9.69E-05	1.51
UBN1	ubinuclein 1	0.00021	-1.569
UBR2	ubiquitin protein ligase E3 component n-recognin 2	4.18E-05	-1.764
UBR4	ubiquitin protein ligase E3 component n-recognin 4	0.000392	-1.701
UBR5	ubiquitin protein ligase E3 component n-recognin 5	0.000556	-1.624
UBXN8	UBX domain protein 8	8.06E-05	1.685
UCHL3	ubiquitin C-terminal hydrolase L3	0.000752	1.984
UCK2	uridine-cytidine kinase 2	0.00744	1.551
UHRF1BP1	UHRF1 binding protein 1	0.000499	-1.806
UHRF1BP1L	UHRF1 binding protein 1 like	0.0051	-1.505
UMPS	uridine monophosphate synthetase	0.0015	1.52
UNC13B	unc-13 homolog B	0.011	-1.496
UNKL	unkempt family like zinc finger	0.00168	-1.731
UPF2	UPF2 regulator of nonsense transcripts homolog (yeast)	7.68E-05	-1.815

UPRT	uracil phosphoribosyltransferase homolog	0.000232	1.574
UQCR10	ubiquinol-cytochrome c reductase, complex III subunit X	2.53E-05	1.782
UQCR11	ubiquinol-cytochrome c reductase, complex III subunit XI	0.000309	1.955
UQCRB	ubiquinol-cytochrome c reductase binding protein	0.000769	1.563
UQCRFS1	ubiquinol-cytochrome c reductase, Rieske iron-sulfur polypeptide 1	0.00195	1.655
UQCRH	ubiquinol-cytochrome c reductase hinge protein	6.57E-05	2.235
UQCRQ	ubiquinol-cytochrome c reductase complex III subunit VII	0.000243	1.789
USP19	ubiquitin specific peptidase 19	0.000686	-1.552
USP20	ubiquitin specific peptidase 20	0.00146	-1.661
USP24	ubiquitin specific peptidase 24	0.000544	-1.663
USP32	ubiquitin specific peptidase 32	0.00138	-1.669
USP34	ubiquitin specific peptidase 34	0.00142	-1.618
USP40	ubiquitin specific peptidase 40	0.000454	-1.515
USP42	ubiquitin specific peptidase 42	0.000199	-1.687
USP8	ubiquitin specific peptidase 8	3.12E-05	-1.557
UTRN	utrophin	0.000134	-2.098
UXT	ubiquitously expressed prefoldin like chaperone	9.05E-05	2.107
VAMP3	vesicle associated membrane protein 3	0.000658	1.498
VAMP5	vesicle associated membrane protein 5	0.00227	1.573
VBP1	VHL binding protein 1	1.47E-05	1.673
VDAC1	voltage dependent anion channel 1	0.000222	1.502
VDAC2	voltage dependent anion channel 2	6.31E-05	1.73
VDAC3	voltage dependent anion channel 3	0.000301	1.497
VEZF1	vascular endothelial zinc finger 1	0.000317	-1.547
VIMP	VCP interacting membrane selenoprotein	0.00112	1.528
VMP1	vacuole membrane protein 1	0.000136	1.696
VPS13D	vacuolar protein sorting 13 homolog D	0.000348	-1.747
VPS29	VPS29, retromer complex component	5.76E-06	1.938
VSTM5	V-set and transmembrane domain containing 5	0.00679	-1.898
VTI1A	vesicle transport through interaction with t-SNAREs 1A	0.0002	1.696

WBSCR22	Williams-Beuren syndrome chromosome region 22	0.000509	1.599
WDFY3	WD repeat and FYVE domain containing 3	0.000834	-1.861
WDR12	WD repeat domain 12	0.000473	1.786
WDR61	WD repeat domain 61	2.53E-05	1.695
WDR73	WD repeat domain 73	0.000149	-1.523
WDYHV1	WDYHV motif containing 1	6.13E-05	1.596
WHSC1	Wolf-Hirschhorn syndrome candidate 1	0.000436	-1.985
WIPF3	WAS/WASL interacting protein family member 3	0.00437	-2.307
WNK2	WNK lysine deficient protein kinase 2	0.0312	-1.622
WNT11	Wnt family member 11	0.032	-1.729
WRN	Werner syndrome RecQ like helicase	0.000407	-1.552
WSCD1	WSC domain containing 1	0.0285	-1.57
WWC1	WW and C2 domain containing 1	0.000126	-1.759
WWC2	WW and C2 domain containing 2	0.00043	-1.804
WWC3	WWC family member 3	0.00211	-1.679
XBP1	X-box binding protein 1	0.00157	1.503
XPC	XPC complex subunit, DNA damage recognition and repair factor	0.00251	-1.516
XRN1	5'-3' exoribonuclease 1	0.0314	-1.699
YAE1D1	Yae1 domain containing 1	3.94E-05	1.89
YEATS2	YEATS domain containing 2	4.66E-05	-1.834
YIPF5	Yip1 domain family member 5	4.33E-05	1.734
YPEL5	yippee like 5	0.00034	1.563
YRDC	yrdC N6-threonylcarbamoyltransferase domain containing	0.000358	1.656
YWHAQ	tyrosine 3-monooxygenase/tryptophan 5-monooxygenase activation protein theta	0.0021	1.6
ZAK	sterile alpha motif and leucine zipper containing kinase AZK	0.00105	-1.65
ZBTB18	zinc finger and BTB domain containing 18	0.000408	-1.687
ZBTB38	zinc finger and BTB domain containing 38	0.00118	-1.626
ZBTB40	zinc finger and BTB domain containing 40	0.00969	-1.621
ZBTB47	zinc finger and BTB domain containing 47	0.00174	-1.508
ZBTB49	zinc finger and BTB domain containing 49	0.00614	-1.641

ZBTB7A	zinc finger and BTB domain containing 7A	0.0016	-1.673
ZBTB8OS	zinc finger and BTB domain containing 8 opposite strand	0.000123	1.542
ZC3H11A	zinc finger CCCH-type containing 11A	6.34E-06	-1.853
ZC3H12A	zinc finger CCCH-type containing 12A	0.00483	-2.136
ZC3H12C	zinc finger CCCH-type containing 12C	0.00974	-1.909
ZC3H6	zinc finger CCCH-type containing 6	0.00016	-1.871
ZC3H7B	zinc finger CCCH-type containing 7B	0.000908	-1.567
ZCCHC11	zinc finger CCHC-type containing 11	0.000291	-1.681
ZCCHC24	zinc finger CCHC-type containing 24	0.0126	-1.51
ZCCHC6	zinc finger CCHC-type containing 6	0.000663	-1.722
ZCCHC8	zinc finger CCHC-type containing 8	0.000371	-1.513
ZCCHC9	zinc finger CCHC-type containing 9	0.000564	1.555
ZDHHC4	zinc finger DHHC-type containing 4	9.66E-05	1.515
ZEB1	zinc finger E-box binding homeobox 1	0.000152	-1.916
ZFAT	zinc finger and AT-hook domain containing	0.00141	-1.707
ZFC3H1	zinc finger C3H1-type containing	0.000641	-1.519
ZFP62	ZFP62 zinc finger protein	0.000368	-1.96
ZHX1	zinc fingers and homeoboxes 1	2.59E-05	-1.656
ZKSCAN1	zinc finger with KRAB and SCAN domains 1	7.11E-05	-2.2
ZMPSTE24	zinc metallopeptidase STE24	0.00658	1.612
ZMYND8	zinc finger MYND-type containing 8	0.000177	-2.009
ZNF106	zinc finger protein 106	0.00042	-1.629
ZNF135	zinc finger protein 135	0.00169	-1.719
ZNF142	zinc finger protein 142	0.00463	-1.563
ZNF182	zinc finger protein 182	0.00148	-1.774
ZNF184	zinc finger protein 184	0.000627	-1.504
ZNF197	zinc finger protein 197	0.0018	-1.682
ZNF202	zinc finger protein 202	0.00277	-1.736
ZNF214	zinc finger protein 214	0.0298	-1.703
ZNF217	zinc finger protein 217	0.00111	-1.664

ZNF235	zinc finger protein 235	0.000952	-1.819
ZNF236	zinc finger protein 236	0.000482	-1.954
ZNF292	zinc finger protein 292	0.000331	-1.894
ZNF318	zinc finger protein 318	3.02E-05	-2.045
ZNF32	zinc finger protein 32	0.00236	1.578
ZNF34	zinc finger protein 34	0.00273	-1.503
ZNF354B	zinc finger protein 354B	0.00112	-1.55
ZNF398	zinc finger protein 398	0.00908	-1.639
ZNF407	zinc finger protein 407	0.000486	-1.68
ZNF41	zinc finger protein 41	0.00661	-1.6
ZNF445	zinc finger protein 445	0.00413	-1.501
ZNF469	zinc finger protein 469	0.0305	-1.638
ZNF507	zinc finger protein 507	0.00132	-1.721
ZNF512B	zinc finger protein 512B	0.00179	-1.764
ZNF592	zinc finger protein 592	0.000806	-1.687
ZNF593	zinc finger protein 593	0.00864	1.623
ZNF605	zinc finger protein 605	0.00014	-2.23
ZNF608	zinc finger protein 608	1.76E-05	-2.394
ZNF609	zinc finger protein 609	0.0021	-1.68
ZNF638	zinc finger protein 638	2.92E-05	-1.84
ZNF644	zinc finger protein 644	7.46E-05	-1.619
ZNF646	zinc finger protein 646	0.0145	-1.523
ZNF653	zinc finger protein 653	0.00178	-1.767
ZNF677	zinc finger protein 677	0.00264	-1.557
ZNF710	zinc finger protein 710	0.00635	-1.511
ZNF74	zinc finger protein 74	0.00585	-1.532
ZNF75D	zinc finger protein 75D	0.000571	1.506
ZNF827	zinc finger protein 827	0.00589	-1.74
ZNF862	zinc finger protein 862	0.00536	-1.767
ZNRD1	zinc ribbon domain containing 1	0.00661	1.566

ZSCAN29	zinc finger and SCAN domain containing 29	3.82E-05	-1.858
ZSWIM6	zinc finger SWIM-type containing 6	0.00277	-1.548
ZSWIM8	zinc finger SWIM-type containing 8	4.26E-05	-1.636
ZZEF1	zinc finger ZZ-type and EF-hand domain containing 1	0.0039	-1.751

[1] Fold changes are up or down in PUFA pregnant animals compared to Control pregnant animals

Table S9. Differentially expressed genes in Control, not pregnant, low diet versus Control, pregnant, low diet

Symbol	Entrez Gene Name	p-value	Fold Change[1]
ACTG2	actin, gamma 2, smooth muscle, enteric	2.21E-06	7.582
ADAR	adenosine deaminase, RNA specific	0.000159	-3.524
AGRN	agrin	0.00024	-2.952
ATP8B4	ATPase phospholipid transporting 8B4 (putative)	0.000176	-7.661
BATF2	basic leucine zipper ATF-like transcription factor 2	0.000123	-5.834
BCL2L12	BCL2 like 12	0.000434	-3.001
BPI	bactericidal/permeability-increasing protein	0.000141	-13.675
C2	complement component 2	0.000779	-7.531
CA1	carbonic anhydrase 1	6.16E-08	195.89
CASP4	caspase 4	0.000849	-3.534
CD180	CD180 molecule	0.00027	-7.845
CLEC4F	C-type lectin domain family 4 member F	0.00012	-147.748
CMPK2	cytidine/uridine monophosphate kinase 2	1.04E-05	-15.1
CMTR1	cap methyltransferase 1	0.000373	-2.226
CNP	2',3'-cyclic nucleotide 3' phosphodiesterase	0.000742	-1.901
CXCL10	C-X-C motif chemokine ligand 10	0.000734	-12.771
DDX58	DEXD/H-box helicase 58	1.98E-05	-12.576
DHX58	DEXH-box helicase 58	2.72E-05	-6.92
DRAM1	DNA damage regulated autophagy modulator 1	0.000348	-2.482
DTX3L	deltex E3 ubiquitin ligase 3L	9.73E-05	-5.382
EIF2AK2	eukaryotic translation initiation factor 2 alpha kinase 2	4.28E-05	-5.154
EPSTI1	epithelial stromal interaction 1 (breast)	0.000021	-10.081
FOXS1	forkhead box S1	0.000197	-4.001
GBP2	guanylate binding protein 2	0.000206	-10.608
GRINA	glutamate ionotropic receptor NMDA type subunit associated protein 1	3.91E-05	-3.06
HERC5	HECT and RLD domain containing E3 ubiquitin protein ligase 5	3000000	-6.613
IFI44	interferon induced protein 44	3.63E-05	-17.049
IFI44L	interferon induced protein 44 like	2.01E-05	-18.853
IFI6	interferon alpha inducible protein 6	4.22E-05	-13.211
IFIH1	interferon induced with helicase C domain 1	0.000278	-5.783
IFIT1	interferon induced protein with tetratricopeptide repeats 1	0.000013	-46.835
IFIT2	interferon induced protein with tetratricopeptide repeats 2	3.12E-05	-41.012

IFIT3	interferon induced protein with tetratricopeptide repeats 3	1.71E-05	-34.367
IFIT5	interferon induced protein with tetratricopeptide repeats 5	3.34E-05	-4.655
IFITM1	interferon induced transmembrane protein 1	3.95E-05	-8.339
IFITM5	interferon induced transmembrane protein 5	0.0003	-4.673
IRF5	interferon regulatory factor 5	0.000423	-2.616
IRF7	interferon regulatory factor 7	4470000	-9.73
IRF9	interferon regulatory factor 9	1.31E-05	-6.985
ISG15	ISG15 ubiquitin-like modifier	6.17E-06	-73.22
ISG20	interferon stimulated exonuclease gene 20	4.96E-05	-48.493
JCHAIN	joining chain of multimeric IgA and IgM	0.000609	-47.853
LGALS3BP	galectin 3 binding protein	0.000275	-2.622
LGALS9	galectin 9	0.000118	-3.11
MB21D1	Mab-21 domain containing 1	0.000356	-3.49
MT-CYB	cytochrome b	0.000334	2.863
MX2	MX dynamin like GTPase 2	0.000015	-92.581
NTS	neurotensin	0.000234	-14.956
OAS2	2'-5'-oligoadenylate synthetase 2	0.00013	-30.033
PARP10	poly(ADP-ribose) polymerase family member 10	3.66E-05	-4.506
PARP12	poly(ADP-ribose) polymerase family member 12	9.17E-05	-4.818
PARP14	poly(ADP-ribose) polymerase family member 14	3.62E-05	-6.443
PARP9	poly(ADP-ribose) polymerase family member 9	8.81E-05	-3.864
PDCD1	programmed cell death 1	0.000305	-2.608
Pdlim3	PDZ and LIM domain 3	2.62E-08	5.019
PLEKHA4	pleckstrin homology domain containing A4	0.0004	-4.406
PNPT1	polyribonucleotide nucleotidyltransferase 1	3.49E-05	-6.163
PSMF1	proteasome inhibitor subunit 1	0.000306	-2.695
PTPRE	protein tyrosine phosphatase, receptor type E	0.000421	-3.904
RBCK1	RANBP2-type and C3HC4-type zinc finger containing 1	0.000311	-2.032
RSAD2	radical S-adenosyl methionine domain containing 2	2.11E-05	-55.667
RTP4	receptor transporter protein 4	4.62E-05	-8.017
SAMD9	sterile alpha motif domain containing 9	0.000151	-11.236
SCLY	selenocysteine lyase	0.000544	-2.49
SERPINI1	serpin family I member 1	4.86E-05	2.665
SLC15A3	solute carrier family 15 member 3	0.000661	-4.544
SLFN11	schlafen family member 11	8.51E-06	-14.212

STAT1	signal transducer and activator of transcription 1	0.000166	-3.987
STAT2	signal transducer and activator of transcription 2	0.000544	-2.65
TAP1	transporter 1, ATP binding cassette subfamily B member	0.000421	-2.866
TDRD7	tudor domain containing 7	0.000536	-2.417
TMEM140	transmembrane protein 140	0.000716	-2.924
TRANK1	tetratricopeptide repeat and ankyrin repeat containing 1	0.000364	-4.07
TREX1	three prime repair exonuclease 1	5.48E-05	-4.424
UBA7	ubiquitin like modifier activating enzyme 7	0.000054	-12.089
UBE2L6	ubiquitin conjugating enzyme E2 L6	0.000712	-2.894
UPB1	beta-ureidopropionase 1	0.000269	-56.239
USP18	ubiquitin specific peptidase 18	4.96E-06	-19.723
XAF1	XIAP associated factor 1	1.75E-05	-9.078
ZBP1	Z-DNA binding protein 1	5650000	-24.613
ZNFX1	zinc finger NFX1-type containing 1	0.000055	-5.825

[1] Fold changes are up or down in control, non-pregnant heifers compared to control, pregnant heifers

Table S10. Differentially expressed genes between PUFA supplemented, not pregnant, low diet versus PUFA supplemented, pregnant, low diet

Symbol	Entrez Gene Name	p-value	Fold Change[1]
ABCA10	ATP binding cassette subfamily A member 10	0.0011	2.486
ABCA3	ATP binding cassette subfamily A member 3	0.000477	-1.536
ABCA5	ATP binding cassette subfamily A member 5	0.00171	1.581
ABCA9	ATP binding cassette subfamily A member 9	0.00231	1.874
ABCB1	ATP binding cassette subfamily B member 1	0.00202	1.761
ABCC1	ATP binding cassette subfamily C member 1	0.000212	-1.899
ABCC3	ATP binding cassette subfamily C member 3	1.51E-05	-2.749
ABCG2	ATP binding cassette subfamily G member 2 (Junior blood group)	0.00189	1.681
ABHD1	abhydrolase domain containing 1	1.13E-05	-3.344
ABHD11	abhydrolase domain containing 11	0.000198	-1.772
ABLIM1	actin binding LIM protein 1	0.00216	1.528
ACKR4	atypical chemokine receptor 4	0.0003	-6.786
ACO2	aconitase 2	0.0025	-1.723
ACOX2	acyl-CoA oxidase 2	0.00592	1.584
ACSL5	acyl-CoA synthetase long-chain family member 5	0.00183	-1.662
ACSS1	acyl-CoA synthetase short-chain family member 1	0.00064	-1.956
ACSS2	acyl-CoA synthetase short-chain family member 2	7.07E-06	-2.754
ACYP1	acylphosphatase 1	0.00255	-1.547
ADAM23	ADAM metallopeptidase domain 23	0.00993	1.874
ADAMTS2	ADAM metallopeptidase with thrombospondin type 1 motif 2	0.00151	1.739
ADAMTS7	ADAM metallopeptidase with thrombospondin type 1 motif 7	0.00646	-1.783
ADAR	adenosine deaminase, RNA specific	1.3E-06	-3.717
ADCY5	adenylate cyclase 5	0.00673	1.63
AFAP1L1	actin filament associated protein 1 like 1	0.000941	2.258
AGO1	argonaute 1, RISC catalytic component	0.00167	1.538
AGO2	argonaute 2, RISC catalytic component	0.00525	-1.666
AGR2	anterior gradient 2, protein disulphide isomerase family member	7.68E-05	-3.151
AGRN	agrin	1.79E-05	-2.781
AHSG	alpha 2-HS glycoprotein	0.00351	-4.409
AIFM1	apoptosis inducing factor, mitochondria associated 1	0.000138	-1.786
AJUBA	ajuba LIM protein	0.00274	1.509
AK2	adenylate kinase 2	0.000784	-1.77

AKAP1	A-kinase anchoring protein 1	0.000981	-1.624
AKAP5	A-kinase anchoring protein 5	0.00971	1.599
AKR1A1	aldo-keto reductase family 1 member A1	0.00448	-1.678
ALAS1	5'-aminolevulinate synthase 1	5.05E-06	-2.704
ALDH2	aldehyde dehydrogenase 2 family (mitochondrial)	0.00709	-1.555
ALDH5A1	aldehyde dehydrogenase 5 family member A1	0.000113	-2.011
ALG3	ALG3, alpha-1,3- mannosyltransferase	1.5E-06	-2.143
ALOX12	arachidonate 12-lipoxygenase, 12S type	2.45E-05	-3.637
ALOX5AP	arachidonate 5-lipoxygenase activating protein	0.00324	-2.579
AMIGO1	adhesion molecule with Ig like domain 1	0.001	1.504
AMMECR1	Alport syndrome, mental retardation, midface hypoplasia and elliptocytosis chromosomal region gene 1	0.000514	-1.826
AMPD3	adenosine monophosphate deaminase 3	0.011	-2.267
ANGPTL1	angiopoietin like 1	0.00318	2.272
ANO3	anoctamin 3	0.011	1.862
ANO4	anoctamin 4	0.00216	2.402
ANXA1	annexin A1	0.00425	-2.129
APOLD1	apolipoprotein L domain containing 1	0.000349	2.553
APRT	adenine phosphoribosyltransferase	0.00322	-1.675
AQP1	aquaporin 1 (Colton blood group)	0.00128	2.054
AQP8	aquaporin 8	0.00653	-21.89
ARG2	arginase 2	0.000485	-2.29
ARHGAP15	Rho GTPase activating protein 15	0.00519	-1.978
ARHGAP44	Rho GTPase activating protein 44	0.011	1.658
ARHGEF10	Rho guanine nucleotide exchange factor 10	0.000189	1.793
ARHGEF4	Rho guanine nucleotide exchange factor 4	0.00212	2.055
ARSE	arylsulfatase E (chondrodysplasia punctata 1)	0.00884	-1.655
ARSK	arylsulfatase family member K	0.00591	1.553
ASNS	asparagine synthetase (glutamine-hydrolyzing)	0.00391	-1.61
ASPN	asporin	0.011	1.641
ATAD1	ATPase family, AAA domain containing 1	0.000113	-1.958
ATF3	activating transcription factor 3	0.00535	-2.357
ATG2B	autophagy related 2B	0.0014	1.591
ATIC	5-aminoimidazole-4-carboxamide ribonucleotide formyltransferase/IMP cyclohydrolase	0.00069	-1.658
ATP13A4	ATPase 13A4	0.00316	-2.049

ATP13A5	ATPase 13A5	0.00198	-2.008
ATP6V0A2	ATPase H+ transporting V0 subunit a2	0.000536	-2.216
ATP6V0A4	ATPase H+ transporting V0 subunit a4	6.14E-05	-4.024
ATP8B4	ATPase phospholipid transporting 8B4 (putative)	2.89E-05	-5.894
ATXN3	ataxin 3	0.00845	-1.638
B2M	beta-2-microglobulin	0.0014	-2.098
B3GALNT1	beta-1,3-N-acetylgalactosaminyltransferase 1 (globoside blood group)	7.58E-05	-3.961
BACE2	beta-site APP-cleaving enzyme 2	0.000357	-2.228
BAK1	BCL2 antagonist/killer 1	0.000173	-1.901
BATF2	basic leucine zipper ATF-like transcription factor 2	2.11E-05	-4.645
BCAM	basal cell adhesion molecule (Lutheran blood group)	0.00239	-1.621
BCAS4	breast carcinoma amplified sequence 4	0.00298	1.593
BCL2L1	BCL2 like 1	0.000957	-1.523
BCL2L12	BCL2 like 12	0.000441	-2.399
BCL2L14	BCL2 like 14	0.00161	-2.781
BCL2L15	BCL2 like 15	0.000014	-4.653
BCL6B	B-cell CLL/lymphoma 6B	0.000202	2.482
BDH1	3-hydroxybutyrate dehydrogenase, type 1	0.00194	-2.324
BHLHE40	basic helix-loop-helix family member e40	0.00161	-1.659
BOC	BOC cell adhesion associated, oncogene regulated	1.45E-05	-3.053
BPI	bactericidal/permeability-increasing protein	4.65E-05	-8.353
BPNT1	3'(2'), 5'-bisphosphate nucleotidase 1	0.000952	-1.725
BRI3BP	BRI3 binding protein	0.00154	-1.754
BYSL	bystin like	0.000285	-1.786
BZW2	basic leucine zipper and W2 domains 2	1.4E-06	-3.562
C14orf1	chromosome 14 open reading frame 1	2.02E-05	-2.208
C19orf66	chromosome 19 open reading frame 66	0.000323	-2.009
C1QL2	complement C1q like 2	0.00696	-8.076
C1RL	complement C1r subcomponent like	0.00151	-4.176
C2	complement component 2	0.00126	-4.382
C3AR1	complement component 3a receptor 1	0.00523	-2.293
C3orf38	chromosome 3 open reading frame 38	0.00237	-1.533
C5orf30	chromosome 5 open reading frame 30	0.00159	-2.073
CA2	carbonic anhydrase 2	0.00233	-3.304
CACHD1	cache domain containing 1	0.00325	-1.765

CAP1	adenylate cyclase associated protein 1	0.00277	-1.524
CAPG	capping actin protein, gelsolin like	0.00239	-1.606
CASP4	caspase 4	1.68E-05	-3.762
CASP8	caspase 8	4.12E-05	-2.926
CCDC158	coiled-coil domain containing 158	0.00275	1.64
CCDC58	coiled-coil domain containing 58	0.000609	-1.633
CCDC6	coiled-coil domain containing 6	0.00015	-1.679
CCL14	C-C motif chemokine ligand 14	0.00334	1.96
CCL8	C-C motif chemokine ligand 8	0.000109	-7.325
CCR7	C-C motif chemokine receptor 7	0.00132	-4.458
CCT6A	chaperonin containing TCP1 subunit 6A	0.00307	-1.627
CD109	CD109 molecule	0.00529	1.606
CD180	CD180 molecule	0.00135	-3.902
CD274	CD274 molecule	0.00342	-4.339
CD300LG	CD300 molecule like family member g	0.00235	2.031
CD3E	CD3e molecule	0.00221	-1.996
CD40	CD40 molecule	0.00919	-2.071
CD48	CD48 molecule	0.00117	-2.063
CD53	CD53 molecule	5.64E-05	-3.075
CD69	CD69 molecule	0.00313	-2.398
CD9	CD9 molecule	0.000706	-1.551
CD93	CD93 molecule	0.00217	2.312
CDADC1	cytidine and dCMP deaminase domain containing 1	0.00165	-1.742
CDC14A	cell division cycle 14A	0.00809	1.569
CDC42EP5	CDC42 effector protein 5	0.00555	1.928
CDC45	cell division cycle 45	0.000982	1.655
CDH1	cadherin 1	0.000331	-1.769
CDH17	cadherin 17	5.21E-06	-3.262
CDKN1A	cyclin dependent kinase inhibitor 1A	0.00244	-1.755
Cdkn1c	cyclin-dependent kinase inhibitor 1C (P57)	0.00163	1.973
CDKN2AIP	CDKN2A interacting protein	0.00382	-1.738
CEP85L	centrosomal protein 85 like	0.0076	1.693
CFB	complement factor B	8.09E-05	-4.882
CFD	complement factor D	0.00358	1.501
CFLAR	CASP8 and FADD like apoptosis regulator	0.00233	-1.554

CFTR	cystic fibrosis transmembrane conductance regulator	0.00791	-2.261
CGREF1	cell growth regulator with EF-hand domain 1	0.00369	1.558
CHCHD2	coiled-coil-helix-coiled-coil-helix domain containing 2	0.00159	-1.607
CHCHD6	coiled-coil-helix-coiled-coil-helix domain containing 6	0.0106	1.641
CHST15	carbohydrate (N-acetylgalactosamine 4-sulfate 6-O) sulfotransferase 15	0.00427	1.865
CIB1	calcium and integrin binding 1	0.000696	-1.637
CKS2	CDC28 protein kinase regulatory subunit 2	0.00206	-1.747
CLDN4	claudin 4	7.44E-05	-3.287
CLDN5	claudin 5	0.000437	1.913
CLEC10A	C-type lectin domain family 10 member A	7.05E-06	-6.051
CLEC12A	C-type lectin domain family 12 member A	6.48E-05	-2.9
CLEC14A	C-type lectin domain family 14 member A	0.00268	1.652
CLEC4F	C-type lectin domain family 4 member F	0.000425	-21.852
CLIC1	chloride intracellular channel 1	0.00135	-1.694
CLSTN2	calsyntenin 2	0.0045	-2.674
CMPK2	cytidine/uridine monophosphate kinase 2	2.61E-06	-8.965
CMTM7	CKLF like MARVEL transmembrane domain containing 7	2.32E-06	-1.876
CMTR2	cap methyltransferase 2	5.28E-05	-2.11
CNOT9	CCR4-NOT transcription complex subunit 9	4.24E-05	-1.523
CNP	2',3'-cyclic nucleotide 3' phosphodiesterase	0.00196	-1.614
CNTFR	ciliary neurotrophic factor receptor	0.00327	2.253
CNTN4	contactin 4	0.00386	2.365
COA7	cytochrome c oxidase assembly factor 7 (putative)	0.000878	-1.818
COQ2	coenzyme Q2, polyprenyltransferase	0.00503	-1.501
CORO2A	coronin 2A	0.000655	-1.692
CP	ceruloplasmin (ferroxidase)	0.00215	1.732
CPM	carboxypeptidase M	0.00668	-2.856
CPT1B	carnitine palmitoyltransferase 1B	0.00661	-1.587
CPT1C	carnitine palmitoyltransferase 1C	0.00633	1.713
CPXM2	carboxypeptidase X (M14 family), member 2	0.00113	-3.814
CR2	complement component 3d receptor 2	0.00774	2.546
CRACR2B	calcium release activated channel regulator 2B	0.000628	2.241
CRBN	cereblon	0.00473	-1.522
CRIM1	cysteine rich transmembrane BMP regulator 1	0.00472	-2.2
CRTAP	cartilage associated protein	0.00168	1.797

CRYAB	crystallin alpha B	0.00247	-2.404
CRYM	crystallin mu	0.000105	-5.032
CSDC2	cold shock domain containing C2	0.000213	-2.08
CSTB	cystatin B	0.000275	-1.723
CTNNAL1	catenin alpha like 1	0.000364	1.557
CTSL	cathepsin L	0.00481	-2.686
CTSS	cathepsin S	0.00425	-1.667
CTSV	cathepsin V	0.00344	-1.662
CUBN	cubilin	0.00461	-3.174
CWH43	cell wall biogenesis 43 C-terminal homolog	2.98E-05	-4.099
CXCL10	C-X-C motif chemokine ligand 10	0.000169	-8.88
CXCL11	C-X-C motif chemokine ligand 11	0.00477	-4.355
CXCL14	C-X-C motif chemokine ligand 14	0.00907	1.66
CXCL9	C-X-C motif chemokine ligand 9	0.00462	-4.068
CYB5R1	cytochrome b5 reductase 1	0.00703	-1.508
CYBRD1	cytochrome b reductase 1	0.000523	1.855
CYCS	cytochrome c, somatic	0.0016	-1.979
CYP24A1	cytochrome P450 family 24 subfamily A member 1	3.91E-06	-9.829
CYP51A1	cytochrome P450 family 51 subfamily A member 1	0.00277	-1.633
CYTH3	cytohesin 3	0.000228	1.637
DAG1	dystroglycan 1	0.00357	-1.52
DAO	D-amino acid oxidase	0.0107	-2.584
DAXX	death domain associated protein	0.000307	-1.629
DDC	dopa decarboxylase	0.00549	-2.728
DDX19B	DEAD-box helicase 19B	0.00166	-1.559
DDX39A	DEAD-box helicase 39A	0.000757	-1.648
DDX58	DEXD/H-box helicase 58	2.19E-06	-8.684
DEGS1	delta 4-desaturase, sphingolipid 1	0.0025	-1.689
DEPTOR	DEP domain containing MTOR-interacting protein	0.000233	2.165
DHCR24	24-dehydrocholesterol reductase	0.0007	-1.829
DHTKD1	dehydrogenase E1 and transketolase domain containing 1	0.000738	1.582
DHX58	DEXH-box helicase 58	1.34E-06	-5.787
DIMT1	DIM1 dimethyladenosine transferase 1 homolog	0.000311	-1.841
DIRC2	disrupted in renal carcinoma 2	0.000479	-1.816
DLAT	dihydrolipoamide S-acetyltransferase	0.00708	-1.506

DMGDH	dimethylglycine dehydrogenase	0.000135	2.201
DNAJB11	DnaJ heat shock protein family (Hsp40) member B11	0.000548	-1.732
DNASE2	deoxyribonuclease II, lysosomal	0.000213	-2.384
DPP3	dipeptidyl peptidase 3	3.01E-06	-2.011
DPY19L3	dpy-19 like 3 (C. elegans)	0.011	1.553
DRAM1	DNA damage regulated autophagy modulator 1	0.000843	-1.975
DTX3L	deltex E3 ubiquitin ligase 3L	6.82E-06	-4.672
DUSP26	dual specificity phosphatase 26 (putative)	0.00519	2.046
DUSP4	dual specificity phosphatase 4	1.31E-05	-3.011
DUSP5	dual specificity phosphatase 5	0.000101	-2.603
DZIP3	DAZ interacting zinc finger protein 3	0.000282	1.622
ECSCR	endothelial cell surface expressed chemotaxis and apoptosis regulator	0.00815	1.509
EEF2K	eukaryotic elongation factor 2 kinase	0.00451	1.535
EFEMP1	EGF containing fibulin like extracellular matrix protein 1	0.00133	1.681
EGFL7	EGF like domain multiple 7	0.00863	1.665
EGR3	early growth response 3	0.00159	-2.518
EHD4	EH domain containing 4	0.00228	-1.881
EIF1AD	eukaryotic translation initiation factor 1A domain containing	0.000181	-1.71
EIF2AK2	eukaryotic translation initiation factor 2 alpha kinase 2	1.47E-06	-4.656
EIF4A1	eukaryotic translation initiation factor 4A1	0.000455	-2.054
ELF3	E74 like ETS transcription factor 3	0.00701	-1.501
ELF4	E74 like ETS transcription factor 4	0.00138	-1.702
ELK3	ELK3, ETS transcription factor	0.0027	1.583
ELMOD1	ELMO domain containing 1	9.84E-05	-2.923
ELOVL1	ELOVL fatty acid elongase 1	0.00102	-1.531
ELOVL2	ELOVL fatty acid elongase 2	0.000571	-1.886
ELOVL6	ELOVL fatty acid elongase 6	0.00305	-1.596
EMB	embigin	6.98E-05	-3.321
EMC9	ER membrane protein complex subunit 9	0.00521	-1.588
EML1	echinoderm microtubule associated protein like 1	5.63E-05	1.596
EML2	echinoderm microtubule associated protein like 2	0.000264	-1.563
EMP1	epithelial membrane protein 1	0.000664	-2.402
ENPP1	ectonucleotide pyrophosphatase/phosphodiesterase 1	0.00135	-1.584
ENPP2	ectonucleotide pyrophosphatase/phosphodiesterase 2	0.000244	1.648
ENPP6	ectonucleotide pyrophosphatase/phosphodiesterase 6	0.00742	1.697

EPAS1	endothelial PAS domain protein 1	0.00192	-1.837
EPSTI1	epithelial stromal interaction 1 (breast)	2.17E-06	-7.334
ERAP2	endoplasmic reticulum aminopeptidase 2	0.00929	-1.6
ESAM	endothelial cell adhesion molecule	0.00604	1.539
EVI2A	ecotropic viral integration site 2A	0.00471	-1.837
EVI2B	ecotropic viral integration site 2B	0.00058	-2.463
F8	coagulation factor VIII	0.00374	1.566
FABP3	fatty acid binding protein 3	3.75E-05	-7.013
FAM111B	family with sequence similarity 111 member B	0.00119	-1.759
FAM124B	family with sequence similarity 124 member B	0.00176	1.961
FAM167A	family with sequence similarity 167 member A	0.00113	-2.516
FAM171A2	family with sequence similarity 171 member A2	0.00849	1.606
FAM210B	family with sequence similarity 210 member B	0.000275	1.507
FAM214A	family with sequence similarity 214 member A	0.00157	1.528
FAM3B	family with sequence similarity 3 member B	1.22E-06	-3.802
FAM46A	family with sequence similarity 46 member A	0.0073	-1.548
FAM46C	family with sequence similarity 46 member C	0.00964	-1.702
FAM69A	family with sequence similarity 69 member A	0.000329	-2.045
FAM92A1	family with sequence similarity 92 member A1	0.000146	1.819
FAP	fibroblast activation protein alpha	0.00305	1.809
FBXL7	F-box and leucine rich repeat protein 7	0.0113	1.501
FBXO10	F-box protein 10	0.00195	1.663
FCER1A	Fc fragment of IgE receptor Ia	0.00628	2.316
FCER1G	Fc fragment of IgE receptor Ig	0.00446	-2.248
FCGR1A	Fc fragment of IgG receptor Ia	0.0039	-2.421
FDFT1	farnesyl-diphosphate farnesyltransferase 1	0.000192	-1.548
FDPS	farnesyl diphosphate synthase	0.00011	-1.803
FGD2	FYVE, RhoGEF and PH domain containing 2	0.00658	-2.046
FGF1	fibroblast growth factor 1	0.000716	1.888
FGF13	fibroblast growth factor 13	0.00347	-1.902
FGF9	fibroblast growth factor 9	0.00034	-2.133
FGFBP1	fibroblast growth factor binding protein 1	4.74E-05	-9.582
FGFR1	fibroblast growth factor receptor 1	0.00759	1.657
FHL1	four and a half LIM domains 1	1.4E-06	1.649
FILIP1	filamin A interacting protein 1	0.0087	1.84

FLVCR2	feline leukemia virus subgroup C cellular receptor family member 2	5.14E-05	-1.91
FOSL1	FOS like 1, AP-1 transcription factor subunit	0.000014	-6.958
FOXS1	forkhead box S1	0.000108	-3.134
FRK	fyn related Src family tyrosine kinase	0.00301	-1.657
FSD2	fibronectin type III and SPRY domain containing 2	0.00632	1.673
FST	follistatin	0.00025	-2.775
FUT6	fucosyltransferase 6	5.64E-06	-3.609
FYB	FYN binding protein	0.011	-1.983
GABRP	gamma-aminobutyric acid type A receptor pi subunit	0.000527	-8.704
GALE	UDP-galactose-4-epimerase	0.000882	-1.92
GALNS	galactosamine (N-acetyl)-6-sulfatase	0.0075	-1.527
GALNT15	polypeptide N-acetylgalactosaminyltransferase 15	0.00758	2.026
GALNT18	polypeptide N-acetylgalactosaminyltransferase 18	0.01	1.528
GALNT3	polypeptide N-acetylgalactosaminyltransferase 3	0.00701	-1.584
GAPT	GRB2-binding adaptor protein, transmembrane	0.00181	-3.423
GARS	glycyl-tRNA synthetase	0.000021	-1.772
GBP1	guanylate binding protein 1	6.03E-05	-6.064
GBP2	guanylate binding protein 2	8.65E-06	-9.284
GBP5	guanylate binding protein 5	0.000201	-6.02
GBP6	guanylate binding protein family member 6	0.00148	-3.065
GCH1	GTP cyclohydrolase 1	0.00501	-1.76
GCK	glucokinase	0.00161	1.572
GCLC	glutamate-cysteine ligase catalytic subunit	2.77E-05	-2.125
GDA	guanine deaminase	9.04E-06	-4.734
GDAP2	ganglioside induced differentiation associated protein 2	9.94E-05	-1.995
GDF7	growth differentiation factor 7	0.00229	2.194
GDPD2	glycerophosphodiester phosphodiesterase domain containing 2	0.0103	-2.264
GDPGP1	GDP-D-glucose phosphorylase 1	3.37E-05	-2.153
GIMAP1-GIMAP5	GIMAP1-GIMAP5 readthrough	0.0027	-2.04
GIMAP7	GTPase, IMAP family member 7	0.00269	-2.024
GIPC2	GIPC PDZ domain containing family member 2	0.00323	-1.561
GJB2	gap junction protein beta 2	0.00888	-3.454
GLRX	glutaredoxin	0.00179	-1.685
GMPR	guanosine monophosphate reductase	0.00137	-3.293

GNPTAB	N-acetylglucosamine-1-phosphate transferase alpha and beta subunits	0.00057	1.556
GOT1	glutamic-oxaloacetic transaminase 1	8.29E-07	-2.603
GPHN	gephyrin	6.88E-05	-1.737
GPLD1	glycosylphosphatidylinositol specific phospholipase D1	7.86E-05	-7.808
GPR155	G protein-coupled receptor 155	0.00207	1.68
GPR65	G protein-coupled receptor 65	0.00428	-2.208
GPRC5A	G protein-coupled receptor class C group 5 member A	0.000017	-4.878
GPRC5C	G protein-coupled receptor class C group 5 member C	0.00214	-1.521
GRIK1	glutamate ionotropic receptor kainate type subunit 1	7.91E-05	3.211
GRINA	glutamate ionotropic receptor NMDA type subunit associated protein 1	1.09E-05	-2.609
GRIP2	glutamate receptor interacting protein 2	0.000265	-5.038
GRN	granulin	8.51E-05	-2.144
GSAP	gamma-secretase activating protein	0.00219	1.583
GSPT1	G1 to S phase transition 1	0.000225	-1.56
GSR	glutathione-disulfide reductase	4.84E-05	-1.747
GSS	glutathione synthetase	4.67E-06	-2.828
GSTM1	glutathione S-transferase mu 1	0.00331	-1.955
GTF2B	general transcription factor IIB	0.000262	-2.317
H2AFZ	H2A histone family member Z	0.00873	1.531
H2-T24	histocompatibility 2, T region locus 24	0.000549	-2.897
HACD3	3-hydroxyacyl-CoA dehydratase 3	3.52E-05	-1.822
HAS3	hyaluronan synthase 3	0.000232	2.289
HCAR3	hydroxycarboxylic acid receptor 3	0.000139	-3.771
HCRTR1	hypocretin receptor 1	0.00405	-2.409
HELZ	helicase with zinc finger	0.00639	-1.572
HERC5	HECT and RLD domain containing E3 ubiquitin protein ligase 5	6.17E-07	-4.804
HERC6	HECT and RLD domain containing E3 ubiquitin protein ligase family member 6	1.17E-06	-8.052
HES2	hes family bHLH transcription factor 2	0.00728	1.625
HEXB	hexosaminidase subunit beta	0.000126	-2.201
HIF1A	hypoxia inducible factor 1 alpha subunit	0.000145	-2.075
HIGD1A	HIG1 hypoxia inducible domain family member 1A	1.89E-05	-3.867
HIP1	huntingtin interacting protein 1	0.00618	1.641
HKDC1	hexokinase domain containing 1	0.000427	-3.448
HLA-B	major histocompatibility complex, class I, B	0.00167	-2.554
HLA-DMB	major histocompatibility complex, class II, DM beta	0.00477	-1.987

HMCN1	hemicentin 1	0.00253	2.452
HMGCR	3-hydroxy-3-methylglutaryl-CoA reductase	0.00631	-1.551
HMGCS1	3-hydroxy-3-methylglutaryl-CoA synthase 1	0.00239	-1.692
HMMR	hyaluronan mediated motility receptor	0.00263	1.581
HNF1B	HNF1 homeobox B	0.00332	-1.643
HOMER3	homer scaffolding protein 3	0.00415	1.537
HSD17B12	hydroxysteroid 17-beta dehydrogenase 12	2.52E-05	-2.31
HSH2D	hematopoietic SH2 domain containing	2.53E-05	-5.249
HSPA12B	heat shock protein family A (Hsp70) member 12B	0.000267	1.548
HSPA8	heat shock protein family A (Hsp70) member 8	0.00992	-1.563
HTRA3	HtrA serine peptidase 3	0.00307	1.73
ICA1L	islet cell autoantigen 1 like	0.00792	-1.815
IDI1	isopentenyl-diphosphate delta isomerase 1	9.24E-05	-1.953
IDO1	indoleamine 2,3-dioxygenase 1	1.95E-05	-18.108
IFI30	IFI30, lysosomal thiol reductase	0.00299	-1.642
IFI35	interferon induced protein 35	0.000035	-2.708
IFI44	interferon induced protein 44	3.46E-05	-8.29
IFI44L	interferon induced protein 44 like	6.79E-06	-10.255
IFI6	interferon alpha inducible protein 6	1.8E-06	-10.387
IFIH1	interferon induced with helicase C domain 1	2.13E-05	-5.098
IFIT1	interferon induced protein with tetratricopeptide repeats 1	1.48E-05	-15.616
IFIT2	interferon induced protein with tetratricopeptide repeats 2	1.94E-06	-24.668
IFIT3	interferon induced protein with tetratricopeptide repeats 3	2.58E-06	-17.982
IFIT5	interferon induced protein with tetratricopeptide repeats 5	8.81E-07	-4.295
IFITM1	interferon induced transmembrane protein 1	1.83E-06	-6.904
IFITM5	interferon induced transmembrane protein 5	3.57E-05	-4.067
IGDCC4	immunoglobulin superfamily DCC subclass member 4	0.00445	1.828
IGFBP7	insulin like growth factor binding protein 7	0.00154	1.571
IL18	interleukin 18	9.83E-05	-1.864
IL18BP	interleukin 18 binding protein	0.00157	-8.699
IL1RN	interleukin 1 receptor antagonist	0.00027	-2.619
IL23A	interleukin 23 subunit alpha	0.00343	-1.875
IL33	interleukin 33	0.000243	2.268
ILDR2	immunoglobulin like domain containing receptor 2	0.0111	1.799
INHBB	inhibin beta B subunit	0.00838	1.779

IRF3	interferon regulatory factor 3	2.99E-05	-1.963
IRF6	interferon regulatory factor 6	0.0019	-1.788
IRF7	interferon regulatory factor 7	4.73E-07	-6.892
IRF8	interferon regulatory factor 8	0.00732	-1.648
IRF9	interferon regulatory factor 9	2.71E-07	-6.135
ISG15	ISG15 ubiquitin-like modifier	1.4E-06	-28.248
ISG20	interferon stimulated exonuclease gene 20	3.62E-07	-44.33
ISLR2	immunoglobulin superfamily containing leucine rich repeat 2	8.42E-05	4.252
ITGA3	integrin subunit alpha 3	8.47E-05	-1.775
ITGB5	integrin subunit beta 5	0.0019	-2.076
ITGB6	integrin subunit beta 6	0.00525	-2.358
JAM2	junctional adhesion molecule 2	0.00457	1.669
JPH1	junctophilin 1	0.000883	-1.788
JTB	jumping translocation breakpoint	6.99E-05	1.56
KCNK1	potassium two pore domain channel subfamily K member 1	0.000213	-1.973
KCNK5	potassium two pore domain channel subfamily K member 5	0.00139	-1.911
KCNK7	potassium two pore domain channel subfamily K member 7	0.00393	-2.775
KCNMA1	potassium calcium-activated channel subfamily M alpha 1	0.000138	2.024
KCTD7	potassium channel tetramerization domain containing 7	0.0061	1.504
KIAA1147	KIAA1147	0.000289	1.676
KIAA1462	KIAA1462	0.00274	1.733
KIF17	kinesin family member 17	0.00162	1.705
KIF5C	kinesin family member 5C	1.19E-05	-6.532
KIT	KIT proto-oncogene receptor tyrosine kinase	4.81E-05	2.49
KITLG	KIT ligand	2.61E-05	2.175
KLK12	kallikrein related peptidase 12	0.00081	-2.236
KNG1	kininogen 1	3.67E-05	-10.392
KPNA2	karyopherin subunit alpha 2	0.00113	-1.745
KRT17	keratin 17	4.83E-06	-26.538
KRTCAP3	keratinocyte associated protein 3	0.00013	-1.786
LAMB3	laminin subunit beta 3	0.000368	-3.053
LAMP3	lysosomal associated membrane protein 3	0.00029	-3.153
Lao1	L-amino acid oxidase 1	0.0034	-5.12
LCAT	lecithin-cholesterol acyltransferase	0.000215	-2.001
LCORL	ligand dependent nuclear receptor corepressor like	0.00494	1.504

LDLR	low density lipoprotein receptor	0.00156	-1.697
LGALS3BP	galectin 3 binding protein	1.8E-06	-2.822
LGALS9	galectin 9	4.37E-05	-2.623
LGMN	legumain	0.00456	-1.998
LMCD1	LIM and cysteine rich domains 1	0.000678	1.776
LNX1	ligand of numb-protein X 1	0.00782	-1.59
LPAR3	lysophosphatidic acid receptor 3	0.005	-1.632
LRRC32	leucine rich repeat containing 32	0.00163	1.567
LRRC66	leucine rich repeat containing 66	3.82E-05	-5.013
LRRC8D	leucine rich repeat containing 8 family member D	0.000793	-1.644
LRRK2	leucine rich repeat kinase 2	0.0113	1.512
LSR	lipolysis stimulated lipoprotein receptor	1.76E-05	-1.673
LTV1	LTV1 ribosome biogenesis factor	0.000909	-1.587
LY6E	lymphocyte antigen 6 complex, locus E	3.29E-09	-3.725
LY6G6C	lymphocyte antigen 6 complex, locus G6C	0.000103	-3.75
LYNX1	Ly6/neurotoxin 1	9.44E-05	-3.049
LYPD6	LY6/PLAUR domain containing 6	1.24E-05	-2.894
LYSMD2	LysM domain containing 2	0.00453	-1.913
MAD2L1BP	MAD2L1 binding protein	0.00353	-1.534
MAD2L2	MAD2 mitotic arrest deficient-like 2 (yeast)	0.000131	-2.018
MAGEF1	MAGE family member F1	0.00114	1.529
MALT1	MALT1 paracaspase	7.07E-05	1.557
MAOA	monoamine oxidase A	0.000212	1.614
MAP2K3	mitogen-activated protein kinase kinase 3	3.07E-05	-1.63
MAP3K8	mitogen-activated protein kinase kinase kinase 8	0.00667	-2.016
MAP7D3	MAP7 domain containing 3	0.00412	1.592
MAPRE1	microtubule associated protein RP/EB family member 1	6.96E-05	-1.66
MARCH3	membrane associated ring-CH-type finger 3	0.000319	-2.435
MARS2	methionyl-tRNA synthetase 2, mitochondrial	0.000336	-1.73
MATK	megakaryocyte-associated tyrosine kinase	0.000105	-2.673
MATN4	matrilin 4	0.00134	-3.086
MB21D1	Mab-21 domain containing 1	2.55E-06	-3.839
MBTD1	mbt domain containing 1	0.00035	1.567
MBTPS1	membrane bound transcription factor peptidase, site 1	0.000379	1.625
MCEE	methylmalonyl-CoA epimerase	0.00219	-1.566

MCHR1	melanin concentrating hormone receptor 1	0.00419	-2.464
MCRIP2	MAPK regulated corepressor interacting protein 2	0.00702	-1.65
MECR	mitochondrial trans-2-enoyl-CoA reductase	0.000439	-1.779
METTL1	methyltransferase like 1	0.0112	-1.61
METTL17	methyltransferase like 17	0.00467	-1.545
METTL22	methyltransferase like 22	0.000556	1.628
MFNG	MFNG O-fucosylpeptide 3-beta-N-acetylglucosaminyltransferase	0.00409	1.726
MFSD4A	major facilitator superfamily domain containing 4A	0.00275	1.933
MGAT4A	mannosyl (alpha-1,3-)-glycoprotein beta-1,4-N-acetylglucosaminyltransferase, isozyme A	0.00134	-2.921
MGAT4C	MGAT4 family member C	0.00585	1.924
MISP	mitotic spindle positioning	8.94E-05	-2.466
MLKL	mixed lineage kinase domain-like	0.00455	-2.533
MME	membrane metallo-endopeptidase	0.00888	1.901
MMGT1	membrane magnesium transporter 1	0.00465	-1.527
MMP15	matrix metallopeptidase 15	0.00263	1.886
MMP28	matrix metallopeptidase 28	0.00153	1.621
MORC3	MORC family CW-type zinc finger 3	0.00347	-1.719
MOV10	Mov10 RISC complex RNA helicase	5.86E-05	-2.012
MPND	MPN domain containing	0.00365	1.585
MPV17L2	MPV17 mitochondrial inner membrane protein like 2	0.000457	-2.11
MPZL2	myelin protein zero like 2	7.91E-05	-2.76
MRI1	methylthioribose-1-phosphate isomerase 1	0.000715	-1.715
MRPL15	mitochondrial ribosomal protein L15	0.00106	-1.532
MRPL32	mitochondrial ribosomal protein L32	0.000674	-1.712
MRPS17	mitochondrial ribosomal protein S17	0.0091	-1.519
MRPS31	mitochondrial ribosomal protein S31	0.000865	-1.61
MS4A8	membrane spanning 4-domains A8	2.39E-06	-4.502
MSMO1	methylsterol monooxygenase 1	0.00554	-1.586
MST1R	macrophage stimulating 1 receptor	1.45E-05	-3.651
MTFP1	mitochondrial fission process 1	0.00422	-1.7
MTHFD2	methylenetetrahydrofolate dehydrogenase (NADP+ dependent) 2, methenyltetrahydrofolate cyclohydrolase	0.000516	-1.89
MTHFD2L	methylenetetrahydrofolate dehydrogenase (NADP+ dependent) 2-like	0.000708	-2.118
MTUS1	microtubule associated tumor suppressor 1	0.000189	-2.009
MX1	MX dynamin like GTPase 1	1.43E-06	-12.236

MX2	MX dynamin like GTPase 2	9.31E-06	-27.392
MXRA8	matrix remodeling associated 8	0.00198	1.794
MYBBP1A	MYB binding protein 1a	0.00102	-1.505
MYCT1	myc target 1	0.00207	1.944
MYH7B	myosin heavy chain 7B	0.00833	-2.041
MYL3	myosin light chain 3	0.000202	-3.164
MYO1A	myosin IA	0.00213	-1.977
MYO1D	myosin ID	0.00383	-1.565
MYO6	myosin VI	0.00288	-1.527
MYOC	myocilin	3.95E-06	2.198
MZB1	marginal zone B and B1 cell specific protein	0.00778	-2.422
NAMPT	nicotinamide phosphoribosyltransferase	0.000252	-1.837
NCDN	neurochondrin	0.00327	-2.183
NCLN	nicalin	0.000112	-1.747
NDFIP2	Nedd4 family interacting protein 2	0.00103	-1.649
NDUFA5	NADH:ubiquinone oxidoreductase subunit A5	0.00316	-1.531
NEDD4L	neural precursor cell expressed, developmentally down-regulated 4-like, E3 ubiquitin protein ligase	2.12E-05	-2.538
NEU1	neuraminidase 1 (lysosomal sialidase)	0.00904	-1.715
NEURL1B	neuralized E3 ubiquitin protein ligase 1B	0.00355	1.975
NFE2L3	nuclear factor, erythroid 2 like 3	0.00456	-1.688
NLRC4	NLR family CARD domain containing 4	0.00497	-2.241
NME2	NME/NM23 nucleoside diphosphate kinase 2	0.0054	-1.635
NMI	N-myc and STAT interactor	0.000426	-2.095
NOCT	nocturnin	0.000672	-2.115
NOL6	nucleolar protein 6	0.000191	-1.81
NOS1	nitric oxide synthase 1	0.000759	-3.108
NOTCH2	notch 2	0.00443	-1.601
NRP1	neuropilin 1	0.00698	1.648
NSDHL	NAD(P) dependent steroid dehydrogenase-like	0.000188	-1.579
NT5DC2	5'-nucleotidase domain containing 2	0.00262	1.515
NUP93	nucleoporin 93	2.17E-05	-2.717
NUPL2	nucleoporin like 2	0.00105	-1.562
NXPE2	neurexophilin and PC-esterase domain family member 2	0.00892	2.325
OAS1	2'-5'-oligoadenylate synthetase 1	3.62E-07	-10.089

OAS2	2'-5'-oligoadenylate synthetase 2	2.26E-06	-26.704
OPTN	optineurin	0.00311	-1.684
ORAI2	ORAI calcium release-activated calcium modulator 2	0.000558	-1.745
OSBPL6	oxysterol binding protein like 6	0.00123	1.52
OSBPL8	oxysterol binding protein like 8	0.00597	1.622
OSMR	oncostatin M receptor	0.00339	-1.905
OTUB2	OTU deubiquitinase, ubiquitin aldehyde binding 2	0.00559	-1.643
OVOL1	ovo like transcriptional repressor 1	0.000272	-2.14
P2RY13	purinergic receptor P2Y13	0.00508	-2.227
P2RY6	pyrimidinergic receptor P2Y6	0.00285	-2.626
PAFAH2	platelet activating factor acetylhydrolase 2	0.00981	-1.647
PAPD4	PAP associated domain containing 4	0.00231	-1.568
PAPD7	PAP associated domain containing 7	0.00154	-1.584
PARM1	prostate androgen-regulated mucin-like protein 1	0.00179	-1.916
PARP10	poly(ADP-ribose) polymerase family member 10	6.84E-06	-3.665
PARP12	poly(ADP-ribose) polymerase family member 12	4.01E-06	-4.393
PARP14	poly(ADP-ribose) polymerase family member 14	3.65E-06	-5.161
PARP8	poly(ADP-ribose) polymerase family member 8	0.00186	-1.739
PARP9	poly(ADP-ribose) polymerase family member 9	4.91E-06	-3.537
PATL1	PAT1 homolog 1, processing body mRNA decay factor	0.00003	-2.262
PCDH12	protocadherin 12	0.00289	2.473
PCK2	phosphoenolpyruvate carboxykinase 2, mitochondrial	2.66E-05	-2.684
PCYOX1	prenylcysteine oxidase 1	0.000607	1.574
PCYOX1L	prenylcysteine oxidase 1 like	0.00344	-1.546
PDCD1	programmed cell death 1	0.00648	-1.775
PDE12	phosphodiesterase 12	7.43E-05	-2.139
PDE6D	phosphodiesterase 6D	0.00183	-1.819
PDGFD	platelet derived growth factor D	0.00162	1.715
PDLIM4	PDZ and LIM domain 4	0.00051	1.576
PEBP4	phosphatidylethanolamine binding protein 4	0.00724	-1.582
PECAM1	platelet and endothelial cell adhesion molecule 1	0.000424	2.121
PFKFB4	6-phosphofructo-2-kinase/fructose-2,6-biphosphatase 4	0.000221	-2.214
PGK1	phosphoglycerate kinase 1	0.00178	-1.669
PGM2L1	phosphoglucomutase 2 like 1	0.00206	1.692
PGP	phosphoglycolate phosphatase	0.00675	-1.613

PHF10	PHD finger protein 10	0.000167	1.605
PI16	peptidase inhibitor 16	0.00401	2.635
PLA2G3	phospholipase A2 group III	2.09E-05	-9.375
PLA2G4A	phospholipase A2 group IVA	0.00127	-1.871
PLAUR	plasminogen activator, urokinase receptor	0.00111	-2.346
PLD1	phospholipase D1	0.00467	-2.184
PLEKHA4	pleckstrin homology domain containing A4	2.35E-05	-4.139
PLEKHA5	pleckstrin homology domain containing A5	0.00875	-1.673
PLET1	placenta expressed transcript 1	0.000803	-19.535
PLIN2	perilipin 2	8.71E-05	-2.091
PLK5	polo like kinase 5	0.000278	-11.322
PLP1	proteolipid protein 1	0.00395	2.004
PLS1	plastin 1	0.000883	-2.099
PLS3	plastin 3	0.00479	-1.504
PMVK	phosphomevalonate kinase	0.00152	-1.855
PNMAL1	paraneoplastic Ma antigen family like 1	0.00989	1.691
PNPT1	polyribonucleotide nucleotidyltransferase 1	1.17E-06	-5.437
POLH	DNA polymerase eta	0.000387	-1.606
POLK	polymerase (DNA) kappa	0.00956	-1.691
PPA1	pyrophosphatase (inorganic) 1	7.5E-06	-4.01
PPAN	peter pan homolog (Drosophila)	0.00403	-1.62
PPARGC1B	PPARG coactivator 1 beta	0.00105	-3.172
PPP1R16B	protein phosphatase 1 regulatory subunit 16B	0.00346	2.065
PQLC3	PQ loop repeat containing 3	0.00546	-2.051
PRDX1	peroxiredoxin 1	0.00187	-1.556
PREB	prolactin regulatory element binding	0.00118	-1.701
PROM2	prominin 2	0.0023	-2.289
PROSER2	proline and serine rich 2	0.000861	1.631
PRSS23	protease, serine 23	5.79E-06	-2.772
PRSS8	protease, serine 8	0.000235	-2.617
PSAT1	phosphoserine aminotransferase 1	0.00232	-2.706
PSMA2	proteasome subunit alpha 2	4.74E-05	-2.101
PSMA6	proteasome subunit alpha 6	0.0024	-1.528
PSMB10	proteasome subunit beta 10	0.00315	-1.7
PSMB2	proteasome subunit beta 2	0.00077	-1.576

PSMB8	proteasome subunit beta 8	0.00137	-1.769
PSMB9	proteasome subunit beta 9	0.000147	-2.271
PSMD14	proteasome 26S subunit, non-ATPase 14	0.00479	-1.55
PSME1	proteasome activator subunit 1	0.000778	-1.699
PSME2	proteasome activator subunit 2	0.000204	-1.88
PSMF1	proteasome inhibitor subunit 1	3.27E-05	-2.52
PSMG2	proteasome assembly chaperone 2	0.000571	-1.637
PSPH	phosphoserine phosphatase	0.0108	-3.044
PTGES2	prostaglandin E synthase 2	0.00274	-1.549
PTGS2	prostaglandin-endoperoxide synthase 2	0.00255	-2.812
PTP4A3	protein tyrosine phosphatase type IVA, member 3	0.00955	1.754
PTPRE	protein tyrosine phosphatase, receptor type E	7.91E-05	-3.383
PTPRJ	protein tyrosine phosphatase, receptor type J	0.000258	-2.004
PTPRO	protein tyrosine phosphatase, receptor type O	0.002	-3.158
PUM3	pumilio RNA binding family member 3	0.000515	-2.055
PUS1	pseudouridylate synthase 1	0.00211	-1.607
QPCT	glutaminyl-peptide cyclotransferase	0.0102	1.619
RAB25	RAB25, member RAS oncogene family	0.000426	-1.516
RAB27B	RAB27B, member RAS oncogene family	0.00164	-2.226
RAB3B	RAB3B, member RAS oncogene family	0.000286	-3.662
RAP1GAP	RAP1 GTPase activating protein	0.000647	-1.763
RAP2A	RAP2A, member of RAS oncogene family	0.000048	-2.109
RAPGEF4	Rap guanine nucleotide exchange factor 4	0.00777	1.883
RAPH1	Ras association (RalGDS/AF-6) and pleckstrin homology domains 1	0.00462	-1.936
RASEF	RAS and EF-hand domain containing	3.55E-06	-2.857
RASGRF2	Ras protein specific guanine nucleotide releasing factor 2	0.00648	1.654
RBCK1	RANBP2-type and C3HC4-type zinc finger containing 1	0.00042	-1.756
RBL2	RB transcriptional corepressor like 2	0.000118	1.528
RBM3	RNA binding motif (RNP1, RRM) protein 3	9.86E-06	-2.463
Rcan1	regulator of calcineurin 1	0.0029	-2.096
RCC2	regulator of chromosome condensation 2	0.00077	-1.528
RDH11	retinol dehydrogenase 11 (all-trans/9-cis/11-cis)	0.000504	-1.776
REEP5	receptor accessory protein 5	0.00776	-1.695
RELL2	RELT like 2	0.00089	1.525
RHBG	Rh family B glycoprotein (gene/pseudogene)	0.000129	-4.321

RIPK3	receptor interacting serine/threonine kinase 3	0.000785	-2.257
RNASE1	ribonuclease A family member 1, pancreatic	0.00603	-1.781
RNASE6	ribonuclease A family member k6	0.000293	-2.663
RNF114	ring finger protein 114	0.00206	-1.658
RNF19B	ring finger protein 19B	0.000403	-1.832
RORB	RAR related orphan receptor B	0.0064	1.705
RSAD2	radical S-adenosyl methionine domain containing 2	2.08E-05	-17.921
RTP4	receptor transporter protein 4	3.82E-06	-6.346
RUSC2	RUN and SH3 domain containing 2	0.00962	1.511
S100A12	S100 calcium binding protein A12	0.000227	-4.411
S100A2	S100 calcium binding protein A2	1.96E-07	-4.296
S100B	S100 calcium binding protein B	0.00876	1.795
SAA1	serum amyloid A1	0.00667	-3.594
SAMD9	sterile alpha motif domain containing 9	3.06E-05	-7.77
SASS6	SAS-6 centriolar assembly protein	0.00198	-2.03
SCARA5	scavenger receptor class A member 5	0.00542	1.505
SCD	stearoyl-CoA desaturase	0.000483	-2.03
SCG3	secretogranin III	0.00478	-2.776
SCIN	scinderin	0.00163	-2.459
SCLY	selenocysteine lyase	0.000818	-2.027
SCN4B	sodium voltage-gated channel beta subunit 4	0.00183	2.331
SCN7A	sodium voltage-gated channel alpha subunit 7	0.00487	1.977
SCRN1	secernin 1	0.00314	1.581
SEC11A	SEC11 homolog A, signal peptidase complex subunit	0.00632	-1.522
Sectm1b	secreted and transmembrane 1B	0.00405	-2.761
SELT	selenoprotein T	0.000254	-1.629
SEMA4B	semaphorin 4B	0.00349	-1.684
SENP3	SUMO1/sentrin/SMT3 specific peptidase 3	0.00198	-1.556
SERINC2	serine incorporator 2	0.000161	-2.081
SERPINA11	serpin family A member 11	0.00697	-2.089
SERPINB1	serpin family B member 1	0.00255	-1.578
SERTAD1	SERTA domain containing 1	0.000128	-2.336
SESN2	sestrin 2	0.000816	-1.871
SFN	stratifin	7.95E-06	-3.003
SFXN3	sideroflexin 3	0.00388	1.699

SGSM3	small G protein signaling modulator 3	0.000584	-1.623
SH3GL2	SH3 domain containing GRB2 like 2, endophilin A1	0.000233	-2.898
SH3TC2	SH3 domain and tetratricopeptide repeats 2	0.00856	1.852
SHE	Src homology 2 domain containing E	0.00144	2.007
SHISA2	shisa family member 2	0.00213	-4.605
SHISA5	shisa family member 5	1.94E-06	-3.362
SIGLEC1	sialic acid binding Ig like lectin 1	0.000098	-6.186
SLAMF7	SLAM family member 7	0.00328	-2.593
SLC11A2	solute carrier family 11 member 2	0.00539	-1.708
SLC12A2	solute carrier family 12 member 2	0.00833	-1.788
SLC15A3	solute carrier family 15 member 3	9.97E-05	-3.965
SLC16A1	solute carrier family 16 member 1	0.000251	-2.783
SLC18A2	solute carrier family 18 member A2	0.0034	1.598
SLC1A5	solute carrier family 1 member 5	4.67E-05	-2.447
SLC23A1	solute carrier family 23 member 1	0.000054	-2.131
SLC25A25	solute carrier family 25 member 25	0.00258	-1.823
SLC25A28	solute carrier family 25 member 28	0.00272	-1.739
SLC25A3	solute carrier family 25 member 3	0.00328	-1.521
SLC25A33	solute carrier family 25 member 33	1.54E-06	-2.261
SLC25A43	solute carrier family 25 member 43	5.47E-05	-1.744
SLC25A5	solute carrier family 25 member 5	3.77E-05	-2.67
SLC27A5	solute carrier family 27 member 5	0.00302	-2.209
SLC27A6	solute carrier family 27 member 6	0.00873	2.47
SLC2A1	solute carrier family 2 member 1	3.12E-05	-3.307
SLC2A3	solute carrier family 2 member 3	0.000233	1.753
SLC35B1	solute carrier family 35 member B1	0.00283	-1.526
SLC35C1	solute carrier family 35 member C1	0.000014	-2.038
SLC38A5	solute carrier family 38 member 5	0.00812	-1.891
SLC39A10	solute carrier family 39 member 10	0.00216	1.623
SLC40A1	solute carrier family 40 member 1	0.0029	-2.112
SLC44A3	solute carrier family 44 member 3	4.85E-05	-1.591
SLC44A4	solute carrier family 44 member 4	7.42E-05	-1.903
SLC45A2	solute carrier family 45 member 2	0.000235	-3.301
SLC4A11	solute carrier family 4 member 11	0.00256	-2.364
SLC5A1	solute carrier family 5 member 1	0.00117	-2.524

SLC5A6	solute carrier family 5 member 6	0.00208	-1.763
SLC5A8	solute carrier family 5 member 8	3.12E-05	-3.565
SLFN11	schlafen family member 11	9.41E-07	-9.322
SMC2	structural maintenance of chromosomes 2	0.00282	1.526
SMIM8	small integral membrane protein 8	0.000409	-1.813
SMPDL3B	sphingomyelin phosphodiesterase acid like 3B	0.00383	-2.831
SOAT1	sterol O-acyltransferase 1	2.46E-05	-3.851
SOSTDC1	sclerostin domain containing 1	0.00103	-7.816
SPARCL1	SPARC like 1	0.00547	1.551
SPOCK2	sparc/osteonectin, cwcv and kazal-like domains proteoglycan (testican) 2	0.00554	1.578
SPPL2A	signal peptide peptidase like 2A	0.00442	-1.511
SQLE	squalene epoxidase	0.00425	-1.645
SRPK2	SRSF protein kinase 2	0.00136	1.552
SRXN1	sulfiredoxin 1	8.86E-06	-2.474
SSX2IP	SSX family member 2 interacting protein	0.00458	-1.614
STARD13	StAR related lipid transfer domain containing 13	0.000495	1.527
STAT1	signal transducer and activator of transcription 1	5.86E-05	-3.225
STAT2	signal transducer and activator of transcription 2	0.000357	-2.247
STK17B	serine/threonine kinase 17b	0.00187	-2.243
STRADB	STE20-related kinase adaptor beta	0.000104	-1.769
STX3	syntaxin 3	0.000212	-1.817
STXBP6	syntaxin binding protein 6	0.00447	1.669
STYK1	serine/threonine/tyrosine kinase 1	0.00105	-2.382
SUSD1	sushi domain containing 1	0.00235	-2.079
SUSD2	sushi domain containing 2	0.00964	2.244
SYNC	syncoilin, intermediate filament protein	3.26E-05	1.735
TACC1	transforming acidic coiled-coil containing protein 1	0.00146	1.674
TACSTD2	tumor-associated calcium signal transducer 2	5.46E-06	-3.426
TAP1	transporter 1, ATP binding cassette subfamily B member	0.000102	-2.548
TBC1D16	TBC1 domain family member 16	0.0013	1.794
TBC1D31	TBC1 domain family member 31	0.000179	1.548
TBRG4	transforming growth factor beta regulator 4	0.000342	-1.571
TCAF2	TRPM8 channel associated factor 2	0.00253	-1.535
TCTN1	tectonic family member 1	0.000391	-1.605
TCTN2	tectonic family member 2	0.00671	-1.621

TDRD7	tudor domain containing 7	0.000168	-2.176
TEK	TEK receptor tyrosine kinase	0.000247	1.699
TEKT3	tektin 3	0.0101	1.846
TENM3	teneurin transmembrane protein 3	0.00232	1.919
TFG	TRK-fused gene	2.23E-05	-1.573
TFRC	transferrin receptor	0.00799	-1.958
TGFB2	transforming growth factor beta 2	0.00268	1.805
TGM3	transglutaminase 3	0.00747	-3.753
TGM5	transglutaminase 5	4.99E-07	-60.877
THBD	thrombomodulin	0.00637	1.531
THEM4	thioesterase superfamily member 4	0.00292	-1.649
THRA	thyroid hormone receptor, alpha	0.000394	1.562
TIE1	tyrosine kinase with immunoglobulin like and EGF like domains 1	0.000295	2.433
TIFA	TRAF interacting protein with forkhead associated domain	0.000018	-4.637
TIGAR	TP53 induced glycolysis regulatory phosphatase	4.46E-05	-2.986
TIMD4	T-cell immunoglobulin and mucin domain containing 4	0.000568	-4.191
TIMM23	translocase of inner mitochondrial membrane 23	0.00118	-1.589
TINAGL1	tubulointerstitial nephritis antigen like 1	0.00261	-2.547
TKT	transketolase	0.00193	-1.585
TLCD1	TLC domain containing 1	0.000429	-1.723
TM4SF1	transmembrane 4 L six family member 1	0.00168	-2.156
TM6SF2	transmembrane 6 superfamily member 2	0.000797	-2.436
TMEM107	transmembrane protein 107	0.00205	2.172
TMEM140	transmembrane protein 140	0.000375	-2.472
TMEM156	transmembrane protein 156	2.61E-05	-3.059
TMEM187	transmembrane protein 187	0.00931	1.509
TMEM236	transmembrane protein 236	0.00694	-5.661
TMEM268	transmembrane protein 268	9.65E-06	-2.189
TMEM30B	transmembrane protein 30B	0.00106	-1.524
TMEM40	transmembrane protein 40	2.43E-05	-16.328
TMEM45B	transmembrane protein 45B	0.000445	-2.856
TMEM64	transmembrane protein 64	3.71E-05	-2.521
TMEM79	transmembrane protein 79	4.14E-05	-1.743
TNFRSF12A	TNF receptor superfamily member 12A	0.00164	-2.766
TNFRSF6B	TNF receptor superfamily member 6b	0.000135	-7.585

TPBG	trophoblast glycoprotein	0.00386	1.797
TRANK1	tetratricopeptide repeat and ankyrin repeat containing 1	0.00005	-3.581
TRAT1	T cell receptor associated transmembrane adaptor 1	0.000508	-3.187
TREX1	three prime repair exonuclease 1	6.29E-07	-4.383
TRIM14	tripartite motif containing 14	0.000803	-2.455
TRIM21	tripartite motif containing 21	3.76E-05	-2.323
TRIM25	tripartite motif containing 25	7.67E-05	-2.203
TRIM26	tripartite motif containing 26	0.000127	-1.617
TRIM37	tripartite motif containing 37	0.000425	1.616
TRIM38	tripartite motif containing 38	0.000122	-1.817
TRIM5	tripartite motif containing 5	0.00039	-3.922
TRIM56	tripartite motif containing 56	0.00205	-1.863
TRMT13	tRNA methyltransferase 13 homolog	0.0053	-1.691
TRMT6	tRNA methyltransferase 6	0.002	-1.579
TSNAX	translin associated factor X	0.00113	-1.72
TSPAN12	tetraspanin 12	0.00327	1.515
TSTD1	thiosulfate sulfurtransferase like domain containing 1	0.000957	-1.622
TTC39B	tetratricopeptide repeat domain 39B	0.0059	-1.825
TUBA1C	tubulin alpha 1c	0.00473	-2.444
TUBA4A	tubulin alpha 4a	0.00429	-2.041
TUBB4A	tubulin beta 4A class IVa	2.19E-05	-2.984
TXN	thioredoxin	0.000227	-2.088
TXNDC16	thioredoxin domain containing 16	0.00478	1.711
UBA7	ubiquitin like modifier activating enzyme 7	1.09E-06	-10.643
UBD	ubiquitin D	0.0112	-1.933
UBE2L6	ubiquitin conjugating enzyme E2 L6	0.00112	-2.261
UCHL3	ubiquitin C-terminal hydrolase L3	0.00108	-1.888
UGCG	UDP-glucose ceramide glucosyltransferase	1.99E-05	-2.115
UMPS	uridine monophosphate synthetase	8.41E-05	-1.634
UNC45B	unc-45 myosin chaperone B	4.79E-05	-4.673
UNC93A	unc-93 homolog A (C. elegans)	0.000595	-3.626
UNC93B1	unc-93 homolog B1 (C. elegans)	0.00324	-1.556
UPB1	beta-ureidopropionase 1	0.00144	-9.625
UPK3BL	uroplakin 3B-like	0.00395	-3.458
UQCRFS1	ubiquinol-cytochrome c reductase, Rieske iron-sulfur polypeptide 1	0.00233	-1.596

USHBP1	USH1 protein network component harmonin binding protein 1	0.00613	1.556
USP18	ubiquitin specific peptidase 18	6.51E-07	-11.674
USP25	ubiquitin specific peptidase 25	0.00546	-1.57
USP43	ubiquitin specific peptidase 43	2.11E-05	-2.801
UXS1	UDP-glucuronate decarboxylase 1	0.00114	-1.583
VGLL1	vestigial like family member 1	7.28E-05	-7.381
VIL1	villin 1	0.00147	3.297
VIT	vitrin	0.00128	3.085
VLDLR	very low density lipoprotein receptor	1.01E-05	-2.789
VMP1	vacuole membrane protein 1	0.00131	-1.519
VNN2	vanin 2	0.00694	-2.981
VOPP1	vesicular, overexpressed in cancer, prosurvival protein 1	0.00461	1.527
VWCE	von Willebrand factor C and EGF domains	0.000956	-3.392
WARS	tryptophanyl-tRNA synthetase	0.000138	-2.683
WBSCR17	Williams-Beuren syndrome chromosome region 17	0.000482	-3.992
WDR12	WD repeat domain 12	0.000472	-1.734
WDR4	WD repeat domain 4	0.00867	-1.503
WDR62	WD repeat domain 62	0.00419	-1.689
Wfdc21	WAP four-disulfide core domain 21	0.00208	-8.164
WISP2	WNT1 inducible signaling pathway protein 2	0.000241	2.365
XAF1	XIAP associated factor 1	6.55E-07	-7.399
XRN2	5'-3' exoribonuclease 2	0.00259	-1.545
YARS	tyrosyl-tRNA synthetase	0.00018	-2.036
YPEL1	yippee like 1	0.0104	1.6
YTHDC2	YTH domain containing 2	0.00721	-1.585
ZBP1	Z-DNA binding protein 1	1.73E-06	-12.358
ZFYVE21	zinc finger FYVE-type containing 21	0.000384	1.512
ZNF521	zinc finger protein 521	0.00543	1.672
ZNF750	zinc finger protein 750	0.000042	-2.442
ZNFX1	zinc finger NFX1-type containing 1	2.83E-06	-5.06

[1] Fold changes are up or down in PUFA non-pregnant animals compared to PUFA pregnant animals

Table S11. Differentially expressed genes in PUFA, not pregnant, high diet versus PUFA, pregnant, high diet

Symbol	Entrez Gene Name	p-value	Fold Change[1]
AADAT	aminoadipate aminotransferase	0.00103	1.611
ABCC1	ATP binding cassette subfamily C member 1	0.00044	1.786
ABCD3	ATP binding cassette subfamily D member 3	7.71E-08	2.008
ABHD14B	abhydrolase domain containing 14B	3.70E-06	1.833
ABLIM2	actin binding LIM protein family member 2	0.00336	1.734
ABRACL	ABRA C-terminal like	2.04E-09	2.269
ACER3	alkaline ceramidase 3	0.00158	1.521
ACP5	acid phosphatase 5, tartrate resistant	2.24E-08	3.155
ACPP	acid phosphatase, prostate	0.0025	1.896
ACSF3	acyl-CoA synthetase family member 3	0.0058	-1.406
ACSL3	acyl-CoA synthetase long-chain family member 3	3.91E-08	1.812
ACSL4	acyl-CoA synthetase long-chain family member 4	4.32E-14	4.196
ACSS2	acyl-CoA synthetase short-chain family member 2	0.0013	1.951
ACY1	aminoacylase 1	2.75E-06	1.784
ADCY9	adenylate cyclase 9	0.00057	1.774
ADGRB2	adhesion G protein-coupled receptor B2	0.00319	-1.583
ADIPOR2	adiponectin receptor 2	0.00017	1.628
ADK	adenosine kinase	0.00105	1.508
ADSS	adenylosuccinate synthase	2.15E-07	1.887
AEBP1	AE binding protein 1	0.00357	-1.568
AFMID	arylformamidase	0.00033	2.61
AGTPBP1	ATP/GTP binding protein 1	0.00543	1.567
AIM1	absent in melanoma 1	4.35E-07	2.119
AKAP6	A-kinase anchoring protein 6	2.92E-09	4.865
AKR1B1	aldo-keto reductase family 1 member B	1.69E-07	2.691
ALDH18A1	aldehyde dehydrogenase 18 family member A1	4.24E-10	2.468

ALDH3A2	aldehyde dehydrogenase 3 family member A2	2.07E-06	1.835
ALG8	ALG8, alpha-1,3-glucosyltransferase	2.06E-09	1.8
AMACR	alpha-methylacyl-CoA racemase	0.00016	1.659
AMDHD2	amidohydrolase domain containing 2	5.26E-07	1.947
ANKIB1	ankyrin repeat and IBR domain containing 1	1.08E-10	2.158
ANKRD37	ankyrin repeat domain 37	8.51E-05	3.082
ANKS4B	ankyrin repeat and sterile alpha motif domain containing 4B	0.00545	3.903
ANPEP	alanyl aminopeptidase, membrane	2.65E-25	12.511
AP1S2	adaptor related protein complex 1 sigma 2 subunit	3.60E-07	2.081
AP3M1	adaptor related protein complex 3 mu 1 subunit	9.24E-09	1.827
AP3S2	adaptor related protein complex 3 sigma 2 subunit	3.25E-07	1.577
ARAF	A-Raf proto-oncogene, serine/threonine kinase	5.15E-08	1.879
ARHGAP18	Rho GTPase activating protein 18	0.00197	1.557
ARHGAP26	Rho GTPase activating protein 26	0.00457	1.552
ARHGEF2	Rho/Rac guanine nucleotide exchange factor 2	0.00252	-1.355
ARHGEF37	Rho guanine nucleotide exchange factor 37	0.00013	1.747
ARMT1	acidic residue methyltransferase 1	7.74E-07	1.93
ARSJ	arylsulfatase family member J	7.84E-05	1.683
ASGR2	asialoglycoprotein receptor 2	3.13E-16	6.236
ASPN	asporin	0.00033	2.013
ASRGL1	asparaginase like 1	1.69E-09	2.605
ATP1B3	ATPase Na+/K+ transporting subunit beta 3	0.00094	1.572
ATP5G1	ATP synthase, H+ transporting, mitochondrial Fo complex subunit C1 (subunit 9)	0.00383	1.566
B4GALNT2	beta-1,4-N-acetyl-galactosaminyltransferase 2	3.55E-05	1.871
BAIAP2L1	BAI1 associated protein 2 like 1	0.00028	1.555
BCAS1	breast carcinoma amplified sequence 1	0.00312	-1.643
BCKDHB	branched chain keto acid dehydrogenase E1 subunit beta	3.79E-06	1.976
BCO2	beta-carotene oxygenase 2	3.96E-06	2.101

BDH1	3-hydroxybutyrate dehydrogenase, type 1	5.89E-08	3.714
BDKRB1	bradykinin receptor B1	0.00076	2.421
BDKRB2	bradykinin receptor B2	0.00014	2.444
BEX2	brain expressed X-linked 2	0.00085	1.524
BICDL1	BICD family like cargo adaptor 1	0.00239	1.68
BSPRY	B-box and SPRY domain containing	1.25E-08	2.022
BYSL	bystin like	0.00436	1.55
C14orf2	chromosome 14 open reading frame 2	0.00046	1.581
C1orf122	chromosome 1 open reading frame 122	0.00321	1.618
C1orf21	chromosome 1 open reading frame 21	2.22E-07	2.076
C1QBP	complement C1q binding protein	0.00018	1.51
C2orf76	chromosome 2 open reading frame 76	5.47E-05	1.852
C4orf32	chromosome 4 open reading frame 32	1.17E-07	2.237
CAD	carbamoyl-phosphate synthetase 2, aspartate transcarbamylase, and dihydroorotase	0.0024	-1.419
CAMK2B	calcium/calmodulin dependent protein kinase II beta	6.57E-11	4.556
CCDC9	coiled-coil domain containing 9	0.00359	-1.429
CCNB1	cyclin B1	7.82E-06	3.558
CDC42	cell division cycle 42	0.00035	1.508
CDC42SE2	CDC42 small effector 2	0.00014	1.541
CDR2	cerebellar degeneration related protein 2	0.00014	1.631
CERS6	ceramide synthase 6	0.00015	2.173
CGN	cingulin	0.00299	-1.578
CGNL1	cingulin like 1	0.00555	-1.618
CGREF1	cell growth regulator with EF-hand domain 1	4.94E-05	1.875
CISD1	CDGSH iron sulfur domain 1	0.00012	1.502
CITED4	Cbp/p300 interacting transactivator with Glu/Asp rich carboxy-terminal domain 4	7.37E-06	3.061
CKS2	CDC28 protein kinase regulatory subunit 2	0.00547	1.618
CLIC6	chloride intracellular channel 6	0.00151	1.779

CLYBL	citrate lyase beta like	0.00486	-1.339
CMBL	carboxymethylenebutenolidase homolog	7.80E-09	2.759
CMTM7	CKLF like MARVEL transmembrane domain containing 7	0.00089	1.534
CNKSR3	CNKSR family member 3	4.09E-08	1.785
COMTD1	catechol-O-methyltransferase domain containing 1	5.58E-06	1.936
COPS9	COP9 signalosome subunit 9	3.36E-06	1.741
COQ10A	coenzyme Q10A	4.43E-08	2.107
COQ8A	coenzyme Q8A	0.00184	-1.488
COX11	COX11, cytochrome c oxidase copper chaperone	2.43E-05	1.673
CPAMD8	C3 and PZP like, alpha-2-macroglobulin domain containing 8	0.00171	-1.985
CREB3L4	cAMP responsive element binding protein 3 like 4	0.00256	2.795
CROCC	ciliary rootlet coiled-coil, rootletin	0.00391	-1.907
CRTC2	CREB regulated transcription coactivator 2	0.00049	-1.402
CSTB	cystatin B	0.00253	1.551
CTNNBIP1	catenin beta interacting protein 1	4.48E-05	1.712
CWH43	cell wall biogenesis 43 C-terminal homolog	0.00467	2.321
CYB5B	cytochrome b5 type B	1.08E-15	3.07
CYB5R4	cytochrome b5 reductase 4	3.24E-06	1.673
DACH1	dachshund family transcription factor 1	0.00515	-1.536
DBI	diazepam binding inhibitor, acyl-CoA binding protein	1.19E-10	2.375
DCAF17	DDB1 and CUL4 associated factor 17	0.00013	1.704
DCAKD	dephospho-CoA kinase domain containing	0.00133	1.516
DCHS1	dachsous cadherin-related 1	0.0037	-1.567
DDB2	damage specific DNA binding protein 2	0.00029	1.504
DDT	D-dopachrome tautomerase	2.27E-05	1.913
DENND1A	DENN domain containing 1A	0.00095	-1.465
DFFA	DNA fragmentation factor subunit alpha	3.8E-05	1.524
DGAT2	diacylglycerol O-acyltransferase 2	6.04E-05	2.822

DGKA	diacylglycerol kinase alpha	1.21E-07	2.173
DGKI	diacylglycerol kinase iota	6.44E-15	12.937
DHRS1	dehydrogenase/reductase 1	0.00021	1.525
DIMT1	DIM1 dimethyladenosine transferase 1 homolog	0.00206	1.65
DLG5	discs large MAGUK scaffold protein 5	0.00326	1.515
DMPK	dystrophia myotonica protein kinase	6.36E-11	4.028
DNAJA1	DnaJ heat shock protein family (Hsp40) member A1	0.0002	1.964
DNAJC16	DnaJ heat shock protein family (Hsp40) member C16	1.30E-06	1.618
DNAJC25	DnaJ heat shock protein family (Hsp40) member C25	0.00028	1.817
DPY19L4	dpy-19 like 4 (C. elegans)	1.81E-05	2.194
DYNC1I1	dynein cytoplasmic 1 intermediate chain 1	0.00013	1.885
EBAG9	estrogen receptor binding site associated, antigen, 9	1.39E-06	1.659
EEF1E1	eukaryotic translation elongation factor 1 epsilon 1	0.00011	1.76
EFNA1	ephrin A1	7.48E-05	1.901
EFNA4	ephrin A4	0.0007	1.813
ELOVL5	ELOVL fatty acid elongase 5	1.25E-09	2.087
ENDOG	endonuclease G	5.09E-06	1.777
ENPP1	ectonucleotide pyrophosphatase/phosphodiesterase 1	3.07E-09	2.244
ENPP3	ectonucleotide pyrophosphatase/phosphodiesterase 3	3.70E-14	5.585
ENPP5	ectonucleotide pyrophosphatase/phosphodiesterase 5 (putative)	1.72E-05	1.837
ENTPD1	ectonucleoside triphosphate diphosphohydrolase 1	0.00147	1.765
ENTPD2	ectonucleoside triphosphate diphosphohydrolase 2	1.53E-06	2.819
EPHX2	epoxide hydrolase 2	3.24E-05	2.278
EPN3	epsin 3	2.42E-09	3.196
ERBB2	erb-b2 receptor tyrosine kinase 2	0.00486	-1.497
ERO1A	endoplasmic reticulum oxidoreductase 1 alpha	4.25E-15	2.915
Esrra	estrogen related receptor, alpha	0.00018	1.54
ETV5	ETS variant 5	1.68E-05	-2.051

EVPL	envoplakin	0.00559	2.822
F2RL1	F2R like trypsin receptor 1	0.00187	1.654
FAM162A	family with sequence similarity 162 member A	3.00E-15	3.88
FAM20A	FAM20A, golgi associated secretory pathway pseudokinase	0.00064	-1.844
FAM213B	family with sequence similarity 213 member B	0.00091	1.564
FAM43A	family with sequence similarity 43 member A	2.58E-08	3.218
FAM84A	family with sequence similarity 84 member A	2.64E-05	2.217
FBXO2	F-box protein 2	0.00251	1.818
FITM2	fat storage inducing transmembrane protein 2	5.06E-05	1.938
FKBP5	FK506 binding protein 5	7.77E-05	1.719
FKBP9	FK506 binding protein 9	0.00101	1.546
FLNC	filamin C	0.00199	-1.673
Foxp1	forkhead box P1	0.00169	-1.591
FOXRED2	FAD dependent oxidoreductase domain containing 2	1.03E-18	10.421
FRMD4A	FERM domain containing 4A	0.00429	-1.564
FUT1	fucosyltransferase 1 (H blood group)	3.72E-07	5.636
G6PD	glucose-6-phosphate dehydrogenase	0.0028	1.671
GADD45G	growth arrest and DNA damage inducible gamma	1.87E-05	3.688
GAS2	growth arrest specific 2	0.00093	1.862
GATA2	GATA binding protein 2	0.0014	1.655
GBGT1	globoside alpha-1,3-N-acetylgalactosaminyltransferase 1	2.71E-11	2.682
GDAP1	ganglioside induced differentiation associated protein 1	0.00282	1.524
GDPD1	glycerophosphodiester phosphodiesterase domain containing 1	1.05E-17	4.154
GJB1	gap junction protein beta 1	5.94E-09	3.839
GJB3	gap junction protein beta 3	4.90E-08	2.693
GJB5	gap junction protein beta 5	3.11E-09	6.226
GK5	glycerol kinase 5 (putative)	1.63E-22	5.112
GLRX	glutaredoxin	0.00061	1.738

GM2A	GM2 ganglioside activator	1.33E-08	3.345
GMDS	GDP-mannose 4,6-dehydratase	0.00012	1.712
GNG10	G protein subunit gamma 10	0.00101	1.835
GNMT	glycine N-methyltransferase	1.14E-08	2.861
GNPNAT1	glucosamine-phosphate N-acetyltransferase 1	3.78E-05	1.69
GNS	glucosamine (N-acetyl)-6-sulfatase	1.18E-05	1.511
GPX2	glutathione peroxidase 2	0.00273	2.193
GRIA3	glutamate ionotropic receptor AMPA type subunit 3	6.39E-05	4.174
GRIN1	glutamate ionotropic receptor NMDA type subunit 1	7.29E-09	3.158
GSKIP	GSK3B interacting protein	7.05E-05	1.516
Gstt1	glutathione S-transferase, theta 1	1.75E-06	1.996
Gstt3	glutathione S-transferase, theta 3	6.32E-15	6.655
GUSB	glucuronidase beta	0.00035	1.531
HAUS5	HAUS augmin like complex subunit 5	0.00156	-1.356
HENMT1	HEN1 methyltransferase homolog 1	1.19E-08	3.915
HIP1R	huntingtin interacting protein 1 related	0.00158	-1.606
HIST2H4A	histone cluster 2 H4 family member a	4.09E-05	2.699
HK2	hexokinase 2	7.34E-06	3.24
HLCS	holocarboxylase synthetase	0.00011	1.784
HMGB3	high mobility group box 3	3.31E-05	-2.117
HMGN5	high mobility group nucleosome binding domain 5	0.00204	1.759
HNF4G	hepatocyte nuclear factor 4 gamma	0.00345	-1.567
HOMER1	homer scaffolding protein 1	0.00013	1.661
HSD17B10	hydroxysteroid 17-beta dehydrogenase 10	0.00015	1.618
IHH	indian hedgehog	5.46E-06	2.777
IKBKAP	inhibitor of kappa light polypeptide gene enhancer in B-cells, kinase complex-associated protein	1.77E-07	1.909
IKBKB	inhibitor of kappa light polypeptide gene enhancer in B-cells, kinase beta	0.00152	-1.455
IL17RC	interleukin 17 receptor C	0.00029	-1.497

ILF2	interleukin enhancer binding factor 2	0.00113	1.541
ILVBL	ilvB acetolactate synthase like	9.04E-14	2.822
ISOC1	isochorismatase domain containing 1	0.00087	1.72
ITGA11	integrin subunit alpha 11	0.00454	-1.827
ITGA2	integrin subunit alpha 2	0.0043	1.6
JAK3	Janus kinase 3	1.03E-19	4.738
JARID2	jumonji and AT-rich interaction domain containing 2	2.96E-13	3.644
KBTBD6	kelch repeat and BTB domain containing 6	0.00484	1.504
KDF1	keratinocyte differentiation factor 1	3.80E-07	2.049
KIAA0319L	KIAA0319 like	0.0044	-1.488
KIAA0513	KIAA0513	0.00218	-1.664
KIAA1324	KIAA1324	0.00423	-1.867
KIF17	kinesin family member 17	0.00039	-1.999
KLHDC4	kelch domain containing 4	0.00194	-1.429
KLHL42	kelch like family member 42	4.94E-05	2.022
KLHL7	kelch like family member 7	8.48E-05	1.71
KRT24	keratin 24	1.94E-10	5.89
KRT7	keratin 7	0.0058	-1.934
LCN15	lipocalin 15	0.0001	10.752
LDHA	lactate dehydrogenase A	1.46E-08	2.918
LIMK2	LIM domain kinase 2	1.38E-06	1.823
LINS1	lines homolog 1	7.38E-08	2.014
LPL	lipoprotein lipase	6.73E-09	6.858
LRAT	lecithin retinol acyltransferase (phosphatidylcholine--retinol O-acyltransferase)	5.21E-05	3.541
LRP1	LDL receptor related protein 1	0.0004	-1.821
LRP8	LDL receptor related protein 8	2.13E-05	2.377
LRRC38	leucine rich repeat containing 38	0.00272	-1.789
LRRC71	leucine rich repeat containing 71	0.00451	-1.877

LRRK1	leucine rich repeat kinase 1	0.00238	-1.521
LTF	lactotransferrin	9.24E-12	7.999
LYRM2	LYR motif containing 2	4.22E-05	1.708
LZTS1	leucine zipper tumor suppressor 1	0.00078	2.08
MANEAL	mannosidase endo-alpha like	0.00101	2.628
MAP1LC3A	microtubule associated protein 1 light chain 3 alpha	0.00038	1.664
MAP2	microtubule associated protein 2	0.00353	-1.853
MBOAT2	membrane bound O-acyltransferase domain containing 2	2.03E-09	2.269
MCCC2	methylcrotonoyl-CoA carboxylase 2	2.32E-10	2.896
MCEE	methylmalonyl-CoA epimerase	0.00234	1.547
MED25	mediator complex subunit 25	0.00347	-1.434
MESDC1	mesoderm development candidate 1	1.66E-07	1.791
METAP1	methionyl aminopeptidase 1	3.83E-06	1.726
METTL13	methyltransferase like 13	0.00016	1.601
MFSD4A	major facilitator superfamily domain containing 4A	0.00405	1.89
MICAL2	microtubule associated monooxygenase, calponin and LIM domain containing 2	1.85E-06	2.384
MIEN1	migration and invasion enhancer 1	7.18E-05	1.595
MIF	macrophage migration inhibitory factor (glycosylation-inhibiting factor)	0.00109	2.157
MKL1	megakaryoblastic leukemia (translocation) 1	0.00344	-1.464
MKNK1	MAP kinase interacting serine/threonine kinase 1	1.06E-05	1.543
MLPH	melanophilin	0.00166	2.269
MMP28	matrix metallopeptidase 28	5.51E-06	2
MOB3C	MOB kinase activator 3C	9.41E-05	1.723
MORN4	MORN repeat containing 4	0.00428	1.681
MOXD1	monooxygenase DBH like 1	6.51E-08	3.853
MPP7	membrane palmitoylated protein 7	5.46E-16	3.847
MRFAP1	Morf4 family associated protein 1	2.70E-07	1.74
MRPL19	mitochondrial ribosomal protein L19	1.96E-06	1.59

MRPS25	mitochondrial ribosomal protein S25	2.84E-05	1.701
MRPS36	mitochondrial ribosomal protein S36	6.19E-29	10.671
MSH2	mutS homolog 2	0.0006	1.621
MSX1	msh homeobox 1	4.82E-07	2.461
MSX2	msh homeobox 2	0.00493	1.8
MTERF2	mitochondrial transcription termination factor 2	0.00085	1.562
MTHFD2	methylenetetrahydrofolate dehydrogenase (NADP+ dependent) 2, methenyltetrahydrofolate cyclohydrolase	0.00049	1.836
MTURN	maturin, neural progenitor differentiation regulator homolog	0.00027	1.989
MUM1L1	MUM1 like 1	9.5E-05	2.16
MYO1B	myosin IB	5.81E-07	1.863
MYRF	myelin regulatory factor	0.00426	-1.657
MYRIP	myosin VIIA and Rab interacting protein	0.00527	-1.85
NAALAD2	N-acetylated alpha-linked acidic dipeptidase 2	2.6E-05	2.897
NCF1	neutrophil cytosolic factor 1	0.00425	-2.399
NDFIP2	Nedd4 family interacting protein 2	6.79E-05	1.794
NDRG1	N-myc downstream regulated 1	0.00514	-1.31
NDUFA4	NDUFA4, mitochondrial complex associated	0.00216	1.514
NDUFA5	NADH:ubiquinone oxidoreductase subunit A5	0.00028	1.668
NDUFAF5	NADH:ubiquinone oxidoreductase complex assembly factor 5	0.00016	2.199
NDUFB6	NADH:ubiquinone oxidoreductase subunit B6	0.00036	1.642
NDUFC2	NADH:ubiquinone oxidoreductase subunit C2	8.87E-05	1.67
NEBL	nebulette	3.68E-05	2.242
NETO2	neuropilin and tolloid like 2	3.33E-08	4.261
NIPSNAP3A	nipsnap homolog 3A	0.0001	1.713
NKD2	naked cuticle homolog 2	0.00206	-1.693
NLK	nemo like kinase	2.05E-06	1.612
NOL10	nucleolar protein 10	1.72E-05	1.535
NPL	N-acetylneuraminate pyruvate lyase	0.00334	-1.884

NPPC	natriuretic peptide C	2.2E-05	7.482
NSDHL	NAD(P) dependent steroid dehydrogenase-like	0.00018	1.563
NUDCD3	NudC domain containing 3	3.26E-09	1.831
NUP88	nucleoporin 88	1.37E-05	1.62
NUPR1	nuclear protein 1, transcriptional regulator	0.00163	2.294
NXNL2	nucleoredoxin-like 2	9.99E-26	7.552
NXT2	nuclear transport factor 2 like export factor 2	0.00267	1.536
ODC1	ornithine decarboxylase 1	1.80E-21	4.505
OGDH	oxoglutarate dehydrogenase	1.58E-14	3.156
OSTM1	osteopetrosis associated transmembrane protein 1	1.03E-09	2.172
P2RX3	purinergic receptor P2X 3	4.63E-08	3.852
PACS1	phosphofurin acidic cluster sorting protein 1	0.00241	-1.503
PACSIN1	protein kinase C and casein kinase substrate in neurons 1	3.12E-07	3.666
PANK1	pantothenate kinase 1	1.27E-05	2.128
PAQR4	progestin and adipoQ receptor family member 4	0.005	1.591
PAQR5	progestin and adipoQ receptor family member 5	0.00497	-1.899
PAQR8	progestin and adipoQ receptor family member 8	2.42E-06	2.127
PCCA	propionyl-CoA carboxylase alpha subunit	9.57E-08	1.848
PDCD11	programmed cell death 11	0.00448	-1.319
PDCD4	programmed cell death 4 (neoplastic transformation inhibitor)	1.49E-09	1.895
PDXK	pyridoxal (pyridoxine, vitamin B6) kinase	1.32E-05	2.106
PDZK1	PDZ domain containing 1	2.80E-14	4.81
PDZK1IP1	PDZK1 interacting protein 1	8.19E-09	4.975
PEPD	peptidase D	3.03E-09	3.265
PEX1	peroxisomal biogenesis factor 1	3.68E-08	1.773
PFKFB2	6-phosphofructo-2-kinase/fructose-2,6-biphosphatase 2	1.42E-12	3.894
PHGDH	phosphoglycerate dehydrogenase	3.05E-05	3.273
PHLDB1	pleckstrin homology like domain family B member 1	0.00289	-1.408

PIGF	phosphatidylinositol glycan anchor biosynthesis class F	0.00142	1.524
PIK3AP1	phosphoinositide-3-kinase adaptor protein 1	8.71E-05	1.762
PIK3CB	phosphatidylinositol-4,5-bisphosphate 3-kinase catalytic subunit beta	0.00047	1.606
PKIA	protein kinase (cAMP-dependent, catalytic) inhibitor alpha	0.003	1.756
PLA2G3	phospholipase A2 group III	6.50E-06	5.931
PLAC9	placenta specific 9	0.0029	1.567
PLEKHG1	pleckstrin homology and RhoGEF domain containing G1	8.27E-06	2.377
PLLP	plasmolipin	1.58E-11	3.258
PLS1	plastin 1	0.00443	1.822
POLD1	DNA polymerase delta 1, catalytic subunit	0.00337	-1.489
POLE2	DNA polymerase epsilon 2, accessory subunit	0.00112	1.757
POLR2E	RNA polymerase II subunit E	8.08E-05	1.505
POP5	POP5 homolog, ribonuclease P/MRP subunit	0.00067	1.553
PPM1H	protein phosphatase, Mg2+/Mn2+ dependent 1H	0.00102	1.674
PRDX2	peroxiredoxin 2	0.00012	1.575
PRKAB2	protein kinase AMP-activated non-catalytic subunit beta 2	0.00063	1.587
PRKAG2	protein kinase AMP-activated non-catalytic subunit gamma 2	9.82E-06	1.537
PRKCQ	protein kinase C theta	1.39E-05	2.613
PROCR	protein C receptor	0.00514	1.536
PRPSAP1	phosphoribosyl pyrophosphate synthetase associated protein 1	1.41E-07	1.82
PRSS35	protease, serine 35	0.00086	3.051
PSAT1	phosphoserine aminotransferase 1	1.78E-11	6.833
PTCH2	patched 2	0.00508	1.906
PTGER4	prostaglandin E receptor 4	2.69E-05	2.24
PTPN1	protein tyrosine phosphatase, non-receptor type 1	2.74E-07	1.851
RAB8B	RAB8B, member RAS oncogene family	0.00083	1.618
RABGEF1	RAB guanine nucleotide exchange factor 1	1.79E-09	1.58
RASA4	RAS p21 protein activator 4	0.00105	-1.837

RASAL1	RAS protein activator like 1	0.00513	1.708
RBM11	RNA binding motif protein 11	0.00376	1.593
RCN1	reticulocalbin 1	2.35E-11	2.871
RDH16	retinol dehydrogenase 16 (all-trans)	0.00286	-2.818
RFFL	ring finger and FYVE like domain containing E3 ubiquitin protein ligase	0.0012	1.63
RHOBTB3	Rho related BTB domain containing 3	6.75E-05	1.778
RIDA	reactive intermediate imine deaminase A homolog	7.77E-10	1.971
RNASEH2A	ribonuclease H2 subunit A	0.00248	1.562
RNF122	ring finger protein 122	3.26E-07	2.663
RNF125	ring finger protein 125	0.00326	1.593
RNF128	ring finger protein 128, E3 ubiquitin protein ligase	0.00166	1.638
RNF180	ring finger protein 180	7.96E-06	1.787
RNF183	ring finger protein 183	1.95E-05	2.037
RORC	RAR related orphan receptor C	0.0021	-1.862
RP2	retinitis pigmentosa 2 (X-linked recessive)	0.00067	1.531
RPAIN	RPA interacting protein	0.0033	1.569
RPS6KA5	ribosomal protein S6 kinase A5	0.00021	1.564
RSPRY1	ring finger and SPRY domain containing 1	7.65E-10	2.098
RTCA	RNA 3'-terminal phosphate cyclase	3.09E-05	1.534
RTKN	rhotekin	0.00239	-1.495
RTN1	reticulon 1	2.17E-07	2.412
RTP3	receptor transporter protein 3	0.00055	1.77
RWDD4	RWD domain containing 4	1.20E-10	2.119
S100A10	S100 calcium binding protein A10	2.52E-07	1.844
S100B	S100 calcium binding protein B	8.12E-05	2.414
SAA1	serum amyloid A1	2.35E-08	9.398
SAT1	spermidine/spermine N1-acetyltransferase 1	1.73E-05	1.812
SAT2	spermidine/spermine N1-acetyltransferase family member 2	0.00276	1.627

SBSPON	somatomedin B and thrombospondin type 1 domain containing	5.83E-06	2.404
SCNN1A	sodium channel epithelial 1 alpha subunit	0.0035	1.989
SDS	serine dehydratase	0.00056	2.628
SEC31A	SEC31 homolog A, COPII coat complex component	4.80E-06	1.726
SEL1L3	SEL1L family member 3	4.89E-05	1.655
SEMA3G	semaphorin 3G	0.00374	-1.984
SEMA4C	semaphorin 4C	0.00297	-1.421
SEMA4G	semaphorin 4G	0.00491	-1.659
Sep-04	septin 4	6.30E-06	1.94
SERPINA5	serpin family A member 5	0.00016	1.947
SGPP1	sphingosine-1-phosphate phosphatase 1	0.00209	1.628
SH3BP2	SH3 domain binding protein 2	0.00414	-1.519
SIDT1	SID1 transmembrane family member 1	0.00025	-3.088
SKAP1	src kinase associated phosphoprotein 1	0.00061	-1.56
SLC13A5	solute carrier family 13 member 5	1.65E-17	43.42
SLC16A13	solute carrier family 16 member 13	0.00045	2.024
SLC25A15	solute carrier family 25 member 15	0.00016	2.087
SLC25A29	solute carrier family 25 member 29	5.55E-06	1.955
SLC27A4	solute carrier family 27 member 4	7.70E-07	2.196
SLC28A3	solute carrier family 28 member 3	4.16E-08	3.605
SLC30A5	solute carrier family 30 member 5	0.00156	1.571
SLC31A1	solute carrier family 31 member 1	0.0052	1.549
SLC31A2	solute carrier family 31 member 2	4.46E-08	2.473
SLC37A4	solute carrier family 37 member 4	7.60E-11	2.601
SLC38A1	solute carrier family 38 member 1	0.00376	1.869
SLC38A3	solute carrier family 38 member 3	0.00143	-2.473
SLC43A3	solute carrier family 43 member 3	3.92E-11	3.597
SLC50A1	solute carrier family 50 member 1	2.06E-12	2.771

SLC6A9	solute carrier family 6 member 9	6.31E-05	1.725
SLC7A4	solute carrier family 7 member 4	1.25E-06	2.238
SLC7A8	solute carrier family 7 member 8	0.00241	2.176
SLCO4C1	solute carrier organic anion transporter family member 4C1	0.00169	2.054
SLIT3	slit guidance ligand 3	0.0036	-1.545
SMAD3	SMAD family member 3	0.00138	-1.452
SMIM14	small integral membrane protein 14	0.00022	1.651
SMOX	spermine oxidase	4.51E-07	2.377
SMPD3	sphingomyelin phosphodiesterase 3	0.00013	3.083
SMS	spermine synthase	0.00045	2.02
SMTNL2	smoothelin like 2	1.42E-12	2.648
SNPH	syntaphilin	0.0003	-2.079
SNX8	sorting nexin 8	0.00046	1.502
SOD3	superoxide dismutase 3, extracellular	0.00461	-1.598
SPC24	SPC24, NDC80 kinetochore complex component	2.82E-11	4.758
SPDEF	SAM pointed domain containing ETS transcription factor	3.53E-07	5.702
SPDYC	speedy/RINGO cell cycle regulator family member C	3.5E-05	2.192
SPHK1	sphingosine kinase 1	3.57E-15	4.584
SPIN1	spindlin 1	0.00032	1.526
SPOPL	speckle type BTB/POZ protein like	0.00451	1.56
SPOUT1	SPOUT domain containing methyltransferase 1	2.79E-07	1.923
SPPL3	signal peptide peptidase like 3	5.06E-06	1.703
SPRY2	sprouty RTK signaling antagonist 2	0.00034	-1.505
SPX	spexin hormone	0.00323	1.539
SRPX	sushi repeat containing protein, X-linked	5.06E-05	2.207
SRSF9	serine and arginine rich splicing factor 9	1.47E-09	1.775
SRXN1	sulfiredoxin 1	0.00159	1.815
SS18L1	SS18L1, nBAF chromatin remodeling complex subunit	7.88E-15	3.92

ST5	suppression of tumorigenicity 5	3.15E-05	-1.642
STAT6	signal transducer and activator of transcription 6	0.00175	-1.43
STC2	stanniocalcin 2	0.00281	-2.339
SUGP1	SURP and G-patch domain containing 1	0.00396	-1.314
Sult1a1	sulfotransferase family 1A, phenol-preferring, member 1	4.48E-09	2.757
SUSD2	sushi domain containing 2	0.00183	2.652
SV2B	synaptic vesicle glycoprotein 2B	0.00579	1.56
SVOPL	SVOP like	0.00069	-2.631
SYMPK	symplekin	0.0041	-1.32
SYNE4	spectrin repeat containing nuclear envelope family member 4	5.06E-08	2.746
SYNJ1	synaptojanin 1	0.0018	-1.549
TAF3	TATA-box binding protein associated factor 3	0.00182	-1.624
TBC1D30	TBC1 domain family member 30	5.48E-07	2.268
TBC1D9B	TBC1 domain family member 9B	0.0022	-1.339
TCAF2	TRPM8 channel associated factor 2	1.96E-16	2.97
TCOF1	treacle ribosome biogenesis factor 1	0.00464	-1.584
TDGF1	teratocarcinoma-derived growth factor 1	7.88E-09	14.37
TDRKH	tudor and KH domain containing	3.06E-05	1.723
TES	testin LIM domain protein	8.08E-06	1.641
TGFB2	transforming growth factor beta 2	0.00066	1.967
TIMM8A	translocase of inner mitochondrial membrane 8 homolog A (yeast)	0.00294	1.529
TK1	thymidine kinase 1	3.13E-06	1.857
TLN1	talin 1	0.00249	-1.445
TM7SF2	transmembrane 7 superfamily member 2	9.13E-06	1.964
TM9SF3	transmembrane 9 superfamily member 3	1.03E-06	1.723
TMED3	transmembrane p24 trafficking protein 3	4.52E-08	1.802
TMEM141	transmembrane protein 141	0.00028	1.634
TMEM163	transmembrane protein 163	0.00276	1.556

TMEM164	transmembrane protein 164	0.0002	1.594
TMEM165	transmembrane protein 165	0.00163	1.522
TMEM217	transmembrane protein 217	1.36E-05	3.321
TMEM229B	transmembrane protein 229B	0.00154	1.548
TMEM35A	transmembrane protein 35A	0.00088	1.833
TNKS1BP1	tankyrase 1 binding protein 1	0.0006	-1.491
TNS2	tensin 2	4.2E-05	-1.725
TNXB	tenascin XB	0.00498	-1.655
TOMM34	translocase of outer mitochondrial membrane 34	0.0002	1.684
TOR1B	torsin family 1 member B	5.24E-08	1.906
TP53I11	tumor protein p53 inducible protein 11	0.00173	1.776
TPCN1	two pore segment channel 1	2.09E-05	2.937
TPD52L1	tumor protein D52-like 1	0.00201	2.341
TPMT	thiopurine S-methyltransferase	2.60E-08	2.008
TRIM45	tripartite motif containing 45	0.00518	1.549
TRIM9	tripartite motif containing 9	1.65E-15	21.038
TRIT1	tRNA isopentenyltransferase 1	0.00076	1.61
TRMT44	tRNA methyltransferase 44 homolog (S. cerevisiae)	0.00513	-1.711
TSC22D3	TSC22 domain family member 3	0.00172	1.519
TST	thiosulfate sulfurtransferase	2.14E-08	3.132
TTC22	tetratricopeptide repeat domain 22	4.86E-08	2.877
TUBA8	tubulin alpha 8	0.00298	1.775
TXNDC16	thioredoxin domain containing 16	0.00578	1.706
TXNRD1	thioredoxin reductase 1	2.95E-23	5.688
UBIAD1	UbiA prenyltransferase domain containing 1	0.0003	1.572
UCP2	uncoupling protein 2	4.85E-08	2.768
UNC13B	unc-13 homolog B	0.00237	-1.615
UPK1B	uroplakin 1B	1.24E-09	8.532

USP12	ubiquitin specific peptidase 12	4.15E-05	1.561
USP46	ubiquitin specific peptidase 46	2.06E-05	1.633
VAMP4	vesicle associated membrane protein 4	7.28E-05	1.579
VDAC2	voltage dependent anion channel 2	0.00014	1.615
VMA21	VMA21 vacuolar H+-ATPase homolog (S. cerevisiae)	9.27E-06	1.562
VNN2	vanin 2	4.79E-07	5.774
WASHC3	WASH complex subunit 3	0.00119	1.501
WNT11	Wnt family member 11	1.31E-09	3.784
WSB2	WD repeat and SOCS box containing 2	6.02E-09	1.562
YBEY	ybeY metallopeptidase (putative)	0.00289	1.559
YEATS2	YEATS domain containing 2	0.00269	1.505
YIPF4	Yip1 domain family member 4	5.03E-05	1.518
ZCCHC12	zinc finger CCHC-type containing 12	0.00289	2.528
ZFP36L2	ZFP36 ring finger protein like 2	0.00157	-1.69
ZKSCAN1	zinc finger with KRAB and SCAN domains 1	0.00582	-1.726
ZMIZ1	zinc finger MIZ-type containing 1	0.00275	-1.579
ZNF277	zinc finger protein 277	0.00514	2.206
ZPBP2	zona pellucida binding protein 2	0.00073	1.854
ZYX	zyxin	0.00578	-1.385

[1] Fold changes are up or down in PUFA, not pregnant, high diet heifers compared with PUFA, pregnant, high diet

Table S12. Networks of biological interest from each comparison (generated using Ingenuity Pathway Analysis software)

Network ID	Top function	Molecules in comparison: Control, pregnant, low diet versus *n-3* PUFA, pregnant, Low diet	Score	Focus Molecules
12	Cellular Development, Cellular Growth and Proliferation, Embryonic Development	*ADAMTS2, Ap1, ARHGEF10, ARID4A , ARID4B, Cdc2, Cg, CHD7, Collagen(s), DUSP4, Ecm, EMB, Fibrinogen, FILIP1L, FSH, GLIS2, GPIIB-IIIA, Growth hormone, IFN alpha/beta, Integrin, Laminin ,LD ,Lh, LRRC32, LTBP4, Mapk, MARCH3, PGF, Pka catalytic subunit, POP5, SETD2, Smad, SOSTDC1, SPARCL1 ,Tgf beta*	21	17
16	Reproductive System Development and Function, Cellular Function and Maintenance, Organ Development	*ATAD5, BAZ1B, CATSPER1, CATSPER2, CATSPER3, CATSPER4, CATSPERB, CATSPERD, CATSPERG, CHIC2, DBT, GPX1, GPX7, GSTO1, HNF4A, KLF15, LIPT1, METTL12, MGST1, MGST3, MRPS35, MS4A8, ORMDL1, ORMDL2, SDHAF3, SDHAF4, SEC11A, SEC11C, SLC25A20, SPCS2, SRPRA, SSR2, TMEM208, TMEM30A, XPNPEP3*	18	15
23	Embryonic Development, Nervous System Development and Function, Organ Development	*ADAM10 ,ARHGEF4, B3GALNT1, CHP1, COL27A1, COL4A5, CST4, CTDNEP1, CTNNB1, Dgk, DGKQ, DIXDC1, DUSP11, DUSP14, FAM193B, FAM65C, FBXO45, HARS, MAPK8, MEGF8, MPZL2, NTN4, NXF1, PITPNM1, RAB7B, RAC3, RHOA, RNF144B, RWDD1, SLC45A2,T HBS2, TP73, Ubiquitin, ZNF605, ZNF644*	13	12

Network ID	Top functions	Molecules in network in comparison: Control, Non pregnant, High diet versus *n-3* PUFA, Not pregnant, High diet	Score	Focus molecules
1	Cellular Function and Maintenance, Nervous System Development and Function, Cell-To-Cell Signaling and Interaction	*alcohol group acceptor phosphotransferase, AP-3, AP3B1, AP3M1, AP3S2, APC (complex), ARAF, ATP6V1A, DMPK, DNAJA2, ENPP1, EPN3, FUCA1, Ikb, IKBKAP, LIMK2, MAST2, MOG, MRFAP1, NFkB (complex), P2RX3, PHGDH, RNF11, RNF115, RTKN, SCFD1, SEPT4, SLC37A4, TBKBP1, TK1, TPMT, TRIM9, TXNRD1, Ubiquitin, ZC3H12A*	50	29
6	Post-Translational Modification, Cell-To-Cell Signalling and Interaction, Cellular Assembly and	*ACO1, ACP5, AKAP6, Calcineurin protein(s), CAMK2B, CaMKII, Ck2, CNKSR3, Cpla2, Creb, EPHX2, FGL1, GM2A, HSD17B4, MAP2K1/2, Mek, MKNK1, NMDA Receptor, NSDHL, P38 MAPK, p70 S6k, Pdgf (complex), PDGF BB, phosphatase, Pkc(s), PPP2CB, PTPN1, PTPN23, SAT1, Shc, SNAPC4, SNPH, Sos, STX1A, SYNJ1*	27	19

	Organization			
12	Tissue Morphology, Immunological Disease, Organ Morphology	*ACTN3, ANKIB1, ARSJ, CAD, CDCA7L, CSNK1D, CSTB, ENPP4, FAM9B, FBP2, FOXP1, FOXP3, HLA-B, IL22, LENG8, LYRM2, METAP1, MKS1, MRC1, MYC, NCAPH2, NEBL, PLEKHG1, RTCA, SIVA1, SLC16A3, SMAD2, SMG9, SQRDL, T,TCF7L2, TDGF1, Tgf beta receptor, TNFRSF4, TRAF2*	21	16
**Network ID**	**Top functions**	**Molecules in network in comparison: *n-3* PUFA, Not pregnant, Low diet versus *n-3* PUFA, Not pregnant, High diet**	**Score**	**Focus molecules**
1	Cell Morphology, Cellular Assembly and Organization, Cell Cycle	*60S ribosomal subunit, AEBP2, ARHGAP18, EBAG9, EMP1, ERO1A, EZH1, FBLN5, GDE1, JARID2, KIF2A, LARP1B, LRAT, LRCH1, MOB1A, MORC3, NDP, NPL, PHF1, PTGES2, REEP5, RNF187, RPL29, PL32, RPL36, RPL37, RPL35A, RPL36A, RPL37A, RTCA, SHISA2, STK3, STK38L, TMEM109, Vegf*	43	33
5	Cellular Development, Embryonic Development, Hematological System Development and Function	*Arf, CHCHD6, CITED2, Ck2, CSPG5, DNA-methyltransferase, EEF1D, GATA2, HDAC5, HIF1A, HISTONE, ILF2, MAGED2, MRPL52, MTR, NAP1L1, NIP7, NPM1, ODC1, RPL10, RPL21, RPL22, RPL24, SLC25A3, SLC25A5, SLC5A8, SPDEF, TCERG1, TDRD7, TFRC, TMEM40, TMEM79, TOP1, TPGS1, UBA2*	39	31
19	Antimicrobial Response, Inflammatory Response, Infectious Diseases	*acid phosphatase, ACP2, ACP5, ACPP, ANPEP, ARG2, Caspase 3/7, Ciap, DTX3L, Hat, IFIT1, IFIT3, IFIT5, IFN alpha receptor, IFN alpha/beta, IFN Beta, Ifnar, IRF7, IRF9, Lpa receptor, LYPLA1, MHC Class I (complex), MICB, MX1, MX2, NUS1, OAS2 ,PARP9, PARP12, PARP14, Ras, RSAD2, RTP4, STAT2, XAF1*	25	24
**Network ID**	**Top functions**	**Molecules in network in comparison: *n-3* PUFA, pregnant, Low diet versus *n-3* PUFA, Pregnant, High diet**	**Score**	**Focus molecules**
11	Cellular Development, Cellular Growth and Proliferation, Embryonic Development	*ABCB1, ALMS1, ARID1B, ARID4A, ARID4B, Ctbp, ETV3, GPATCH8, Hdac, HISTONE, histone deacetylase, IFITM1, KAT6B, KCNRG, KDM4B, KMT2C, KMT2E, LACC1, LDL-cholesterol, MYB, MZF1, N-Cadherin, Npm, P38 MAPK, Pias, PLP2, ROBO2, SEMA3C, SLC1A5, TCF, TOP2A, ZBED6, ZEB1, ZNF202, ZNF292*	30	25
22	Tissue Development, Tissue Morphology, Embryonic Development	*AChR, AKR1A1, Ap1, BPIFB1, DGUOK, elastase, ESR2, estrogen receptor, GLRX3, GREB1, GRN, HAX1, HEBP1, HR, IgG2a, JINK1/2, LTF, MT-CO2, N-cor, NAPB, NFAT (complex), Nr1h, NR5A2, NRIP1, Nuclear factor 1, OOEP, PEPCK, PKIB, PRX, Rar, Ras*	19	19

		homolog, Rxr, thymidine kinase, Tnf receptor, UTRN		
**Network ID**	**Top functions**	**Molecules in network in interaction of diet and *n-3* PUFA on pregnant animals**	**Score**	**Focus molecules**
1	RNA Damage and Repair, Molecular Transport, RNA Trafficking	*BICD2, CH25H, CLNS1A, CYBA, DNASE2, EIF4A3, ERH, EXOSC8, FUNDC1, GSR, IKBKB, KANK1, KIF21A, MAGOH, MTERF3, NAA20, NCBP2, NEK8, NUDCD2, RBM11, SCAPER, SERF2, SERGEF, SMG1, SON, TELO2, THOC2, THOC3, TMEM50A, TSN, TXNDC17, UPF2, XRN1, ZC3H11A, ZCCHC8*	40	35
13	Cellular Assembly and Organization, Cellular Development, Cellular Growth and Proliferation	*ALMS1, ANKRD12, ARID4A, ARID4B, B3GALNT1, BET1, CHAD, DKKL1, EEA1, ERK1/2, GAREM1, GLIPR2, GOLGB1, GPATCH8, GRHL2, HOXB3, LACC1, NAE1, NAPB, NTN4, PLP2, Rab5, RFX2, SELENBP1, Sema3, SEMA3C, SEMA3F, SLC12A7, Snare, STX8, TPCN1, UBA3, VAMP3, VTI1A, WNK2*	31	31
24	Cell Signalling, Cellular Movement, Embryonic Development	*ABL2, ACOT9, Akt, ARHGAP5, ARHGAP12, ARHGAP35, ARHGAP44, CHCHD2, DGKD, DLC1, DNAH2, DNAH5, DNAH12, DOCK5, ECSCR, Erm, FKHR, HN1, INPP4A, LAMTOR2, NMB, NYAP1, palmitoyl-CoA hydrolase, PHIP, Plexin B, PLXNB1, PLXNB2, PPT1, RhoGap, STARD13, THEM4, TTC3, WWC1, WWC2, WWC3*	27	29
**Network ID**	**Top functions**	**Molecules in network in comparison: Control, not pregnant, low diet versus Control, pregnant, Low diet**	**Score**	**Focus molecules**
1	Antimicrobial Response, Inflammatory Response, Infectious Diseases	*AGRN,CNP,DDX58,DHX58,ERK1/2,IFI44,IFIH1,IFIT1,IFIT2,IFIT3,IFIT5,Ifn,IFN alpha/beta, IFN type 1,Ifnar,Interferon-α Induced,IRF,IRF5,IRF9,ISG15,ISG20,ISGF3,MB21D1,MHC Class I (complex),NTS,OAS2,PARP9,PTPRE,RSAD2,STAT2,STAT-1/2,Stat1-Stat2,TREX1,UBA7,UBE2L6*	54	24
2	Cell Signalling, Antimicrobial Response, Inflammatory Response	*Alp,BCL2L12,BPI,CD180,CXCL10,DTX3L,EIF2AK2,IFI44L,IFITM1,IFN Beta,Ifn gamma,Iga,IgG,IgG1,Igg3,IgG2a,IgG2b,Igm,IL12 (complex),Immunoglobulin,JAK,JCHAIN,LDL,LGALS9,NFkB (complex),PARP,PARP10,PARP12,PARP14,PDCD1,PNPT1,RBCK1,SCLY,Tgf beta,XAF1*	37	18
**Network ID**	**Top function**	**Molecules in comparison: *n-3* PUFA, not pregnant, low diet versus *n-3* PUFA, pregnant, low diet**	**Score**	**Focus Molecules**
3	Lipid Metabolism, Small Molecule Biochemistry, Vitamin and Mineral Metabolism	*ACSS2,Akt,ANGPTL1,CYP51A1,DHCR24,ECSCR,FDFT1,FDPS,FUT6,GCK,HMG CoA synthase,HMGCR,LYPD6,MBTPS1,Mir122a,b,MSMO1,NCDN,OSBPL8,PARM1,PDGF-DD,PDGFD,PPARGC1B,PSPH,PTP4A3,SLC39A10,SOAT1,Sphk,SQLE,Srebp,STARD13,TEK,THEM4,TIE1,TIMM23,UXS1*	38	29

5	Embryonic Development, Organismal Development, Tissue Development	*ALG3,BRI3BP,CD8,DUB,HLA-B,JPH1,KCNK1,KIR,MHC Class I (complex),MISP,MYO6,NCLN,NSDHL,OPTN,PLS3,PREB,Rab5,Rab11,RAB25,Rac,SASS6,SEC11A,SGSM3,SLC1A5,SLC38A5,TCTN1,TCTN2,TFRC,TLCD1,TRIM56,TRMT6,TXNDC16,UCHL3,USP25,USP43*	36	28
**Network ID**	**Top function**	**Molecules in comparison: *n-3* PUFA, not pregnant, high diet versus *n-3* PUFA, pregnant, high diet**	**Score**	**Focus Molecules**
1	Developmental Disorder, Hereditary Disorder, Metabolic Disease	*ACY1,ALDH18A1,ARHGAP18,C1QBP,COX11,cytochrome-c oxidase,EBAG9,EPN3,ERO1A,Esrra,Foxp1,Gstt1,HLCS,HSD17B10,IL-2R,IL17RC,LTF,MCCC2,MFSD4A,MHC Class I (complex),Mitochondrial complex 1,MKL1,MRPL19,MRPS25,NADH dehydrogenase,NDUFA4,NDUFA5,NDUFB6,NPL,OGDH,PCCA,PHGDH,RNF125,TST,Vegf*	45	29
2	Cell Death and Survival, Cellular Function and Maintenance, Embryonic Development	*14-3-3,Adaptor protein 1,AEBP1,alcohol group acceptor phosphotransferase,ANPEP,AP1S2,ARAF,collagen,DENND1A,DGKA,DGKI,DMPK,ENaC,ERK1/2,GTPase,LIMK2,NDFIP2,NPPC,PEPD,PIGF,PLS1,PRKCQ,Rab5,RABGEF1,Raf,RASA4,RCN1,RFFL,SEMA4C,SGPP1,SLC43A3,SPRY2,TNS2,TPCN1,TPMT*	38	26
10	Embryonic Development, Nervous System Development and Function, Organismal Development	*ADRB,Ap1,ASGR2,BCAS1,C1q,Cbp/p300,CD3,CMBL,Creb,CREB3L4,CROCC,Dynamin,Hdac,HISTONE,ILF2,JAK,JAK3,Mek,MSX1,MSX2,NAALAD2,NETO2,NFAT (complex),POLE2,PTPN1,Ras,RDH16,RNASEH2A,RORC,SMS,STAT6,STAT5a/b,Sult1a1,TRIM45,ZKSCAN1*	28	21
